# Photoredox-catalyzed direct formylation of alkenes to access α,β-unsaturated aldehydes

**DOI:** 10.1039/d6sc05707a

**Published:** 2026-09-18

**Authors:** Qi Jiang, Ruixiang Wang, Junhua Xu, Mingyou Hu, Xin-Hua Duan, Le Liu

**Affiliations:** a School of Chemistry, Xi'an Key Laboratory of Sustainable Energy Material Chemistry, Xi'an Jiaotong University Xi'an 710049 China le.liu@xjtu.edu.cn

## Abstract

α,β-Unsaturated aldehydes are ubiquitous and versatile synthetic building blocks in organic synthesis. Nevertheless, the direct construction of such skeletons *via* site-selective β-formylation of readily available alkenes still remains challenging and largely unexplored. Herein, we disclose a visible-light photoredox-catalyzed selective β-formylation strategy of various alkenes, employing bench-stable α-chloro-*N*-methoxyphthalimide as a practical formyl radical precursor. This mild photoredox protocol delivers diverse α,β-unsaturated aldehydes with broad substrate generality toward electron-rich/deficient, sterically hindered, cyclic and heterocyclic olefins, and is suitable for late-stage modification of complex molecules. Mechanistic studies confirm a radical-polar crossover catalytic cycle involving formyl radical addition and carbocation induced elimination.

## Abstract 2

A visible-light photoredox-catalyzed selective β-formylation of alkenes is developed for facile access to diverse α,β-unsaturated aldehydes using a bench-stable formyl radical precursor through a radical-polar crossover catalytic cycle.

## Graphical abstract

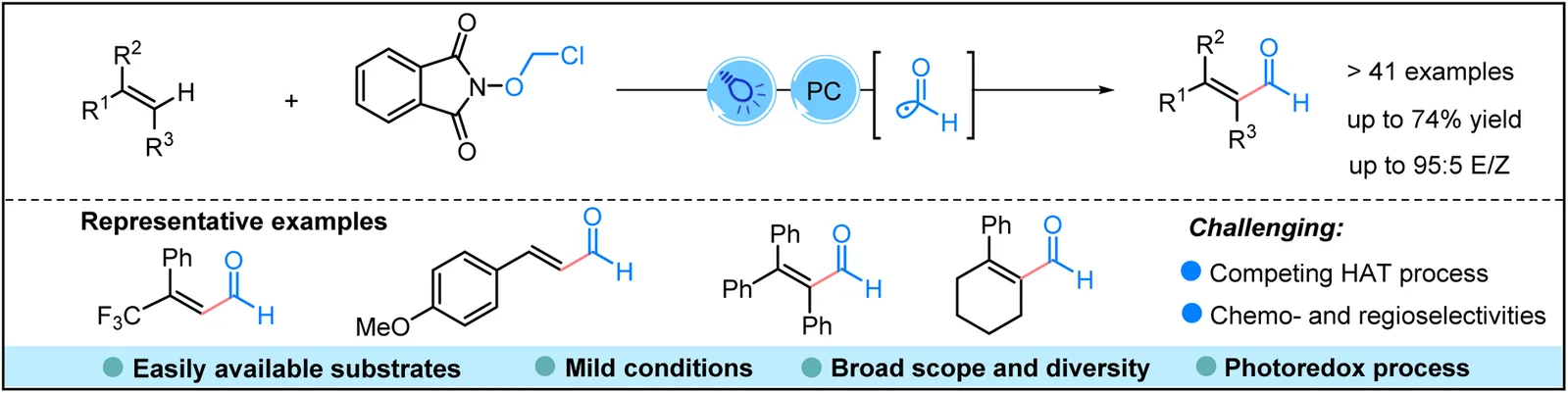

## Introduction

α,β-Unsaturated aldehydes are pivotal structural motifs across agrochemical, pharmaceutical and fine chemical fields (Fig. 1A).^1–4^ The conjugated enal system serves as a versatile handle for diverse transformations, including Michael additions, cycloadditions, and hydrogenations, enabling the rapid construction of molecular complexity.^5–7^ Consequently, the development of efficient, selective, and practical synthetic routes to α,β-unsaturated aldehydes has remained a longstanding goal in organic synthesis.^8–12^

**Fig. 1:**
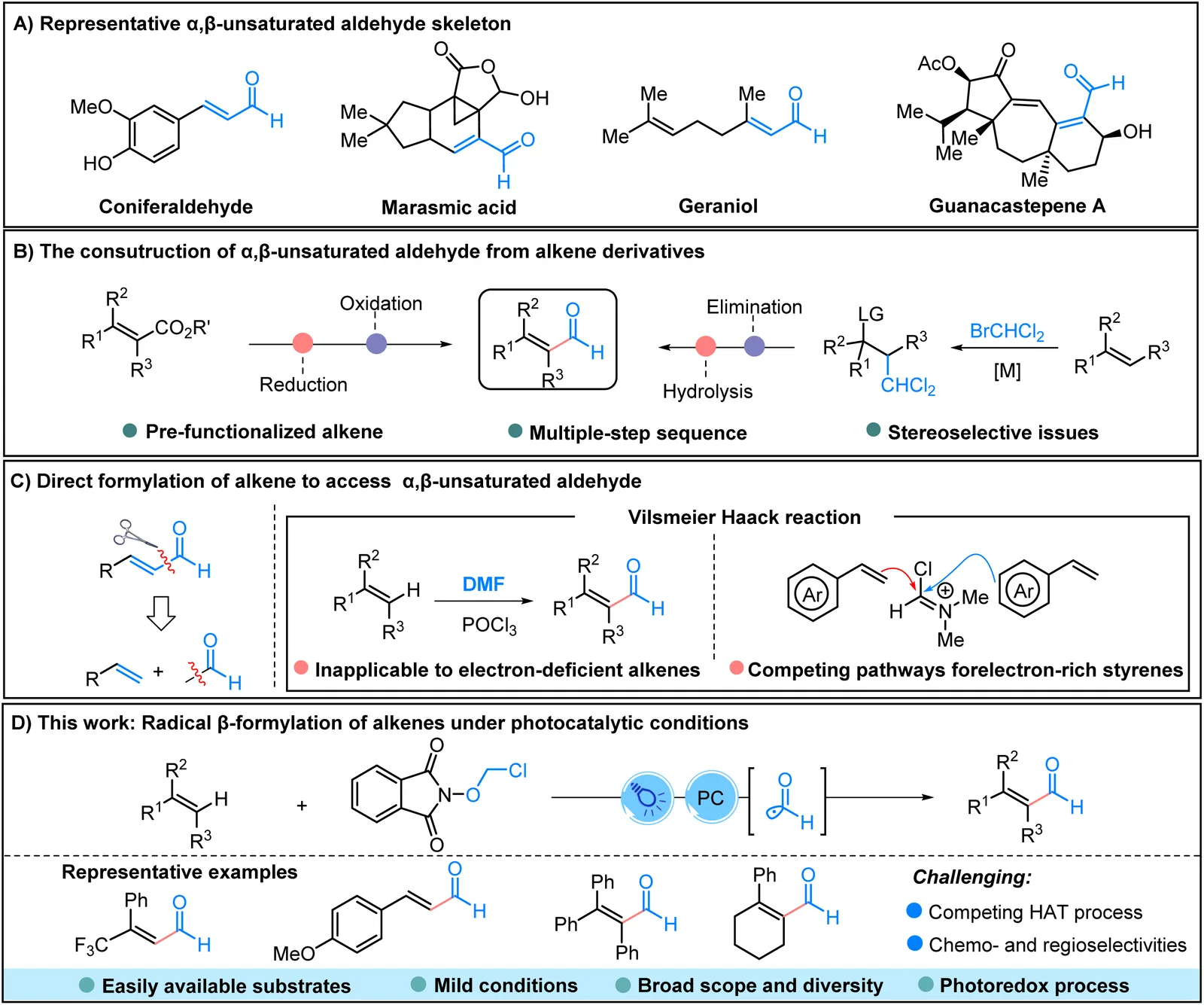
α,β-Unsaturated aldehydes and rationale for the construction of this skeleton.

Classical routes toward these compounds typically rely on alkyne hydroformylation with toxic syngas,^13,14^ or oxidative dehydrogenation of saturated aldehydes.^15,16^ Assembly of α,β-unsaturated aldehydes from simple alkenes constitutes an attractive alternative. Conventional indirect routes include reduction of Wittig/HWE-derived unsaturated esters to allylic alcohols, followed by oxidation to target aldehydes. Several radical alkene difunctionalization/elimination sequences have also been reported, yet these strategies require tedious multistep manipulations with low atom efficiency and frequently produce undesired C=C isomerization byproducts (Fig. 1B).^17,18^ Direct formylation of readily accessible alkenes would therefore be an ideal, albeit challenging, disconnection, as it circumvents prefunctionalization and delivers a more concise, sustainable synthetic manifold. The well-established Vilsmeier–Haack reaction enables alkene formylation, but demands stoichiometric toxic POCl_3_ and fails with electron-poor alkenes.^19,20^ More importantly, electron-rich styrenes afford poor regioselectivity under these conditions, owing to competitive electrophilic attack of the Vilsmeier reagent at both the electron-rich aromatic ring and the alkene C=C bond (Fig. 1C). Thus, the straightforward construction of α,β-unsaturated aldehydes *via* selective β-formylation of alkene remains an unmet synthetic challenge of high demand.

Radical hydroformylation has emerged as a mild alternative in recent years,^21–24^ but most established variants proceed *via* dominant hydrogen atom transfer (HAT) process to furnish saturated aldehydes, and catalytic radical strategies enabling one-step access to enals from alkenes remain rare.^25,26^ Inspired by the formylating capability of α-chloro-*N*-methoxyphthalimide under photoredox catalysis^27,28^ and our long-standing interest in alkene radical functionalization,^29–39^ we envisioned that suppressing the competitive HAT pathways after formyl radical addition would enable the formation of α,β-unsaturated aldehydes. Nevertheless, several fundamental challenges were anticipated: undesired benzylic HAT, problematic chemo- and regiocontrol for alkenes spanning broad redox potential windows, and over-reaction of freshly formed enals with coexisting nucleophiles.^40–44^

Guided by systematic conditions screening and mechanistic interrogation, we herein disclose a visible-light-driven regioselective formylation of aryl alkenes to construct α,β-unsaturated aldehydes using α-chloro-*N*-methoxyphthalimide as the formyl precursor. This protocol is compatible with diverse styrene derivatives and challenging α-CF_3_-substituted alkenes to deliver enals in moderate to high yields. Remarkably, our photocatalytic strategy suppresses the competing aryl formylation pathway inherent to conventional Vilsmeier–Haack transformations of electron-rich styrenes. Overall, this method constitutes a practical and complementary route for the direct construction of α,β-unsaturated aldehydes from commercially accessible alkene feedstocks.

## Results and discussion

We commenced our investigations by utilizing α-chloro-*N*-methoxyphthalimide (2aa) as the formylating reagent to explore its reactivity toward 4-(3,3,3-trifluoroprop-1-en-2-yl)-1,1′-biphenyl (1aa) under photocatalytic conditions. To our delight, the targeted α,β-unsaturated aldehyde 3aa was isolated in 58% yield using Ir(ppy)_3_ as the photocatalyst and acetonitrile as the solvent (Table 1, Entry 1). A subsequent photocatalyst screening demonstrated that no alternative photocatalysts outperformed Ir(ppy)_3_ (Table 1, Entries 2–4). Notably, water exhibited a detrimental effect on the reaction efficiency; the yield dropped to 26% upon the addition of water as a co-solvent. Gratifyingly, removing water with molecular sieves significantly elevated the yield to 73% while retaining outstanding stereoselectivity (Table 1, Entries 5–6). A series of formylation reagent analogues (2ab–2ad) were subsequently screened in this reaction. Unfortunately, only analogue 2ab delivered the desired product in a modest yield of 53%, whereas no trace of product was detected with the remaining analogues (Table 1, Entries 7 and 8). Elevated light intensity diminished the reaction efficiency and deteriorated stereoselectivity, which was presumably attributed to the photoinstability of precursor 2aa as well as light-dependent changes in catalytic-intermediate populations (see the SI for details) (Table 1, Entry 9). Further optimizations were performed, yet no noticeable improvement in the reaction outcome was observed (see the SI for details). Moreover, control experiments unambiguously verified that both the photocatalyst and light irradiation are essential for this reaction transformation (Table 1, Entry 10).

**Table 1:** Optimization of the reaction conditions^a^

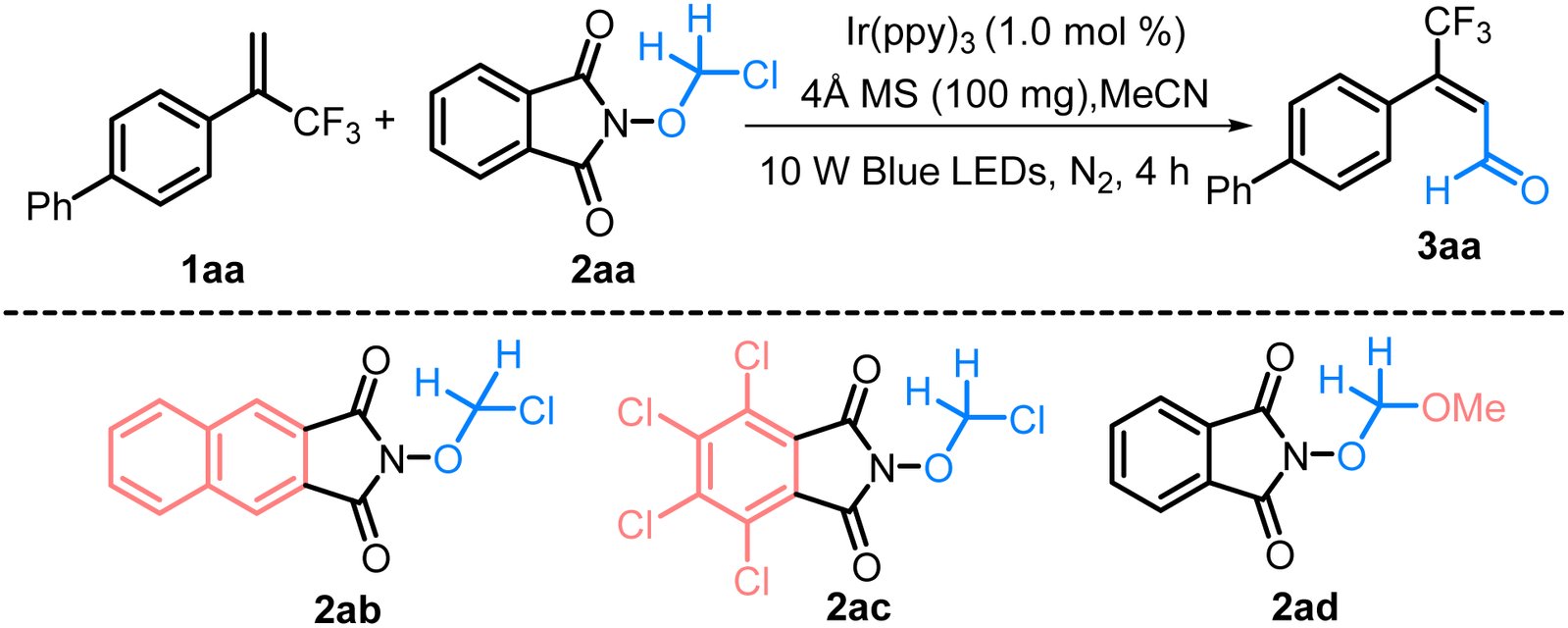			
Entry	Variation from standard conditions	3aa (%)^b^	*E*/*Z*^c^
1	Without MS	58	95 : 5
2^d^	Ir[(dF(CF_3_)ppy)]_2_(dtbbpy)PF_6_ as AC	36	92 : 8
3^d^	Eosin Y as PC	ND	—
4^d^	Ru(bpy)_3_Cl_2_ as PC	ND	—
5^d^	Water use as the co-solvent (v/v 20 : 1)	26	96 : 4
**6**	**None**	**73**	**96** **:** **4**
7	2ab as the substrate	53	95 : 5
8	2ac or 2ad as the substrate	ND	—
9	30 W blue LEDs	19	81 : 19
10	In dark or without PC	ND	—

a Reaction mixture of 1aa (0.20 mmol), 2aa (0.30 mmol), 4 Å MS (100 mg) and Ir(ppy)_3_ (1.0 mol%) in MeCN (0.05 M, dry) was irradiated with 10 W blue LEDs under N_2_ for 4 h.

b Isolated yields are given, n.r. = no reaction, n.d. = not detected.

c Determined by crude ^19^F NMR.

d In the absence of 4 Å MS in MeCN (0.1 M, dry).

To explore the generality of this photoredox-catalyzed formylation protocol, we systematically investigated the substrate scope with a diverse array of α-trifluoromethyl styrene derivatives (Scheme 1, 3aa–3az). Gratifyingly, the reaction proved highly general, delivering the corresponding trifluoromethylated α,β-unsaturated aldehydes in generally good yields and with high to excellent stereoselectivities favoring the *E*-alkene isomer. Simple aryl-substituted olefins, including biphenyl (3aa, 72%, *E*/*Z* = 95 : 5) and alkyl-substituted derivatives (3ab–3ad), reacted smoothly under the optimized conditions. Notably, both electronically neutral (unsubstituted phenyl, 3af) and electron-rich substrates, including those bearing alkoxy (3ag), phenoxy (3ah), trimethylsilyl (3ai), bulky *tert*-butyl (3aj), methyl (3ak) and morpholine (3ao) moieties, were well-tolerated, affording the corresponding products in moderate yields and high stereoselectivities. Notably, substrates bearing extended π-conjugated systems, such as *meta*-phenyl (3ae), 2-naphthyl (3al) and terphenyl (3as), also afforded the desired products in good yields (up to 73%) and high stereoselectivities (*E*/*Z* up to 95 : 5). Furthermore, a wide range of functional groups were found to be compatible, including halides (3am, 3at), esters (3an), and an internal alkyne moiety (3ap), highlighting the excellent chemoselectivity of the transformation. This broad functional group compatibility provides versatile handles for further synthetic elaboration of the resulting products. Heteroaromatic scaffolds, including benzothiophene (3au), dibenzofuran (3av) and dibenzothiophene (3aw, 3ax), were also successfully converted, albeit with slightly reduced yields in some cases (*e.g.*, 3au, 17%), likely due to competing side reactions involving the heteroaromatic core. The high stereoselectivity, with *E*/*Z* ratios often exceeding 90 : 10, is a particularly attractive feature, as it provides direct access to geometrically defined alkenes, which are valuable building blocks in organic synthesis. Intriguingly, studies on bis(α-trifluoromethylalkene) substrates revealed exclusive formation of the monoformylated product 3ay (55% yield). No diformylated byproduct was observed even with 4.0 equivalents of 2aa, presumably due to the increased electron deficiency of the alkene moiety following the initial formylation step. The utility of this method was further demonstrated by its successful application to more complex molecules, such as the menthol-derived substrate 3az, underscoring its potential for late-stage functionalization of advanced intermediates.

**Scheme 1:**
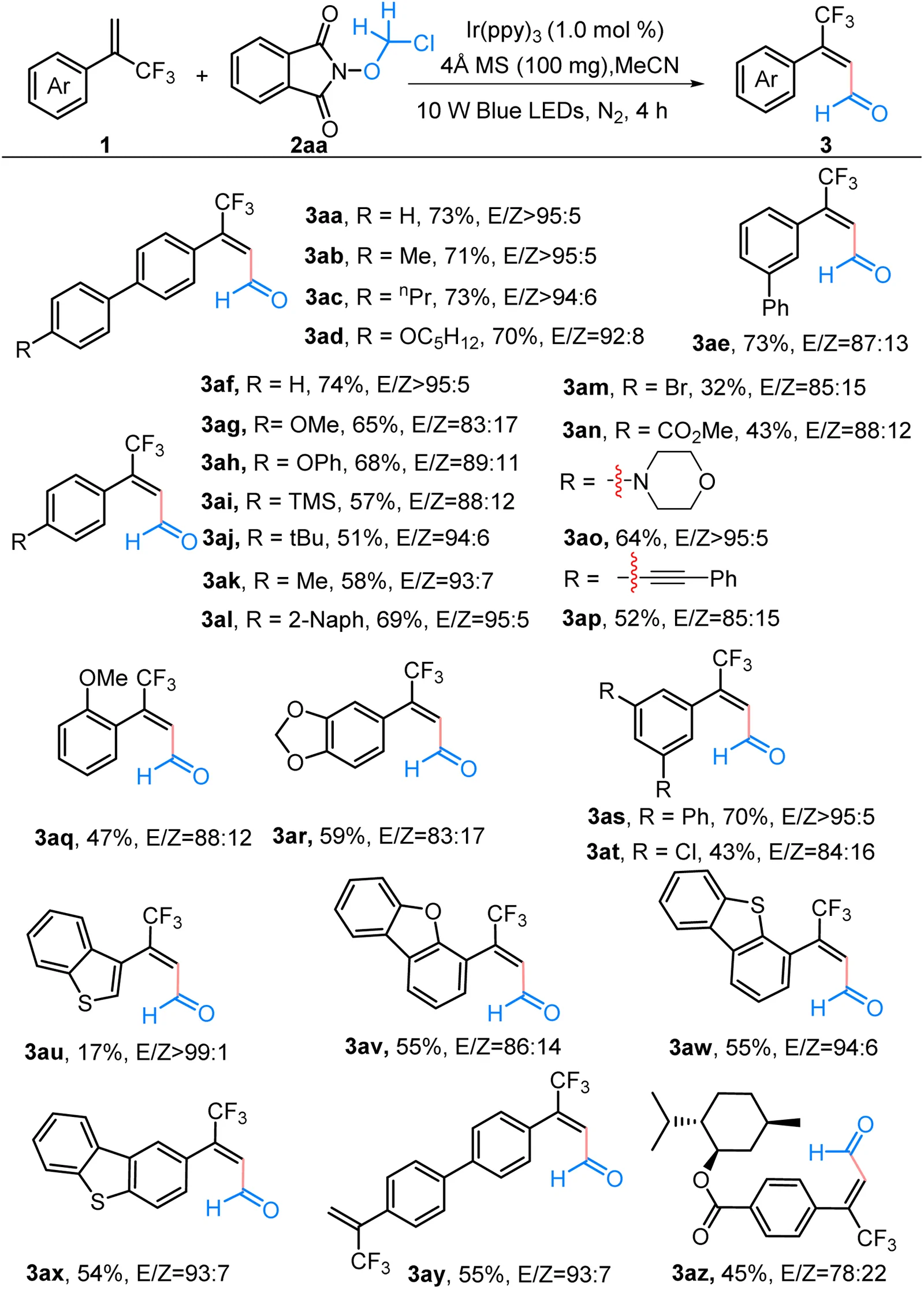
Substrate scope investigation. Reaction conditions: reaction mixture of 1 (0.20 mmol), 2aa (0.30 mmol), 4 Å MS (100 mg) and Ir(ppy)_3_ (1.0 mol%) in MeCN (0.05 M, dry) was irradiated with 10 W blue LEDs under N_2_ for 4 h, isolated yields are given and *E*/*Z* ratio was determined by crude ^19^F NMR.

We further investigated a series of styrene-derived alkenes to broaden the applicability of this formylation protocol. As shown in Scheme 2, these substrates underwent efficient formylation to deliver a range of cinnamaldehyde derivatives in moderate yields. The reaction exhibited moderate sensitivity to steric hindrance. For instance, shifting the methoxy substituent from the *para*- to the *ortho*-position led to a slight decrease in yield (3ba–3bc), likely due to increased steric encumbrance around the double bond. Unsubstituted styrene (3bg) afforded the target aldehyde in a synthetic useful 39% yield. Styrenes bearing electron-donating substituents, such as alkyl and alkoxy groups (3ba–3bf), generally provided higher yields than their electron-deficient counterparts (3bh–3bk). Notably, 1,1-diphenylethylene derivatives were also compatible with the reaction conditions, furnishing the corresponding formylated product (3bl) in moderate yield. Furthermore, internal alkenes (3bm), sterically congested tetra-substituted olefins (3bn), and cyclic olefins (3bo) were all viable substrates, further highlighting the broad scope and excellent functional group tolerance of this visible-light-mediated formylation strategy. Unactivated alkenes turned out to be incompatible, only trace amount of product was detected. This can be rationalized by the unfavorable addition of the formyl radical to unactivated alkenes: the resulting alkyl radical lacks adequate stabilization, leading to an extremely low reaction rate. Additionally, heteroaryl-substituted styrenes such as 2-vinylpyridine and 2-vinylthiophene were also incompatible, which is presumably attributed to the perturbation of the photocatalytic cycle by heteroatoms.

**Scheme 2:**
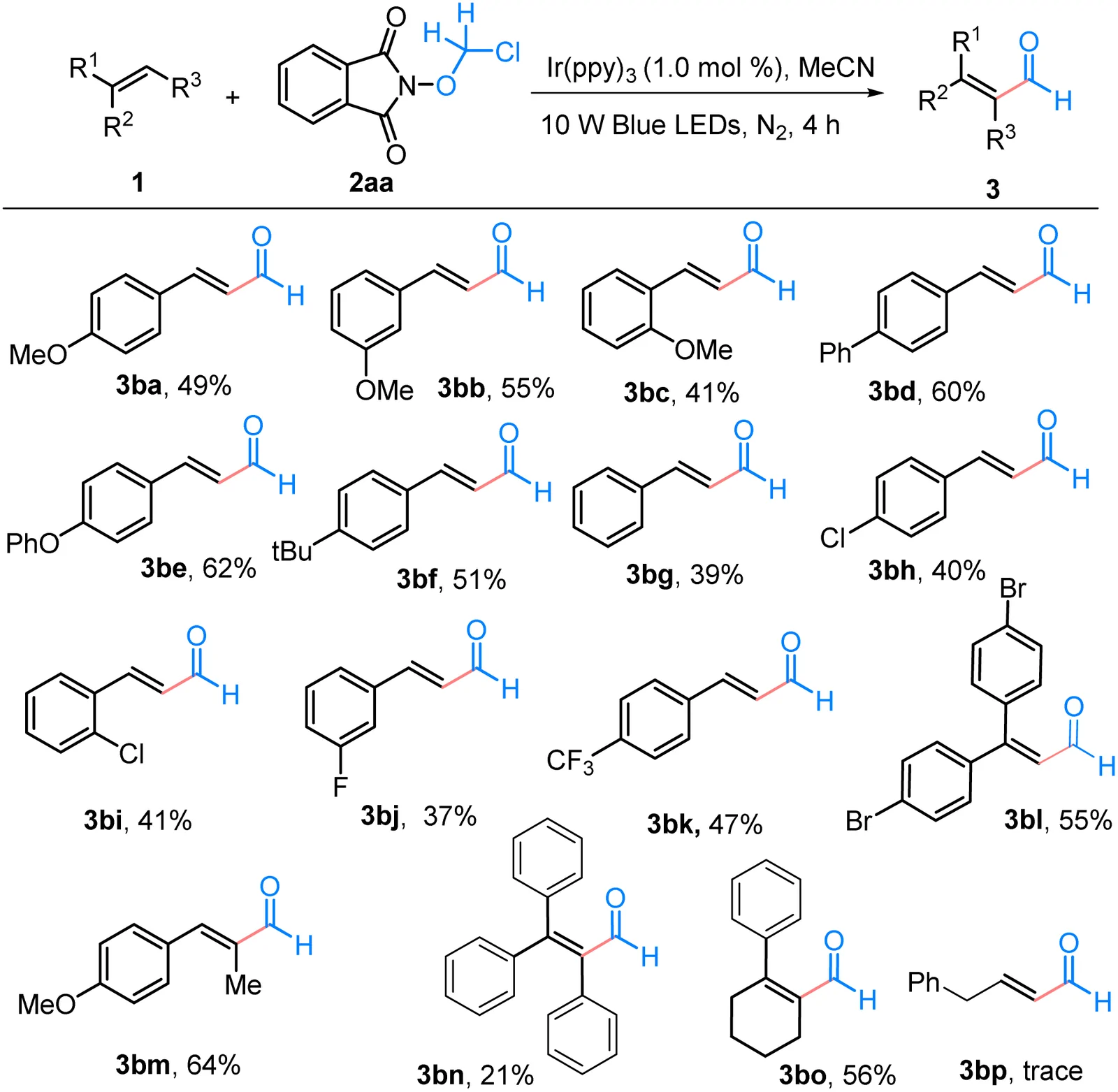
β-Formylation of styrene derivatives. Reaction mixture of 1 (0.2 mmol), 2aa (0.30 mmol) and Ir(ppy)_3_ (1.0 mol%) in MeCN (0.05 M, dry) was irradiated with 10 W blue LEDs under N_2_ for 4 h, isolated yields are given and all the *E*/*Z* > 95 : 5 based on crude ^1^H-NMR analysis.

To further demonstrate the synthetic practicability of this formylation strategy, we carried out a gram-scale transformation using unsubstituted styrene as the substrate. The target α,β-unsaturated aldehyde 3ah was successfully isolated in a moderate yield of 65% (Fig. 2A). To highlight the versatile synthetic utility of the enal product, we subsequently conducted a series of downstream derivatizations on the α,β-unsaturated aldehyde scaffold. Chemoselective reduction of the formyl group in 3ag was realized efficiently by treatment with sodium borohydride in methanol, delivering synthetically useful allylic alcohol 4 in 83% isolated yield. Moreover, reductive amination at the aldehyde site readily converted the conjugated enal into the corresponding alkenylamine 5. Such facile post-functionalization greatly expands the structural diversity of our product library (Fig. 2A).

**Fig. 2:**
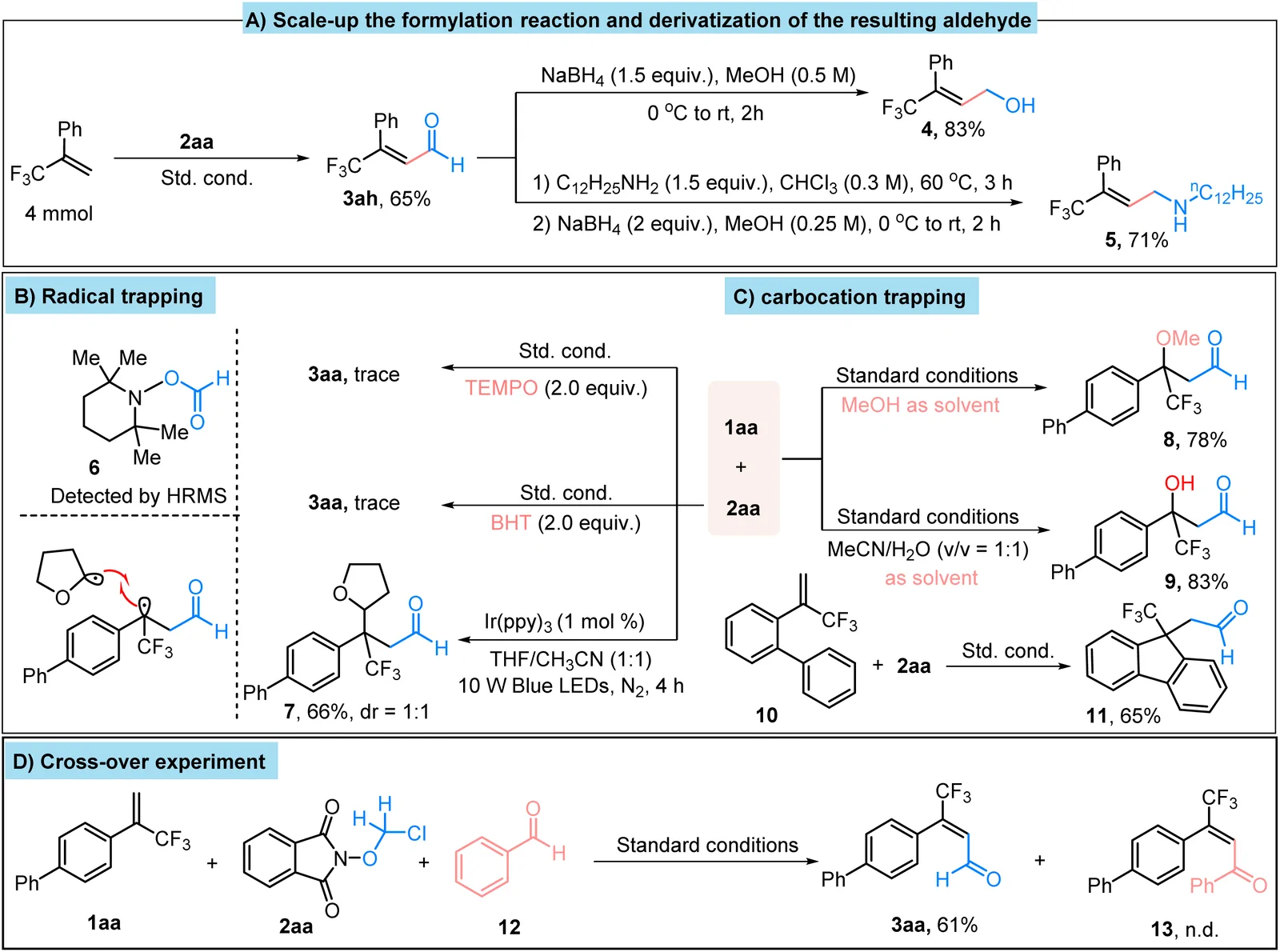
Scale-up reaction and mechanism investigation.

Subsequently, we conducted a series of control experiments to elucidate the mechanistic underpinnings of this transformation.^27,28^ Addition of the common radical scavengers TEMPO or BHT completely suppressed product formation, and the corresponding TEMPO adduct 6 was detected by HRMS (Fig. 2B). These observations strongly indicate that a radical-mediated pathway is dominant in this process. Furthermore, when the reaction was carried out in MeCN with THF as an additive, alkylated formylated product 7 was isolated in a 33% yield. This outcome is attributed to facile hydrogen atom abstraction from THF, followed by radical–radical coupling (ses SI for details); this observation corroborates the proposed radical cascade process. Notably, when the reaction was carried out in methanol or water, the corresponding methyl ether 8 and alcohol 9 were obtained in good yield, respectively (Fig. 2C). We propose that these adducts originate from nucleophilic addition of methanol or water to a carbocation intermediate. This mechanistic hypothesis is further supported by the following observation: *ortho*-phenyl-substituted trifluoromethyl styrene undergo intramolecular Friedel–Crafts cyclization onto the neighboring aromatic ring post-formylation, ultimately delivering cyclized product 11.^45–47^ Collectively, these results confirm the involvement of a radical-polar crossover pathway.

We next investigated the possible involvement of a formaldehyde intermediate that could generate the formyl radical *via* an intermolecular HAT pathway (Fig. 2D). In the crossover experiments using benzaldehyde 12 and substrate 2aa, only the expected formyl radical addition product 3aa was observed in 70% yield. In contrast, the benzoyl radical addition product 13 derived from benzaldehyde was not detected, thereby ruling out the intermolecular HAT process.

Furthermore, Stern–Volmer quenching experiments revealed that 2aa is the only effective quencher of the excited photocatalyst (Fig. 3A and B). Cyclic voltammetry measurements of 2aa showed a first reduction peak at −1.25 V (*vs.* SCE, Fig. 3C), indicating that single-electron transfer (SET) reduction of 2aa by excited Ir(ppy)_3_ is thermodynamically favorable.^48^ Light on–off experiments (Fig. 3D), together with the low quantum yield (*Φ* = 0.21), further confirmed that the formation of product 3aa is primarily non-chain in nature, supporting a photoredox catalytic cycle rather than a radical chain mechanism.

**Fig. 3:**
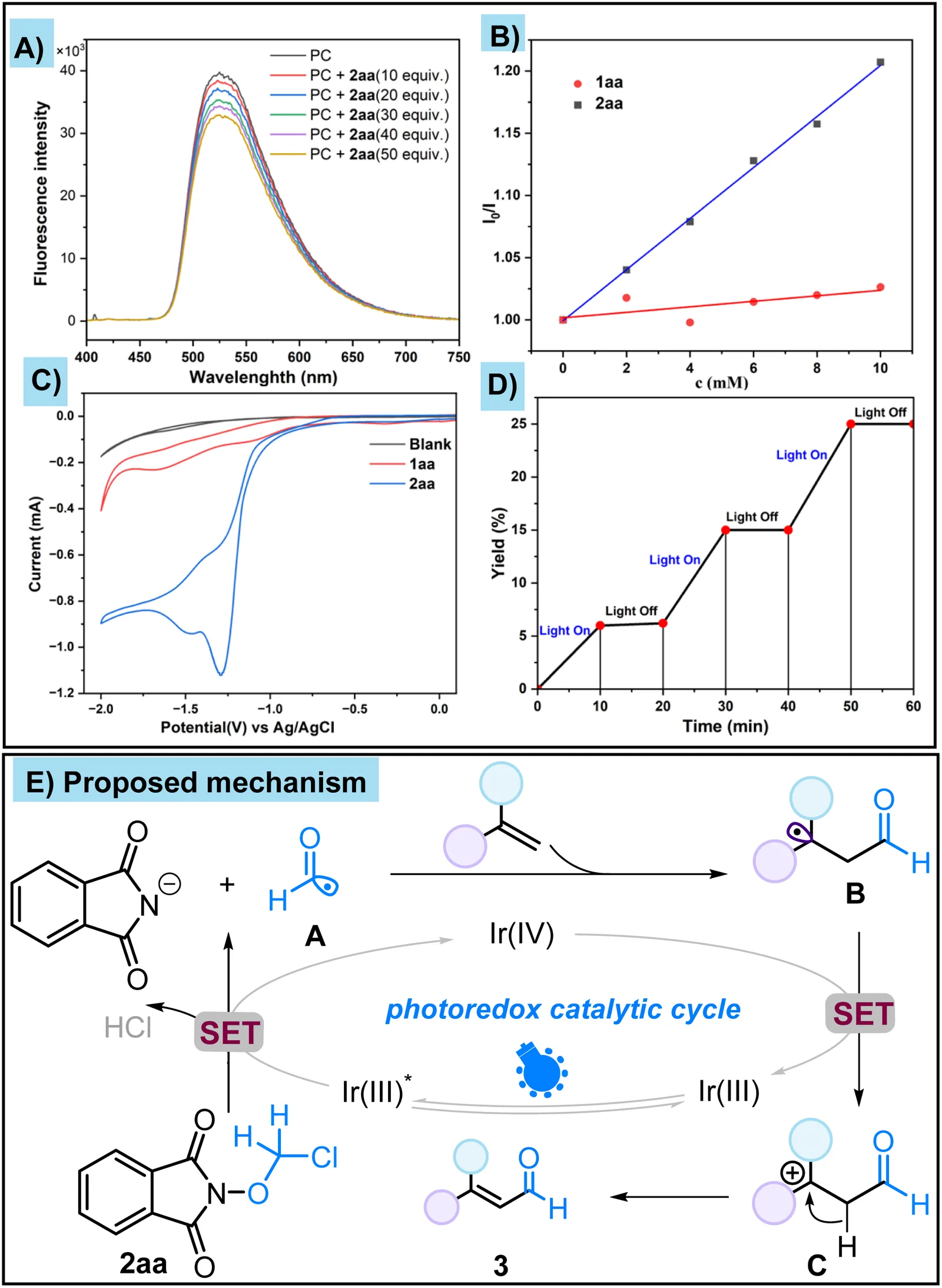
Mechanism investigation and proposed mechanism.

Based on the above experimental findings and literature precedents,^27,28,36,39^ we herein propose a plausible reaction mechanism for alkene formylation, as depicted in Fig. 3E. Initially, the ground-state photocatalyst is excited into its high-energy excited state, with a redox potential of [*E*_1/2_(Ir(iii)*/Ir(iv)) = −1.73 V *vs.* SCE]. Spontaneous single-electron reduction of substrate 2aa (*E*_1/2_ = −1.25 V *vs.* SCE) then takes place in a thermodynamically favourable manner. The resulting reduced intermediate rapidly eliminates HCl to release a phthalimide anion alongside formyl radical A. Subsequent radical addition of A across the alkene C=C bond furnishes benzylic radical B. The more oxidizing Ir(iv) species then oxidizes radical B to form benzylic carbocation C, regenerating the ground-state Ir(iii) catalyst and closing the photocatalytic cycle. Water may competitively intercept carbocation C to form by-product 9, and this side-reaction can be suppressed by 4 Å molecular sieves. Finally, deprotonation of carbocation C delivers the target α,β-unsaturated aldehyde product 3.

## Conclusions

In summary, we have developed a practical and efficient visible-light-mediated β-formylation reaction of styrenes and α-trifluoromethyl alkenes, employing α-chloro-*N*-methoxyphthalimide as a readily available formyl radical precursor. This mild protocol allows straightforward synthesis of structurally diverse α,β-unsaturated aldehydes, featuring broad functional group compatibility and excellent stereoselectivity. Notably, this transformation is well compatible with electron-deficient, sterically hindered, cyclic and heterocyclic alkenes, successfully overcoming the inherent drawbacks of traditional formylation strategies. Mechanistic investigations verify that this reaction proceeds *via* a radical-polar crossover catalytic cycle consisting of formyl radical addition and carbocation-mediated deprotonation. Therefore, this protocol provides a promising and complementary method for the targeted construction of unsaturated aldehyde skeletons from readily accessible alkene feedstocks.

## Author contributions

Q. J. R. W. and J. X. methodology, investigation, validation, writing; M. H. and X. D. supervision, project administration, review & editing, L. L. conceptualization, supervision, writing, review, funding acquisition.

## Conflicts of interest

There are no conflicts to declare.

## Supplementary Material

SC-017-D6SC05707A-s001

## Data Availability

The data supporting the results of this study are available in the supplementary information (SI) of this article. Supplementary information: detailed procedures, characterization data and NMR spectra. See DOI: https://doi.org/10.1039/d6sc05707a.
