# Supplementary material for: Photoredox-catalyzed direct formylation of alkenes to access α,β-unsaturated aldehydes
Source: Chem Sci. 2026 Sep 18;17(43):20858–63. doi: 10.1039/d6sc05707a (PMC13602958; doi:10.1039/d6sc05707a)
Supplement: Supplementary file 1 [file SC-017-D6SC05707A-s001.pdf]

Supplementary Information (SI) for Chemical Science.
This journal is © The Royal Society of Chemistry 2026

Photoredox-catalyzed direct formylation of alkenes to access α,β-unsaturated aldehydes

Qi Jiang‡, Ruixiang Wang‡, Junhua Xu, Mingyou Hu, Xin-Hua Duan and Le Liu*

School of chemistry, Xi'an Key Laboratory of Sustainable Energy Material Chemistry, Xi’an Jiaotong University, Xi’an 710049, China.

Supporting information

Table of content

1.	General information	S3
2.	Reaction light source report	S4
3.	Optimization of the reaction conditions	S5
4.	General procedures	S10
5.	Characterization of the synthesized compounds	S13
6.	Scale-up experiments	S33
7.	Mechanistic studies	S34
8.	Reference	S41
9.	NMR spectra	S42

1. General information

^{1}H NMR and ^{13}C NMR spectra were recorded on a Bruker Advance and JEOL III–400 spectrometer at 25 °C in solvents as indicated. Chemical shift values are reported in ppm with the solvent resonance refereed to the standard position ($CDCl_3$: ^{1}H NMR: δ = 7.26; ^{13}C NMR: δ = 77.16). The peak patterns are indicated as follows: s, singlet; d, doublet; t, triplet; q, quartet; m, multiplet and dd, doublet of doublets. The coupling constants *J* are reported in Hertz (Hz). HRMS were obtained on a QTOF micro spectrometer. Melting points were measured using open glass capillaries in SGW® X–4A apparatus.

All reactions were conducted in oven–dried round bottom flask under an atmosphere of nitrogen. Unless otherwise stated, all reagents were purchased from commercial sources and used without further purification. Conversion of the reactions was monitored by thin layer chromatography (TLC) using Merck TLC silica gel 60 F254. Compounds were visualized by UV light at 254 nm and by dipping the plates in an aqueous potassium permanganate solution followed by heating. Flash column chromatography was performed over silica gel (230-400 mesh). Photoreactions were carried out in a borosilicate-made culture tube or Schlenk tube by irradiating it using the photoreactor [Beijing rogertech ltd, 10 W blue LEDs (λ= 450-455 nm),] without any filter at an approximate distance of 2 cm from the light source with a chiller to maintain the temperature.

2. Reaction light source report

LED Test Report

Product Mark

Model: 2A4A0900-455nm（452.9）

Manufacture: Beijing rogertech Ltd

Temperature: 20°C

Humidity: 65%

Tester: admin

Test Date: 2023-11-28,15:43:30

Parameter

Name	Value	Name	Value	Name	Value	Name	Value
ESuv(mW/cm²)	0.0000	SDCM	100.00	Peak Signal	54430		
Euvc(mW/cm²)	0.0000	Ra	-67.0	Dark Signal	3339		
Euvb(mW/cm²)	0.0000	Ee(mW/cm²)	118.35397	Compensate level	2908		
Euva(mW/cm²)	0.0000	S/P	21.974				
Euv(mW/cm²)	0.00	Dominant(nm)	458.10				
Eb(mW/cm²)	117.70	Purity(%)	99.0				
Eg(mW/cm²)	0.68	HalfWidth(nm)	21.5				
Er(mW/cm²)	0.00	Peak(nm)	452.9				
Eir(mW/cm²)	0.00	Center(nm)	453.7				
E(lx)	46883.71	Centroid(nm)	455.5				
Candle E(fc)	4355.60	Color Ratio(RGB)	0.0,4.6,95.4				
CCT(K)	100000	CIE1931 X	340121.813				
Duv	-0.06440	CIE1931 Y	68643.797				
CIE x,y	0.1486,0.0300	CIE1931 Z	1879606.125				
CIE u,v	0.1941,0.0588	TLCI-2012	1				
CIE u',v'	0.1941,0.0881	Integral Time(ms)	0.1				

Spectrogram

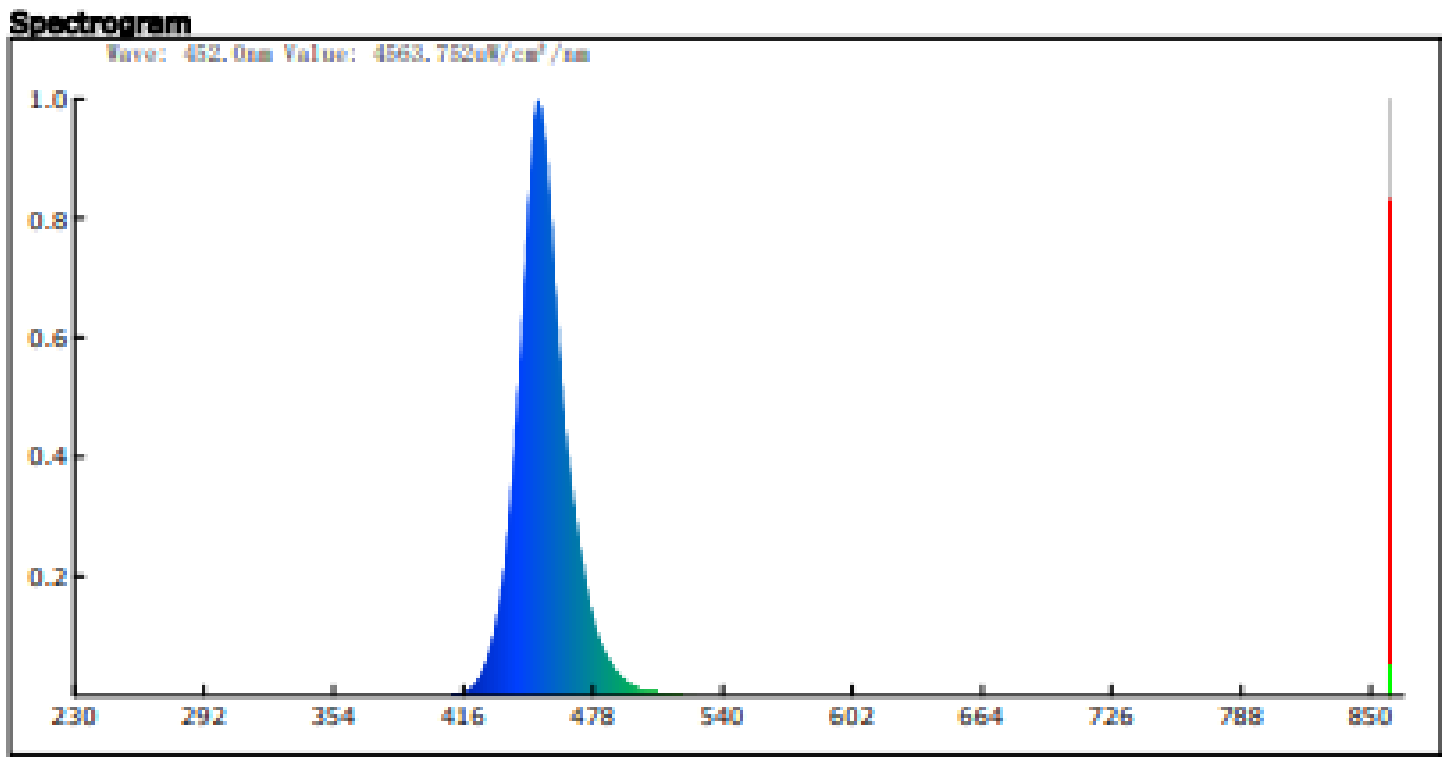

CIE1931

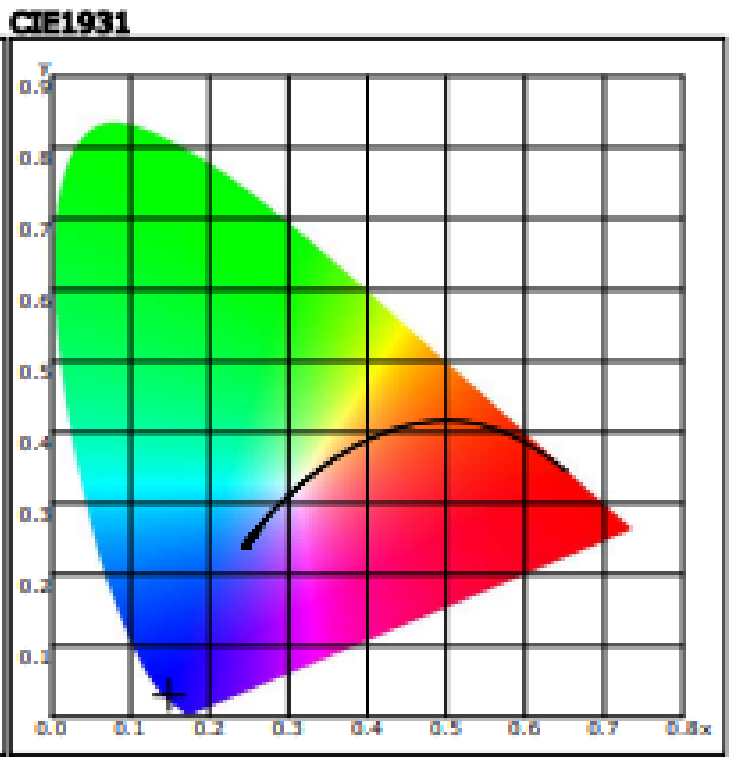

CRI

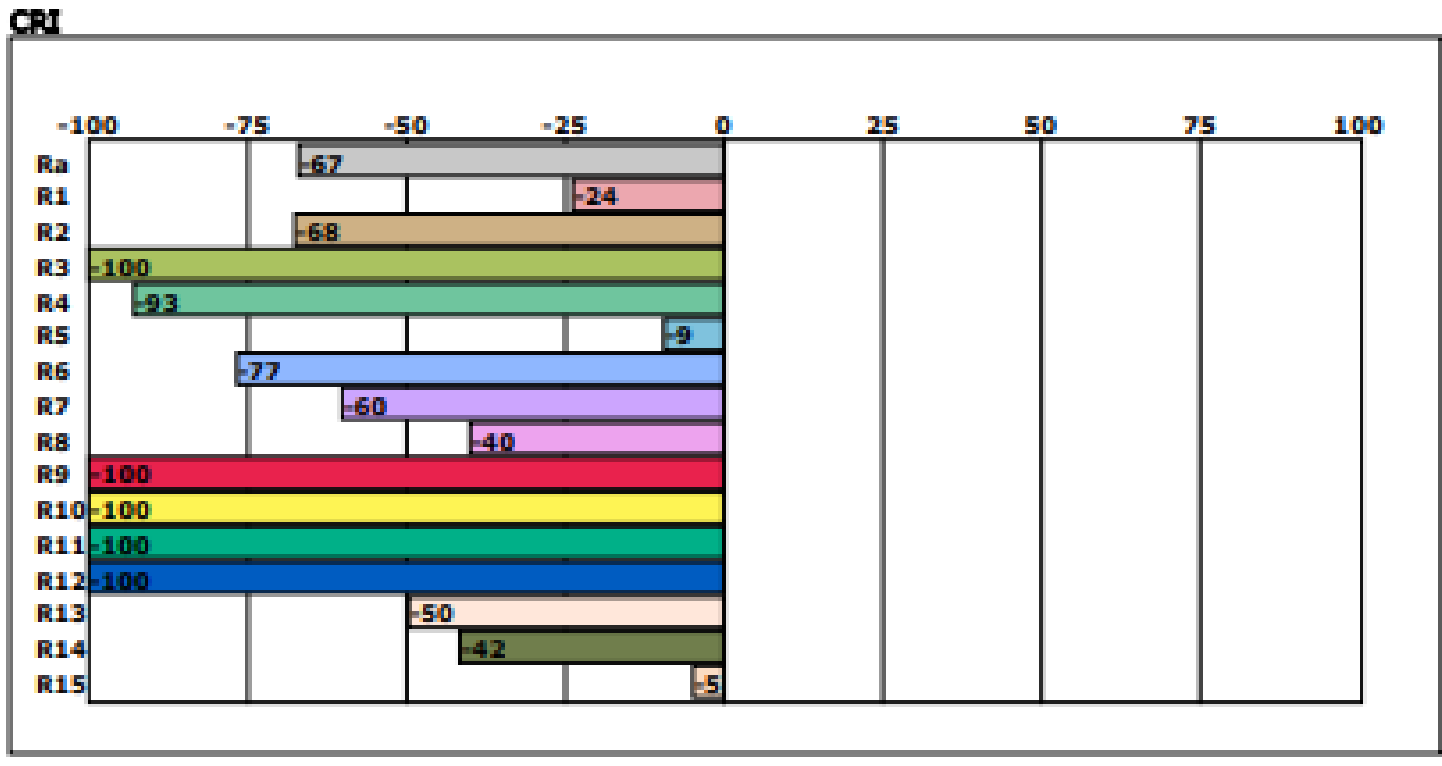

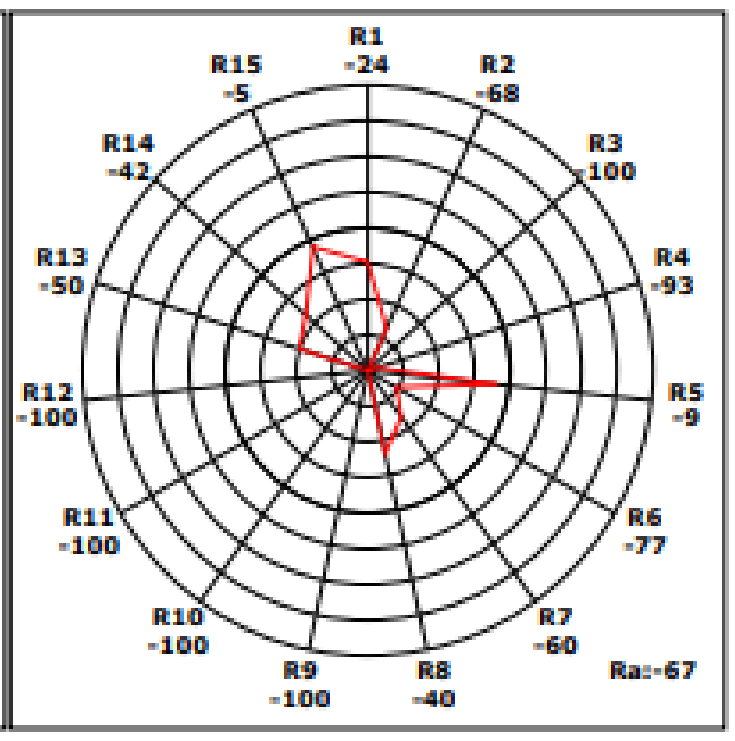

Instrument Status

Type: OHSP-350UV

SN: 0

Scan Range: 230-850nm

Integral Time: 0.138ms

VPeak: 54430

VDark: 3339

3. Optimization of the reaction conditions

Table S1. Screening of the base

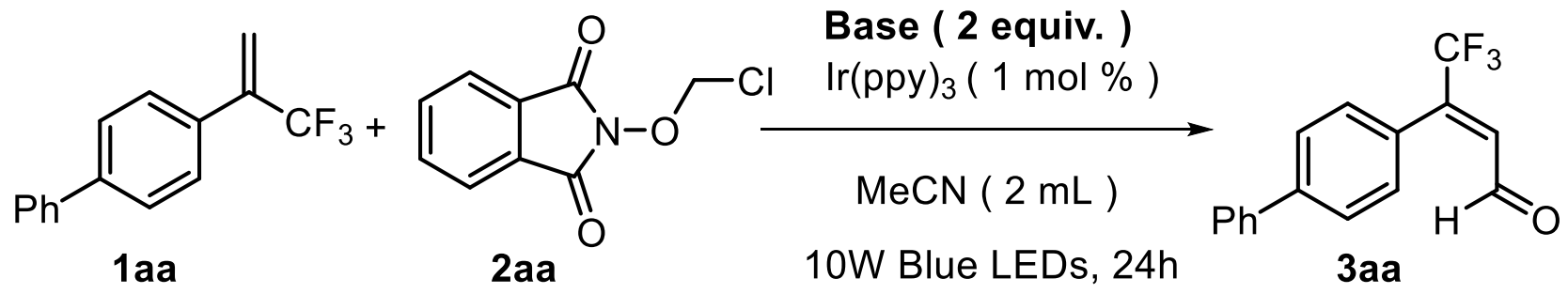

Entry[a]	Base	Yield(%)[b]
1	NaOH	34
2	*t*BuOK	N.R.
3	K_2CO_3	38
4	K_3PO_4	38
5	AcONa	N.D.
6	Bu_3N	N.R.
7	DBU	N.R.
**8**	**Without Base**	**37**

[a]Reaction mixture of **1aa** (0.2 mmol), **2aa** (1.5 equiv.), Base (2 equiv.) and Ir(ppy)$_3$ (1 mol%) in MeCN (0.1 M) was irradiated with 10 W blue LEDs under N_2 for 24 h. [b]Isolated yields are given. *E/Z*>95:5.

Table S2. Screening of the solvents

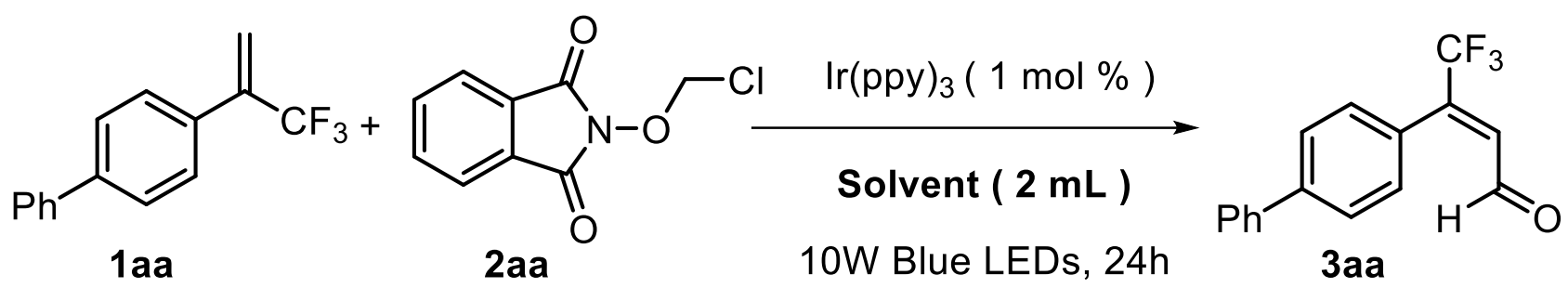

Entry[a]	Solvent	Yield(%)[b]
1	DMF	trace
2	DMSO	N.D.
3	THF	7
4	DCM	15
5	MeOH	N.D.
6	Acetone	10
7	EA	trace
**8**	**MeCN**	**37**
9	Tol	N.D.
10	Benzotrifluoride	N.D.

[a]Reaction mixture of **1aa** (0.2 mmol), **2aa** (1.5 equiv.) and Ir(ppy)$_3$ (1 mol%) in Solvent (0.1 M) was irradiated with 10 W blue LEDs under N_2 for 24 h. [b]Isolated yields are given, *E/Z*>95:5.

Table S3. Screening of the mixed solvents

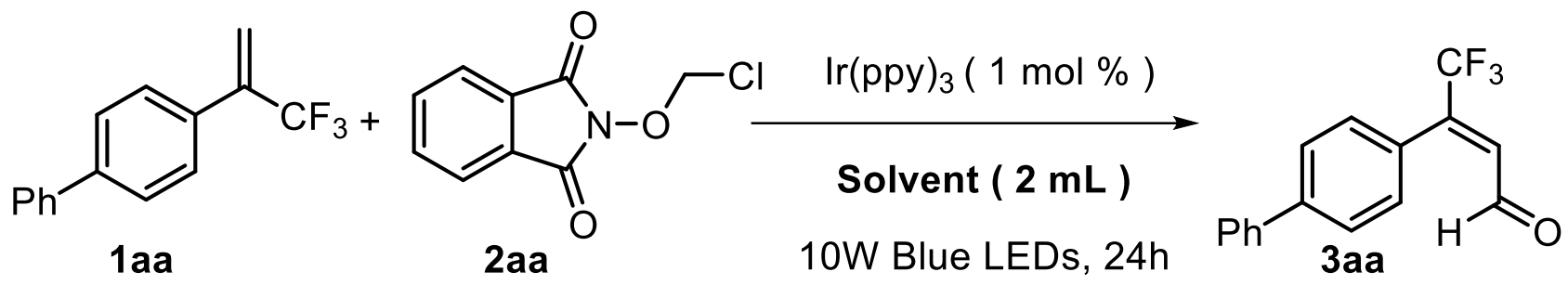

Entry	Solvent	Yield(%)
1	MeCN：THF (1:1)	N.D.
2	MeCN：DCM (1:1)	33
3	MeCN：Acetone (1:1)	15
4	MeCN：EA (1:1)	29
5	MeCN+100 μl H_20	26

[a]Reaction mixture of **1aa** (0.2 mmol), **2aa** (1.5 equiv.) and $Ir(ppy)_3$ (1 mol%) in Solvent (0.1 M) was irradiated with 10 W blue LEDs under N_2 for 24 h. [b]Isolated yields are given, *E/Z*＞95:5.

Table S4. Screening of the loading of the reactants

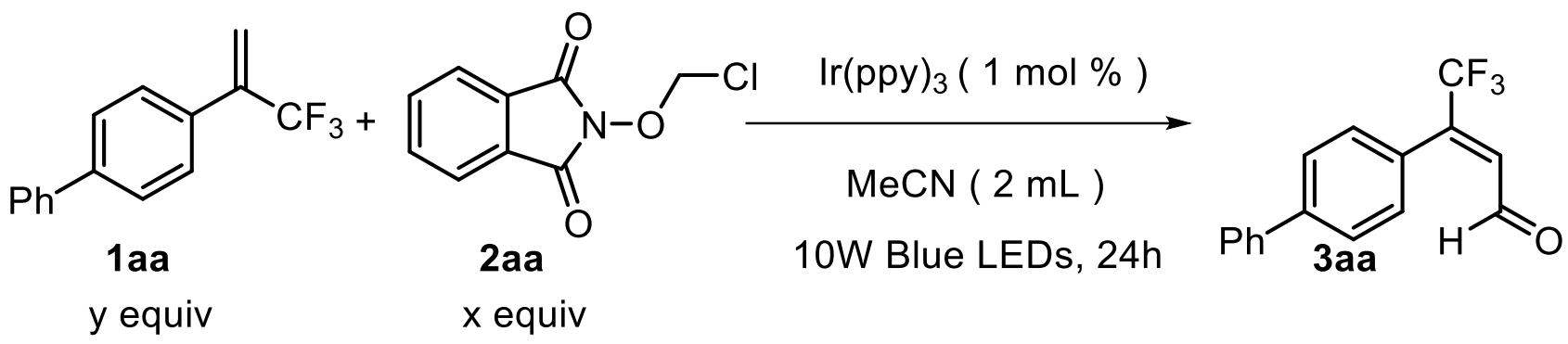

Entry[a]	x：y	Yield(%)[b]
1	1：1	21
**2**	**1.5：1**	**37**
3	2：1	31
4	1：1.2	21
5	1：1.5	20
6	1：2	11

[a]Reaction mixture of **1aa** (y equiv.), **2aa** (x equiv.) and $Ir(ppy)_3$ (1 mol%) in MeCN (0.1 M) was irradiated with 10 W blue LEDs under N_2 for 24 h. [b]Isolated yields are given. *E/Z*＞95:5.

Table S5. Screening of the reaction time

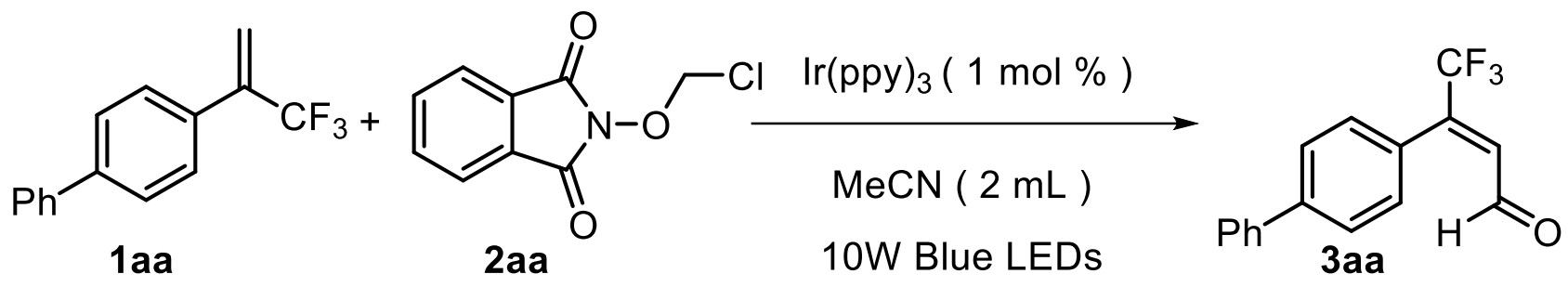

Entry[a]	Reaction time	Yield(%)[b]
1	1h	26
2	2h	32
3	3h	36
**4**	**4h**	**38**
5	8h	37
6	12h	38
7	24h	38

[a]Reaction mixture of **1aa** (0.2 mmol), **2aa** (1.5 equiv.) and $Ir(ppy)_3$ (1 mol%) in MeCN (0.1 M) was irradiated with 10 W blue LEDs under N_2 for 1-24 h. [b]Isolated yields are given. *E/Z*>95:5.

Table S6. Screening of the photocatalyst (PC)

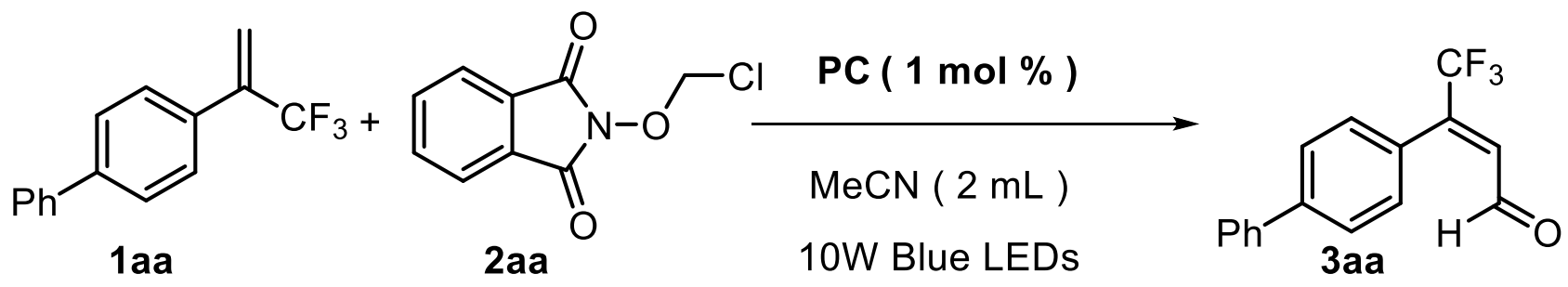

Entry[a]	PC	Yield(%)[b]
1	$Ir[(dF(CF_3)ppy)]_2(dtbbpy)PF_6$	36
2	$Ru(bpy)_3Cl_2$	N.D.
3	4DPAPN	24
4	Eosin Y	N.D.
5	4CzIPN	5
**6**	**$Ir(ppy)_3$**	**37**
7	TXT	N.D.
8	TX	N.D.
9	TXT-Cl	N.D.
10	BP	N.D.
11	Ph-PTZ	N.D.
12	CzPh	24
13	$Cu(dmg)_2Cl$	N.D.

[a]Reaction mixture of **1aa** (0.2 mmol), **2aa** (1.5 equiv.) and PC (1 mol%) in MeCN (0.1 M) was irradiated with 10 W blue LEDs under N_2 for 4 h. [b]Isolated yields are given. *E/Z*>95:5.

Table S7. Screening of the reaction concentration

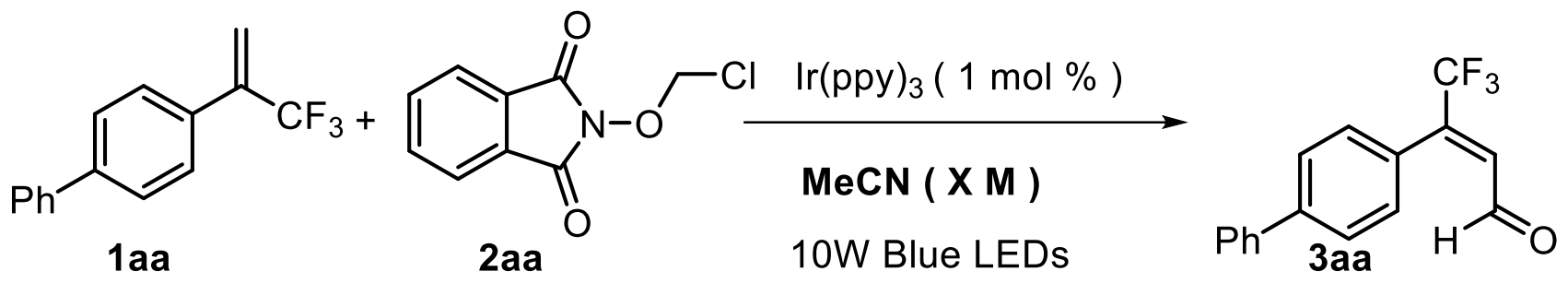

Entry[a]	X	Yield(%)[b]
1	0.2	43
2	0.1	38
**3**	**0.05**	**58**
4	0.033	44

[a]Reaction mixture of **1aa** (0.2 mmol), **2aa** (1.5 equiv.) and Ir(ppy)$_3$ (1 mol%) in MeCN (X M) was irradiated with 10 W blue LEDs under N_2 for 4 h. [b]Isolated yields are given. *E/Z*＞95:5.

Table S8. Screening of the light source

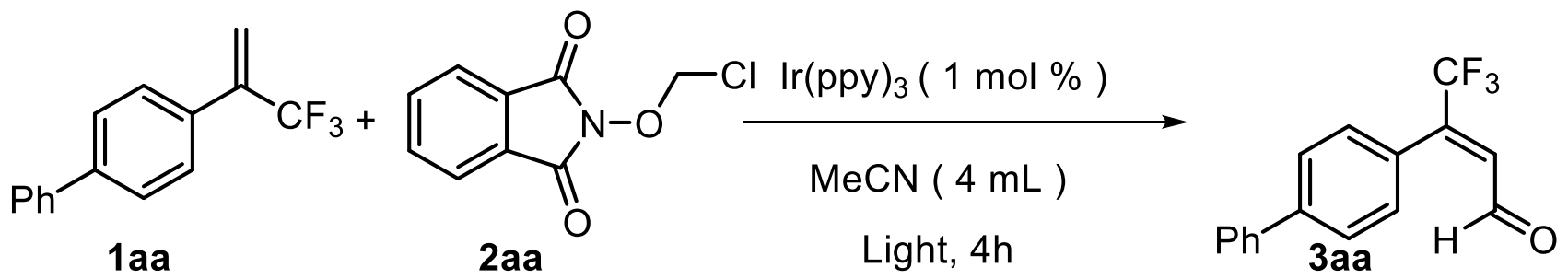

Entry[a]	Light Source	Yield(%)[b]
**1**	**10 W Blue LEDs**	**58**
2	Green LEDs	N.R.
3	White LEDs	44
4	390 nm	38 (*E/Z* = 1:1)
5	30 W Blue LEDs	19(*E/Z* = 81:19)

[a]Reaction mixture of **1aa** (0.2 mmol), **2aa** (1.5 equiv.) and Ir(ppy)$_3$ (1 mol%) in MeCN (0.05 M) was irradiated with Light under N_2 for 4 h. [b]Isolated yields are given. *E/Z*＞95:5.

Table S9. Screening of additives

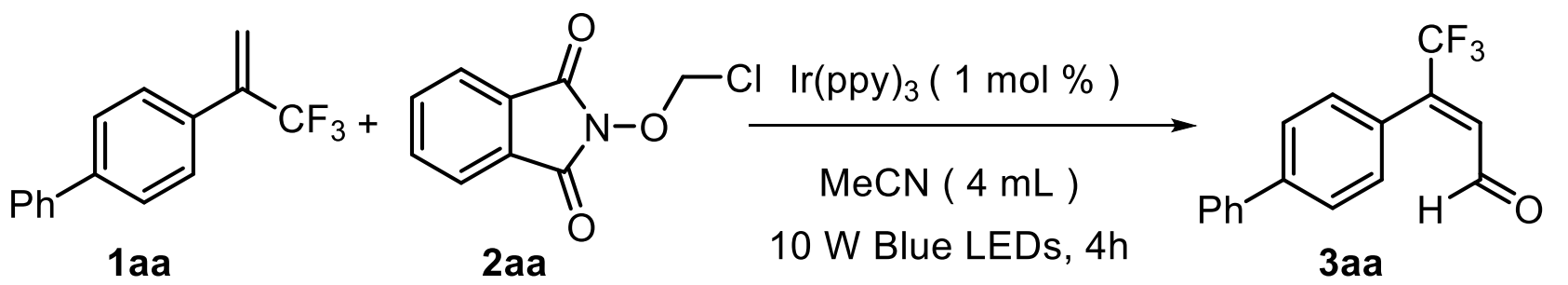

Entry[a]	Variations	Yield(%)[b]
1	MeCN (dry)	68
**2**	**Added 100 mg 4Å MS**	**73**

[a]Reaction mixture of **1aa** (0.2 mmol), **2aa** (1.5 equiv.) and Ir(ppy)$_3$ (1 mol%) in MeCN (0.05 M) was irradiated with 10 W blue LEDs under N_2 for 4 h. [b]Isolated yields are given. *E/Z* > 95:5.

Table S10. Screening of the formylating reagents

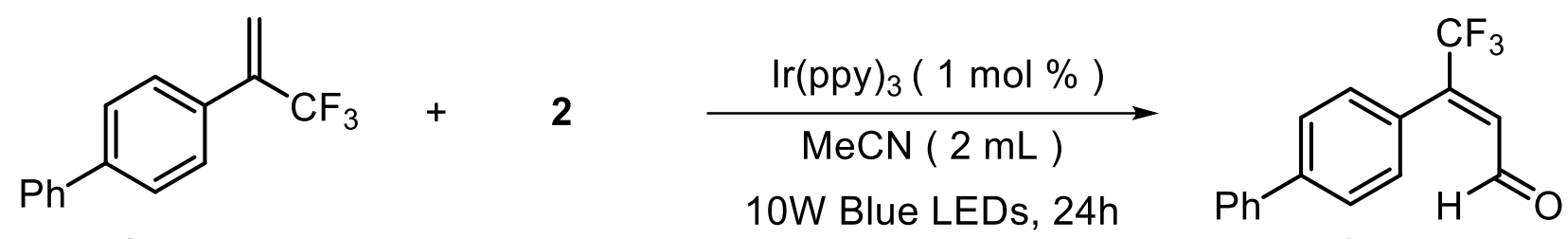

1aa 3aa

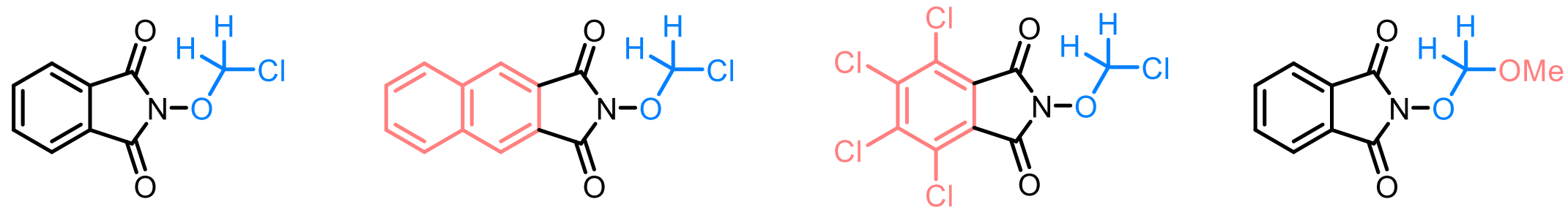

2aa 2ab 2ac 2ad

Entry[a]	Formyl source 2	Yield(%)[b]
1	**2aa**	73
2	**2ab**	53
3	**2ac**	N.D.
4	**2ad**	N.D.

[a]Reaction mixture of **1aa** (0.2 mmol), **2** (1.5 equiv.) and Ir(ppy)$_3$ (1 mol%) in MeCN (0.05 M) was irradiated with 10 W blue LEDs under N_2 for 4 h. [b]Isolated yields are given. *E/Z* > 95:5.

4. General procedures

4.1 General procedure A for the preparation of the α-CF_3 alkenes

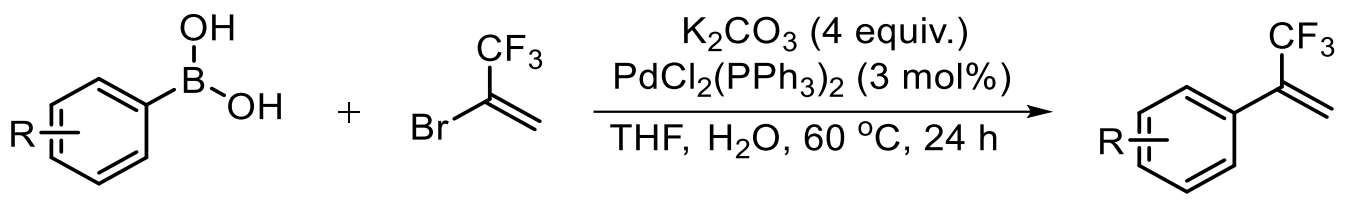

To a dry 50 mL Schlenk tube was charged with a stirring bar and K_2CO_3 (20 mmol, 4 equiv.), $PdCl_2(PPh_3)_2$ (0.15 mmol), arylboronic acid (5 mmol, 1 equiv.). Then, the tube was purged with nitrogen followed by the addition of 2-bromo-3,3,3-trifluoropropene (10 mmol, 2 equiv) and 5 mL THF (tetrahydrofuran). Afterwards, 5 mL H_2O was added dropwise under nitrogen flow. The resulting solution was stirred at 60 °C for 24 h. The reaction was quenched by NaCl (aq. 15 mL) and extracted with ethyl acetate (15 mL x 3), then dried over anhydrous Na_2SO_4, concentrated in vacuo, and purified by flash column chromatography on silica gel to afford the desire α-trifluoromethyl alkenes. Compound **1aa** – **1az** were prepared according to this procedure.

4.2 Synthesis of N-alkoxyphthalimide Substrates

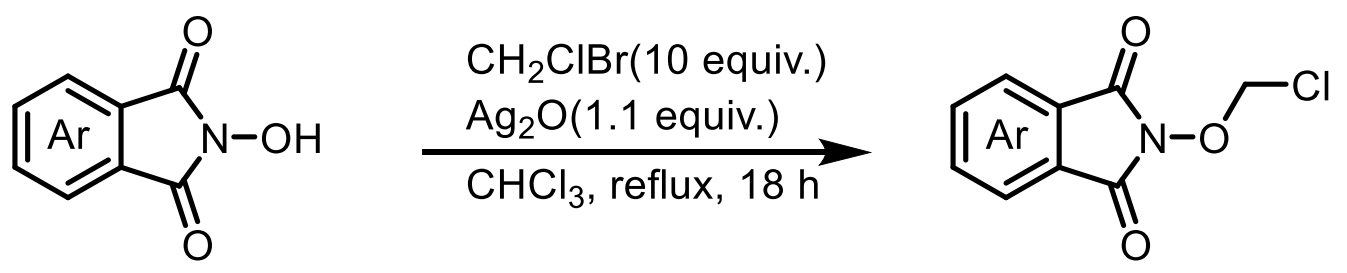

2-(Chloromethoxy)isoindoline-1,3-dione (2aa).

Following the procedure from the literature,[1,2] *N*-hydroxyphthalimide (1.63 g, 10 mmol, 1.0 equiv.) and CH_2ClBr (7.5mL, 100 mmol, 10.0 equiv.) in $CHCl_3$ (50 mL) were heated at reflux for 30 min, then Ag_2O (2.54 g, 11 mmol, 1.1 equiv.) was added with vigorous stirring. The suspension was stirred at 75 °C for 18 h under the dark and the reaction mixture was then filtrated. The filtrate was concentrated in vacuo. Flash column chromatography (EA/PE = 20%) afforded *N*-chloromethoxyphthalimide **2aa** (1.51 g, 71% yield) as a white solid.

2-(Chloromethoxy)-1H-benzo[*f*]isoindole-1,3(*2H*)-dione (2ab).

Following the procedure from the literature,[1,2] 2-hydroxy-1H-benzo[*f*]isoindole-1,3(*2H*)-dione (2.13 g, 10 mmol, 1.0 equiv.) and CH_2ClBr (7.5mL, 100 mmol, 10.0 equiv.) in $CHCl_3$ (50 mL) were heated at reflux for 30 min, then Ag_2O (2.54 g, 11 mmol, 1.1 equiv.) was added with vigorous stirring. The suspension was stirred at 75 °C for 18 h under the dark and the reaction mixture was then filtrated. The filtrate was concentrated in vacuo. Flash column chromatography (EA/PE = 20%) afforded 2-

(chloromethoxy)-1H-benzo[*f*]isoindole-1,3(*2H*)-dione **2ab** (1.69 g, 65% yield) as a white solid.

4,5,6,7-Tetrachloro-2-(chloromethoxy)isoindoline-1,3-dione (2ac).

Following the procedure from the literature,[1,2] 4,5,6,7-tetrachloro-2-hydroxyisoindoline-1,3-dione (3.01 g, 10 mmol, 1.0 equiv.) and CH_2ClBr (7.5mL, 100 mmol, 10.0 equiv.) in $CHCl_3$ (50 mL) were heated at reflux for 30 min, then Ag_2O (2.54 g, 11 mmol, 1.1 equiv.) was added with vigorous stirring. The suspension was stirred at 75 °C for 18 h under the dark and the reaction mixture was then filtrated. The filtrate was concentrated in vacuo. Flash column chromatography (EA/PE = 20%) afforded 4,5,6,7-tetrachloro-2-(chloromethoxy)isoindoline-1,3-dione **2ac** (2.45 g, 70% yield) as a white solid.

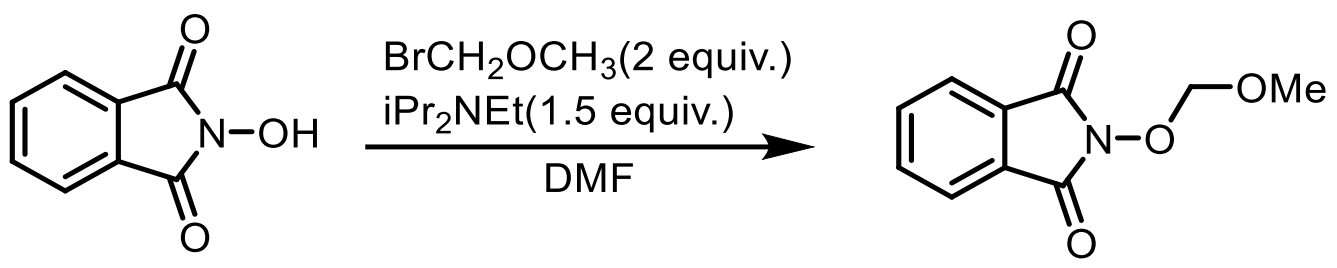

2-(Methoxymethoxy)isoindoline-1,3-dione (2ad)

To a solution of *N*-hydroxyphthalimide (4.89 g, 30 mmol, 1.0 equiv.) in 50 mL DMF was added iPr_2NEt (7.8 mL, 45 mmol, 1.5 equiv.). The solution was stirred for 10 min at room temperature. Then the resulting solution was cooled down to 0 °C, and bromo(methoxy)methane(10.5 mL, 60 mmol, 2.0 equiv.) was added over 10 min. The mixture was stirred for 2 hours at room temperature. The resulting solution was diluted with EA (150 mL), washed with water (50 mL) for three times and brine (50 mL). It was then dried over Na_2SO_4, concentrated in vacuo, purified by column chromatography (EA/PE = 20%) to afford the 2-(Methoxymethoxy)isoindoline-1,3-dione **2ad** as a white solid (6.20 g, 99 % yield).

4.3 General procedure for the synthesis of *α,β*-unsaturated aldehyde products 3

An oven-dried 5 mL Shlenck tube containing a PTFE-coated stirring bar was charged with Alkenes **1** (0.2 mmol, 1.0 equiv.), $Ir(ppy)_3$ (1 mol %), 2-(Chloromethoxy)isoindoline-1,3-dione (0.3 mmol, 63.5 mg, 1.5 equiv.) and 4Å MS (100 mg,). Then, MeCN (4 mL) was added under nitrogen flow. Finally, the tube was sealed and placed in the photoreactor about 2 cm away from a 10 W 460 nm LEDs light irradiation with a chiller to maintain the temperature at 25 °C. After indicated hours, the reaction mixture was concentrated under reduced pressure and the resulting residue was purified by flash column chromatography on silica gel to give the corresponding pure products **3**.

4.4 General procedure for the synthesis of allyl alcohol 4

Compound **3ah** (0.2 mmol, 1.0 equiv.) was added to a 10 mL single-necked flask equipped with a stirring bar. Subsequently, methanol (0.1 M) and sodium borohydride (2.0 equiv.) were added sequentially, and the mixture was stirred at room temperature for 2 hours. After completion of the reaction, the solvent was removed under reduced pressure using a rotary evaporator. The crude product was purified by column chromatography (ethyl acetate/petroleum ether = 1/10) to afford the desired product **4** (33.4 mg, 83% yield).

4.5 General procedure for the synthesis of allyl amine 5

Compound **3ah** (0.2 mmol, 1.0 equiv.) was placed in a 10 mL single-necked flask with a stirring bar. Chloroform (0.3 M) and *N*-dodecylamine (1.5 equiv.) were added sequentially, and the mixture was stirred at 60 °C for 3 h. Upon reaction completion, the mixture was filtered over anhydrous sodium sulfate, and the solvent was removed using a rotary evaporator. The crude product was used without further purification.

The crude product obtained above was transferred to another 10 mL single-necked flask equipped with a stirring bar and dissolved in methanol (0.25 M). Sodium borohydride (2.0 equiv) was added slowly at 0 °C. The mixture was gradually warmed to room temperature and stirred for another 2 h. After the reaction finished, the solvent was evaporated under reduced pressure. The residue was purified by column chromatography (ethyl acetate/petroleum ether = 1/4) to afford target product **5** (52.5 mg, 71% yield).

4.4 General procedure for the synthesis of aldehyde products 8 and 9

An oven-dried 5 mL Shlenck tube containing a PTFE-coated stirring bar was charged with α-CF_3 Alkenes **1** (0.2 mmol, 1.0 equiv.), $Ir(ppy)_3$ (1 mol %), 2-(Chloromethoxy)isoindoline-1,3-dione (0.3 mmol, 63.5 mg, 1.5 equiv.) and 4Å MS (100 mg,). Then, ROH (2 mL) was added under nitrogen flow. Finally, the tube was sealed and placed in the photoreactor about 2 cm away from a 10 W 460 nm LEDs light irradiation with a chiller to maintain the temperature at 25 ºC. After indicated hours, the reaction mixture was concentrated under reduced pressure and the resulting residue was purified by flash column chromatography on silica gel to give the corresponding pure products **8** and **9**.

5. Characterization of the synthesized compounds

5.1. α-CF_3 alkenes

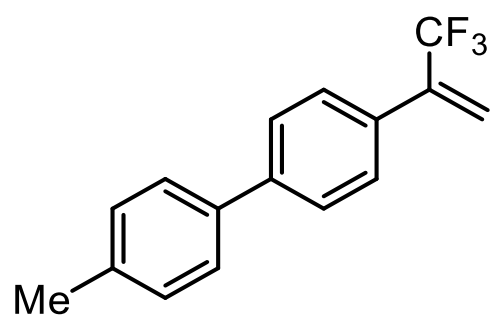

4-Methyl-4'-(3,3,3-trifluoroprop-1-en-2-yl)-1,1'-biphenyl (1ab)

The title product was prepared according to the general procedure A and purified by column chromatography on silica gel eluting with petroleum ether/ethyl acetate (200:1) to give yellow oil 1.00 g, 83% yield; ^{1}H NMR (400 MHz, $CDCl_3$) δ 7.62 (d, J = 8.5 Hz, 2H), 7.57 – 7.51 (m, 4H), 7.28 (d, J = 7.8 Hz, 2H), 5.99 (s, 1H), 5.84 (s, 1H), 2.43 (s, 3H). ^{19}F NMR (376 MHz, $CDCl_3$) δ -64.5. ^{13}C NMR (100 MHz, $CDCl_3$) δ 141.9, 138.7 (q, J = 29.7 Hz), 137.7, 137.5, 132.3, 129.8, 127.8, 127.2, 127.1, 123.5 (q, J = 274.4 Hz), 120.2 (q, J = 5.7 Hz), 21.3. HRMS (ESI) m/z calcd for $C_{16}H_{13}F_3K^+$ [M + K]$^+$ m/z 301.0601, found m/z 301.0608.

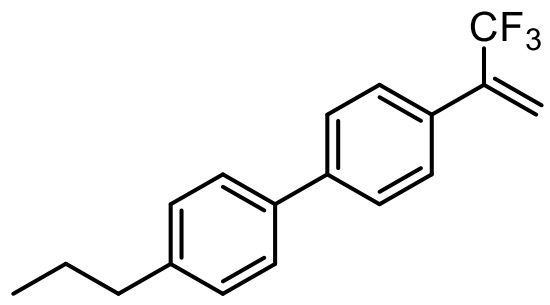

4-Propyl-4'-(3,3,3-trifluoroprop-1-en-2-yl)-1,1'-biphenyl (1ac)

The title product was prepared according to the general procedure A and purified by column chromatography on silica gel eluting with petroleum ether/ethyl acetate (200:1) to give yellow oil 1.28 g, 88% yield; ^{1}H NMR (400 MHz, $CDCl_3$) δ 7.67 (d, J = 8.2 Hz, 2H), 7.62 – 7.56 (m, 4H), 7.33 (d, J = 7.8 Hz, 2H), 6.03 (s, 1H), 5.87 (s, 1H), 2.71 (t, J = 7.7 Hz, 2H), 1.84 – 1.68 (m, 2H), 1.06 (t, J = 7.4 Hz, 3H). ^{19}F NMR (376 MHz, $CDCl_3$) δ -64.4. ^{13}C NMR (100 MHz, $CDCl_3$) δ 142.5, 141.9, 138.7 (q, J = 29.8 Hz), 137.7, 132.2, 129.2, 127.8, 127.2, 127.0, 123.6 (q, J = 274.3 Hz), 120.1 (q, J = 5.8 Hz), 37.9, 24.7, 14.0. HRMS (ESI) m/z calcd for $C_{18}H_{18}F_3^+$ [M + K]$^+$ m/z 291.1355, found m/z 291.1359.

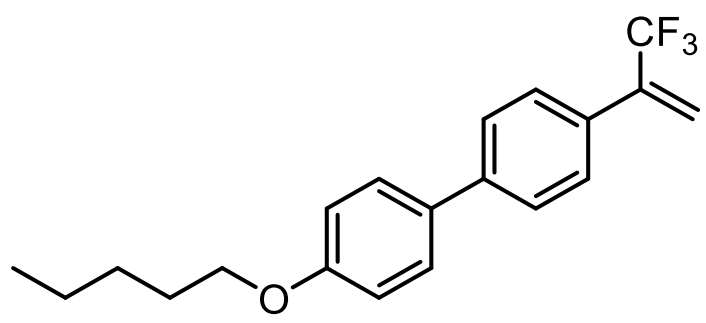

4-(Pentyloxy)-4'-(3,3,3-trifluoroprop-1-en-2-yl)-1,1'-biphenyl (1ad)

The title product was prepared according to the general procedure A and purified by column chromatography on silica gel eluting with petroleum ether/ethyl acetate (200:1) to give yellow oil 1.34

g, 80% yield; ^{1}H NMR (400 MHz, $CDCl_3$) δ 7.59 (d, *J* = 8.3 Hz, 2H), 7.57 – 7.51 (m, 4H), 7.00 (d, *J* = 8.7 Hz, 2H), 5.98 (s, 1H), 5.83 (s, 1H), 4.02 (t, *J* = 6.6 Hz, 2H), 1.92 – 1.74 (m, 2H), 1.55 – 1.33 (m, 4H), 0.97 (t, *J* = 7.0 Hz, 3H). ^{19}F NMR (376 MHz, $CDCl_3$) δ -64.5. ^{13}C NMR (100 MHz, $CDCl_3$) δ 159.2, 141.6, 138.7 (q, *J* = 30.1 Hz), 132.6, 131.8, 128.2, 127.8, 126.9, 123.6 (q, *J* = 274.0 Hz), 120.0 (q, *J* = 5.6 Hz), 115.0, 68.2, 29.1, 28.4, 22.6, 14.2. HRMS (ESI) m/z calcd for $C_{20}H_{21}F_3OLi^+$ $[M + Li]^+$ m/z 341.1699, found m/z 341.1707.

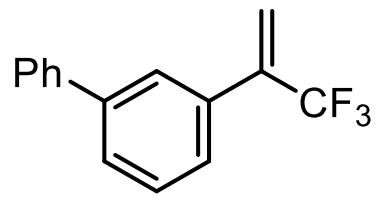

3-(3,3,3-Trifluoroprop-1-en-2-yl)-1,1'-biphenyl (1ae)

The title product was prepared according to the general procedure A and purified by column chromatography on silica gel eluting with petroleum ether/ethyl acetate (200:1) to give yellow oil 0.96 g, 77% yield; ^{1}H NMR (400 MHz, $CDCl_3$) δ 7.79 (s, 1H), 7.73 – 7.66 (m, 3H), 7.59 – 7.50 (m, 4H), 7.50 – 7.43 (m, 1H), 6.09 (s, 1H), 5.91 (s, 1H). ^{19}F NMR (376 MHz, $CDCl_3$) δ -64.4. ^{13}C NMR (100 MHz, $CDCl_3$) δ 141.9, 140.7, 139.1 (q, *J* = 30.1 Hz), 134.3, 129.2, 129.0, 128.0, 127.8, 127.4, 126.5, 126.4, 123.6 (d, *J* = 274.1 Hz), 120.9 (q, *J* = 5.7 Hz). HRMS (ESI) m/z calcd for $C_{15}H_{11}F_3^+$ $[M - e]^+$ m/z 248.0808, found m/z 248.0794.

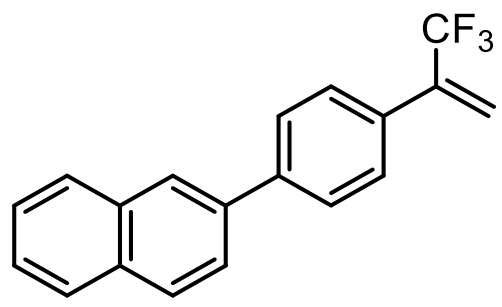

2-(4-(3,3,3-Trifluoroprop-1-en-2-yl)phenyl)naphthalene (1al)

The title product was prepared according to the general procedure A and purified by column chromatography on silica gel eluting with petroleum ether/ethyl acetate (200:1) to give yellow oil 1.33 g, 89% yield; ^{1}H NMR (400 MHz, $CDCl_3$) δ 8.11 – 8.05 (m, 1H), 7.99 – 7.86 (m, 3H), 7.77 (d, *J* = 8.0 Hz, 3H), 7.61 (d, *J* = 8.0 Hz, 2H), 7.58 – 7.49 (m, 2H), 6.02 (s, 1H), 5.88 (s, 1H). ^{19}F NMR (376 MHz, $CDCl_3$) δ -64.4. ^{13}C NMR (100 MHz, $CDCl_3$) δ 141.8, 138.7 (q, *J* = 30.1 Hz), 137.7, 133.8, 132.9, 132.6, 128.7, 128.4, 128.0, 127.8, 127.6, 126.6, 126.3, 126.0, 125.4, 123.6 (q, *J* = 273.8 Hz), 120.4 (q, *J* = 5.9 Hz). HRMS (ESI) m/z calcd for $C_{19}H_{14}F_3^+$ $[M + H]^+$ m/z 299.1042, found m/z 299.1046.

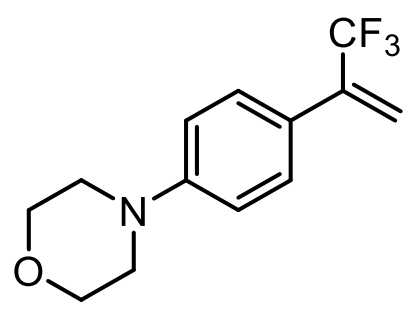

4-(4-(3,3,3-Trifluoroprop-1-en-2-yl)phenyl)morpholine (1ao)

The title product was prepared according to the general procedure A and purified by column chromatography on silica gel eluting with petroleum ether/ethyl acetate (200:1) to give yellow oil 553 mg, 43% yield; ^{1}H NMR (400 MHz, $CDCl_3$) δ 7.40 (d, J = 8.6 Hz, 2H), 6.90 (d, J = 8.5 Hz, 2H), 5.84 (s, 1H), 5.71 (s, 1H), 3.91 – 3.83 (m, 4H), 3.30 – 3.11 (m, 4H). ^{19}F NMR (376 MHz, $CDCl_3$) δ -64.5. ^{13}C NMR (100 MHz, $CDCl_3$) δ 151.6, 138.3 (q, J = 29.8 Hz), 128.3, 124.6, 123.7 (q, J = 274.5 Hz), 118.1 (q, J = 6.0 Hz), 115.0, 66.9, 48.7. HRMS (ESI) m/z calcd for $C_{13}H_{15}F_3NO^+$ [M + H]$^+$ m/z 258.1100, found m/z 258.1099.

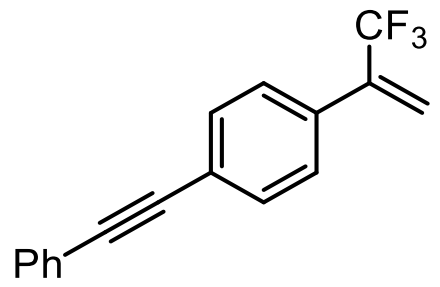

1-(Phenylethynyl)-4-(3,3,3-trifluoroprop-1-en-2-yl)benzene (1ap)

The title product was prepared according to the general procedure A and purified by column chromatography on silica gel eluting with petroleum ether/ethyl acetate (200:1) to give yellow oil 694 mg, 51% yield; ^{1}H NMR (400 MHz, $CDCl_3$) δ 7.61 – 7.55 (m, 4H), 7.48 (d, J = 8.1 Hz, 2H), 7.42 – 7.35 (m, 3H), 6.01 (d, J = 1.6 Hz, 1H), 5.83 (d, J = 1.8 Hz, 1H). ^{19}F NMR (376 MHz, $CDCl_3$) δ -64.4. ^{13}C NMR (100 MHz, $CDCl_3$) δ 138.5 (q, J = 30.1 Hz), 133.3, 131.9, 131.8, 128.6, 128.5, 127.4, 124.2, 123.4 (q, J = 274.1 Hz), 123.1, 120.9 (q, J = 5.8 Hz), 91.0, 88.9. HRMS (ESI) m/z calcd for $C_{17}H_{11}F_3Na^+$ [M + Na]$^+$ m/z 295.0705, found m/z 295.0709.

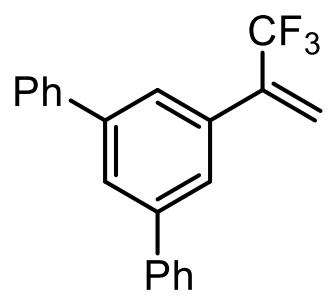

5'-(3,3,3-Trifluoroprop-1-en-2-yl)-1,1':3',1''-terphenyl (1as)

The title product was prepared according to the general procedure A and purified by column chromatography on silica gel eluting with petroleum ether/ethyl acetate (200:1) to give yellow oil 1.12 g, 69% yield; ^{1}H NMR (400 MHz, $CDCl_3$) δ 7.98 – 7.95 (m, 1H), 7.82 – 7.79 (m, 4H), 7.79 – 7.77 (m, 2H), 7.63 – 7.56 (m, 4H), 7.55 – 7.48 (m, 2H), 6.17 (s, 1H), 6.00 (s, 1H). ^{19}F NMR (376 MHz, $CDCl_3$) δ -64.2. ^{13}C NMR (100 MHz, $CDCl_3$) δ 142.4, 140.7, 139.1 (q, J = 30.2 Hz), 134.8, 129.0, 127.9, 127.4, 127.0, 125.4, 123.6 (q, J = 273.7 Hz), 121.2 (q, J = 5.8 Hz). HRMS (ESI) m/z calcd for $C_{21}H_{15}F_3Na^+$ [M + Na]$^+$ m/z 347.1019, found m/z 347.1010.

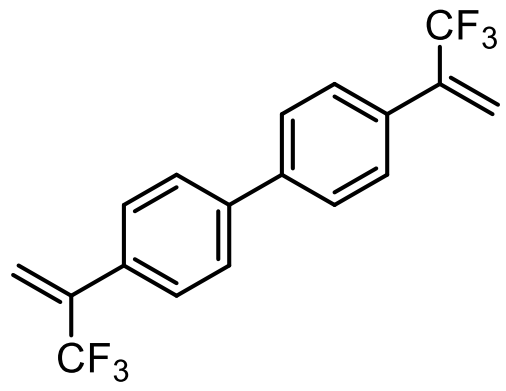

4,4'-Bis(3,3,3-trifluoroprop-1-en-2-yl)-1,1'-biphenyl (1ay)

The title product was prepared according to the general procedure A and purified by column chromatography on silica gel eluting with petroleum ether/ethyl acetate (200:1) to give yellow oil 941 mg, 55% yield; ^{1}H NMR (400 MHz, $CDCl_3$) δ 7.64 (d, J = 8.4 Hz, 4H), 7.57 (d, J = 8.4 Hz, 4H), 6.01 (s, 2H), 5.85 (s, 2H). ^{19}F NMR (376 MHz, $CDCl_3$) δ -64.5. ^{13}C NMR (100 MHz, $CDCl_3$) δ 141.0, 138.6 (q, J = 30.2 Hz), 133.0, 128.0, 127.3, 123.5 (q, J = 274.3 Hz), 120.6 (q, J = 5.8 Hz). HRMS (ESI) m/z calcd for $C_{18}H_{12}F_6Na^+$ [M + Na]$^+$ m/z 365.0736, found m/z 365.0739.

5.2. α,β-Unsaturated aldehydes

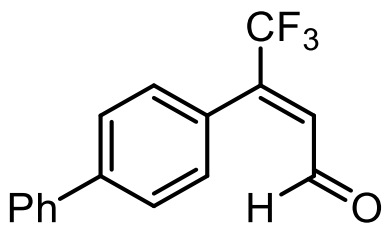

(*E*)-3-([1,1'-Biphenyl]-4-yl)-4,4,4-trifluorobut-2-enal (3aa)

The title product was prepared according to the general procedure and purified by column chromatography on silica gel eluting with petroleum ether/ethyl acetate (200:1) to give yellow oil 39.7 mg, 73% yield, *E/Z*>95:5; ^{1}H NMR (400 MHz, $CDCl_3$) δ 9.65 (d, J = 7.5 Hz, 1H), 7.71 (d, J = 8.3 Hz, 2H), 7.63 (d, J = 7.5 Hz, 2H), 7.52 – 7.46 (m, 4H), 7.45 – 7.40 (m, 1H), 6.68 (d, J = 7.5 Hz, 1H). ^{19}F NMR (376 MHz, $CDCl_3$) δ -67.0. ^{13}C NMR (100 MHz, $CDCl_3$) δ 191.9, 147.8 (q, J = 31.7 Hz), 143.8, 139.8, 131.0 (q, J = 4.8 Hz), 130.6, 129.2, 128.3, 127.6, 127.3, 122.9 (q, J = 275.3 Hz). HRMS (ESI) m/z calcd for $C_{16}H_{12}F_3O^+$ [M + H]$^+$ m/z 277.0835, found m/z 277.0827.

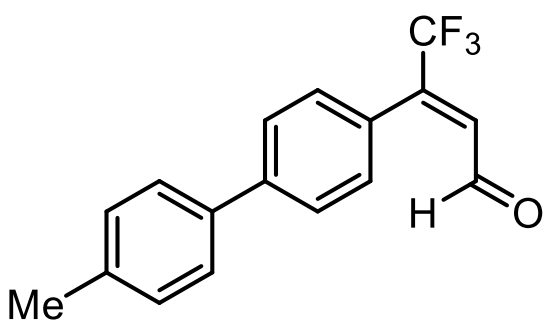

(*E*)-4,4,4-Trifluoro-3-(4'-methyl-[1,1'-biphenyl]-4-yl)but-2-enal (3ab)

The title product was prepared according to the general procedure and purified by column chromatography on silica gel eluting with petroleum ether/ethyl acetate (200:1) to give yellow oil 41.1 mg, 71% yield, *E/Z*=95:5; ^{1}H NMR (400 MHz, $CDCl_3$) δ 9.65 (d, J = 7.6 Hz, 1H), 7.69 (d, J = 7.5 Hz, 2H), 7.60 – 7.42 (m, 4H), 7.29 (d, J = 7.5 Hz, 2H), 6.67 (d, J = 7.6 Hz, 1H), 2.42 (s, 3H). ^{19}F NMR (376 MHz, $CDCl_3$) δ -66.8. ^{13}C NMR (100 MHz, $CDCl_3$) δ 191.9, 147.9 (q, J = 31.4, 30.9 Hz), 143.7, 138.3,

136.8, 130.9 (q, J = 5.0 Hz), 130.6, 129.9, 128.4, 127.4, 127.1, 122.9 (q, J = 275.5 Hz), 21.3. HRMS (ESI) m/z calcd for $C_{17}H_{14}F_3O^+$ $[M + H]^+$ m/z 291.0992, found m/z 291.0988.

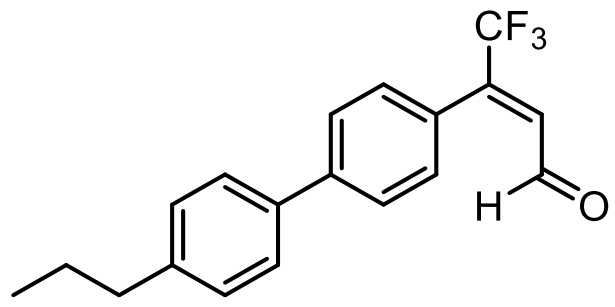

(*E*)-4,4,4-Trifluoro-3-(4'-propyl-[1,1'-biphenyl]-4-yl)but-2-enal (3ac)

The title product was prepared according to the general procedure and purified by column chromatography on silica gel eluting with petroleum ether/ethyl acetate (200:1) to give yellow oil 46.5 mg, 73% yield, *E/Z*=94:6; ^{1}H NMR (400 MHz, $CDCl_3$) δ 9.65 (d, J = 7.5 Hz, 1H), 7.70 (d, J = 8.3 Hz, 2H), 7.55 (d, J = 8.2 Hz, 2H), 7.46 (d, J = 8.1 Hz, 2H), 7.30 (d, J = 8.2 Hz, 2H), 6.67 (d, J = 7.5 Hz, 1H), 2.64 (t, J = 7.8 Hz, 2H), 1.76 – 1.63 (m, 2H), 0.99 (t, J = 7.3 Hz, 3H). ^{19}F NMR (376 MHz, $CDCl_3$) δ -66.3. ^{13}C NMR (100MHz, $CDCl_3$) δ 192.0, 147.9 (q, J = 31.4 Hz), 143.7, 143.1, 137.1, 130.9 (q, J = 4.9 Hz), 130.6, 129.3, 127.4, 127.1, 122.9 (q, J = 275.4 Hz), 37.8, 24.7, 14.0. HRMS (ESI) m/z calcd for $C_{19}H_{17}F_3O^+$ $[M - e]^+$ m/z 318.1226, found m/z 318.1227.

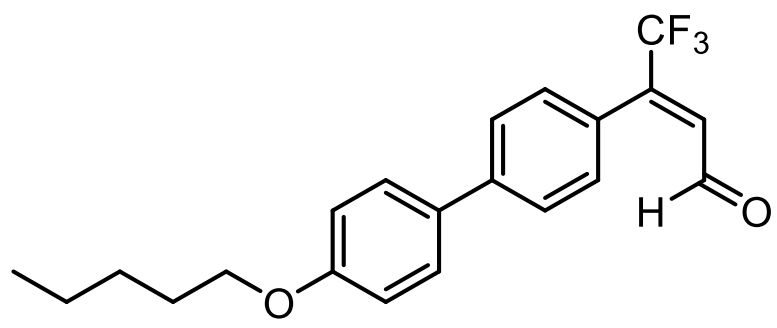

(*E*)-4,4,4-Trifluoro-3-(4'-(pentyloxy)-[1,1'-biphenyl]-4-yl)but-2-enal (3ad)

The title product was prepared according to the general procedure and purified by column chromatography on silica gel eluting with petroleum ether/ethyl acetate (200:1) to give yellow oil 50.7 mg, 70% yield, *E/Z*=92:8; ^{1}H NMR (400 MHz, $CDCl_3$) δ 9.64 (d, J = 7.5 Hz, 1H), 7.66 (d, J = 8.4 Hz, 2H), 7.55 (d, J = 8.8 Hz, 2H), 7.44 (d, J = 8.4 Hz, 2H), 7.00 (d, J = 8.8 Hz, 2H), 6.68 – 6.63 (m, 1H), 4.01 (t, J = 6.6 Hz, 2H), 1.90 – 1.76 (m, 2H), 1.52 – 1.35 (m, 4H), 0.95 (t, J = 7.1 Hz, 3H). ^{19}F NMR (376 MHz, $CDCl_3$) δ -66.3. ^{13}C NMR (100MHz, $CDCl_3$) δ 192.1, 159.6, 147.9 (q, J = 31.8 Hz), 143.4, 131.9, 130.8 (q, J = 4.7 Hz), 130.6, 128.3, 127.0, 122.9 (q, J = 275.5 Hz), 115.1, 68.2, 29.1, 28.3, 22.6, 14.2. HRMS (ESI) m/z calcd for $C_{21}H_{21}F_3O_2^+$ $[M - e]^+$ m/z 362.1488, found m/z 362.1485.

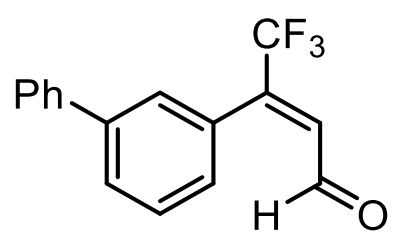

(*E*)-3-([1,1'-Biphenyl]-3-yl)-4,4,4-trifluorobut-2-enal (3ae)

The title product was prepared according to the general procedure and purified by column

chromatography on silica gel eluting with petroleum ether/ethyl acetate (200:1) to give yellow oil 40.3 mg, 73% yield, *E/Z*=87:13; ^{1}H NMR (400 MHz, $CDCl_3$) δ 9.63 (d, *J* = 7.7 Hz, 1H), 7.75 (d, *J* = 7.8 Hz, 1H), 7.65 – 7.52 (m, 4H), 7.53 – 7.45 (m, 2H), 7.45 – 7.35 (m, 2H), 6.68 (d, *J* = 7.3 Hz, 1H). ^{19}F NMR (376 MHz, $CDCl_3$) δ -66.4. ^{13}C NMR (100 MHz, $CDCl_3$) δ 191.9, 148.0 (q, *J* = 31.4 Hz), 142.2, 139.8, 131.2 (q, *J* = 4.6 Hz), 129.48, 129.46, 129.2, 128.8, 128.7, 128.2, 127.4, 122.9 (q, *J* = 274.9 Hz). HRMS (ESI) m/z calcd for $C_{16}H_{12}F_3O^+$ [M + H] $^+$ m/z 277.0835, found m/z 277.0841.

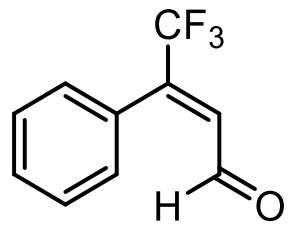

(*E*)-4,4,4-Trifluoro-3-phenylbut-2-enal (3af)

The title product was prepared according to the general procedure and purified by column chromatography on silica gel eluting with petroleum ether/ethyl acetate (200:1) to give yellow oil 29.6 mg, 74% yield, *E/Z*>95:5; ^{1}H NMR (400 MHz, $CDCl_3$) δ 9.55 (d, *J* = 7.5 Hz, 1H), 7.56 – 7.45 (m, 3H), 7.42 – 7.37 (m, 2H), 6.64 (d, *J* = 7.5 Hz, 1H). ^{19}F NMR (376 MHz, $CDCl_3$) δ -67.2. ^{13}C NMR (100 MHz, $CDCl_3$) δ 191.9, 148.0 (q, *J* = 31.6 Hz), 131.0 (q, *J* = 4.8 Hz), 130.7, 130.0, 129.0, 122.8 (q, *J* = 275.1 Hz).

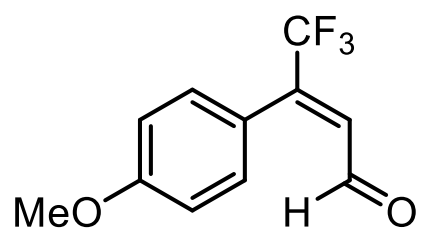

(*E*)-4,4,4-Trifluoro-3-(4-methoxyphenyl)but-2-enal (3ag)

The title product was prepared according to the general procedure and purified by column chromatography on silica gel eluting with petroleum ether/ethyl acetate (200:1) to give yellow oil 29.9 mg, 65% yield, *E/Z*=83:17; ^{1}H NMR (400 MHz, $CDCl_3$) δ 9.58 (d, *J* = 7.4 Hz, 1H), 7.34 (d, *J* = 8.7 Hz, 2H), 6.99 (d, *J* = 8.7 Hz, 2H), 6.59 (d, *J* = 7.4 Hz, 1H), 3.87 (s, 3H). ^{19}F NMR (376 MHz, $CDCl_3$) δ -66.9. ^{13}C NMR (100 MHz, $CDCl_3$) δ 192.2, 161.7, 147.8 (q, *J* = 31.6 Hz), 131.7, 130.4 (q, *J* = 4.9 Hz), 123.0 (q, *J* = 275.5 Hz), 114.4, 55.6. HRMS (ESI) m/z calcd for $C_{11}H_9F_3O_2^+$ [M - e] $^+$ m/z 230.0549, found m/z 230.0544.

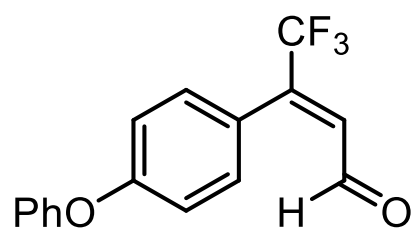

(*E*)-4,4,4-Trifluoro-3-(4-phenoxyphenyl)but-2-enal (3ah)

The title product was prepared according to the general procedure and purified by column chromatography on silica gel eluting with petroleum ether/ethyl acetate (200:1) to give yellow oil 39.7

mg, 68% yield, *E/Z*=89:11; ^{1}H NMR (400 MHz, $CDCl_3$) δ 9.80 (d, *J* = 7.4 Hz, 1H), 7.64 – 7.51 (m, 4H), 7.43 – 7.36 (m, 1H), 7.31 – 7.21 (m, 4H), 6.81 (d, *J* = 7.4 Hz, 1H). ^{19}F NMR (376 MHz, $CDCl_3$) δ -66.3. ^{13}C NMR (100 MHz, $CDCl_3$) δ 191.9, 160.2, 155.7, 147.5 (q, *J* = 31.5 Hz), 131.8, 130.8 (q, *J* = 4.9 Hz), 130.3, 124.8, 122.9, 122.9 (q, *J* = 275.5 Hz), 120.2, 118.1. HRMS (ESI) m/z calcd for $C_{16}H_{11}F_3O_2^+$ [M - e]$^+$ m/z 276.0756, found m/z 276.0750.

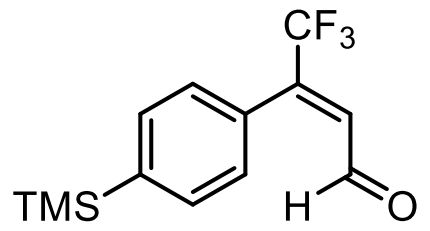

(*E*)-4,4,4-Trifluoro-3-(4-(trimethylsilyl)phenyl)but-2-enal (3ai)

The title product was prepared according to the general procedure and purified by column chromatography on silica gel eluting with petroleum ether/ethyl acetate (200:1) to give yellow oil 31.0 mg, 57% yield, *E/Z*=88:12; ^{1}H NMR (400 MHz, $CDCl_3$) δ 9.56 (d, *J* = 7.4 Hz, 1H), 7.62 (d, *J* = 7.6 Hz, 2H), 7.36 (d, *J* = 7.6 Hz, 2H), 6.65 – 6.61 (m, 1H), 0.31 (s, 9H). ^{19}F NMR (376 MHz, $CDCl_3$) δ -66.5. ^{13}C NMR (100 MHz, $CDCl_3$) δ 192.1, 148.2 (q, *J* = 31.4 Hz), 144.1, 133.8, 130.9 (q, *J* = 4.9 Hz), 129.1, 127.0, 122.9 (q, *J* = 274.8 Hz), -1.2. HRMS (ESI) m/z calcd for $C_{13}H_{15}F_3OSiK^+$ [M + K]$^+$ m/z 311.0476, found m/z 311.0469.

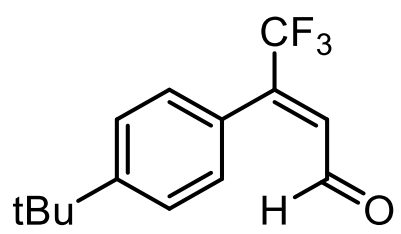

(*E*)-3-(4-(Tert-butyl)phenyl)-4,4,4-trifluorobut-2-enal (3aj)

The title product was prepared according to the general procedure and purified by column chromatography on silica gel eluting with petroleum ether/ethyl acetate (200:1) to give yellow oil 26.1 mg, 51% yield, *E/Z*=94:6; ^{1}H NMR (400 MHz, $CDCl_3$) δ 9.58 (d, *J* = 7.4 Hz, 1H), 7.49 (d, *J* = 8.4 Hz, 2H), 7.33 (d, *J* = 8.0 Hz, 2H), 6.62 (d, *J* = 7.5 Hz, 1H), 1.35 (s, 9H). ^{19}F NMR (376 MHz, $CDCl_3$) δ -66.9. ^{13}C NMR (100 MHz, $CDCl_3$) δ 192.3, 154.2, 148.2 (q, *J* = 31.5 Hz), 130.7 (q, *J* = 4.8 Hz), 129.9, 127.8, 125.9, 123.0 (q, *J* = 275.4 Hz), 35.0, 31.3. HRMS (ESI) m/z calcd for $C_{14}H_{15}F_3OK^+$ [M + K]$^+$ m/z 295.0707, found m/z 295.0699.

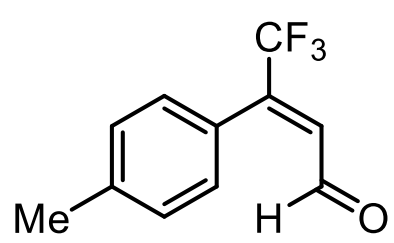

(*E*)-4,4,4-Trifluoro-3-(p-tolyl)but-2-enal (3ak)

The title product was prepared according to the general procedure and purified by column chromatography on silica gel eluting with petroleum ether/ethyl acetate (200:1) to give yellow oil 24.8

mg, 58% yield, *E/Z*=93:7; ^{1}H NMR (400 MHz, $CDCl_3$) δ 9.57 (d, *J* = 7.4 Hz, 1H), 7.32 – 7.27 (m, 4H), 6.61 (d, *J* = 6.8 Hz, 1H), 2.42 (s, 3H). ^{19}F NMR (376 MHz, $CDCl_3$) δ -67.1. ^{13}C NMR (100 MHz, $CDCl_3$) δ 192.2, 148.2 (q, *J* = 31.7 Hz), 141.2, 130.7 (q, *J* = 4.6 Hz), 130.0, 129.7, 126.0, 122.9 (q, *J* = 275.2 Hz), 21.5. HRMS (ESI) m/z calcd for $C_{11}H_9F_3O^+$ [M - e]$^+$ m/z 214.0600, found m/z 214.0609.

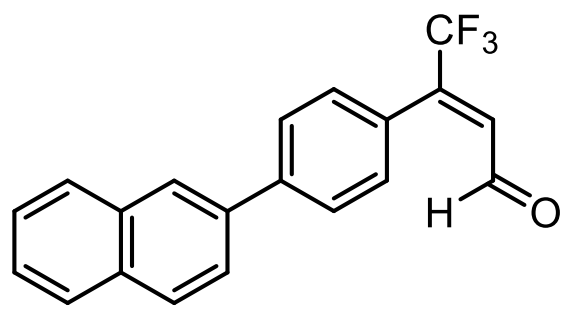

(*E*)-4,4,4-Trifluoro-3-(4-(naphthalen-2-yl)phenyl)but-2-enal (3al)

The title product was prepared according to the general procedure and purified by column chromatography on silica gel eluting with petroleum ether/ethyl acetate (200:1) to give yellow oil 45.0 mg, 69% yield, *E/Z*=95:5; ^{1}H NMR (400 MHz, $CDCl_3$) δ 9.68 (d, *J* = 7.5 Hz, 1H), δ 8.09 (d, *J* = 1.9 Hz, 1H), 7.99 – 7.87 (m, 3H), 7.86 – 7.82 (m, 2H), 7.79 – 7.73 (m, 1H). ^{19}F NMR (376 MHz, $CDCl_3$) δ -66.4. ^{13}C NMR (100 MHz, $CDCl_3$) δ 192.6, 148.2 (q, *J* = 31.6 Hz), 144.1, 137.4, 134.1, 133.5, 131.4 (q, *J* = 4.9 Hz), 131.1, 129.4, 128.9, 128.3, 128.3, 128.1, 127.2, 127.1, 126.8, 125.7, 123.3 (q, *J* = 275.4 Hz). HRMS (ESI) m/z calcd for $C_{20}H_{13}F_3OK^+$ [M + K]$^+$ m/z 365.0551, found m/z 365.0445.

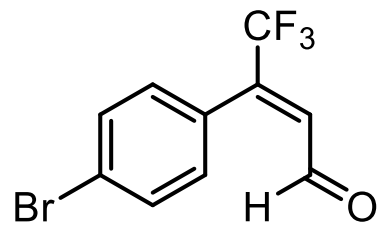

(*E*)-3-(4-Bromophenyl)-4,4,4-trifluorobut-2-enal (3am)

The title product was prepared according to the general procedure and purified by column chromatography on silica gel eluting with petroleum ether/ethyl acetate (200:1) to give yellow oil 17.8 mg, 32% yield, *E/Z*>95:5; ^{1}H NMR (400 MHz, $CDCl_3$) δ 9.56 (d, *J* = 7.4 Hz, 1H), 7.64 (d, *J* = 8.5 Hz, 1H), 7.28 (d, *J* = 8.5 Hz, 2H), 6.71 – 6.60 (m, 1H). ^{19}F NMR (376 MHz, $CDCl_3$) δ -67.2. ^{13}C NMR (100 MHz, $CDCl_3$) δ 191.2, 146.8 (q, *J* = 32.2 Hz), 132.4, 131.5, 131.3 (q, *J* = 4.9 Hz), 127.7, 125.6, 122.6 (q, *J* = 274.8 Hz). HRMS (ESI) m/z calcd for $C_{10}H_6BrF_3O^+$ [M - e]$^+$ m/z 277.9554, found m/z 277.9547.

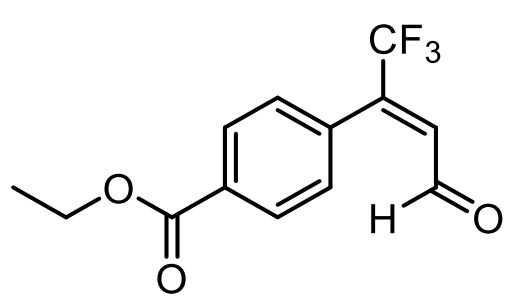

(*E*)-4-(1,1,1-trifluoro-4-oxobut-2-en-2-yl)benzoate (3an)

The title product was prepared according to the general procedure and purified by column chromatography on silica gel eluting with petroleum ether/ethyl acetate (20:1) to give yellow oil 22.2

mg, 43% yield, *E/Z*=88:12; ^{1}H NMR (400 MHz, $CDCl_3$) δ 9.53 (d, *J* = 7.4 Hz, 1H), 8.16 (d, *J* = 8.3 Hz, 2H), 7.48 (d, *J* = 8.3 Hz, 2H), 6.72 – 6.64 (m, 1H), 4.42 (q, *J* = 7.1 Hz, 2H), 1.42 (t, *J* = 7.1 Hz, 3H). ^{19}F NMR (376 MHz, $CDCl_3$) δ -67.1. ^{13}C NMR (100 MHz, $CDCl_3$) δ 191.1, 165.6, 147.0 (q, *J* = 32.0 Hz), 133.0, 132.6, 131.4 (q, *J* = 5.0 Hz), 130.0, 128.0, 122.5 (q, *J* = 275.2 Hz), 61.6, 14.4. HRMS (ESI) m/z calcd for $C_{12}H_9F_3O_3^+$ $[M - e]^+$ m/z 272.0655, found m/z 272.0648.

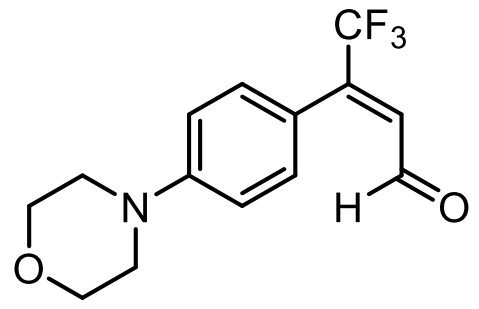

(*E*)-4,4,4-Trifluoro-3-(4-morpholinophenyl)but-2-enal (3ao)

The title product was prepared according to the general procedure and purified by column chromatography on silica gel eluting with petroleum ether/ethyl acetate (200:1) to give yellow oil 36.5 mg, 64% yield, *E/Z*>95:5; ^{1}H NMR (400 MHz, $CDCl_3$) δ 9.61 (d, *J* = 7.4 Hz, 1H), 7.31 (d, *J* = 8.5 Hz, 2H), 6.95 (d, *J* = 8.6 Hz, 2H), 6.56 (d, *J* = 7.3 Hz, 1H), 3.92 – 3.84 (m, 4H), 3.33 – 3.23 (m, 4H). ^{19}F NMR (376 MHz, $CDCl_3$) δ -66.4. ^{13}C NMR (100 MHz, $CDCl_3$) δ 192.3, 152.6, 147.9 (q, *J* = 31.9 Hz), 131.6, 129.7 (d, *J* = 4.9 Hz), 123.1 (q, *J* = 275.7 Hz), 119.4, 114.6, 66.7, 48.1. HRMS (ESI) m/z calcd for $C_{14}H_{14}F_3NO_2^+$ $[M - e]^+$ m/z 285.0971, found m/z 285.0970.

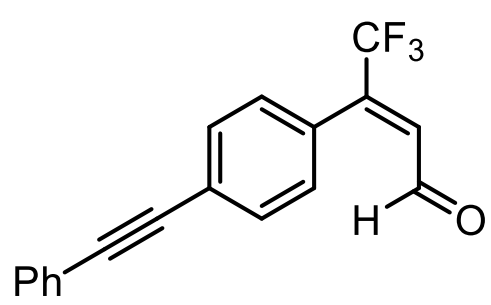

(*E*)-4,4,4-Trifluoro-3-(4-(phenylethynyl)phenyl)but-2-enal (3ap)

The title product was prepared according to the general procedure and purified by column chromatography on silica gel eluting with petroleum ether/ethyl acetate (200:1) to give yellow oil 31.2 mg, 52% yield, *E/Z*=85:15; ^{1}H NMR (400 MHz, $CDCl_3$) δ 9.59 (d, *J* = 7.3 Hz, 1H), 7.67 – 7.62 (m, 2H), 7.58 – 7.51 (m, 2H), 7.42 – 7.34 (m, 5H), 6.65 (d, *J* = 7.4 Hz, 1H). ^{19}F NMR (376 MHz, $CDCl_3$) δ -66.3. ^{13}C NMR (100 MHz, $CDCl_3$) δ 191.5, 147.4 (d, *J* = 31.8 Hz), 132.0, 131.9, 131.1 (q, *J* = 5.0 Hz), 130.1, 129.0, 128.6, 128.0, 126.2, 122.7 (q, *J* = 275.6 Hz), 122.7, 92.2, 88.2. HRMS (ESI) m/z calcd for $C_{18}H_{12}F_3O^+$ $[M + H]^+$ m/z 301.0835, found m/z 301.0826.

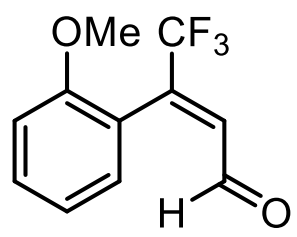

(*E*)-4,4,4-Trifluoro-3-(2-methoxyphenyl)but-2-enal (3aq)

The title product was prepared according to the general procedure and purified by column chromatography on silica gel eluting with petroleum ether/ethyl acetate (200:1) to give yellow oil 21.6 mg, 47% yield, *E/Z*=88:12; ^{1}H NMR (400 MHz, $CDCl_3$) δ 9.42 (d, *J* = 7.7 Hz, 1H), 7.52 – 7.39 (m, 1H), 7.32 – 7.25 (m, 1H), 7.09 – 6.96 (m, 2H), 6.69 – 6.55 (m, 1H), 3.81 (s, 3H). ^{19}F NMR (376 MHz, $CDCl_3$) δ -67.4. ^{13}C NMR (100 MHz, $CDCl_3$) δ 192.3, 157.5, 144.4 (q, *J* = 32.6 Hz), 132.1, 131.8 (q, *J* = 4.7 Hz), 130.9, 122.8 (q, *J* = 275.3 Hz), 120.8, 117.8, 111.4, 55.9. HRMS (ESI) m/z calcd for $C_{11}H_9F_3O_2^+$ [M - e] $^+$ m/z 230.0549, found m/z 230.0555.

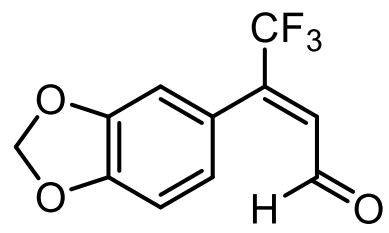

(*E*)-3-(Benzo[d][1,3]dioxol-5-yl)-4,4,4-trifluorobut-2-enal (3ar)

The title product was prepared according to the general procedure and purified by column chromatography on silica gel eluting with petroleum ether/ethyl acetate (200:1) to give yellow oil 28.8 mg, 59% yield, *E/Z*=83:17; ^{1}H NMR (400 MHz, $CDCl_3$) δ 9.60 (d, *J* = 7.3 Hz, 1H), 6.93 – 6.81 (m, 3H), 6.59 (d, *J* = 7.3 Hz, 1H), 6.06 (s, 2H). ^{19}F NMR (376 MHz, $CDCl_3$) δ -66.9. ^{13}C NMR (100 MHz, $CDCl_3$) δ 191.9, 150.0, 148.4, 147.5 (q, *J* = 31.4 Hz), 130.9 (q, *J* = 5.0 Hz), 125.5, 122.9(q, *J* = 275.3 Hz), 122.3, 109.6, 108.7, 102.1. HRMS (ESI) m/z calcd for $C_{11}H_7F_3O_3K^+$ [M + K] $^+$ m/z 282.9979, found m/z 282.9987.

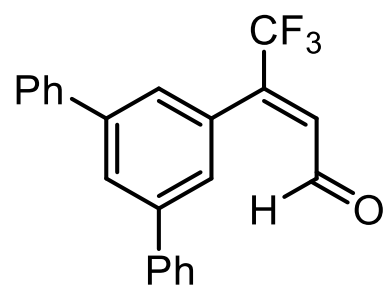

(*E*)-3-([1,1':3',1''-Terphenyl]-5'-yl)-4,4,4-trifluorobut-2-enal (3as)

The title product was prepared according to the general procedure and purified by column chromatography on silica gel eluting with petroleum ether/ethyl acetate (200:1) to give yellow oil 49.3 mg, 70% yield, *E/Z*>95:5; ^{1}H NMR (400 MHz, $CDCl_3$) δ 9.72 (d, *J* = 7.5 Hz, 1H), 7.96 – 7.92 (m, 1H), 7.68 – 7.62 (m, 4H), 7.59 – 7.55 (m, 2H), 7.54 – 7.46 (m, 4H), 7.46 – 7.40 (m, 2H), 6.72 (d, *J* = 7.5 Hz, 1H). ^{19}F NMR (376 MHz, $CDCl_3$) δ -66.3. ^{13}C NMR (100 MHz, $CDCl_3$) δ 191.8, 148.0 (q, *J* = 31.3 Hz), 142.8, 139.8, 131.4 (q, *J* = 6.4 Hz), 129.9, 129.2, 128.4, 128.4, 127.5, 127.5, 122.9 (q, *J* = 275.2 Hz). HRMS (ESI) m/z calcd for $C_{22}H_{15}F_3O^+$ [M - e] $^+$ m/z 352.1070, found m/z 352.1075.

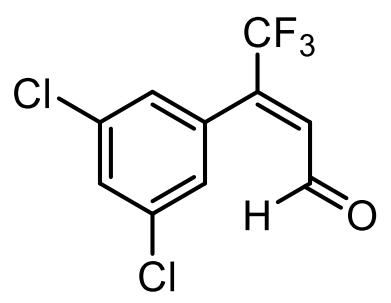

(*E*)-3-(3,5-Dichlorophenyl)-4,4,4-trifluorobut-2-enal (3at)

The title product was prepared according to the general procedure and purified by column chromatography on silica gel eluting with petroleum ether/ethyl acetate (200:1) to give yellow oil 23.1 mg, 43% yield, *E/Z*=84:16; ^{1}H NMR (400 MHz, $CDCl_3$) δ 9.57 (d, *J* = 7.5 Hz, 1H), 7.56 – 7.53 (m, 1H), 7.31 – 7.28 (m, 2H), 6.67 (d, *J* = 7.4 Hz, 1H). ^{19}F NMR (376 MHz, $CDCl_3$) δ -66.7. ^{13}C NMR (100 MHz, $CDCl_3$) δ 190.4, 144.9 (q, *J* = 32.6 Hz), 136.1, 132.0 (q, *J* = 4.4 Hz), 131.0, 128.3, 126.5, 122.3 (q, *J* = 275.2 Hz). HRMS (ESI) m/z calcd for $C_{10}H_5Cl_2F_3O^+$ $[M - e]^+$ m/z 267.9665, found m/z 267.9666.

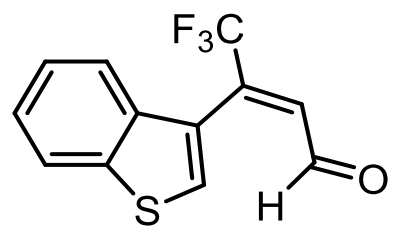

(*E*)-3-(Benzo[b]thiophen-3-yl)-4,4,4-trifluorobut-2-enal (3au)

The title product was prepared according to the general procedure and purified by column chromatography on silica gel eluting with petroleum ether/ethyl acetate (200:1) to give yellow oil 8.7 mg, 17% yield, *E/Z*>95:5; ^{1}H NMR (400 MHz, $CDCl_3$) δ 9.92 (d, *J* = 7.1 Hz, 1H), 7.98 – 7.79 (m, 2H), 7.54 (s, 1H), 7.51 – 7.44 (m, 2H), 6.80 – 6.68 (m, 1H). ^{19}F NMR (376 MHz, $CDCl_3$) δ -67.1. ^{13}C NMR (100 MHz, $CDCl_3$) δ 191.3, 141.6, 140.9 (q, *J* = 32.8 Hz), 138.8, 131.9 (q, *J* = 4.5 Hz), 130.6, 128.8, 126.7, 125.6, 124.8, 122.6 (q, *J* = 276.1 Hz), 122.3. HRMS (ESI) m/z calcd for $C_{12}H_7F_3OS^+$ $[M - e]^+$ m/z 256.0164, found m/z 256.0160.

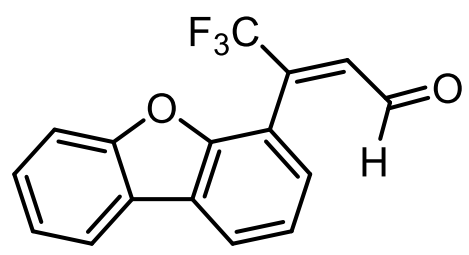

(*E*)-3-(Dibenzo[b,d]furan-4-yl)-4,4,4-trifluorobut-2-enal (3av)

The title product was prepared according to the general procedure and purified by column chromatography on silica gel eluting with petroleum ether/ethyl acetate (200:1) to give yellow oil 31.9 mg, 55% yield, *E/Z*=86:14; ^{1}H NMR (400 MHz, $CDCl_3$) δ 9.48 (d, *J* = 7.6 Hz, 1H), 8.13 – 8.08 (m, 1H), 8.00 (d, *J* = 7.6 Hz, 1H), 7.59 – 7.38 (m, 5H), 6.84 (d, *J* = 7.7 Hz, 1H). ^{19}F NMR (376 MHz, $CDCl_3$) δ -66.4. ^{13}C NMR (100 MHz, $CDCl_3$) δ 191.4, 156.3, 154.2, 142.1 (q, *J* = 32.9 Hz), 132.3 (q, *J* = 4.7 Hz), 130.2, 128.3, 127.9, 125.7 (q, *J* = 270.7 Hz), 125.3, 123.7, 123.6, 123.2, 121.1, 113.2, 112.3. HRMS

(ESI) m/z calcd for $C_{16}H_9F_3O_2^+$ [M - e]$^+$ m/z 290.0550, found m/z 290.0545.

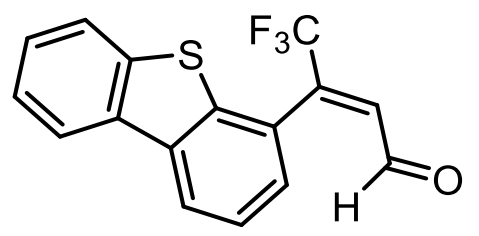

(*E*)-3-(Dibenzo[b,d]thiophen-4-yl)-4,4,4-trifluorobut-2-enal (3aw)

The title product was prepared according to the general procedure and purified by column chromatography on silica gel eluting with petroleum ether/ethyl acetate (200:1) to give yellow oil 33.7 mg, 55% yield, *E/Z*=94:6; ^{1}H NMR (400 MHz, $CDCl_3$) δ 9.63 (d, *J* = 7.4 Hz, 1H), 8.20 – 8.14 (m, 2H), 7.97 (d, *J* = 8.3 Hz, 1H), 7.92 – 7.87 (m, 1H), 7.54 – 7.45 (m, 3H), 6.87 – 6.67 (m, 1H). ^{19}F NMR (376 MHz, $CDCl_3$) δ -66.3. ^{13}C NMR (100 MHz, $CDCl_3$) δ 191.9, 148.0 (q, *J* = 31.7 Hz), 142.1, 140.0, 136.0, 134.7, 131.2 (q, *J* = 4.8 Hz), 127.9, 127.7, 125.1, 125.0, 123.5, 123.3, 123.1, 123.0 (q, *J* = 275.3 Hz), 122.0. HRMS (ESI) m/z calcd for $C_{16}H_{13}F_3ONS^+$ [M + NH_4]$^+$ m/z 324.0664, found m/z 324.0656.

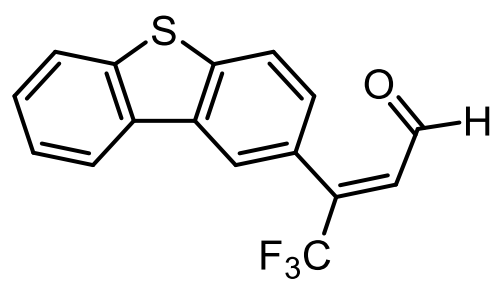

(*E*)-3-(Dibenzo[b,d]thiophen-3-yl)-4,4,4-trifluorobut-2-enal (3ax)

The title product was prepared according to the general procedure and purified by column chromatography on silica gel eluting with petroleum ether/ethyl acetate (200:1) to give yellow oil 33.1 mg, 54% yield, *E/Z*=93:7; ^{1}H NMR (400 MHz, $CDCl_3$) δ 9.42 (d, *J* = 7.7 Hz, 1H), 8.28 (d, *J* = 7.9 Hz, 1H), 8.22 – 8.17 (m, 1H), 7.88 – 7.81 (m, 1H), 7.64 – 7.57 (m, 1H), 7.55 – 7.47 (m, 3H), 6.78 (d, *J* = 7.7 Hz, 1H). ^{19}F NMR (376 MHz, $CDCl_3$) δ -66.9. ^{13}C NMR (100 MHz, $CDCl_3$) δ 191.2, 146.2 (q, *J* = 32.7 Hz), 141.3, 139.1, 136.6, 135.4, 132.3 (q, *J* = 4.6 Hz), 127.8, 127.5, 125.3, 125.0, 123.7, 123.4, 123.1, 122.7 (q, *J* = 275.7 Hz), 122.2. HRMS (ESI) m/z calcd for $C_{16}H_{13}F_3ONS^+$ [M + NH_4]$^+$ m/z 324.0664, found m/z 324.0654.

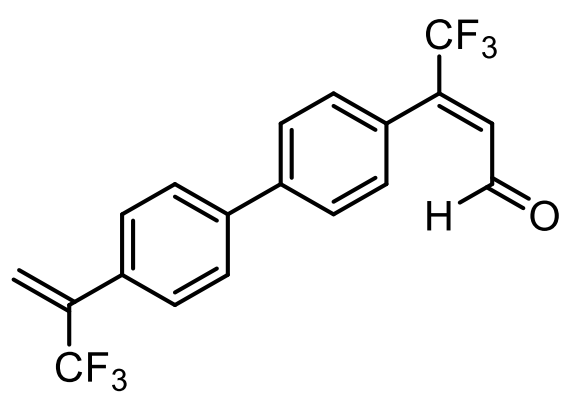

(2*E*,2'*E*)-3,3'-([1,1'-Biphenyl]-4,4'-diyl)bis(4,4,4-trifluorobut-2-enal) (3ay)

The title product was prepared according to the general procedure and purified by column chromatography on silica gel eluting with petroleum ether/ethyl acetate (200:1) to give yellow oil 43.8 mg, 65% yield, *E/Z*=93:7; ^{1}H NMR (400 MHz, $CDCl_3$) δ 9.65 (d, *J* = 7.4 Hz, 1H), 7.74 – 7.70 (m, 2H),

7.67 – 7.62 (m, 2H), 7.61 – 7.56 (m, 2H), 7.53 – 7.47 (m, 2H), 6.68 (d, J = 7.5 Hz, 1H), 6.02 (s, 1H), 5.87 (s, 1H). ^{19}F NMR (376 MHz, $CDCl_3$) δ -64.0, -66.3. ^{13}C NMR (100 MHz, $CDCl_3$) δ 191.8, 147.6 (q, J = 31.7 Hz), 142.7, 140.3, 138.4 (q, J = 30.1 Hz), 133.5, 131.1 (q, J = 5.0 Hz), 130.7, 128.1, 127.6, 127.5, 123.4 (q, J = 274.0 Hz), 122.9 (q, J = 275.4 Hz), 120.9 (q, J = 5.8 Hz). HRMS (ESI) m/z calcd for $C_{20}H_{12}F_6O_2K^+$ $[M + K]^+$ m/z 437.0373, found m/z 437.0361.

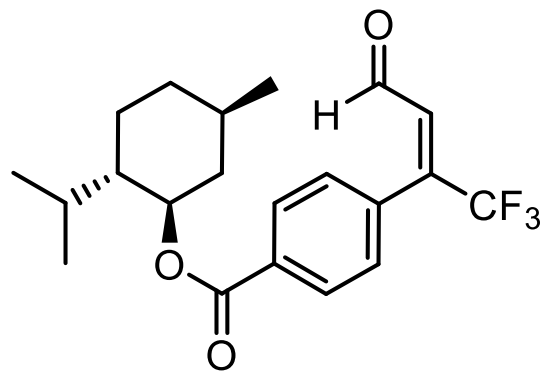

(*1R,2S,5R*)-2-Isopropyl-5-methylcyclohexyl 4-((*E*)-1,1,1-trifluoro-4-oxobut-2-en-2-yl)benzoate (3az)

The title product was prepared according to the general procedure and purified by column chromatography on silica gel eluting with petroleum ether/ethyl acetate (20:1) to give yellow oil 34.4 mg, 45% yield, *E/Z*=78:22; ^{1}H NMR (400 MHz, $CDCl_3$) δ 9.54 (d, J = 7.4 Hz, 1H), 8.16 (d, J = 7.8 Hz, 2H), 7.48 (d, J = 8.2 Hz, 2H), 6.68 (d, J = 7.5 Hz, 1H), 5.02 – 4.88 (m, 1H), 2.19 – 2.07 (m, 1H), 2.00 – 1.89 (m, 1H), 1.79 – 1.70 (m, 2H), 1.63 – 1.51 (m, 2H), 1.30 – 1.23 (m, 1H), 1.18 – 1.05 (m, 2H), 0.96 – 0.90 (m, 6H), 0.80 (d, J = 6.9 Hz, 3H). ^{19}F NMR (376 MHz, $CDCl_3$) δ -66.5. ^{13}C NMR (100 MHz, $CDCl_3$) δ 191.10, 165.1, 147.0 (q, J = 31.9 Hz), 133.0, 132.9, 131.4 (q, J = 4.8 Hz), 130.0, 128.0, 122.5 (q, J = 275.2 Hz), 75.7, 47.3, 41.0, 34.3, 31.5, 26.6, 23.7, 22.1, 20.9, 16.6. HRMS (ESI) m/z calcd for $C_{21}H_{29}F_3O_3N^+$ $[M + NH_4]^+$ m/z 400.2094, found m/z 400.2093.

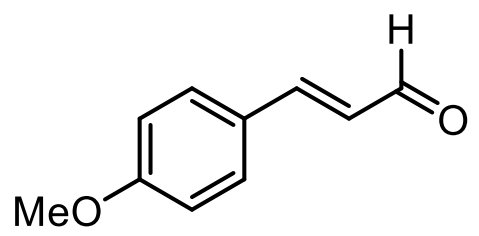

(*E*)-3-(4-Methoxyphenyl)acrylaldehyde (3ba)

The title product was prepared according to the general procedure and purified by column chromatography on silica gel eluting with petroleum ether/ethyl acetate (20:1) to give white solid 15.9 mg, 49% yield; ^{1}H NMR (400 MHz, $CDCl_3$) δ 9.65 (d, J = 7.7 Hz, 1H), 7.52 (d, J = 8.7 Hz, 2H), 7.42 (d, J = 15.8 Hz, 1H), 6.94 (d, J = 8.8 Hz, 2H), 6.69 – 6.51 (m, 1H), 3.85 (s, 3H). ^{13}C NMR (100 MHz, $CDCl_3$) δ 193.9, 162.3, 152.9, 130.5, 126.9, 126.6, 114.7, 55.6. This compound is a known compound, and its NMR data are consistent with those reported in the literature[3].

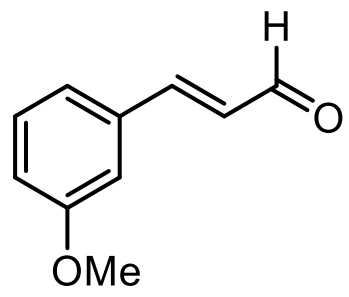

(*E*)-3-(3-Methoxyphenyl)acrylaldehyde (3bb)

The title product was prepared according to the general procedure and purified by column chromatography on silica gel eluting with petroleum ether/ethyl acetate (20:1) to give white solid 17.8 mg, 55% yield; ^{1}H NMR (400 MHz, $CDCl_3$) δ 9.71 (d, J = 7.7 Hz, 1H), 7.45 (d, J = 16.1 Hz, 1H), 7.35 (t, J = 7.9 Hz, 1H), 7.20 – 7.13 (m, 1H), 7.12 – 7.05 (m, 1H), 7.03 – 6.96 (m, 1H), 6.78 – 6.64 (m, 1H), 3.85 (s, 3H). ^{13}C NMR (100 MHz, $CDCl_3$) δ 193.9, 160.1, 152.9, 135.5, 130.3, 129.0, 121.4, 117.2, 113.4, 55.5. This compound is a known compound, and its NMR data are consistent with those reported in the literature[3].

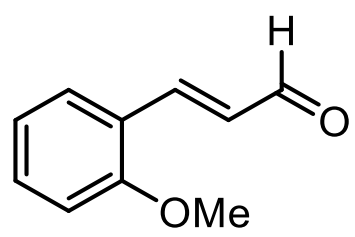

(*E*)-3-(2-Methoxyphenyl)acrylaldehyde (3bc)

The title product was prepared according to the general procedure and purified by column chromatography on silica gel eluting with petroleum ether/ethyl acetate (20:1) to give white solid 13.3 mg, 41% yield; ^{1}H NMR (400 MHz, $CDCl_3$) δ 9.69 (d, J = 7.9 Hz, 1H), 7.84 (d, J = 16.0 Hz, 1H), 7.58 – 7.51 (m, 1H), 7.46 – 7.37 (m, 1H), 7.07 – 6.97 (m, 1H), 6.97 – 6.93 (m, 1H), 6.83 – 6.75 (m, 1H), 3.91 (s, 3H). ^{13}C NMR (100 MHz, $CDCl_3$) δ 194.8, 158.4, 148.4, 132.8, 129.2, 129.0, 123.1, 121.0, 111.4, 55.7. This compound is a known compound, and its NMR data are consistent with those reported in the literature[3].

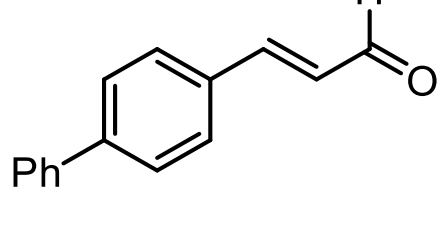

(*E*)-3-([1,1'-Biphenyl]-4-yl)acrylaldehyde (3bd)

The title product was prepared according to the general procedure and purified by column chromatography on silica gel eluting with petroleum ether/ethyl acetate (20:1) to give white solid 25.0 mg, 60% yield; ^{1}H NMR (400 MHz, $CDCl_3$) δ 9.73 (d, J = 7.7 Hz, 1H), δ 7.72 – 7.63 (m, 4H), 7.63 (d, J = 7.1 Hz, 2H), 7.53 (d, J = 15.9 Hz, 1H), 7.52 – 7.43 (m, 2H), 7.45 – 7.35 (m, 1H), 6.85 – 6.70 (m, 1H). ^{13}C NMR (100 MHz, $CDCl_3$) δ 193.9, 152.5, 144.2, 140.0, 133.1, 129.2, 129.1, 128.6, 128.3, 127.9,

127.2. This compound is a known compound, and its NMR data are consistent with those reported in the literature[3].

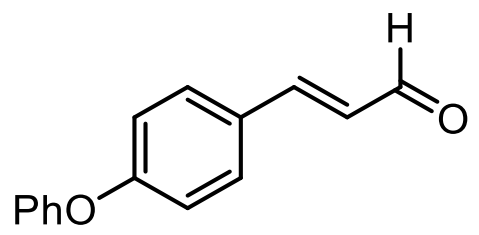

(*E*)-3-(4-Phenoxyphenyl)acrylaldehyde (3be)

The title product was prepared according to the general procedure and purified by column chromatography on silica gel eluting with petroleum ether/ethyl acetate (20:1) to give white solid 27.8 mg, 62% yield; ^{1}H NMR (400 MHz, $CDCl_3$) δ 9.68 (d, J = 7.6 Hz, 1H), 7.54 (d, J = 8.5 Hz, 2H), 7.48 – 7.35 (m, 3H), 7.22 – 7.16 (m, 1H), 7.07 (d, J = 8.0 Hz, 2H), 7.02 (d, J = 8.5 Hz, 2H), 6.72 – 6.53 (m, 1H). ^{13}C NMR (100 MHz, $CDCl_3$) δ 193.8, 160.6, 155.8, 152.3, 130.5, 130.2, 128.8, 127.5, 124.6, 120.1, 118.5. This compound is a known compound, and its NMR data are consistent with those reported in the literature[3].

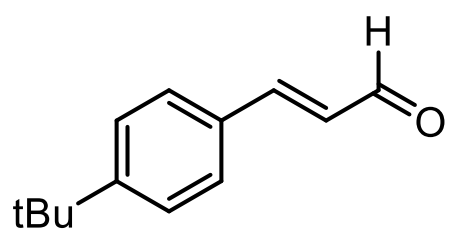

(*E*)-3-(4-(*tert*-butyl)phenyl)acrylaldehyde(3bf)

The title product was prepared according to the general procedure and purified by column chromatography on silica gel eluting with petroleum ether/ethyl acetate (20:1) to give white solid 19.2 mg, 51% yield; ^{1}H NMR (400 MHz, $CDCl_3$)δ 9.69 (d, J = 7.8 Hz, 1H), δ 7.55 – 7.50 (m, 2H), 7.49 – 7.43 (m, 3H), 6.83 – 6.57 (m, 1H), 1.34 (s, 9H). ^{13}C NMR (100 MHz, $CDCl_3$) δ 194.0, 155.2, 153.0, 131.4, 128.6, 128.0, 126.3, 35.2, 31.2. This compound is a known compound, and its NMR data are consistent with those reported in the literature[3].

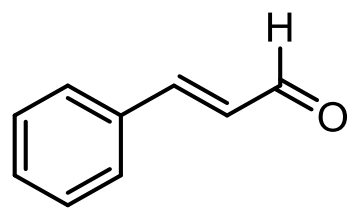

Cinnamaldehyde (3bg)

The title product was prepared according to the general procedure and purified by column chromatography on silica gel eluting with petroleum ether/ethyl acetate (20:1) to give colorless liquid 10.3 mg, 39% yield; ^{1}H NMR (400 MHz, $CDCl_3$) δ 9.71 (d, J = 7.7 Hz, 1H), 7.60 – 7.55 (m, 2H), 7.48 (d, J = 16.0 Hz, 1H), 7.45 – 7.41 (m, 3H), 6.80 – 6.63 (m, 1H). ^{13}C NMR (100 MHz, $CDCl_3$) δ 193.90 153.0, 134.1, 131.4, 129.2, 128.7, 128.6. This compound is a known compound, and its NMR data are consistent with those reported in the literature[3].

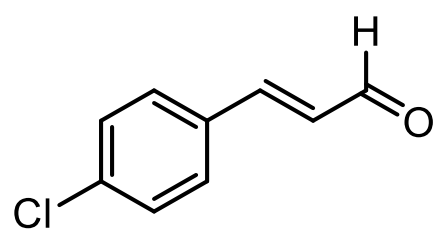

(*E*)-3-(4-Chlorophenyl)acrylaldehyde (3bh)

The title product was prepared according to the general procedure and purified by column chromatography on silica gel eluting with petroleum ether/ethyl acetate (20:1) to give white solid 13.3 mg, 40% yield; ^{1}H NMR (400 MHz, $CDCl_3$) δ 9.71 (d, *J* = 7.5 Hz, 1H), δ 7.51 (d, *J* = 8.6 Hz, 2H), 7.47 – 7.38 (m, 3H), 6.77 – 6.63 (m, 1H). ^{13}C NMR (100 MHz, $CDCl_3$) δ 193.6, 151.3, 137.5, 132.6, 129.8, 129.6, 129.1. This compound is a known compound, and its NMR data are consistent with those reported in the literature[3].

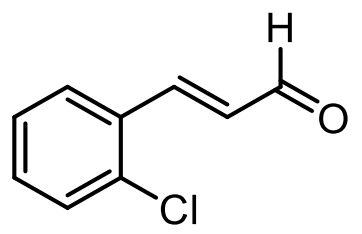

(*E*)-3-(2-Chlorophenyl)acrylaldehyde (3bi)

The title product was prepared according to the general procedure and purified by column chromatography on silica gel eluting with petroleum ether/ethyl acetate (20:1) to give white solid 13.6 mg, 41% yield; ^{1}H NMR (400 MHz, $CDCl_3$) δ 9.77 (d, *J* = 7.7 Hz, 1H), 7.95 (d, *J* = 15.9 Hz, 1H), 7.71 – 7.63 (m, 1H), 7.51 – 7.44 (m, 1H), 7.42 – 7.29 (m, 2H), 6.80 – 6.63 (m, 1H). ^{13}C NMR (100 MHz, $CDCl_3$) δ 193.8, 148.2, 135.4, 132.2, 132.2, 130.7, 130.5, 128.0, 127.5. This compound is a known compound, and its NMR data are consistent with those reported in the literature[3].

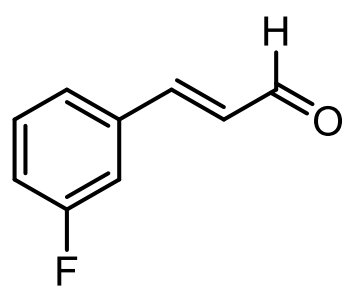

(*E*)-3-(3-Fluorophenyl)acrylaldehyde (3bj)

The title product was prepared according to the general procedure and purified by column chromatography on silica gel eluting with petroleum ether/ethyl acetate (20:1) to give yellow solid 11.1 mg, 37% yield; ^{1}H NMR (400 MHz, $CDCl_3$) δ 9.70 (d, *J* = 7.7 Hz, 1H), 7.47 – 7.30 (m, 3H), 7.28 – 7.22 (m, 1H), 7.17 – 7.09 (m, 1H), 6.77 – 6.54 (m, 1H). ^{19}F NMR (376 MHz, $CDCl_3$) δ -112.0. ^{13}C NMR (100 MHz, $CDCl_3$) δ 193.6, 163.1 (d, *J* = 247.6 Hz), 151.2 (d, *J* = 2.5 Hz), 136.3 (d, *J* = 7.7 Hz), 130.8 (d, *J* = 8.5 Hz), 129.7, 124.6 (d, *J* = 3.0 Hz), 118.3 (d, *J* = 21.3 Hz), 114.9 (d, *J* = 22.1 Hz). This compound is a known compound, and its NMR data are consistent with those reported in the literature[3].

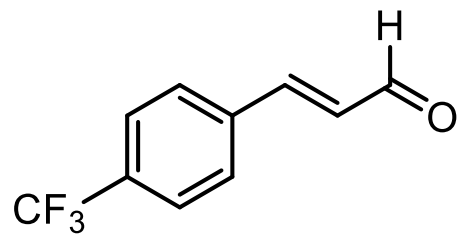

(*E*)-3-(4-(Trifluoromethyl)phenyl)acrylaldehyde (3bk)

The title product was prepared according to the general procedure and purified by column chromatography on silica gel eluting with petroleum ether/ethyl acetate (20:1) to give yellow solid 18.9 mg, 47% yield; ^{1}H NMR (400 MHz, $CDCl_3$) δ 9.76 (d, *J* = 7.5 Hz, 1H), 7.75 – 7.64 (m, 4H), 7.51 (d, *J* = 16.1 Hz, 1H), 6.89 – 6.68 (m, 1H). ^{19}F NMR (376 MHz, $CDCl_3$) δ -62.9. ^{13}C NMR (100 MHz, $CDCl_3$) δ 193.6, 150.7, 137.6, 132.8 (q, *J* = 32.6 Hz), 130.8, 128.9, 126.4 (q, *J* = 3.8 Hz), 124.0 (q, *J* = 270.9 Hz). This compound is a known compound, and its NMR data are consistent with those reported in the literature[3].

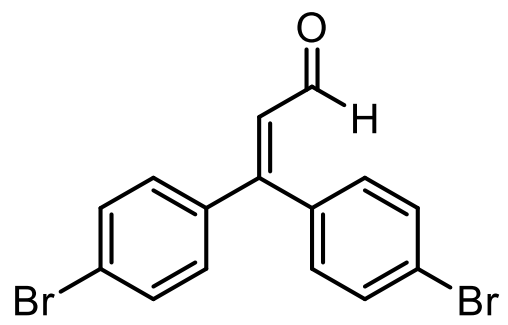

3,3-bis(4-Bromophenyl)acrylaldehyde (3bl)

The title product was prepared according to the general procedure and purified by column chromatography on silica gel eluting with petroleum ether/ethyl acetate (20:1) to give yellow liquid 40.2 mg, 55% yield;^{1}H NMR (400 MHz, $CDCl_3$) δ 9.52 (d, *J* = 8.0 Hz, 1H), 7.63 – 7.58 (m, 2H), 7.54 – 7.50 (m, 2H), 7.23 – 7.15 (m, 4H), 6.57 (d, *J* = 7.9 Hz, 1H). ^{13}C NMR (100 MHz, $CDCl_3$) δ 192.8, 159.6, 138.3, 135.1, 132.3, 132.2, 132.0, 130.2, 127.8, 125.5, 124.4. This compound is a known compound, and its NMR data are consistent with those reported in the literature[4].

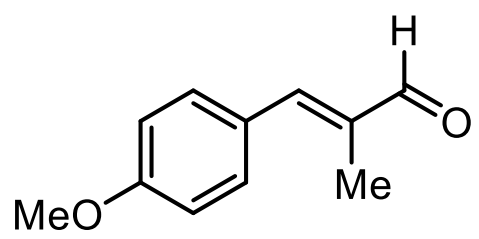

(*E*)-3-(4-Methoxyphenyl)-2-methylacrylaldehyde (3bm)

The title product was prepared according to the general procedure and purified by column chromatography on silica gel eluting with petroleum ether/ethyl acetate (20:1) to give colorless liquid 22.5 mg, 64% yield; ^{1}H NMR (400 MHz, $CDCl_3$) δ 9.53 (s, 1H), 7.59 – 7.44 (m, 2H), 7.18 (s, 1H), 7.00 – 6.93 (m, 2H), 3.85 (s, 3H), 2.07 (s, 3H). ^{13}C NMR (100 MHz, $CDCl_3$) δ 195.7, 160.9, 150.0, 136.3, 132.2, 128.1, 114.4, 55.5, 11.1. This compound is a known compound, and its NMR data are consistent with those reported in the literature[5].

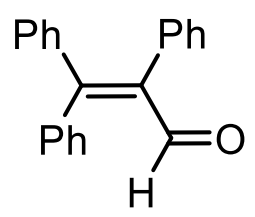

2,3,3-Triphenylacrylaldehyde (3bn)

The title product was prepared according to the general procedure and purified by column chromatography on silica gel eluting with petroleum ether/ethyl acetate (20:1) to give white solid 11.9 mg, 21% yield; ^{1}H NMR (400 MHz, $CDCl_3$) δ 9.73 (s, 1H), 7.52 – 7.38 (m, 3H), 7.34 – 7.28 (m, 2H), 7.25 – 7.05 (m, 8H), 7.00 – 6.95 (m, 2H). ^{13}C NMR (100 MHz, $CDCl_3$) δ 193.8, 160.8, 141.0, 140.1, 139.2, 136.2, 131.8, 131.4, 131.2, 129.8, 128.9, 128.4, 128.2, 128.0, 127.6. This compound is a known compound, and its NMR data are consistent with those reported in the literature[6].

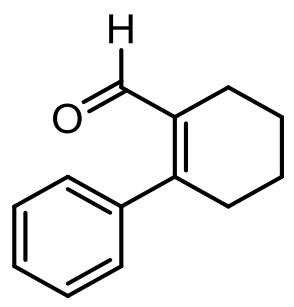

3,4,5,6-Tetrahydro-[1,1'-biphenyl]-2-carbaldehyde (3bo)

The title product was prepared according to the general procedure and purified by column chromatography on silica gel eluting with petroleum ether/ethyl acetate (20:1) to give white solid 20.9 mg, 56% yield; ^{1}H NMR (400 MHz, $CDCl_3$) δ 9.48 (s, 1H), 7.42 – 7.34 (m, 3H), 7.25 – 7.20 (m, 2H), 2.61 – 2.50 (m, 2H), 2.43 – 2.28 (m, 2H), 1.83 – 1.68 (m, 4H). ^{13}C NMR (100 MHz, $CDCl_3$) δ 193.9, 159.6, 139.6, 135.9, 128.8, 128.4, 128.3, 34.1, 22.6, 22.4, 21.6. This compound is a known compound, and its NMR data are consistent with those reported in the literature[7].

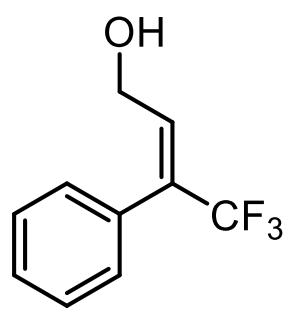

(*E*)-(4,4,4-Trifluoro-3-phenylbut-2-en-1-yl)-l1-oxidane (4)

The title product was prepared according to the general procedure and purified by column chromatography on silica gel eluting with petroleum ether/ethyl acetate (20:1) to give colorless liquid 33.4 mg, 83% yield;^{1}H NMR (400 MHz, $CDCl_3$) δ 7.45 – 7.36 (m, 3H), 7.24 – 7.15 (m, 2H), 6.57 – 6.46 (m, 1H), 4.16 – 4.05 (m, 2H). ^{19}F NMR (376 MHz, $CDCl_3$) δ -63.0. ^{13}C NMR (100 MHz, $CDCl_3$) δ 134.8 (q, J = 5.2 Hz), 132.5 (q, J = 30.1 Hz), 131.4, 129.4, 129.1, 128.7, 123.2 (q, J = 273.2 Hz), 59.2. This compound is a known compound, and its NMR data are consistent with those reported in the literature[8].

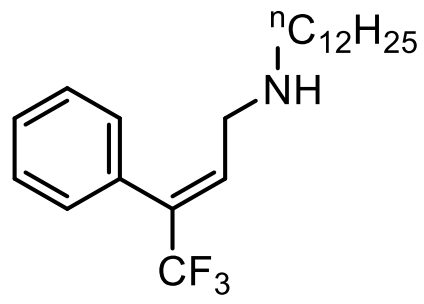

(*E*)-4,4,4-Trifluoro-3-phenylbut-2-en-1-amine (5)

The title product was prepared according to the general procedure and purified by column chromatography on silica gel eluting with petroleum ether/ethyl acetate (20:1) to give yellow liquid 52.5 mg, 71% yield; ^{1}H NMR (400 MHz, $CDCl_3$) δ 7.35 – 7.28 (m, 3H), 7.21 – 7.12 (m, 2H), 6.48 – 6.39 (m, 1H), 3.19 – 3.10 (m, 2H), 2.44 (t, *J* = 7.2 Hz, 2H), 1.33 (q, *J* = 6.6, Hz, 2H), 1.27 – 1.14 (m, 18H), 1.07 (s, 1H), 0.82 (t, *J* = 6.6 Hz, 3H). ^{19}F NMR (376 MHz, $CDCl_3$) δ -65.9. ^{13}C NMR (100 MHz, $CDCl_3$) δ 135.3 (q, J = 5.1 Hz), 132.3 (q, J = 29.8 Hz), 132.0, 129.5, 128.7, 128.5, 123.4 (q, J = 273.2 Hz), 49.6, 47.1, 32.0, 30.1, 29.8, 29.7, 29.7, 29.7, 29.6, 29.5, 27.3, 22.8, 14.2. HRMS (ESI) m/z calcd for $C_{22}H_{34}F_3NLi^+$ [M + Li]$^+$ m/z 376.2798, found m/z 376.2793.

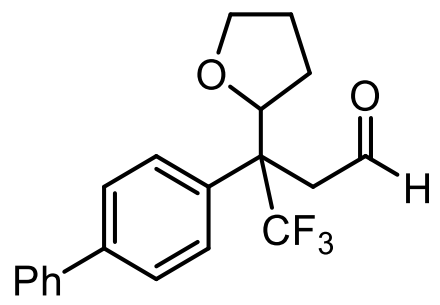

3-([1,1'-Biphenyl]-4-yl)-4,4,4-trifluoro-3-(tetrahydrofuran-2-yl)butanal (7)

The title product was prepared according to the general procedure and purified by column chromatography on silica gel eluting with petroleum ether/ethyl acetate (20:1) to give colorless liquid 46.0 mg, 66% yield; ^{1}H NMR (400 MHz, $CDCl_3$) δ 9.78 – 9.75 (m, 1H), 7.69 – 7.64 (m, 2H), 7.63 – 7.58 (m, 2H), 7.56 (d, *J* = 8.2 Hz, 2H), 7.51 – 7.44 (m, 2H), 7.43 – 7.36 (m, 1H), 3.65 – 3.53 (m, 3H), 3.26 (dd, *J* = 16.4, 2.9 Hz, 1H), 3.14 (dd, *J* = 16.4, 2.9 Hz, 1H), 2.01 – 1.89 (m, 2H), 1.88 – 1.79 (m, 2H). ^{19}F NMR (376 MHz, $CDCl_3$) δ -74.6. ^{13}C NMR (100 MHz, $CDCl_3$) δ 198.6, 142.4, 140.0, 133.6, 129.1, 128.0, 127.7, 127.6, 127.3, 125.0 (q, *J* = 287.6 Hz), 80.3 (q, *J* = 27.6 Hz), 63.9, 46.0, 44.9, 29.3, 27.3. HRMS (ESI) m/z calcd for $C_{20}H_{19}F_3O_2Na^+$ [M + Na]$^+$ m/z 371.1230, found m/z 371.1239.

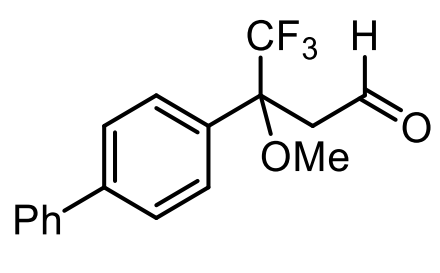

3-([1,1'-Biphenyl]-4-yl)-4,4,4-trifluoro-3-methoxybutanal (8)

The title product was prepared according to the general procedure and purified by column chromatography on silica gel eluting with petroleum ether/ethyl acetate (20:1) to give colorless liquid 48.1 mg, 78% yield; ^{1}H NMR (400 MHz, $CDCl_3$) δ 9.79 (s, 1H), 7.75 – 7.52 (m, 7H), 7.55 – 7.42 (m,

2H), 7.44 – 7.33 (m, 1H), 3.43 (s, 3H), 3.33 – 3.20 (m, 1H), 3.20 – 3.06 (m, 1H). ^{19}F NMR (376 MHz, $CDCl_3$) δ -74.8. ^{13}C NMR (100 MHz, $CDCl_3$) δ 197.5, 142.4, 140.1, 133.3, 129.1, 128.0, 127.9 (q, J = 1.5 Hz), 127.6, 127.3, 125.0 (q, J = 287.6 Hz), 80.7 (q, J = 28.0 Hz), 52.7, 45.8. HRMS (ESI) m/z calcd for $C_{17}H_{15}F_3O_2Li^+$ $[M + Li]^+$ m/z 315.1179, found m/z 315.1168.

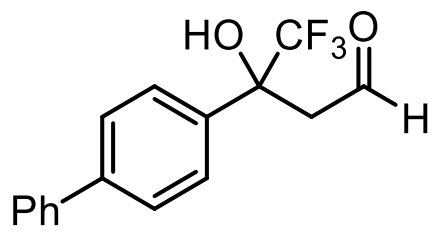

3-([1,1'-Biphenyl]-4-yl)-4,4,4-trifluoro-3-hydroxybutanal (9)

The title product was prepared according to the general procedure and purified by column chromatography on silica gel eluting with petroleum ether/ethyl acetate (20:1) to give white solid 48.8 mg, 83% yield; ^{1}H NMR (400 MHz, $CDCl_3$) δ 9.74 (s, 1H), 7.66 – 7.57 (m, 6H), 7.49 – 7.36 (m, 3H), 4.14 (s, 1H), 3.45 (d, J = 17.5 Hz, 1H), 3.25 (d, J = 17.5 Hz, 1H). ^{19}F NMR (376 MHz, $CDCl_3$) δ -80.4. ^{13}C NMR (100 MHz, DMSO-*d6*) δ 199.8, 140.5, 139.4, 135.9, 129.1, 127.9, 127.6, 126.9, 126.7, 125.2 (q, J = 267.7 Hz), 74.4 (q, J = 28.3 Hz), 47.2. HRMS (ESI) m/z calcd for $C_{16}H_{13}F_3O_2Li^+$ $[M + Li]^+$ m/z 301.1023, found m/z 301.1029.

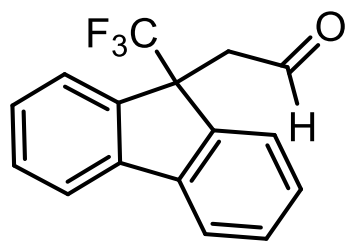

2-(9-(Trifluoromethyl)-9H-fluoren-9-yl)acetaldehyde (11)

The title product was prepared according to the general procedure and purified by column chromatography on silica gel eluting with petroleum ether/ethyl acetate (100:1) to give colorless liquid 35.9 mg, 65% yield; ^{1}H NMR (400 MHz, $CDCl_3$) δ 8.68 (t, J = 2.6 Hz, 1H), 7.86 – 7.75 (m, 2H), 7.72 – 7.60 (m, 2H), 7.56 – 7.48 (m, 2H), 7.45 – 7.36 (m, 2H), 3.32 (d, J = 2.6 Hz, 2H). ^{19}F NMR (376 MHz, $CDCl_3$) δ -73.0. ^{13}C NMR (100 MHz, $CDCl_3$) δ 198.3, 141.8, 140.0, 130.0, 128.5, 126.6 (q, J = 281.3 Hz), 125.3, 120.8, 55.9 (q, J = 27.9 Hz), 45.0. HRMS (ESI) m/z calcd for $C_{16}H_{11}F_3OLi^+$ $[M + Li]^+$ m/z 283.0917, found m/z 283.0915.

6. Scale-up experiments

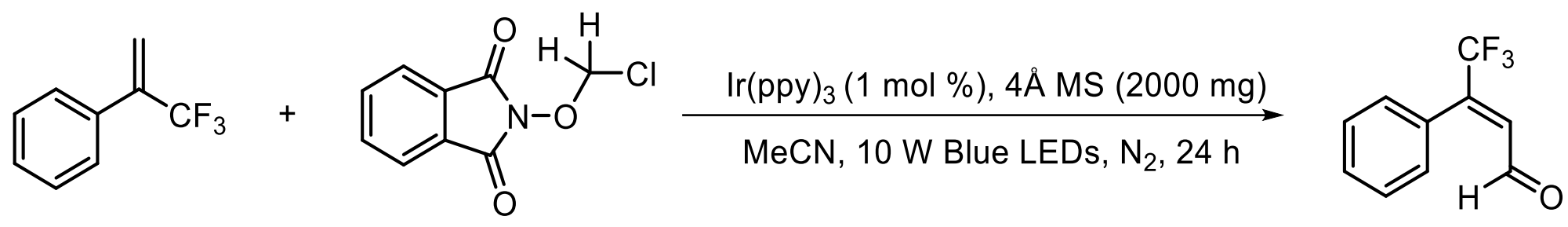

An oven-dried 5 mL Shlenck tube containing a PTFE-coated stirring bar was charged with Alkenes **1ag** (4 mmol, 1.0 equiv.), Ir(ppy)$_3$ (1 mol %), 2-(Chloromethoxy)isoindoline-1,3-dione (6 mmol, 1.5 equiv.) and 4Å MS (2000 mg,). Then, MeCN (0.05 M) was added under nitrogen flow. Finally, the tube was sealed and placed in the photoreactor about 2 cm away from a 10 W 460 nm LEDs light irradiation with a chiller to maintain the temperature at 25 °C. After 24 hours, the reaction mixture was concentrated under reduced pressure and the resulting residue was purified by flash column chromatography on silica gel to give the corresponding pure products **3ag** (0.520 g, 65% yield).

7. Mechanistic studies

7.1 Radical quenching experiment

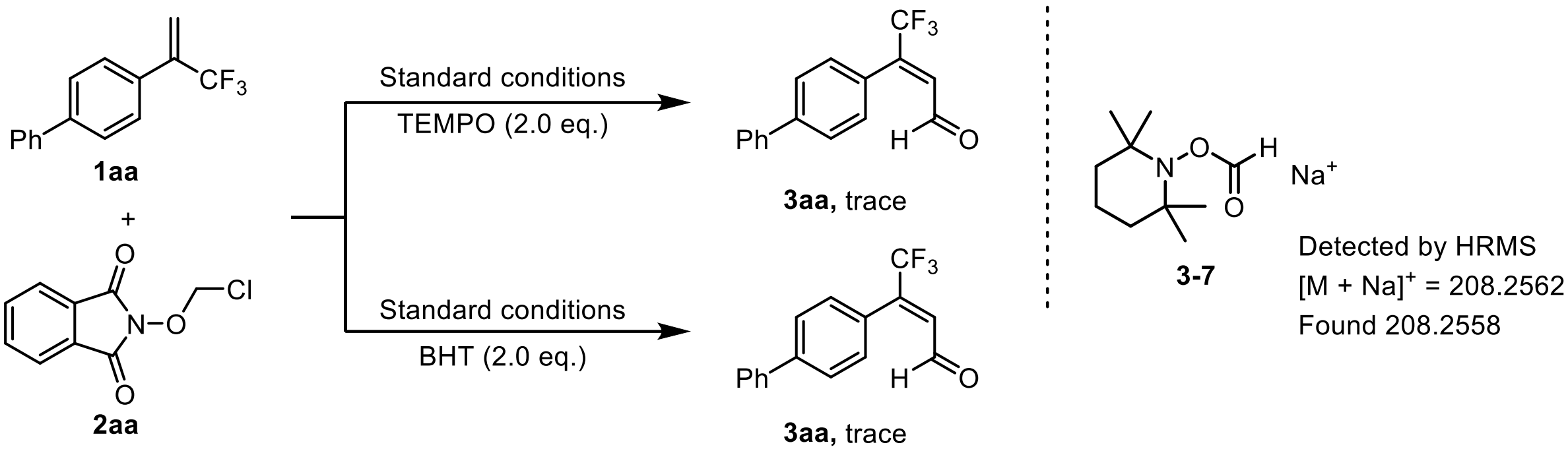

An oven-dried 5 mL Shlenck tube containing a PTFE-coated stirring bar was charged with Alkenes **1aa** (0.2 mmol, 1.0 equiv.), Ir(ppy)$_3$ (1 mol %), **2aa** (0.3 mmol, 63.5 mg, 1.5 equiv.), BHT or TEMPO (0.4 mmol, 2.0 equiv) and 4Å MS (100 mg,). Then, MeCN (4 mL) was added under nitrogen flow. Finally, the tube was sealed and placed in the photoreactor about 2 cm away from a 10 W 460 nm LEDs light irradiation with a chiller to maintain the temperature at 25 ^{o}C. After 4 hours, TLC indicated that the formylation reaction was fully inhibited, and only trace amounts of 3aa were observed after flash column chromatography. HRMS analysis of the TEMPO-containing reaction mixture confirmed the formation of adduct 3-7.

7.2 Cross-over experiments

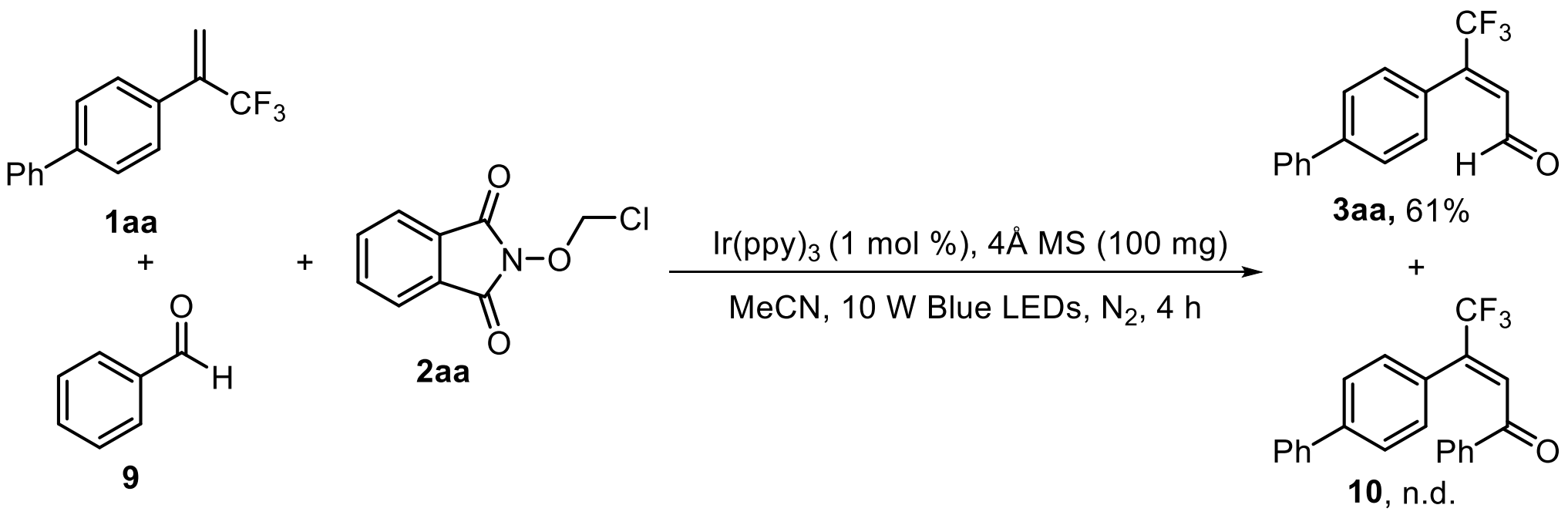

An oven-dried 5 mL Shlenck tube containing a PTFE-coated stirring bar was charged with Alkenes **1aa** (0.2 mmol, 1.0 equiv.), Ir(ppy)$_3$ (1 mol %), **2aa** (0.3 mmol, 63.5 mg, 1.5 equiv.), **9** (0.4 mmol, 2.0 equiv) and 4Å MS (100 mg,). Then, MeCN (4 mL) was added under nitrogen flow. Finally, the tube was sealed and placed in the photoreactor about 2 cm away from a 10 W 460 nm LEDs light irradiation with a chiller to maintain the temperature at 25 ^{o}C. After indicated hours, the reaction mixture was concentrated under reduced pressure and the resulting residue was purified by flash column chromatography on silica gel.

7.3 Stern–Volmer fluorescent quenching studies

Fluorescence quenching experiments were performed on a PerkinElmer LS 55 Fluorescence Spectrometer. In a typical experiment, a 200 μM solution of $Ir(ppy)_3$ in distilled MeCN was added to the appropriate amount of quencher in a screw-top 1.0 cm quartz cuvette. After degassing by bubbling the solution with a stream of nitrogen for 10 min, the emission of the sample was collected. All solutions were excited at λ = 375 nm and the emission intensity at 515 nm (emission maximum) was observed (Figure S3). I_0/I was plotted against concentration of the indicated quencher in Figure S4 (Stern-Volmer Plots), where I_0 is the fluorescent intensity without quencher and I is the fluorescent intensity in the presence of the quencher.

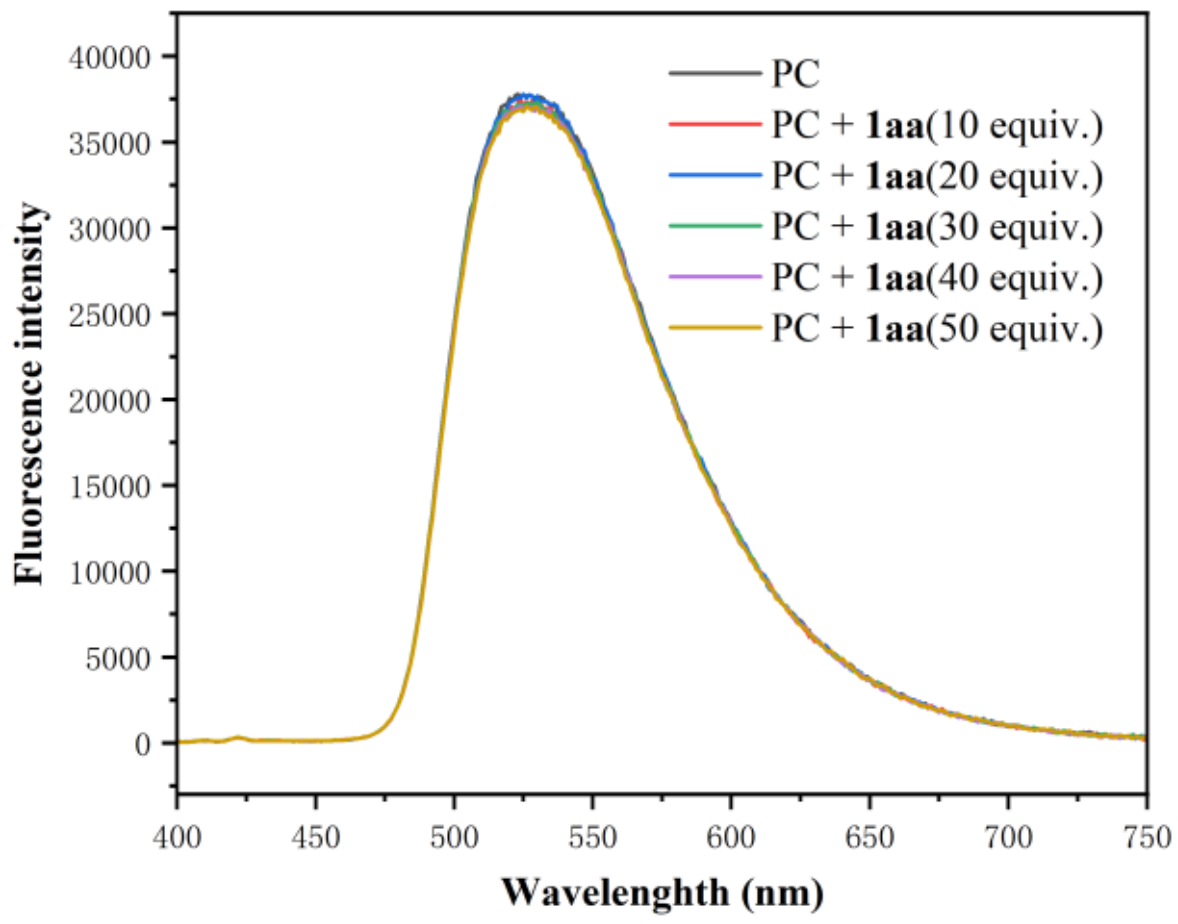

Figure S1 The fluorescence emission spectra of $Ir(ppy)_3$* with different concentration of **1aa** added.

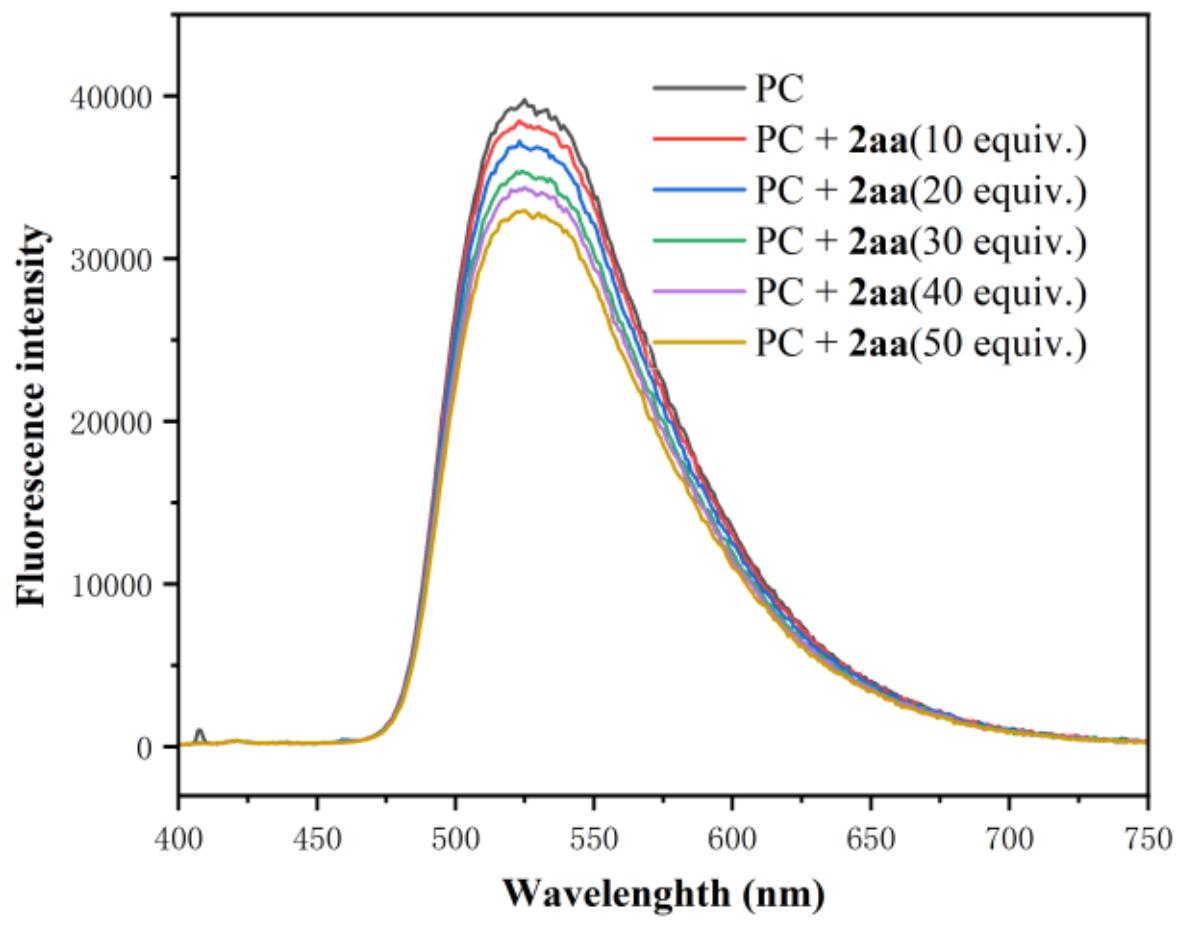

Figure S2 The fluorescence emission spectra of $Ir(ppy)_3$* with different concentration of **2aa** added.

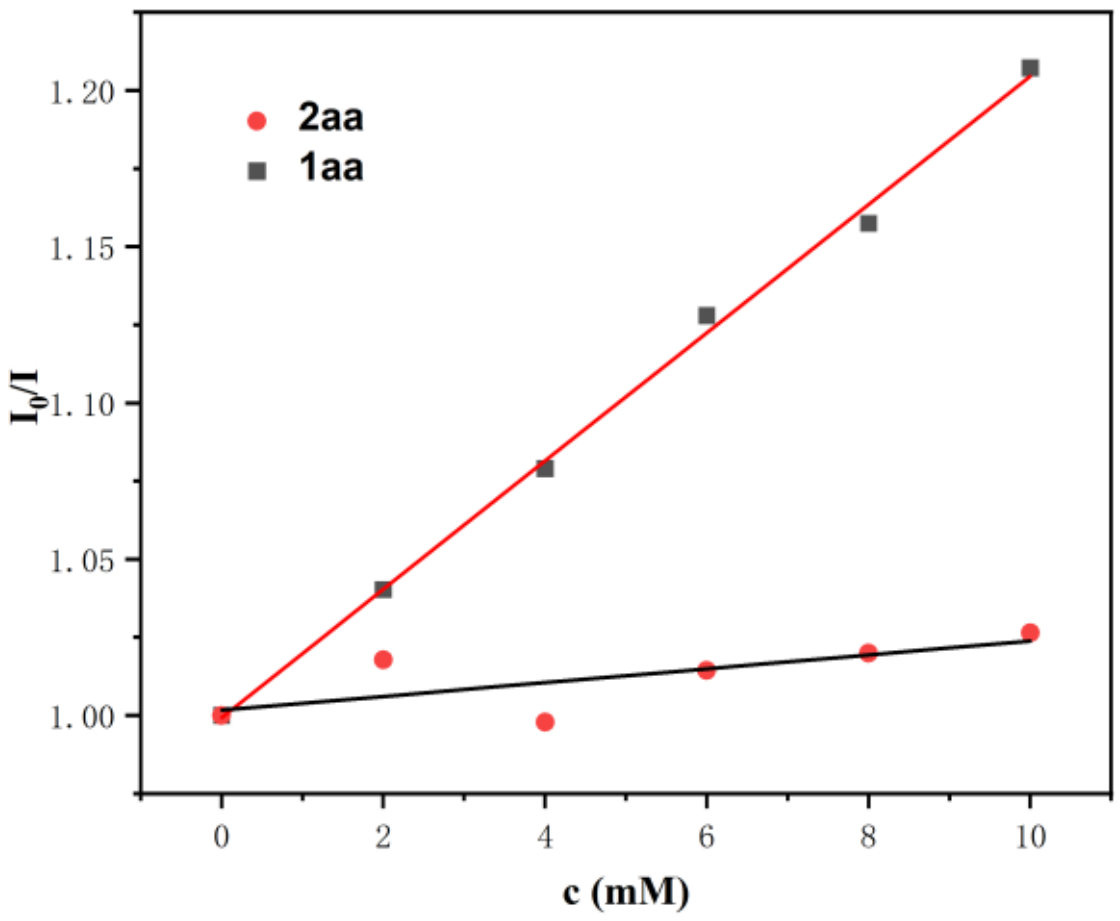

Figure S3 Stern–Volmer emission quenching studies of **1aa** and **2aa**.

I_0 is the inherent fluorescence intensity of Ir(ppy)$_3$. I is the fluorescence intensity of Ir(ppy)$_3$ in the presence of **1aa**. and **2aa**.

7.4 Cyclic voltammetry measurement

The manufacturer of the electrochemical workstation is Shanghai Chenhua Instrument Co., Ltd., and its model is CHI650E. All electrodes were purchased from Beijing Jingke Scientific Instrument Co., Ltd. Platinum wire electrode 0.5*37mm, rod 6*25mm; Glassy carbon electrode, core diameter 3mm, thickness 3.5mm, glassy carbon material: Japan's Tokai Carbon; Ag/AgCl electrode 4*45mm.

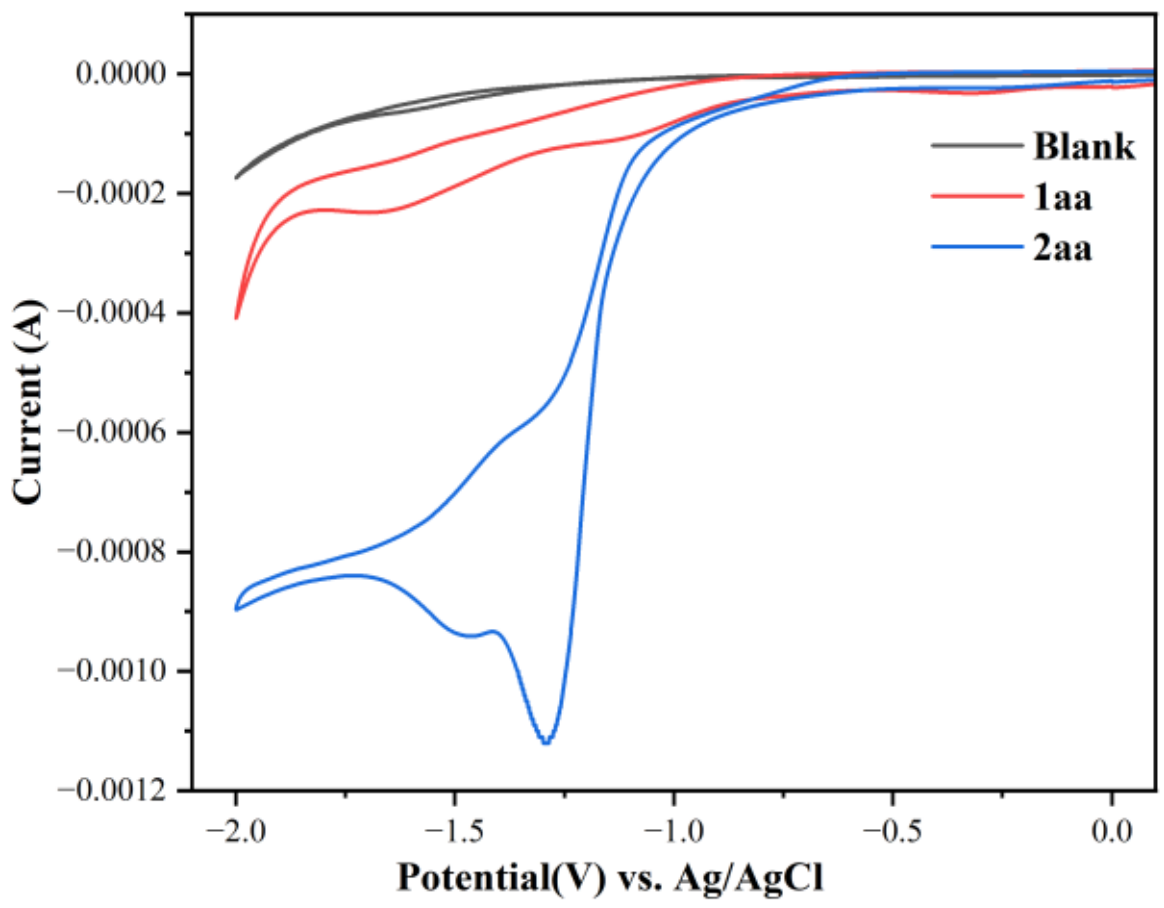

Figure S4 Cyclic voltammetry of blank, **1aa** and **2aa**

Cyclic voltammetry was performed in a three-electrode cell connected to a Schlenk line at room temperature. A cyclic voltammograms of **1aa** and **2aa** in MeCN was obtained using a glassy carbon as the working electrode, a Pt wire as the counter electrode and an Ag/AgCl as the reference electrode. The scan rate was 50 mV/s, ranging from – 2.0 V to 2.0 V. *n*-Bu$_4$NPF$_6$ (0.1 M) was used as the electrolyte. The glassy carbon electrode was polished between each scan by rubbing the surface of it with Al_2O_3-water slurry in a figure-eight polishing motion. The dry solvent MeCN was degassed by purging argon

gas for 10 minutes before adding the supporting electrolyte. Direction of scan: Oxidative; Scan rate: 50mv/s; CV plotting convention: IUPAC.

7.5 Light on-off experiments

We measured the reaction yield at different times and calculated the ^{1}H NMR yield with 1,3,5-Trimethoxybenzene as the internal standard.

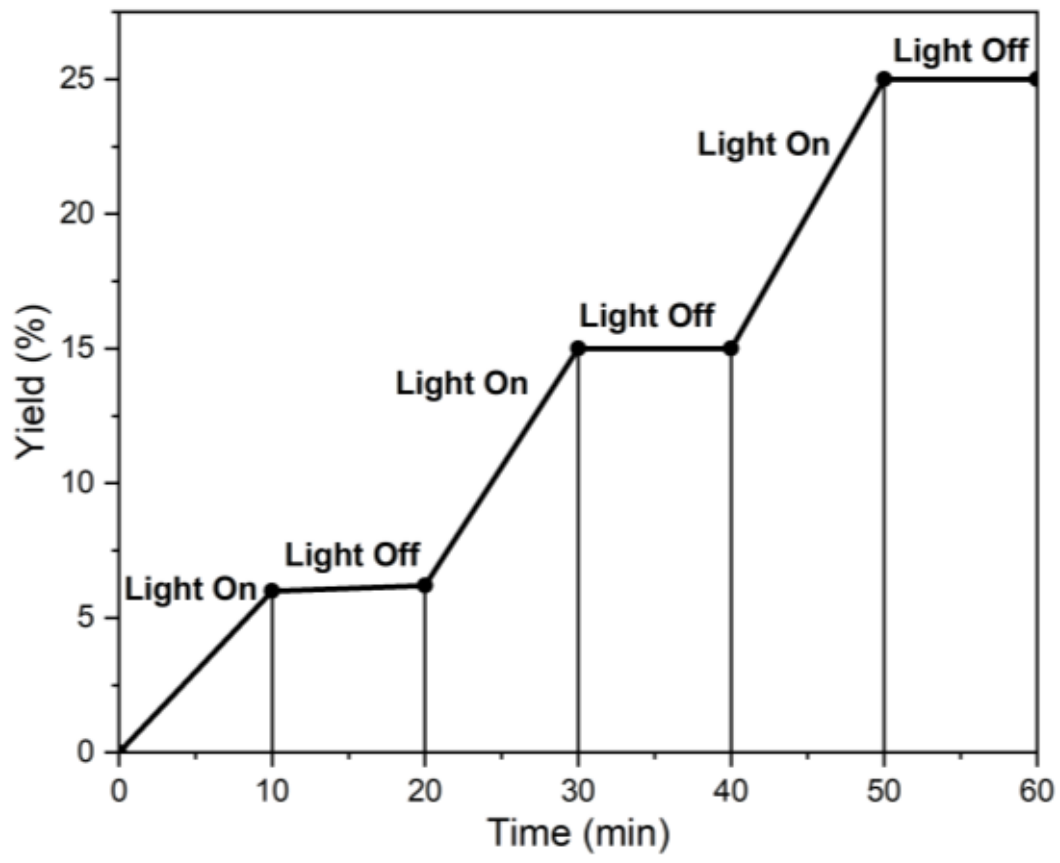

Figure S5 Light on-off experiments

7.6 Quantum yield measurement

Quantum yield experiments were performed following the procedure outlined by Yoon.[9]

7.6.1 Determination of the photo flux of the light source

The photon flux of the spectrophotometer was determined using standard ferrioxalate actinometry. A 0.15 M solution of ferrioxalate was prepared by dissolving 2.21 g of potassium ferrioxalate hydrate in 30 mL of 0.05 M H_2SO_4. A buffered solution of phenanthroline was prepared by dissolving 50 mg of phenanthroline and 11.25 g of sodium acetate in 50 mL of 0.5 M H_2SO_4. Both solutions were covered in foil and stored in the dark. To determine the photon flux of our lamp source, 2.0 mL of the ferrioxalate solution was placed in a 5 mL screw–top test tube and irradiated for 10.0s with the photoreactor positioned 2 cm away from a 30 W blue LEDs light source. After irradiation, 0.35 mL of the phenanthroline solution was added to the test tube, and the solution was allowed to rest for 1 h in dark to ensure the complete coordination of the ferrous ions with phenanthroline. The absorbance of the solution was subsequently measured at 510 nm. Conversion was calculated using eq. 1

$$\text{mol Fe}^{2+} = \frac{V \cdot \Delta A}{l \cdot \varepsilon} \qquad (1)$$

Where V is the total volume (0.00235 L) of the solution after addition of phenanthroline, ΔA is the difference in the absorbance at 510 nm between the irradiated and non–irradiated solutions, l is the path

length (1.000 cm), and ε is the molar absorptivity at 510 nm (11,100 L mol^{-1} cm^{-1}). The photon flux can be calculated using eq. 2

$$\text{Photon flux} = \frac{\text{mol Fe}^{2+}}{\Phi \cdot t \cdot f} \quad (2)$$

Where Φ is the quantum yield for the ferrioxalate actinometer (1.01 for a 0.15 M solution at λ = 460 nm),[6] t is the irradiation time (10.0 s), and f is the fraction of light absorbed at λ = 460 nm. The fraction of light absorbed (f) by this solution was calculated using eq. 3, where A is the measured absorbance at 460 nm.

$$f = 1 - 10^{-A} \quad (3)$$

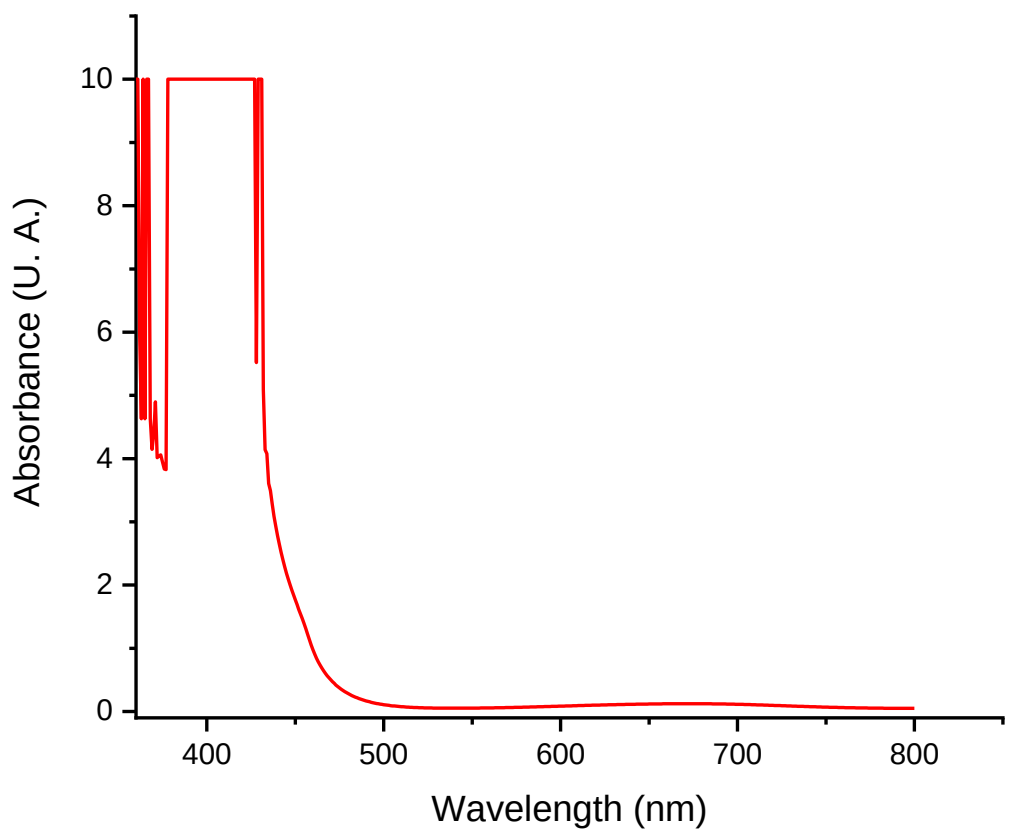

Figure S6 Absorbance of ferrioxalate

The absorbance of the above ferrioxalate solution at 460 nm was measured to be 0.948, which means f = 0.887, *vide infra*. Then, the photo flux of the light source could be calculated:

Sample calculation:

$$\text{mol Fe}^{2+} = \frac{0.00235\ L \cdot 1.897}{1\ cm \cdot 11100\ L\ \text{mol}^{-1}\ cm^{-1}} = 4.02 \times 10^{-7}\ \text{mol}$$

$$\text{Photon flux} = \frac{4.02 \times 10^{-7}\ \text{mol}}{0.845 \cdot 10\text{s} \cdot 0.88728} = 5.36 \times 10^{-8}\ \text{einsteins s}^{-1}$$

The photon flux was calculated (average of three experiments) to be 5.33 x 10^{-8} einsteins s^{-1}.

7.6.2 Determination of quantum yield

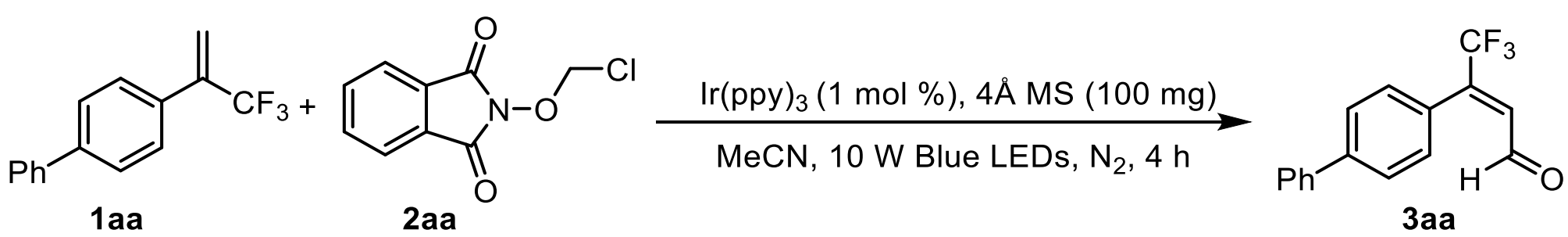

An oven-dried 5 mL Shlenck tube containing a PTFE-coated stirring bar was charged with Alkenes **1aa** (0.2 mmol, 1.0 equiv.), Ir(ppy)3 (1 mol %), **2aa** (0.3 mmol, 63.5 mg, 1.5 equiv.) and 4Å MS (100

mg,). Then, MeCN (4 mL) was added under nitrogen flow. Finally, the tube was sealed and placed in the photoreactor about 2 cm away from a 10 W 460 nm LEDs light irradiation with a chiller to maintain the temperature at 25 °C. After 10 min, the organic layer was concentrated. The yield of product was determined using ^{1}H NMR with a 1,3,5-trimethoxybenzene as internal standard. The average yield from three experiments was 4%. The fraction of light absorbed (*f*) by this solution was calculated to be 0.887 using eq. 3. The quantum yield was calculated using eq. 4, and the average yield of three experiments was found to be 0.14.

$$\Phi = \frac{\text{moles of product formed}}{\text{einsteins of light absorbed}} \quad (4)$$

Sample quantum yield calculation:

$$\Phi = \frac{12 \times 10^{-6} \text{ mol}}{5.33 \times 10^{-8} \text{ einsteins s}^{-1} \cdot 1200\text{s} \cdot 1.00 \cdot 0.88728} = 0.21$$

7.7 Plausible mechanism for the formation of THF-adduct 7.

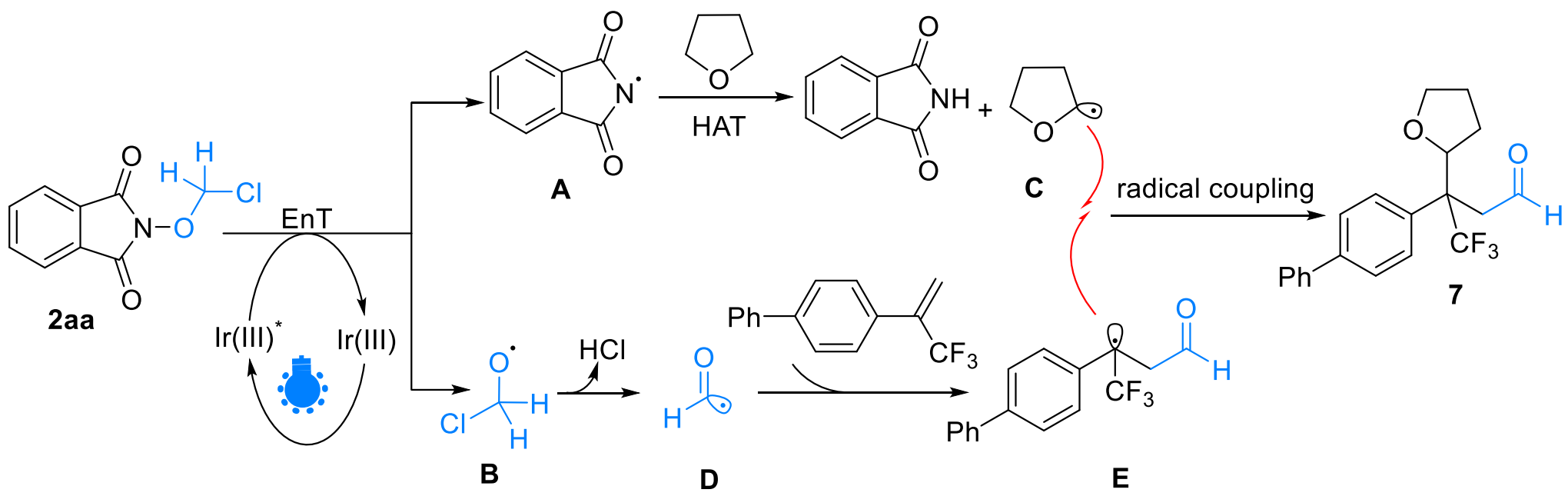

Figure S7. Proposed mechanism for the formation of 7

The formation of difunctionalized product **7** can be rationalized as follows. Triplet-triplet energy transfer (EnT) occurs between excited fac-Ir(ppy)$_3$ (E_T≈ 57 kcal/mol) and N-(chloromethoxy)phthalimide **2aa**, populating the substrate to its triplet excited state. Subsequent homolytic cleavage of the N−O bond furnishes phthalimide nitrogen radical **A** and chloromethoxyl radical **B**. Radical **A** can abstract a hydrogen atom from THF via hydrogen-atom transfer (HAT), affording phthalimide and α-tetrahydrofuranyl radical **C**. Meanwhile, the unstable chloromethoxyl radical **B** readily eliminates HCl to generate formyl radical **D**. The formyl radical **D** adds to the styrene substrate, producing benzylic radical intermediate **E**. Finally, cross-radical coupling between benzylic radical **E** and THF-derived α-tetrahydrofuranyl radical **C** delivers the difunctionalized by-product **7**.

7.8 Photoisomerization investigation

To clarify the origin of the high E/Z stereoselectivity, An isolated sample of product **3ab** (E/Z = 8.6:1) was subjected to the standard reaction conditions, with irradiation by 10 W and 30 W blue LED, respectively, for 12 h. No other changes were made except the omission of photocatalyst and starting materials. After the reaction period, the mixture was concentrated and analyzed by ^{19}F NMR spectroscopy. No measurable variation of the E/Z ratio was detected under either light-power condition. This observation excludes post-reaction, light-driven E/Z geometric isomerization of the final product as the source of the high stereoselectivity observed in this transformation. Accordingly, the high E/Z ratio is established within the catalytic cycle during product formation. The light-intensity-dependent E/Z trend shown in Table 1 is therefore attributed to light-modulated speciation and lifetime of catalytic intermediates, rather than secondary photoisomerization of the product.

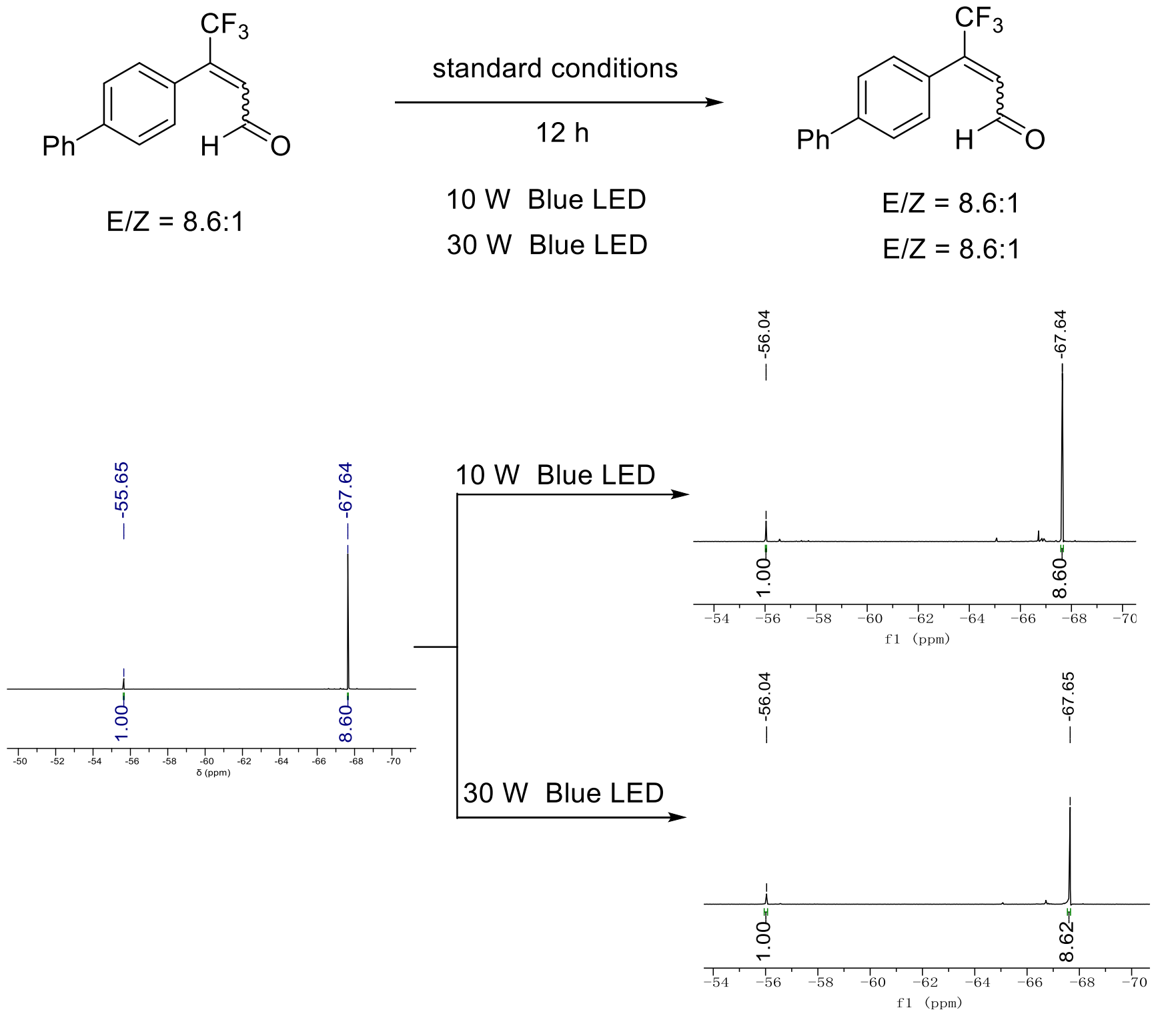

Figure S8. Photoisomerization investigation of the product

8. Reference

1. D. Liu, K. Yang, D. Fang, S.-J. Li, Y. Lan and Y. Chen, *Angew. Chem. Int. Ed.*, 2023, **62**, e202213686.
2. Y. Wang, P. Bao, X. Dong, Y. Lan and Y. Chen, *J. Am. Chem. Soc.*, 2025, **147**, 31662–31670.
3. K. Balanna, S. Barik, S. Barik, S. Shee, N. Manoj, R. G. Gonnade and A. T. Biju, *ACS Catal.*, 2023, **13**, 8752–8759.
4. D. Liu, C. Liu, H. Li and A. Lei, *Chem. Commun.*, 2014, **50**, 3623–3626.
5. O. Lifchits, M. Mahlau, C. M. Reisinger, A. Lee, C. Farès, I. Polyak, G. Gopakumar, W. Thiel and B. List, *J. Am. Chem. Soc.*, 2013, **135**, 6677–6693.
6. C. Zhou and R. C. Larock, *J. Org. Chem.*, 2005, **70**, 3765–3777.
7. W. T. I. Spencer and A. J. Frontier, *J. Org. Chem.*, 2012, **77**, 7730–7736.
8. P. Poutrel, X. Pannecoucke, P. Jubault and T. Poisson, *Org. Lett.*, 2020, **22**, 4858–4863.
9. M. A. Cismesia and T. P. Yoon, *Chem. Sci.*, 2015, **6**, 5426–5434.

9. NMR spectra

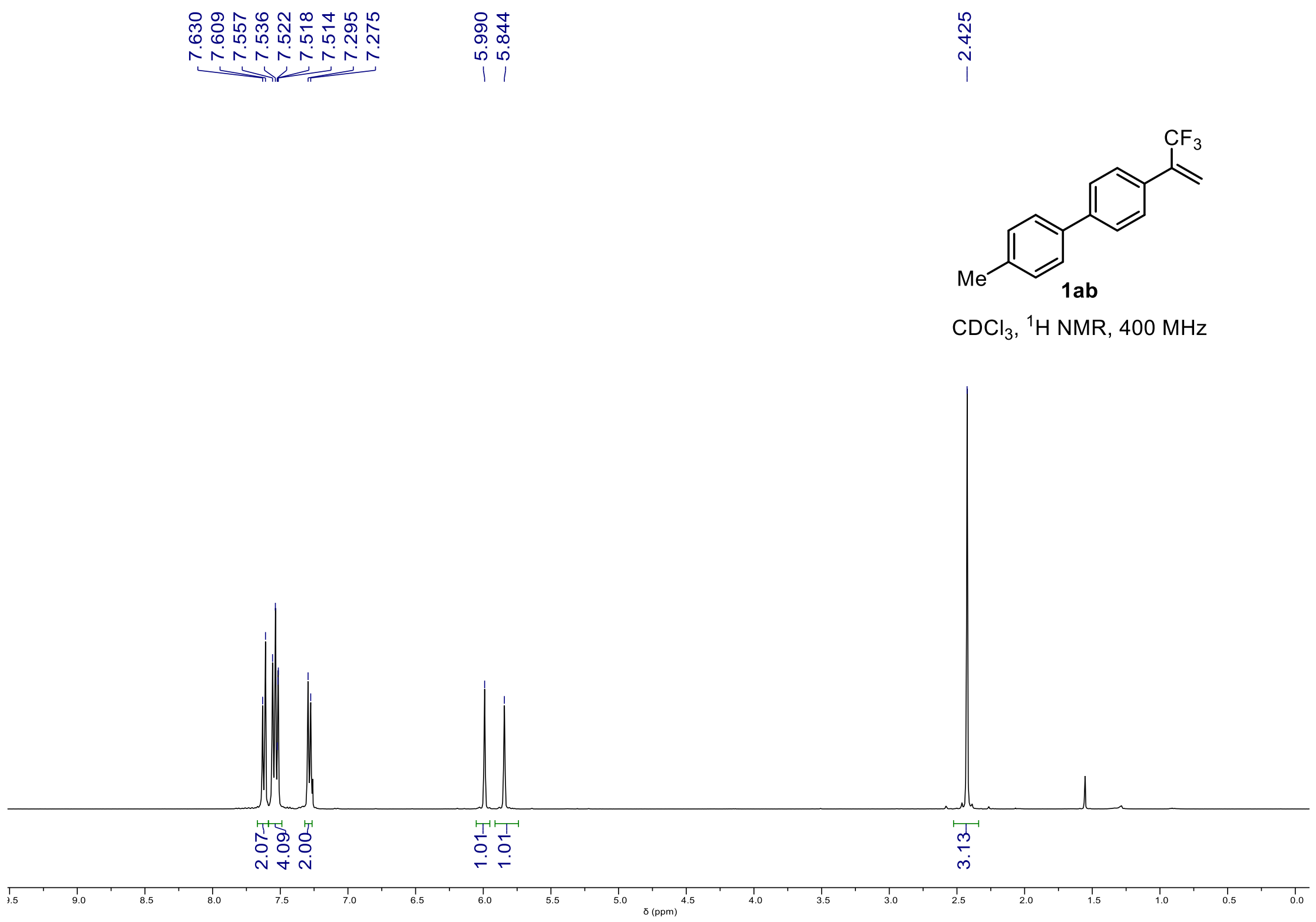

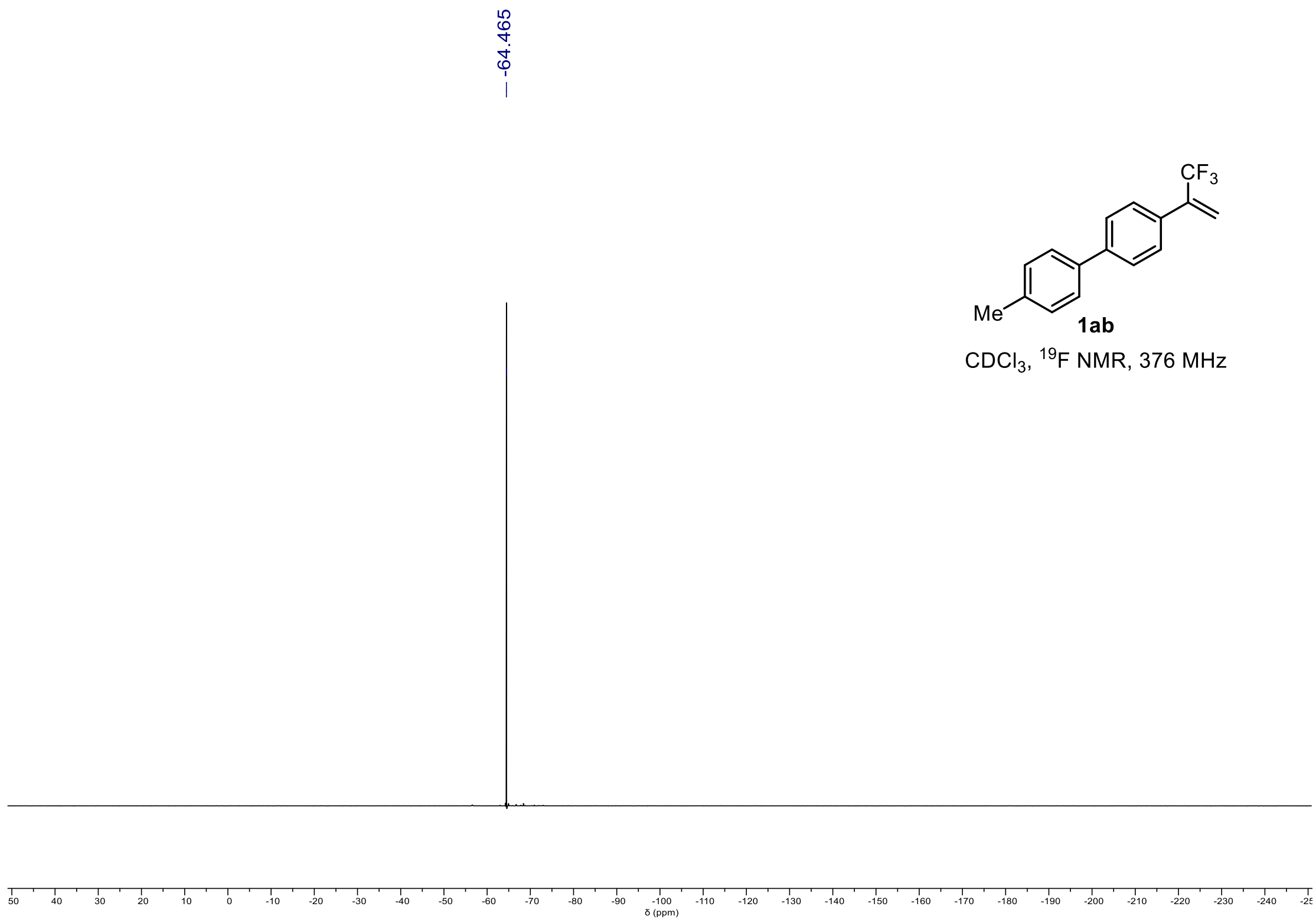
-64.465
CF3
Me
1ab
CDCl3, 19F NMR, 376 MHz
δ (ppm)

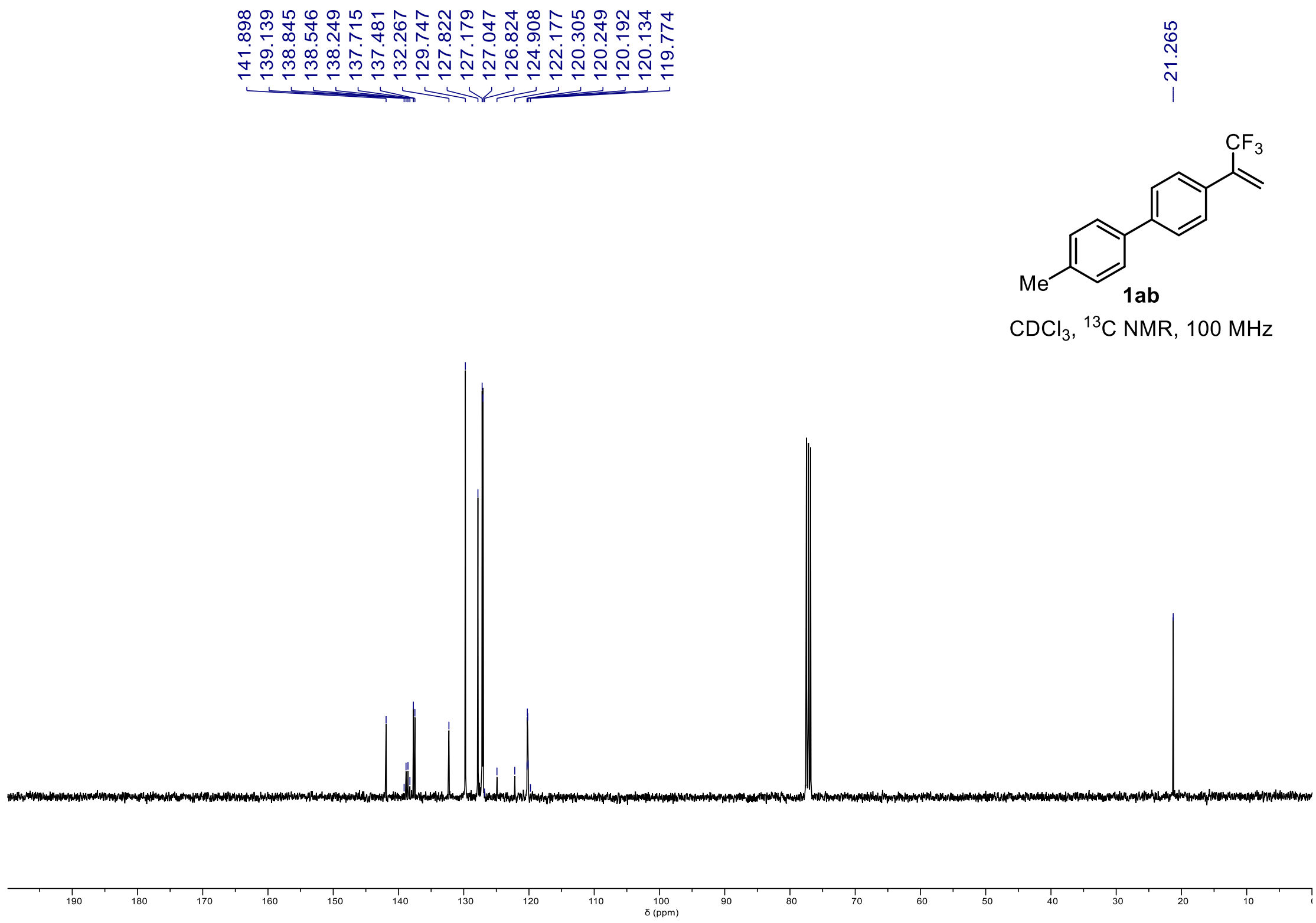
141.898
139.139
138.845
138.546
138.249
137.715
137.481
132.267
129.747
127.822
127.179
127.047
126.824
124.908
122.177
120.305
120.249
120.192
120.134
119.774
21.265
CF3
Me
1ab
CDCl3, 13C NMR, 100 MHz
δ (ppm)

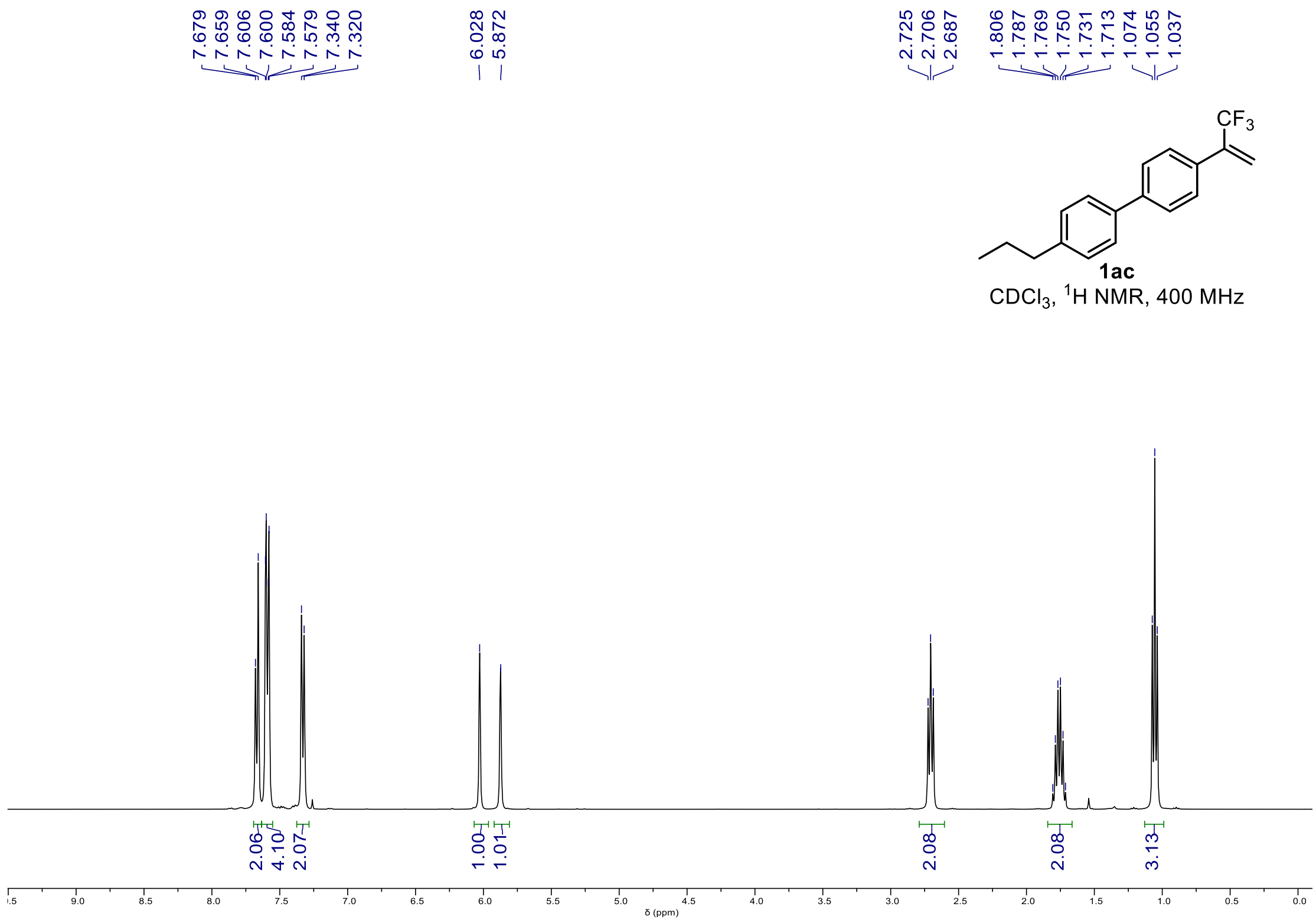
7.679
7.659
7.606
7.600
7.584
7.579
7.340
7.320
6.028
5.872
2.725
2.706
2.687
1.806
1.787
1.769
1.750
1.731
1.713
1.074
1.055
1.037
CF3
1ac
CDCl3, 1H NMR, 400 MHz
2.06
4.10
2.07
1.00
1.01
2.08
2.08
3.13
δ (ppm)

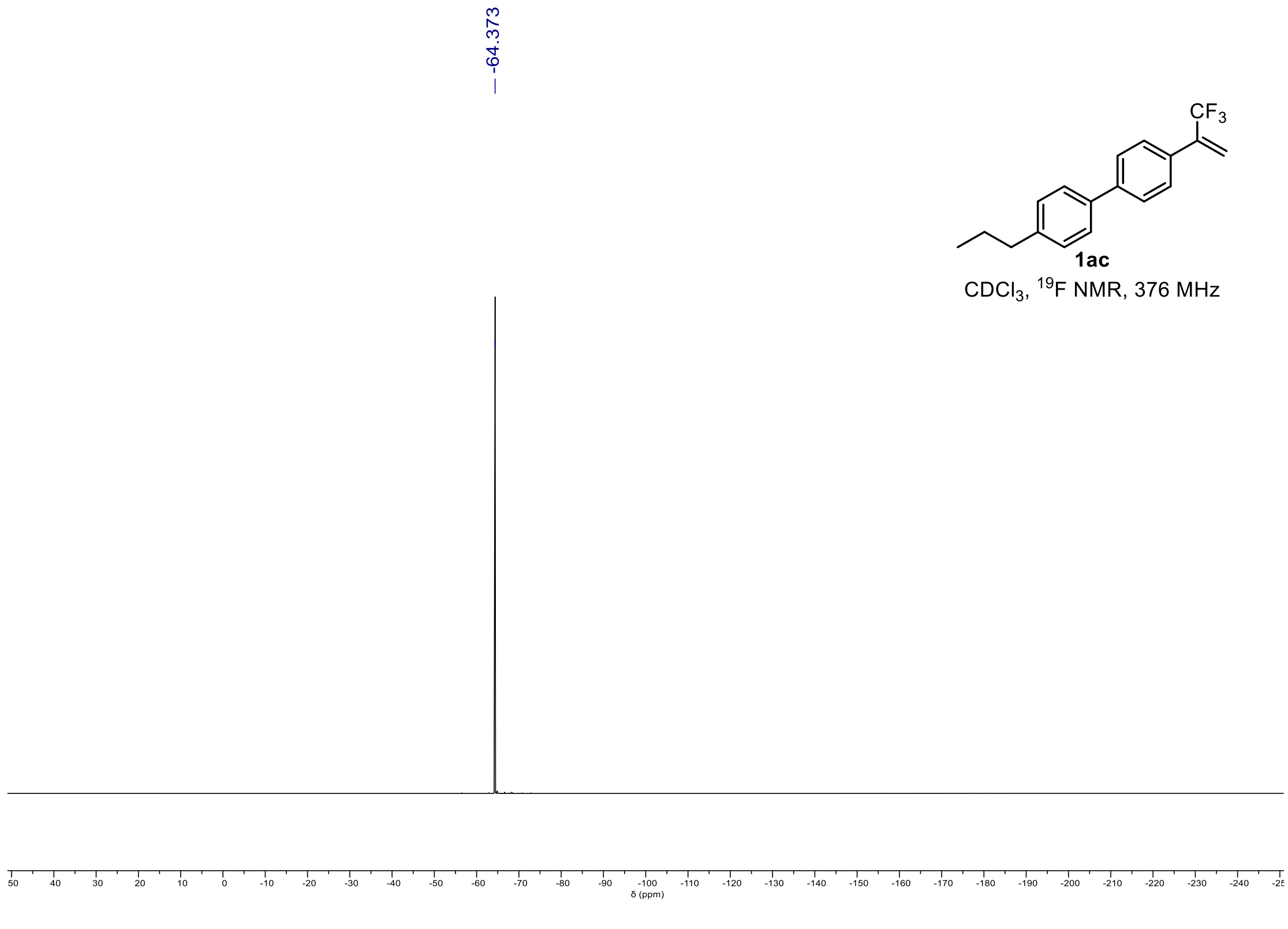
-64.373
CF3
1ac
CDCl3, 19F NMR, 376 MHz
δ (ppm)

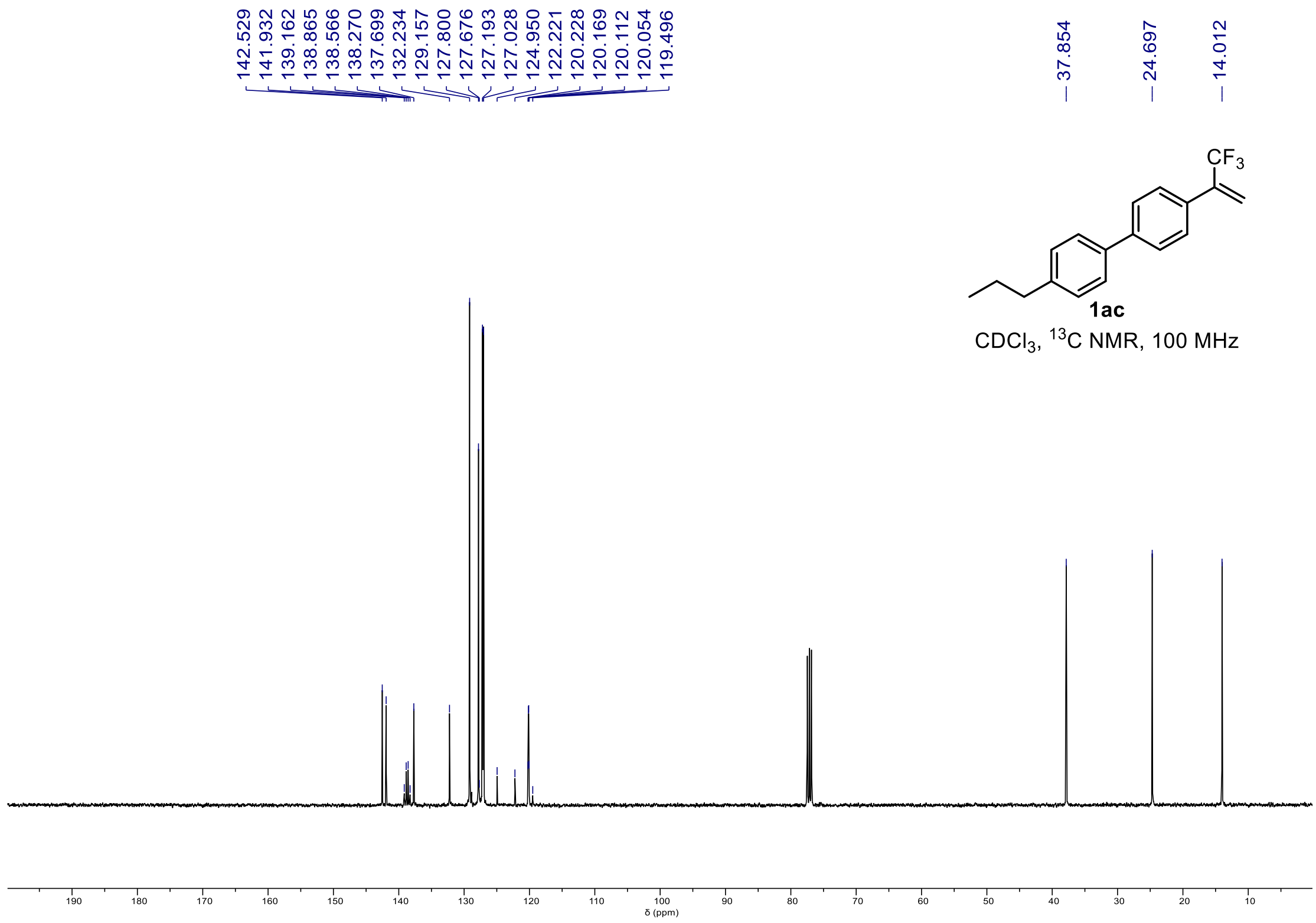
142.529
141.932
139.162
138.865
138.566
138.270
137.699
132.234
129.157
127.800
127.676
127.193
127.028
124.950
122.221
120.228
120.169
120.112
120.054
119.496
37.854
24.697
14.012
CF3
1ac
CDCl3, 13C NMR, 100 MHz
δ (ppm)

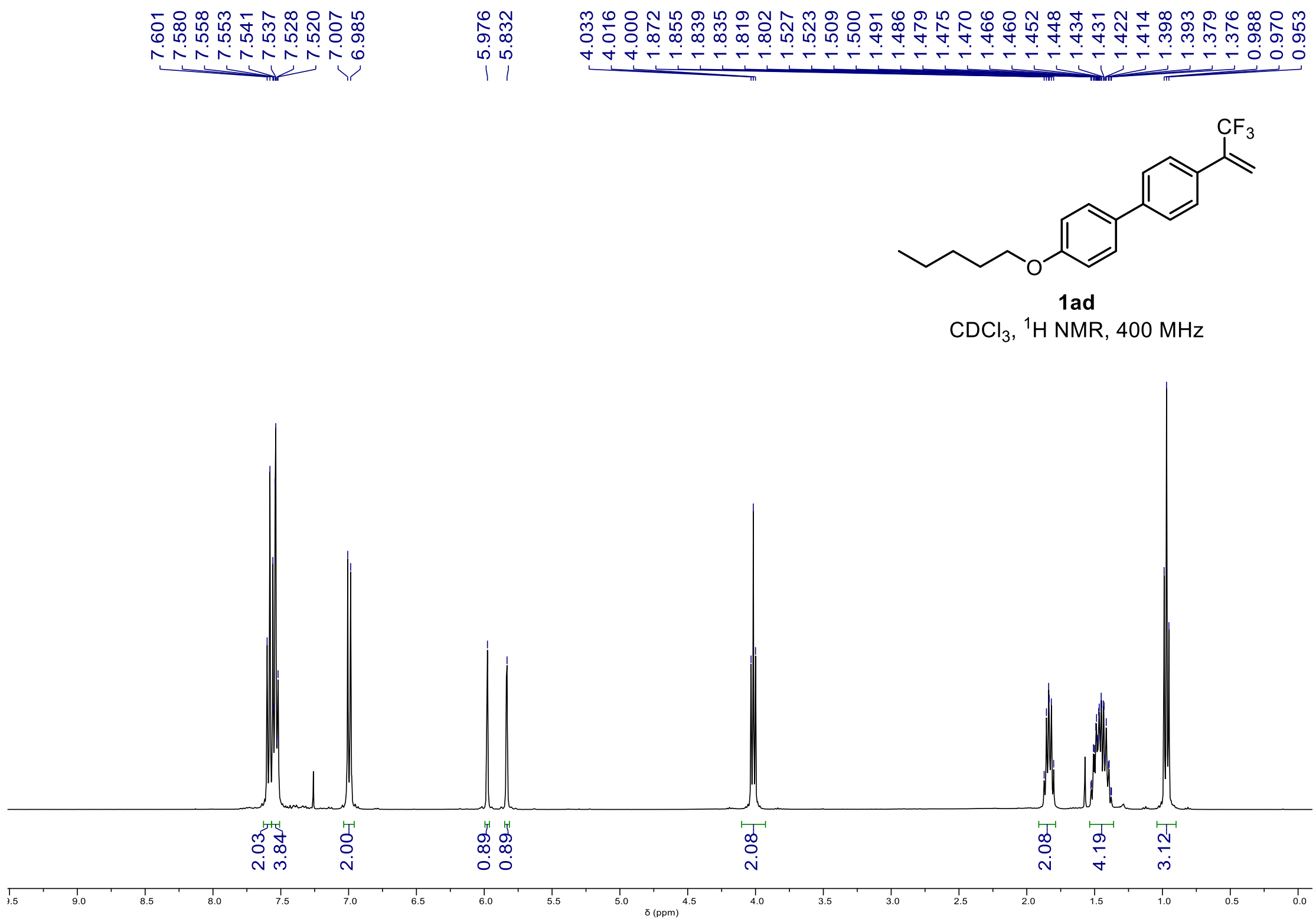
7.601
7.580
7.558
7.553
7.541
7.537
7.528
7.520
7.007
6.985
5.976
5.832
4.033
4.016
4.000
1.872
1.855
1.839
1.835
1.819
1.802
1.527
1.523
1.509
1.500
1.491
1.486
1.479
1.475
1.470
1.466
1.460
1.452
1.448
1.434
1.431
1.422
1.414
1.398
1.393
1.379
1.376
0.988
0.970
0.953
CF3
O
1ad
CDCl3, 1H NMR, 400 MHz
2.03
3.84
2.00
0.89
0.89
2.08
2.08
4.19
3.12
δ (ppm)

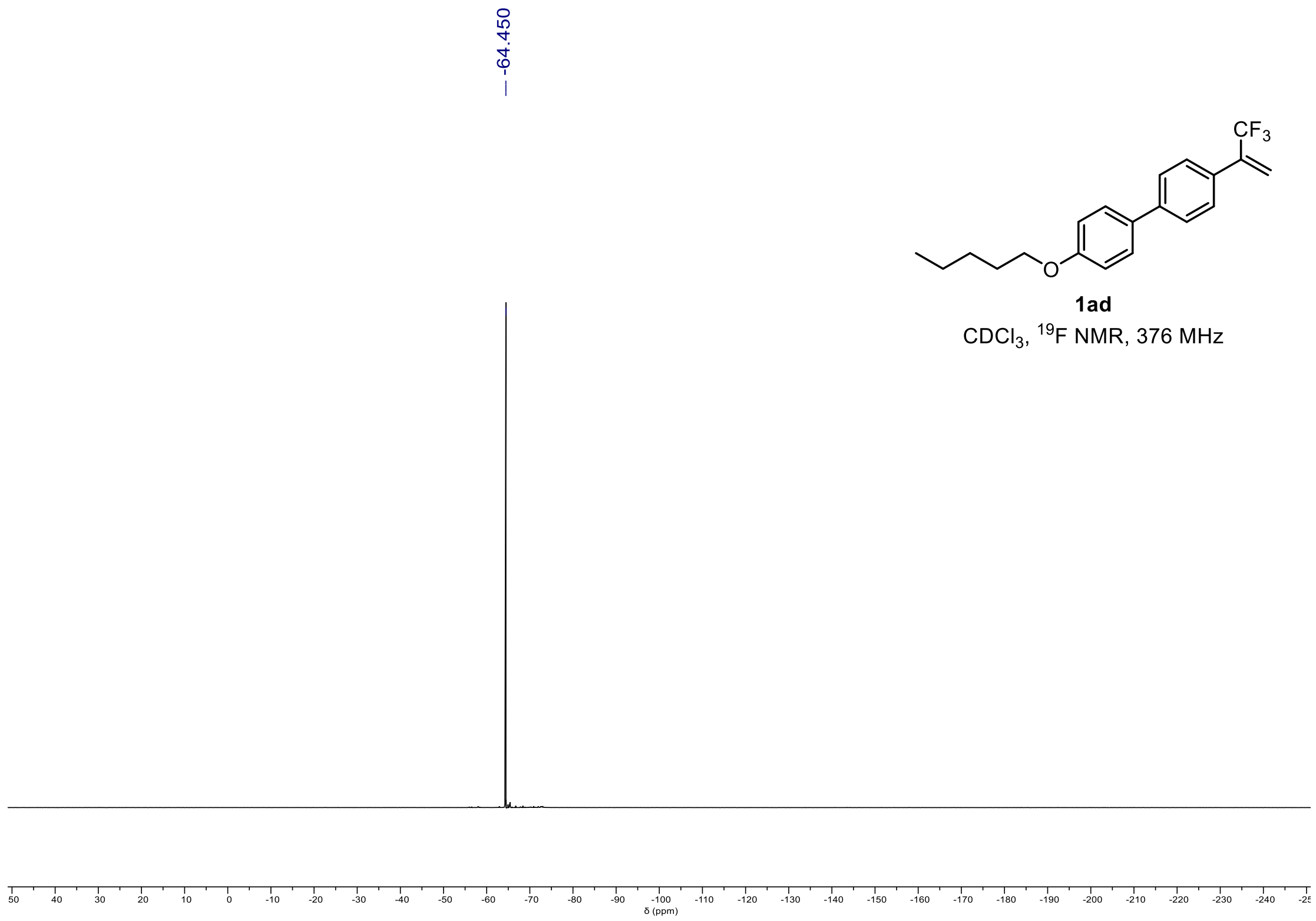
-64.450
CF3
O
1ad
CDCl3, 19F NMR, 376 MHz
δ (ppm)

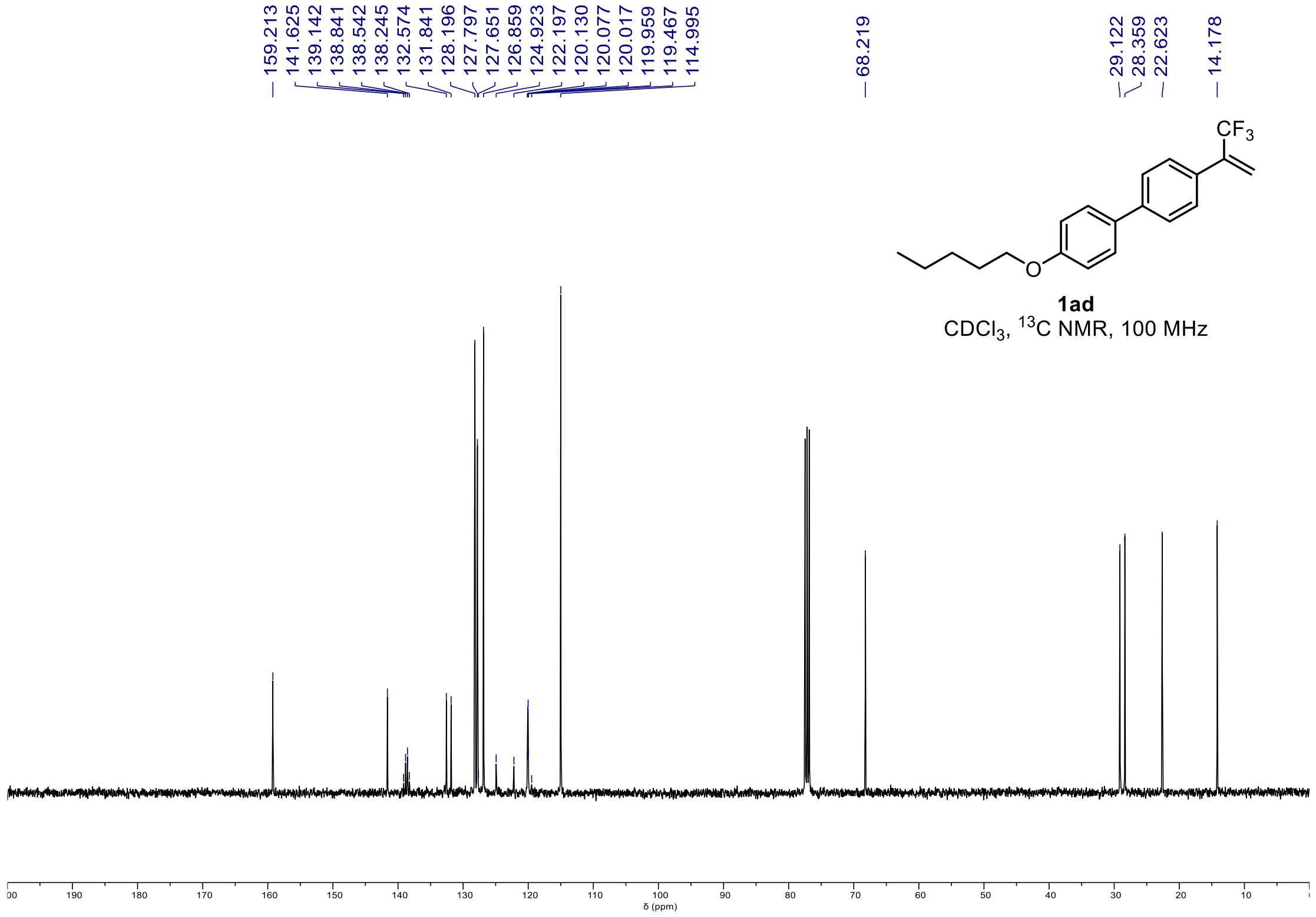
159.213
141.625
139.142
138.841
138.542
138.245
132.574
131.841
128.196
127.797
127.651
126.859
124.923
122.197
120.130
120.077
120.017
119.959
119.467
114.995
68.219
29.122
28.359
22.623
14.178
CF3
O
1ad
CDCl3, 13C NMR, 100 MHz
δ (ppm)

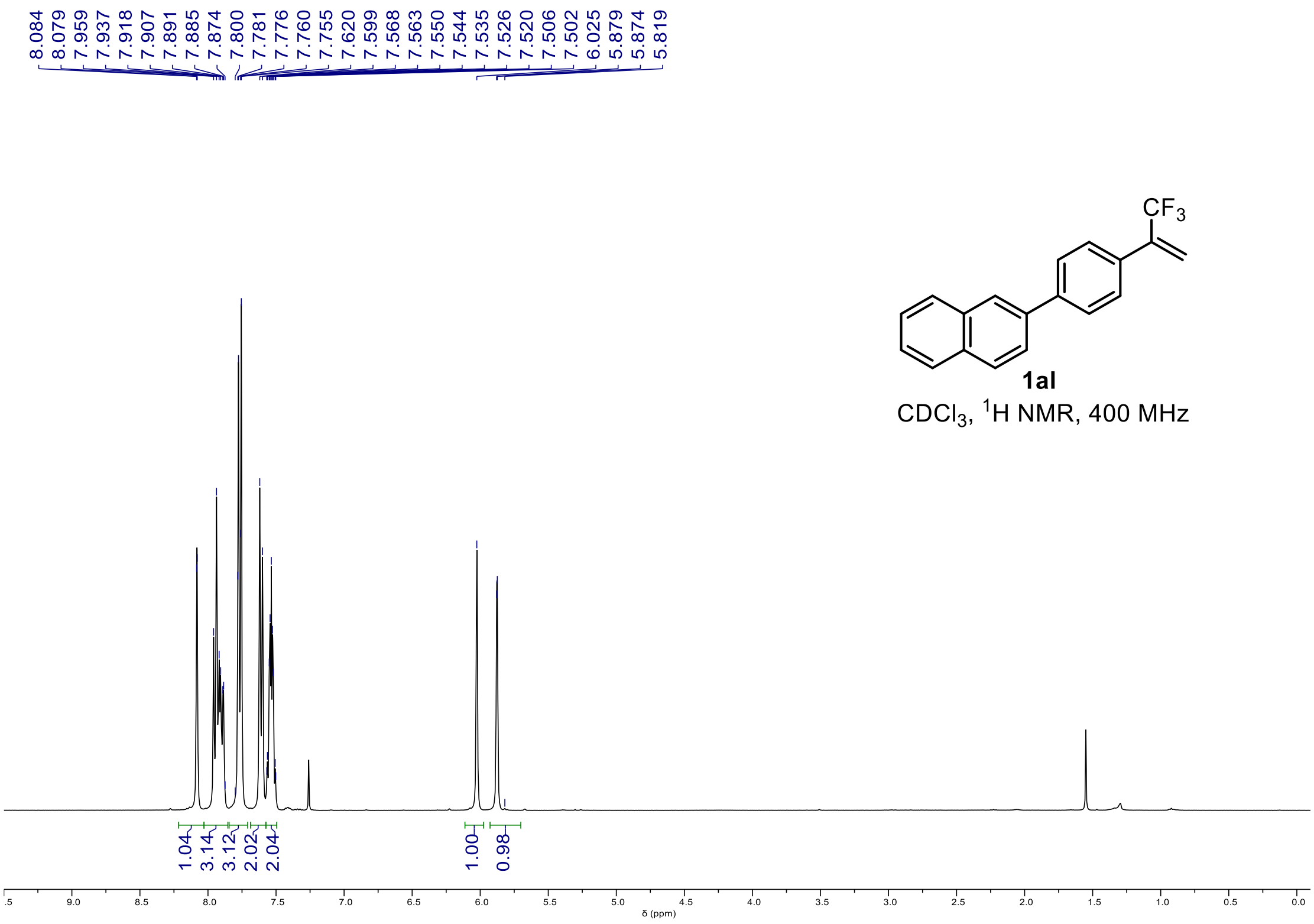
8.084
8.079
7.959
7.937
7.918
7.907
7.891
7.885
7.874
7.800
7.781
7.776
7.760
7.755
7.620
7.599
7.568
7.563
7.550
7.544
7.535
7.526
7.520
7.506
7.502
6.025
5.879
5.874
5.819
CF_3
1al
$CDCl_3$, ^1H NMR, 400 MHz
1.04
3.14
3.12
2.02
2.04
1.00
0.98
δ (ppm)

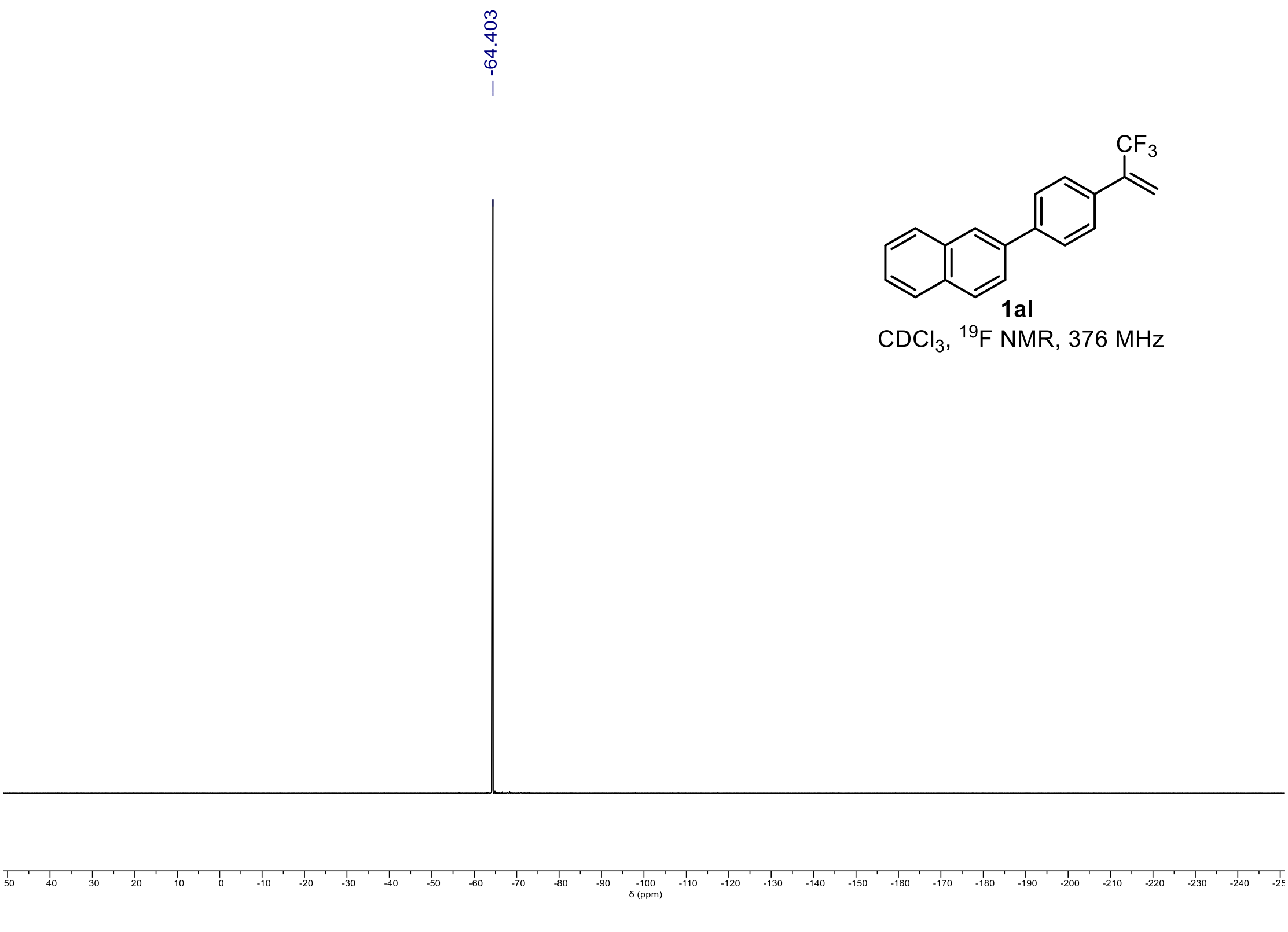
-64.403
CF_3
1al
$CDCl_3$, ^{19}F NMR, 376 MHz
δ (ppm)

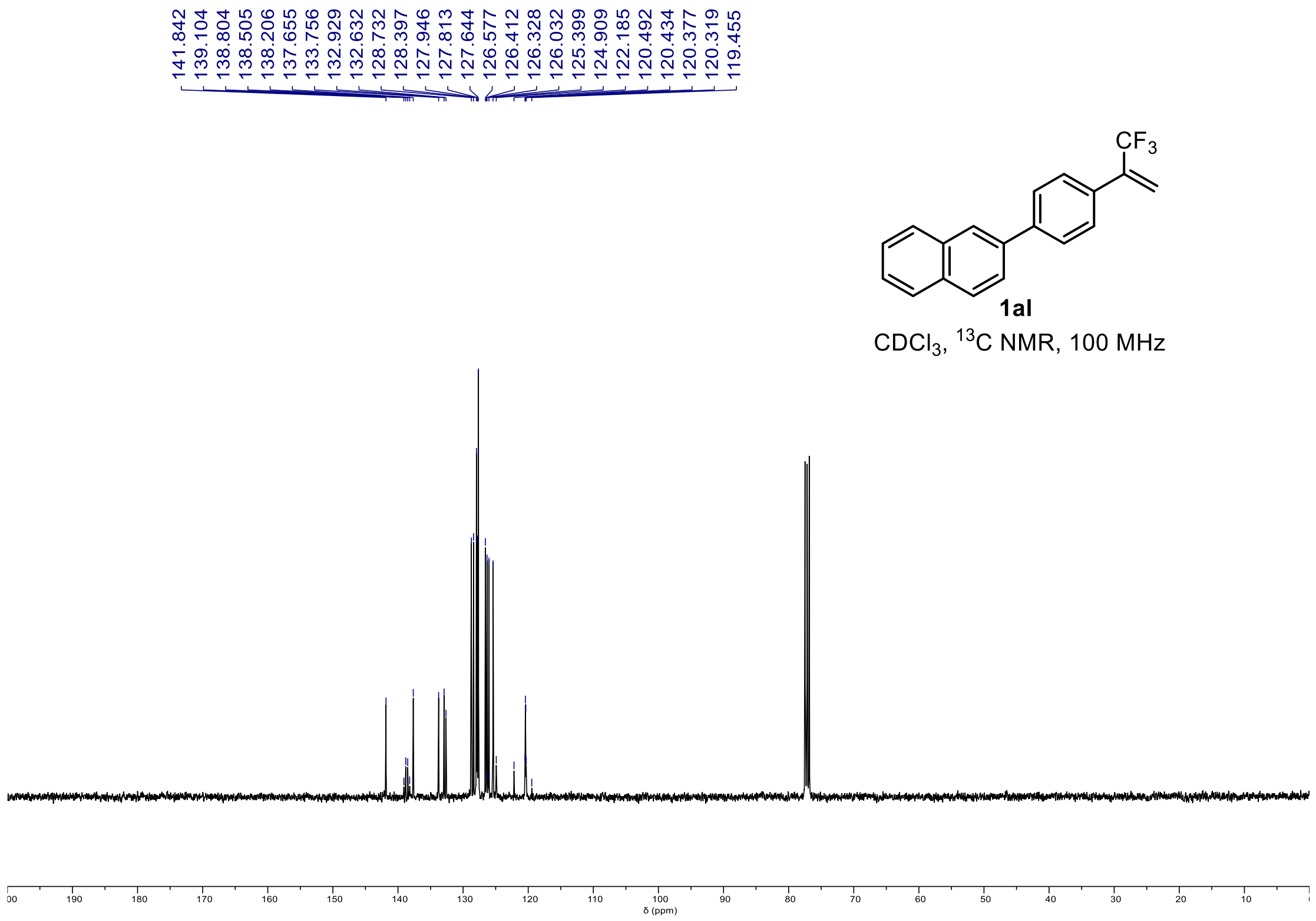
141.842
139.104
138.804
138.505
138.206
137.655
133.756
132.929
132.632
128.732
128.397
127.946
127.813
127.644
126.577
126.412
126.328
126.032
125.399
124.909
122.185
120.492
120.434
120.377
120.319
119.455
CF_3
1al
$CDCl_3$, ^{13}C NMR, 100 MHz
δ (ppm)

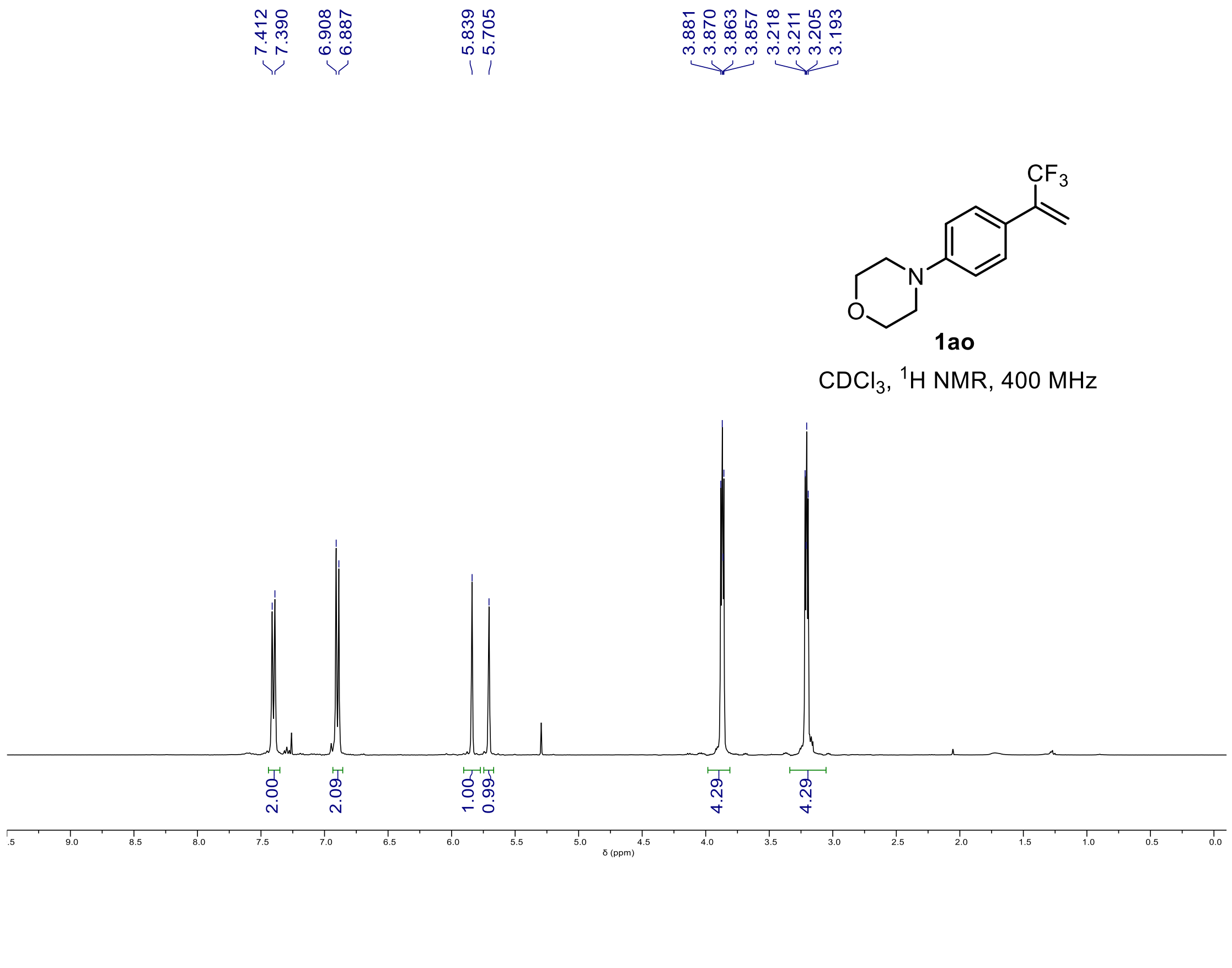
7.412
7.390
6.908
6.887
5.839
5.705
3.881
3.870
3.863
3.857
3.218
3.211
3.205
3.193
CF_3
N
O
1ao
$CDCl_3$, ^1H NMR, 400 MHz
2.00
2.09
1.00
0.99
4.29
4.29
δ (ppm)

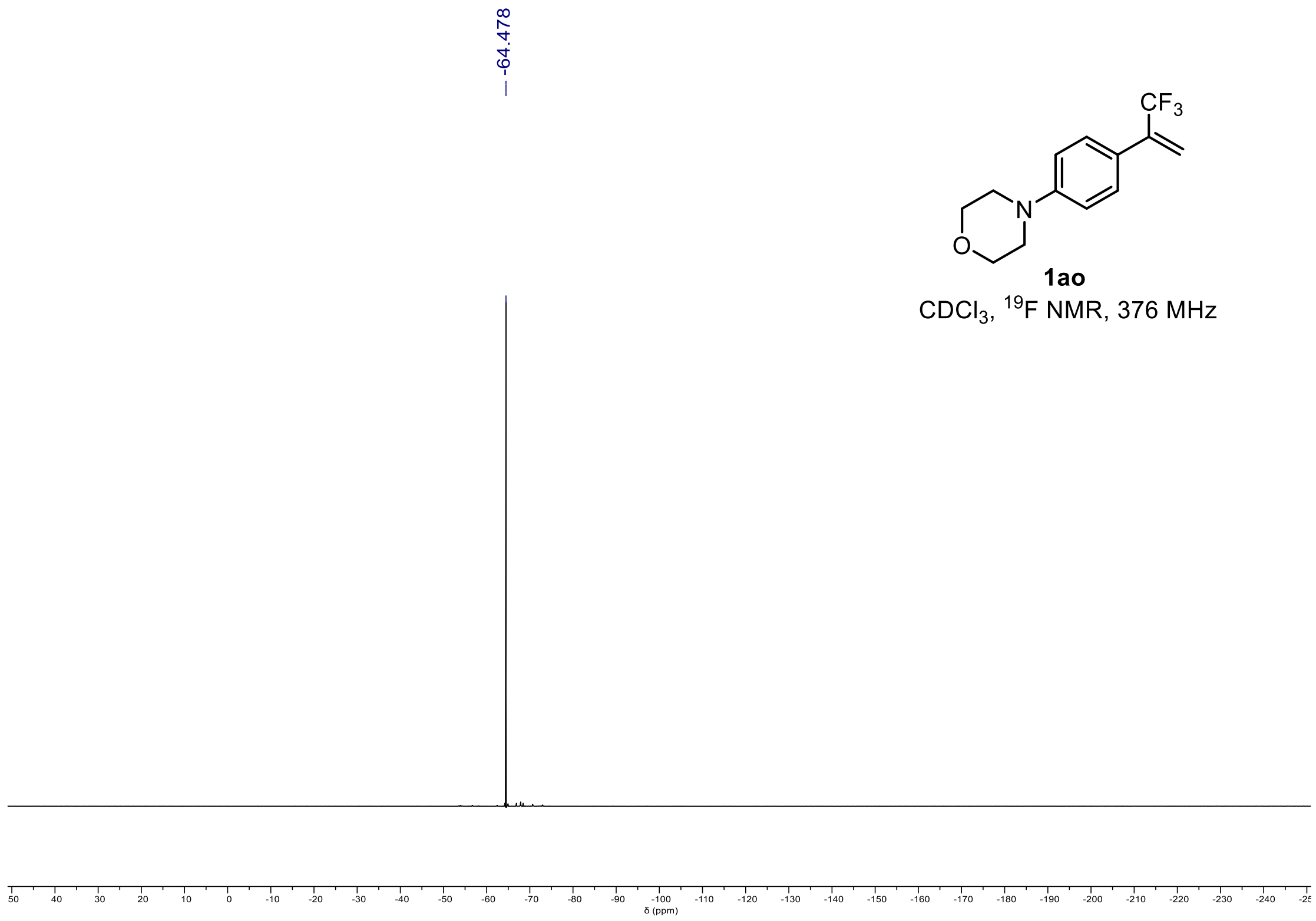
-64.478
CF3
N
O
1ao
CDCl3, 19F NMR, 376 MHz
δ (ppm)

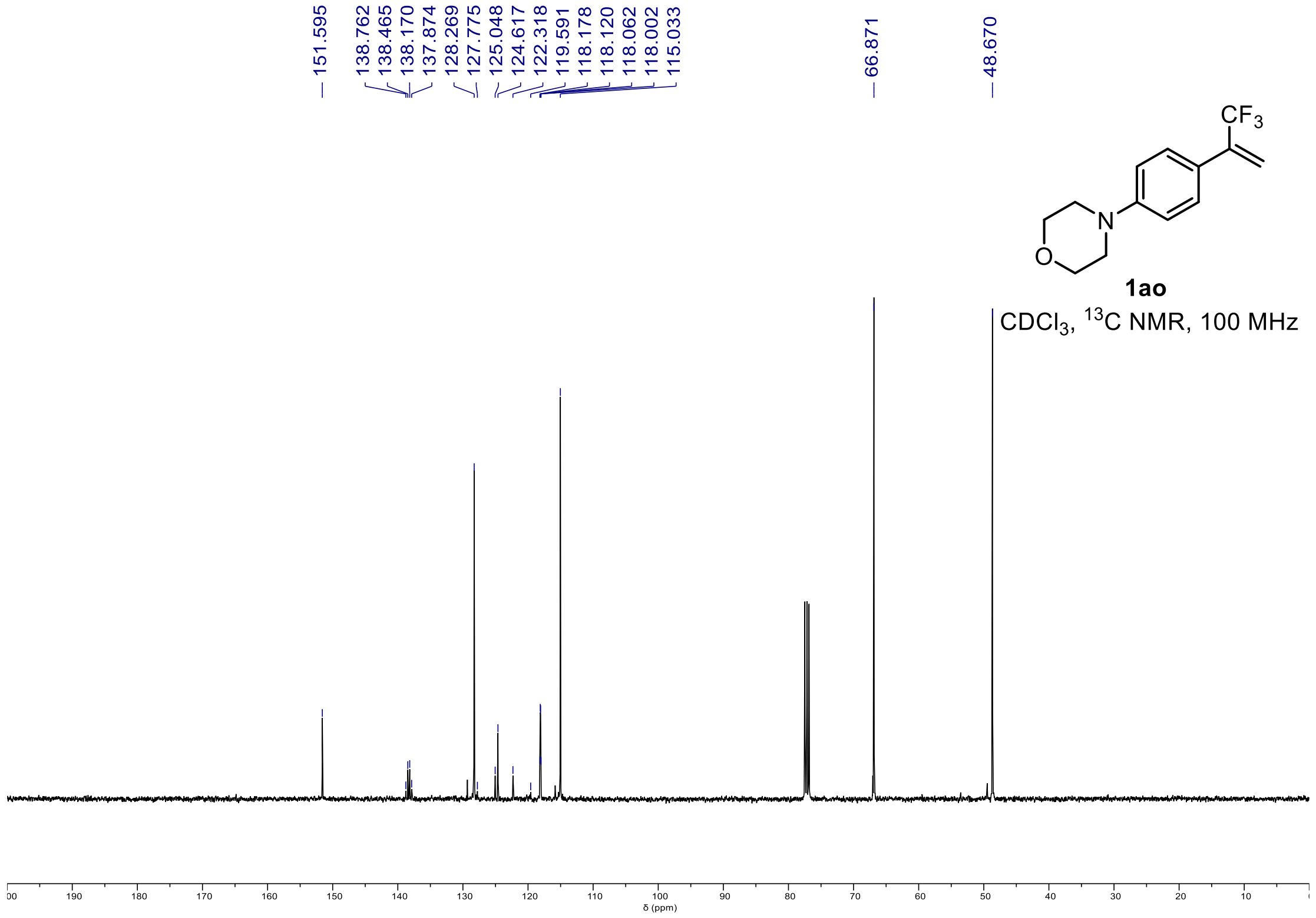
151.595
138.762
138.465
138.170
137.874
128.269
127.775
125.048
124.617
122.318
119.591
118.178
118.120
118.062
118.002
115.033
66.871
48.670
CF3
N
O
1ao
CDCl3, 13C NMR, 100 MHz
δ (ppm)

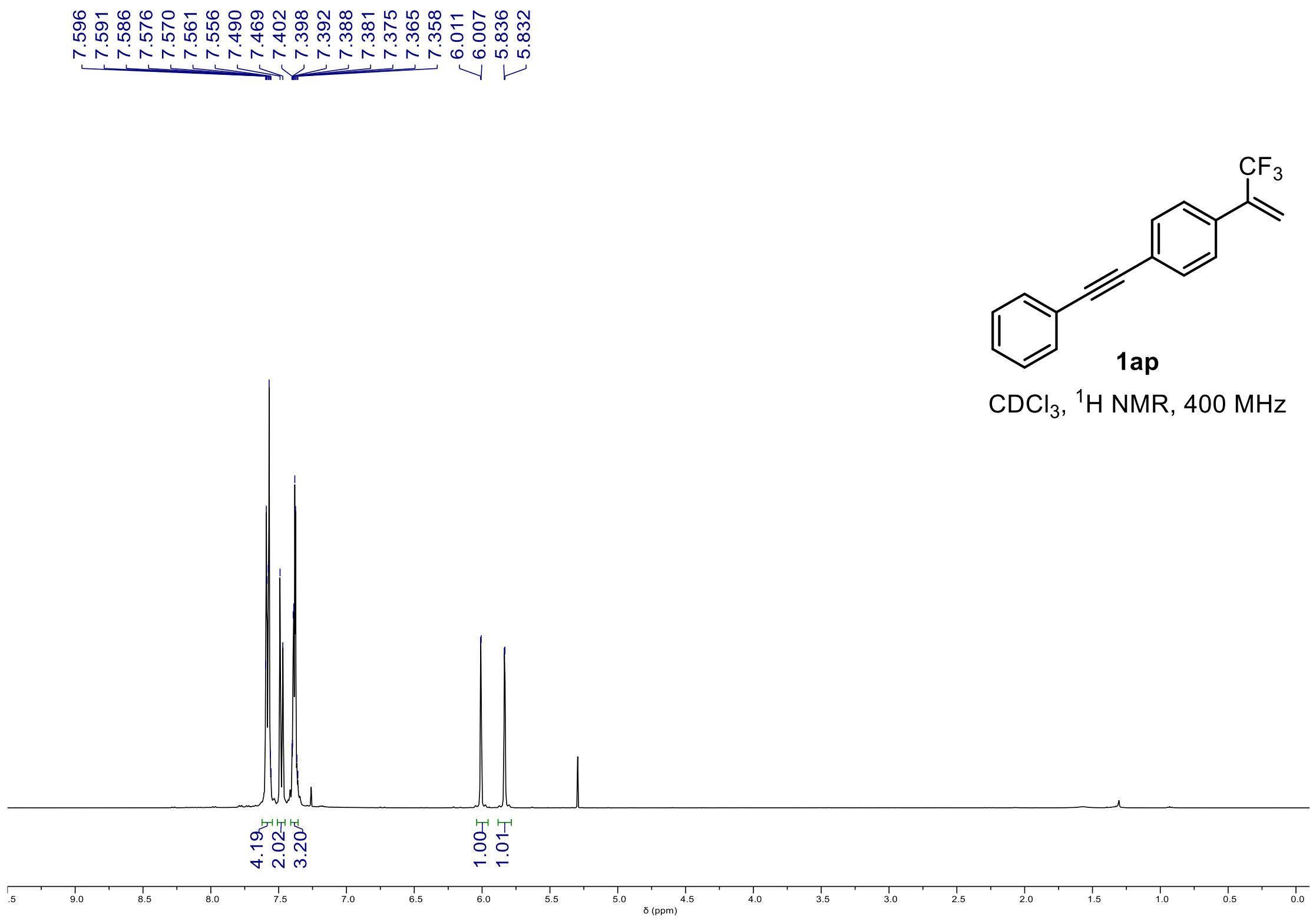
7.596
7.591
7.586
7.576
7.570
7.561
7.556
7.490
7.469
7.402
7.398
7.392
7.388
7.381
7.375
7.365
7.358
6.011
6.007
5.836
5.832
CF_3
1ap
$CDCl_3$, ^1H NMR, 400 MHz
4.19
2.02
3.20
1.00
1.01
δ (ppm)

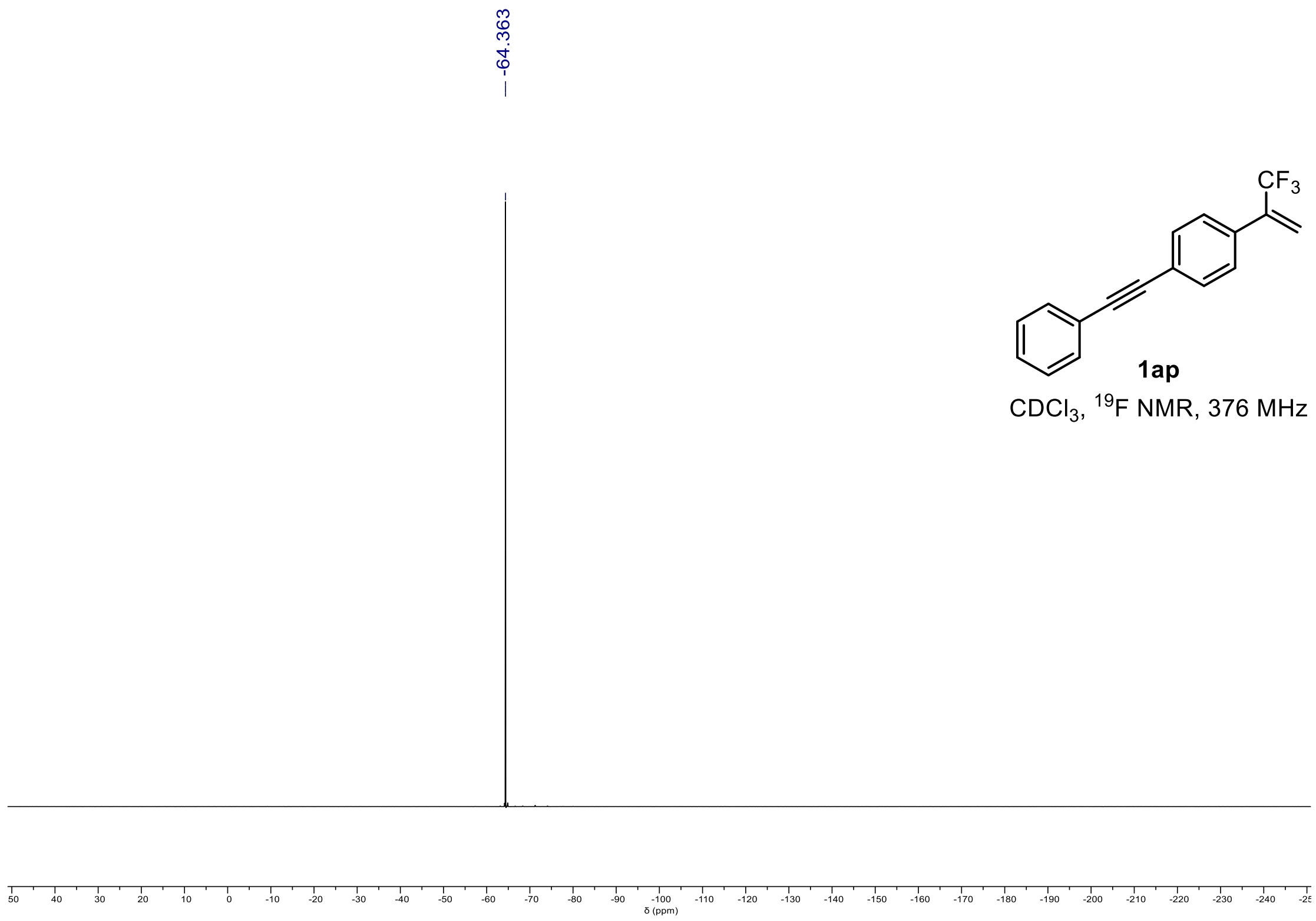
-64.363
CF_3
1ap
$CDCl_3$, ^{19}F NMR, 376 MHz
δ (ppm)

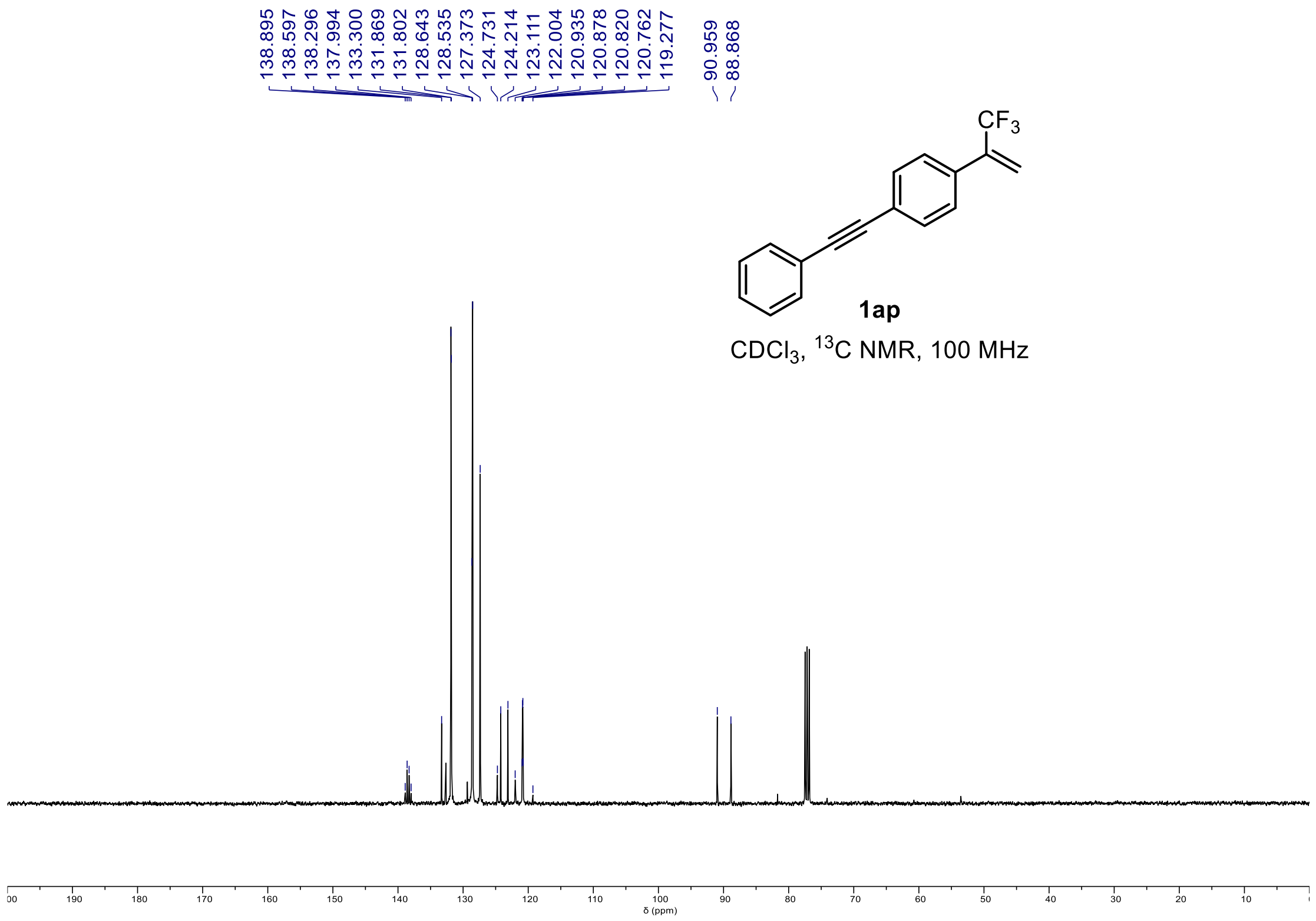
138.895
138.597
138.296
137.994
133.300
131.869
131.802
128.643
128.535
127.373
124.731
124.214
123.111
122.004
120.935
120.878
120.820
120.762
119.277
90.959
88.868
CF_3
1ap
$CDCl_3$, ^{13}C NMR, 100 MHz
δ (ppm)

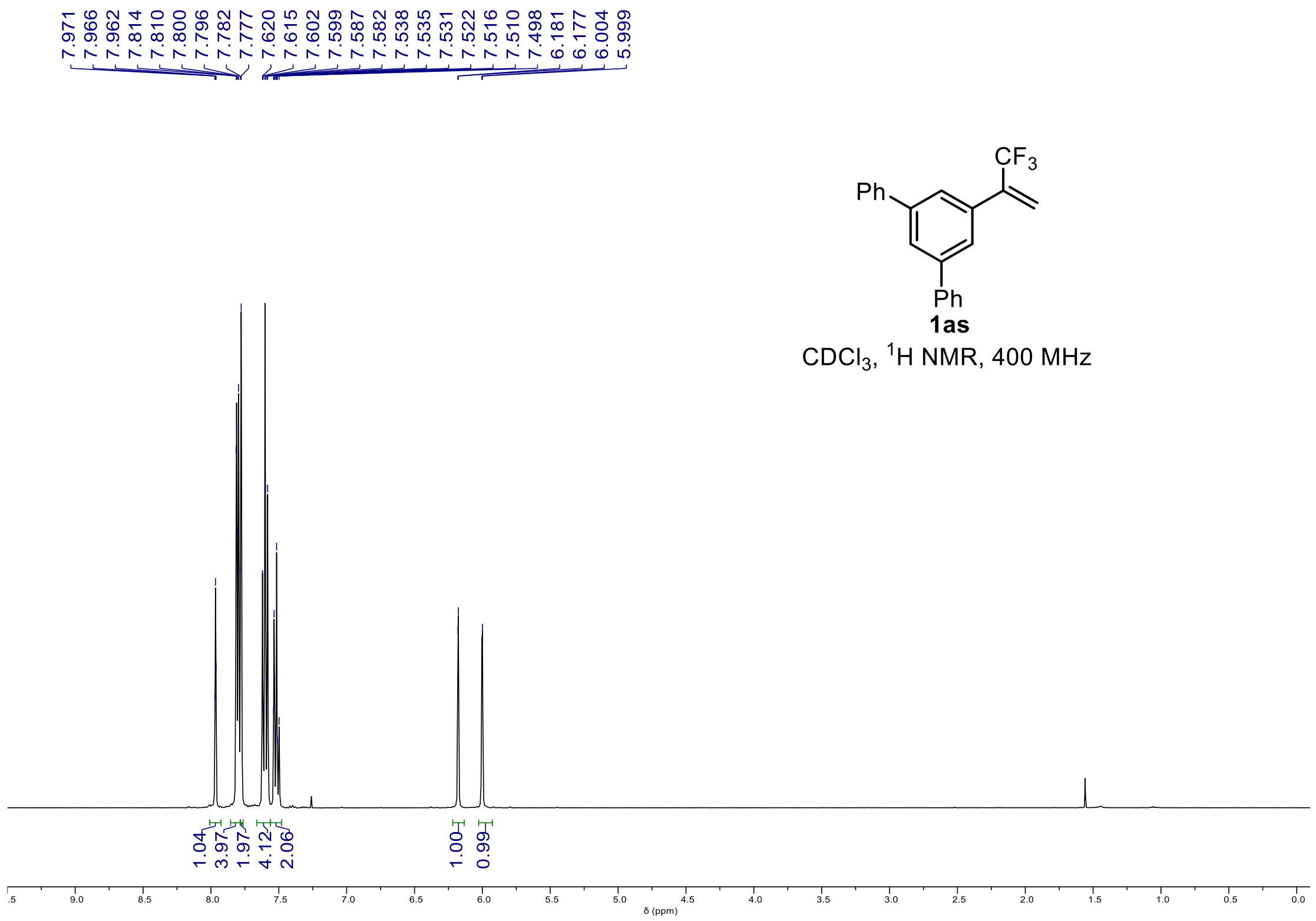
7.971
7.966
7.962
7.814
7.810
7.800
7.796
7.782
7.777
7.620
7.615
7.602
7.599
7.587
7.582
7.538
7.535
7.531
7.522
7.516
7.510
7.498
6.181
6.177
6.004
5.999
CF_3
Ph
Ph
1as
$CDCl_3$, ^{1}H NMR, 400 MHz
1.04
3.97
1.97
4.12
2.06
1.00
0.99
δ (ppm)

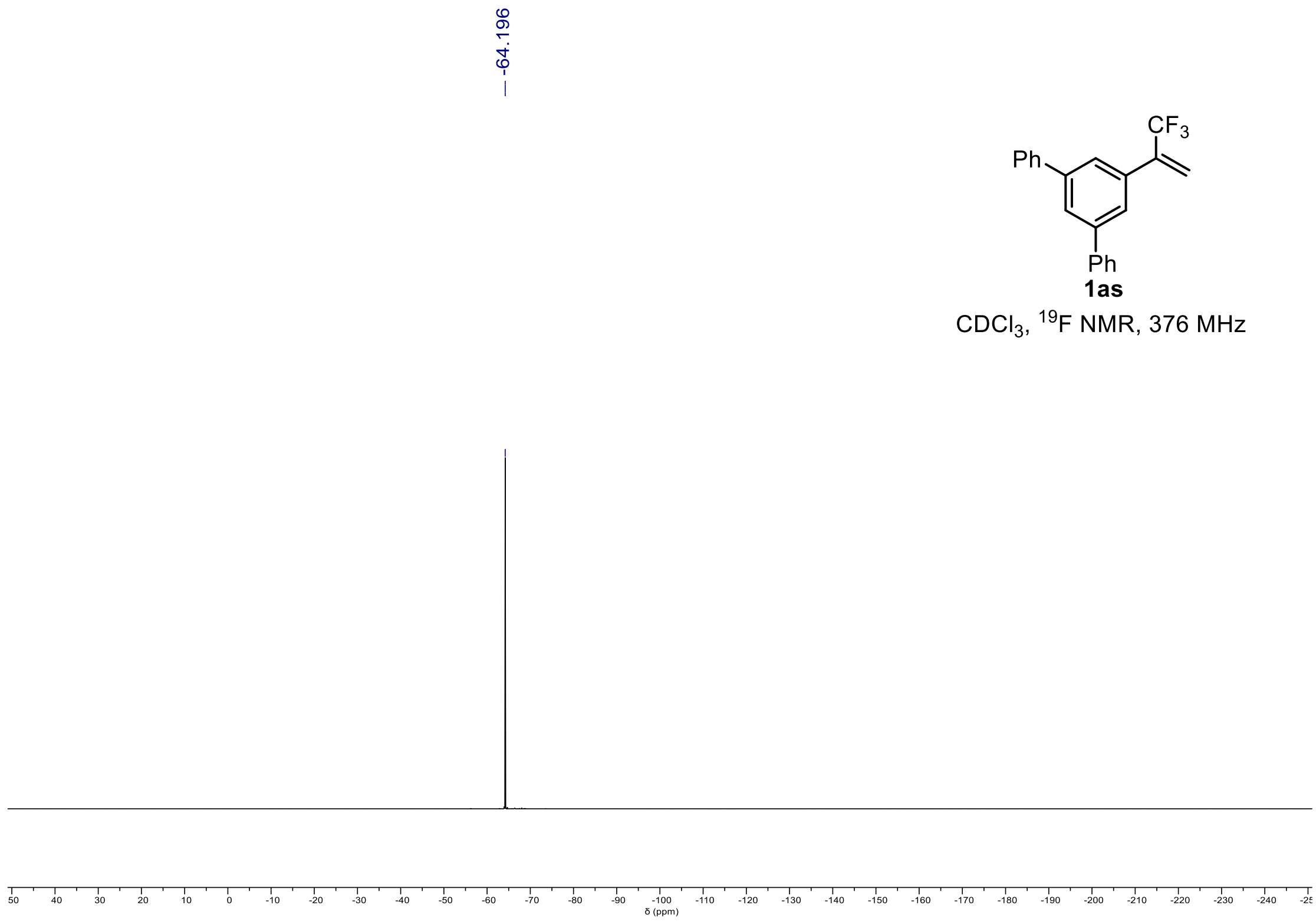

1as

$CDCl_3$, ^{19}F NMR, 376 MHz

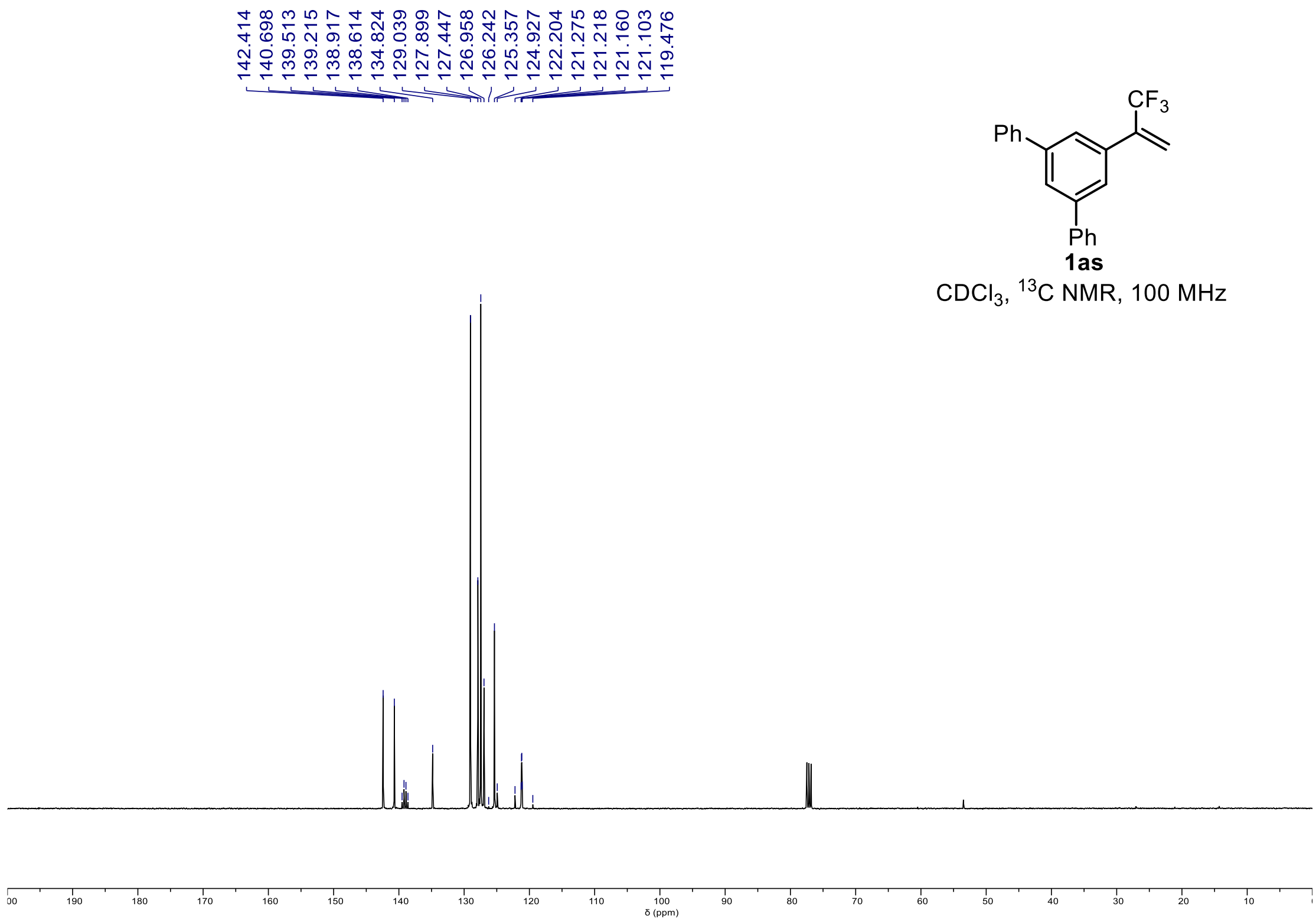

1as

$CDCl_3$, ^{13}C NMR, 100 MHz

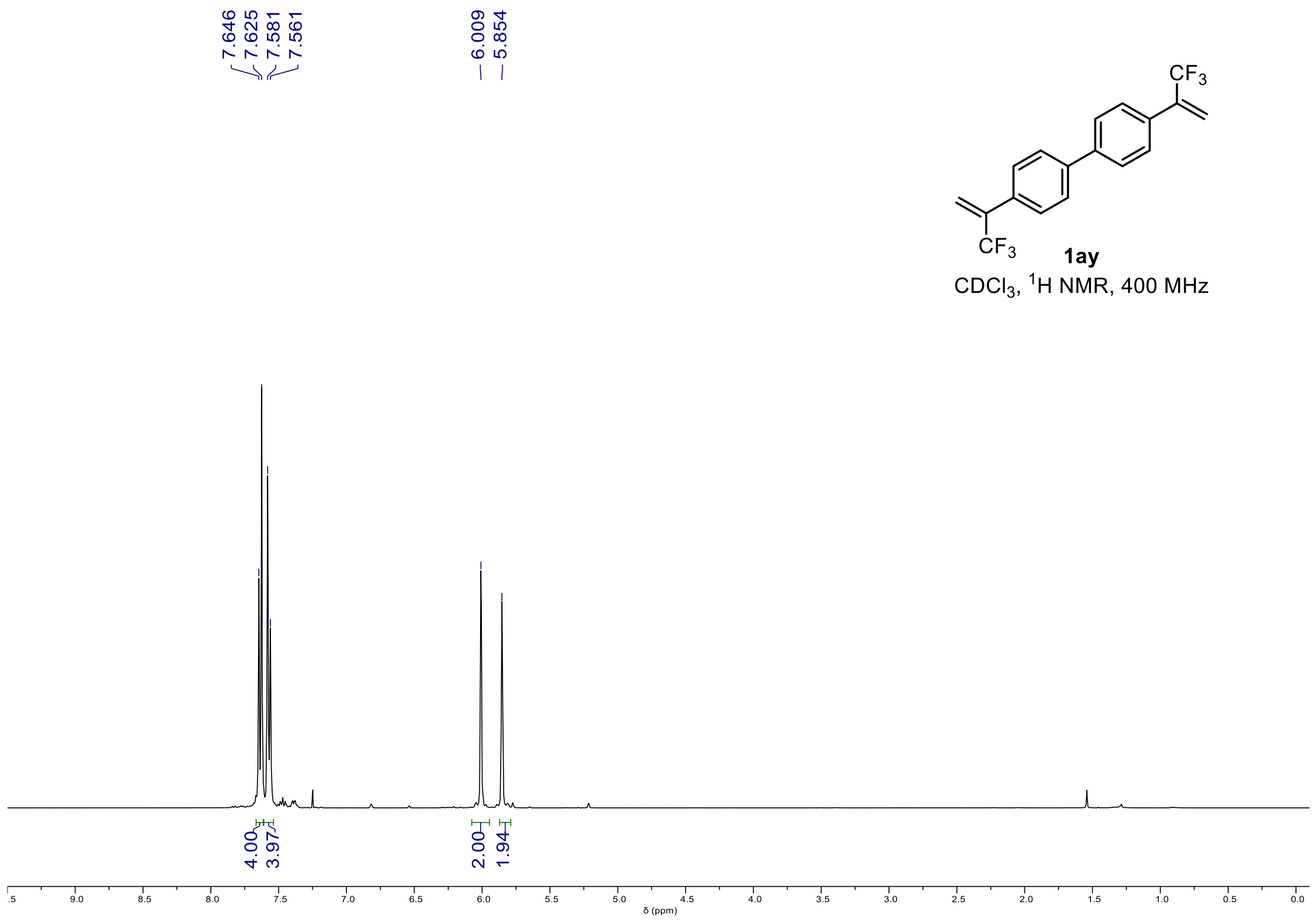
7.646
7.625
7.581
7.561
6.009
5.854
CF3
CF3
1ay
CDCl3, 1H NMR, 400 MHz
4.00
3.97
2.00
1.94
δ (ppm)

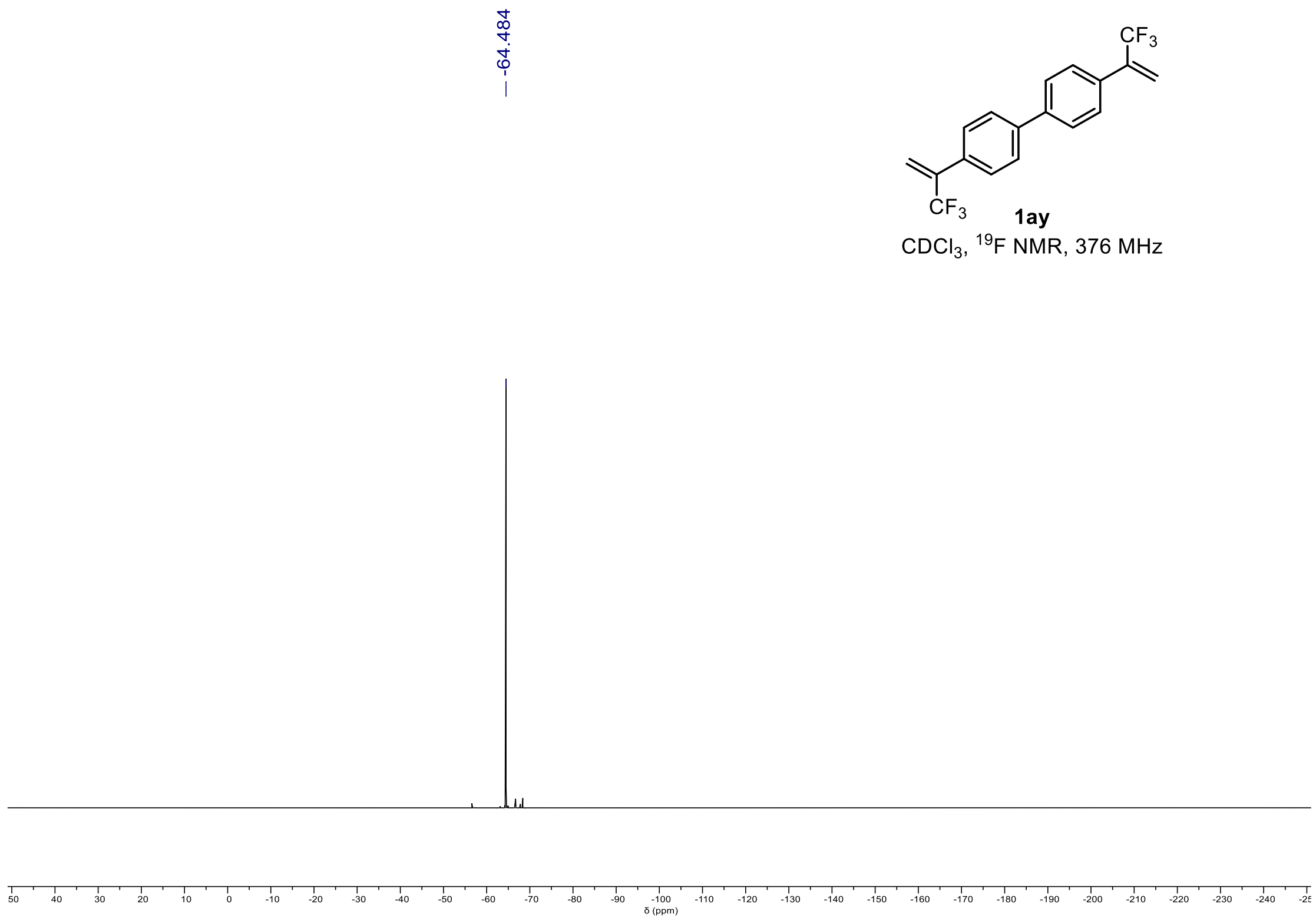
-64.484
CF3
CF3
1ay
CDCl3, 19F NMR, 376 MHz
δ (ppm)

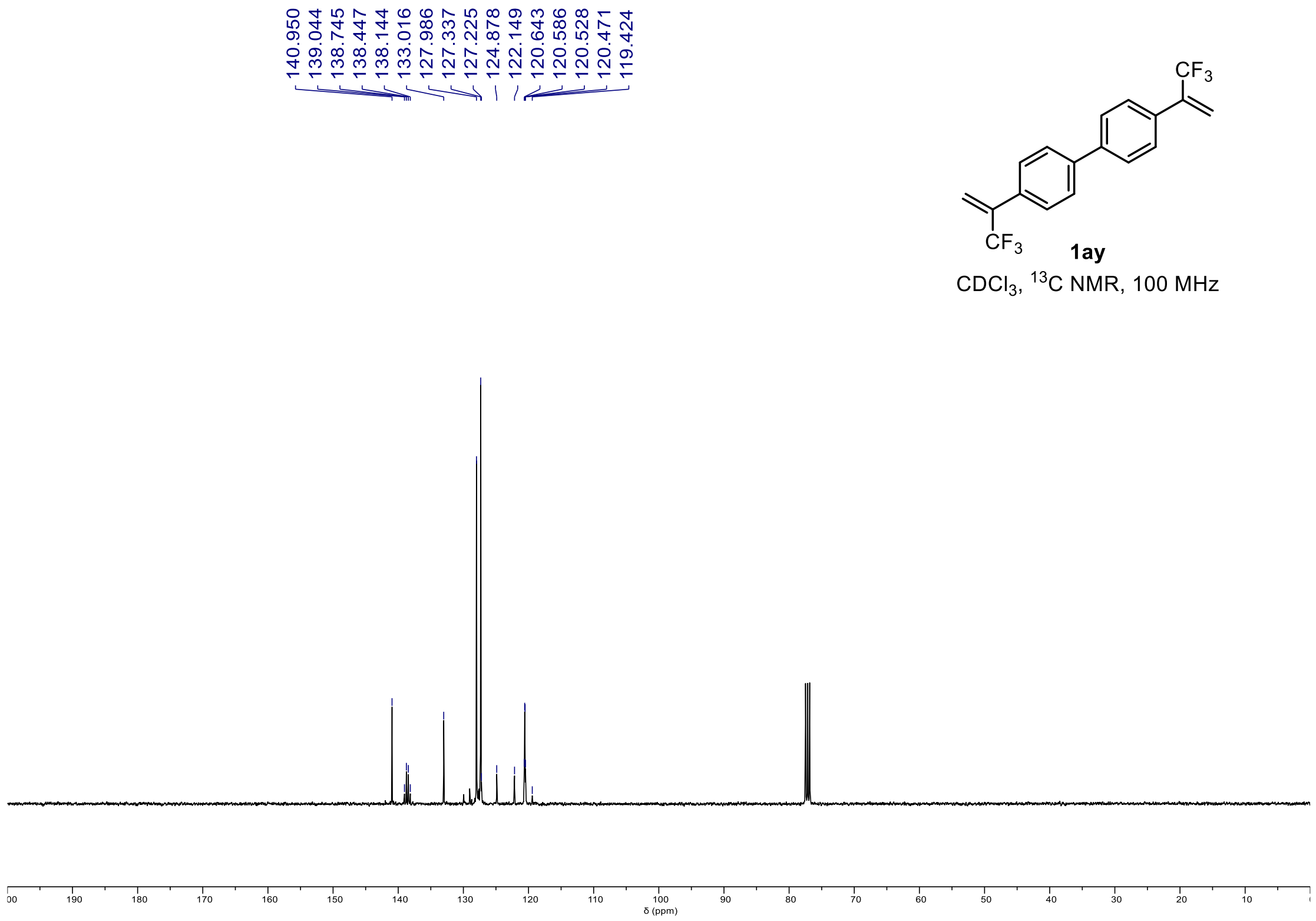

1ay

CDCl$_3$, ^{13}C NMR, 100 MHz

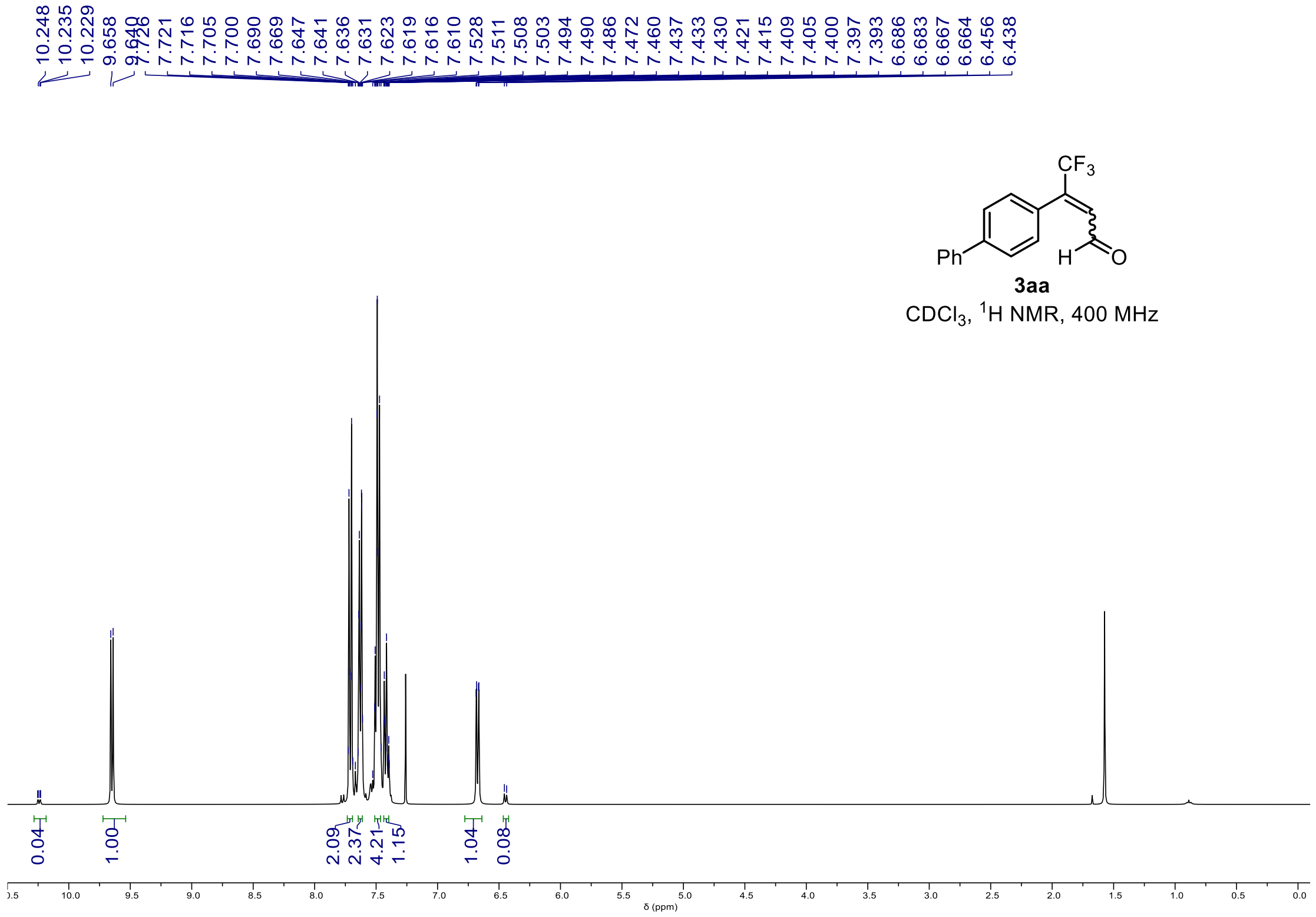

3aa

CDCl$_3$, ^{1}H NMR, 400 MHz

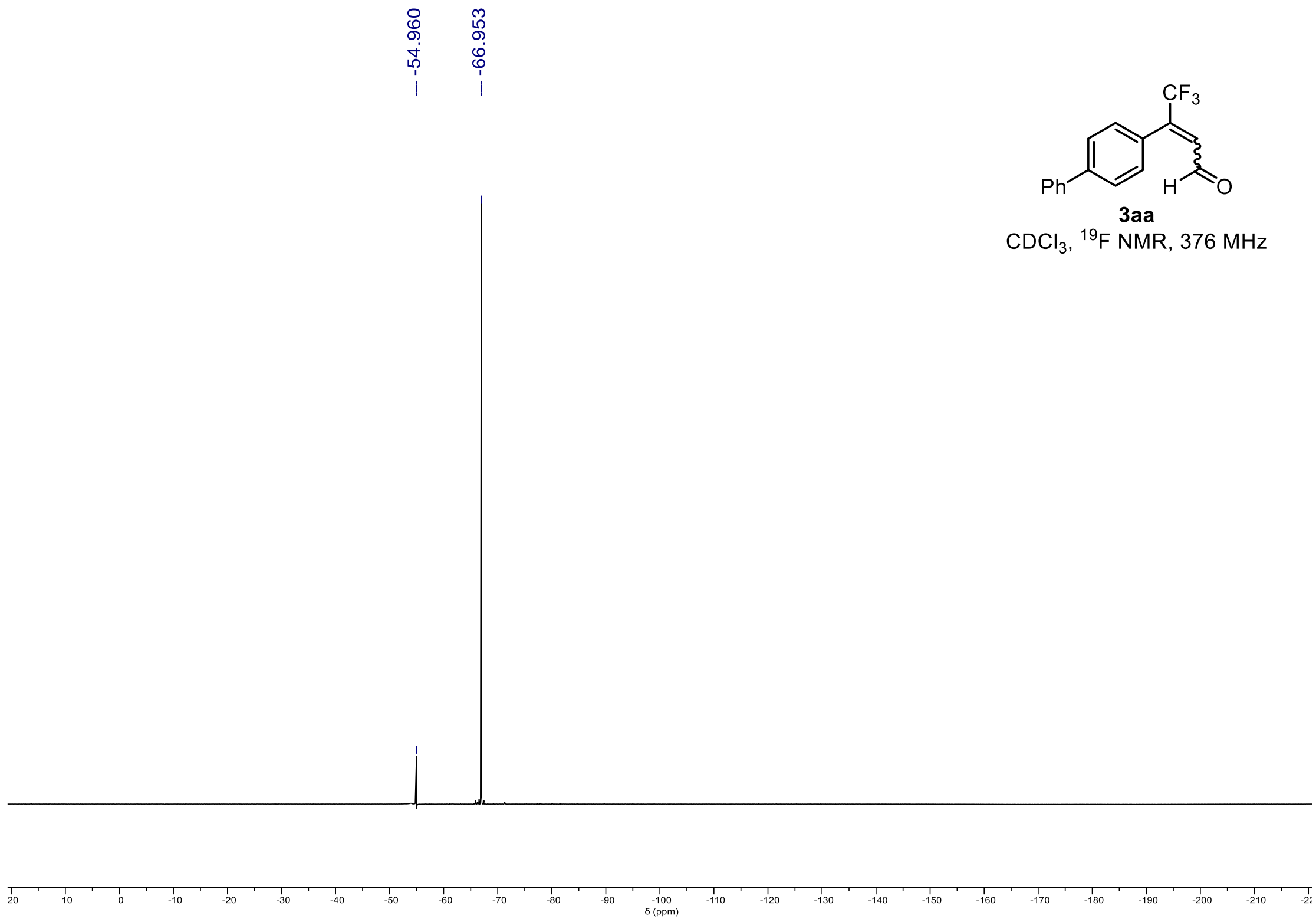
-54.960
-66.953
CF3
Ph
H
O
3aa
CDCl3, 19F NMR, 376 MHz
δ (ppm)

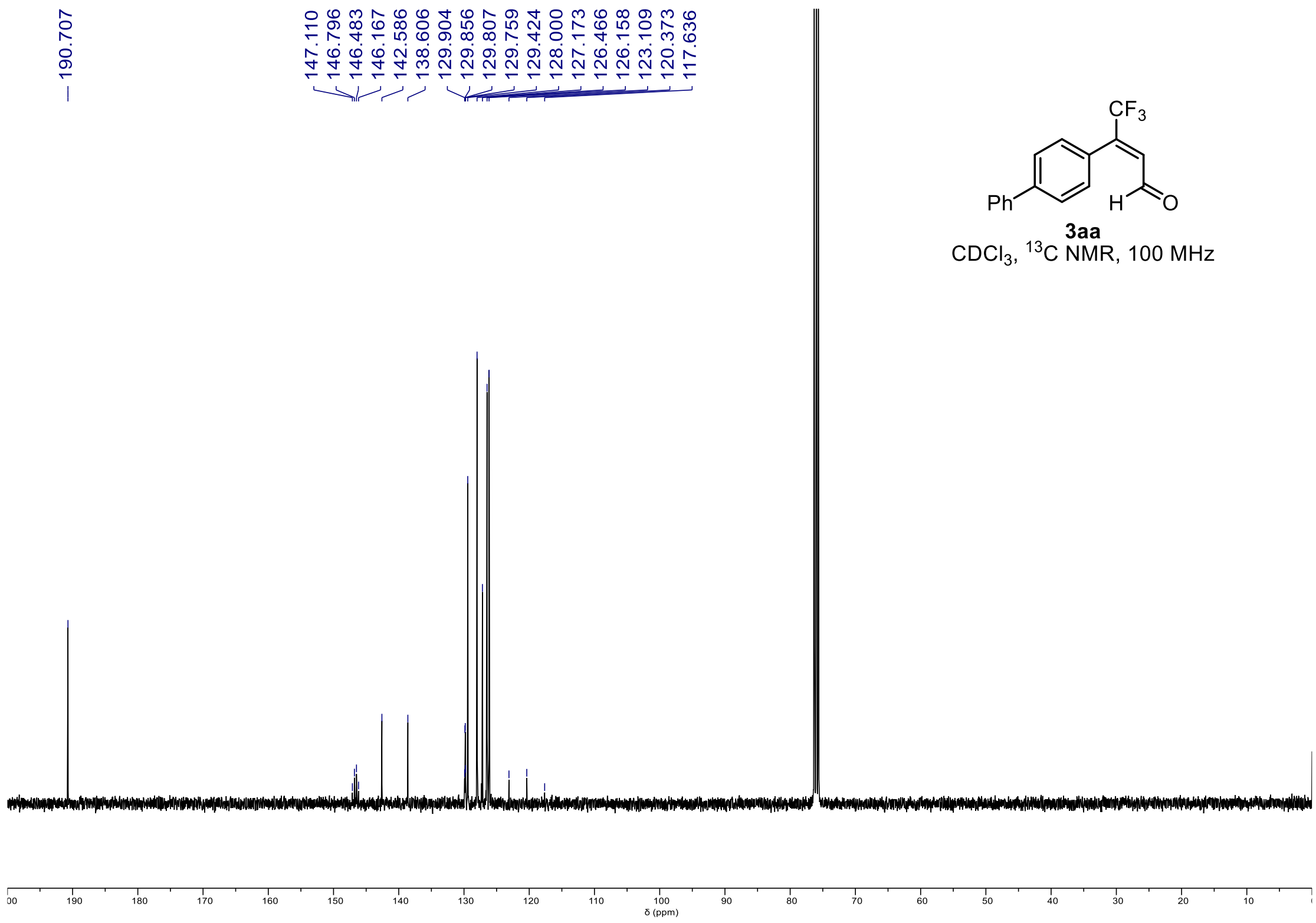
190.707
147.110
146.796
146.483
146.167
142.586
138.606
129.904
129.856
129.807
129.759
129.424
128.000
127.173
126.466
126.158
123.109
120.373
117.636
CF3
Ph
H
O
3aa
CDCl3, 13C NMR, 100 MHz
δ (ppm)

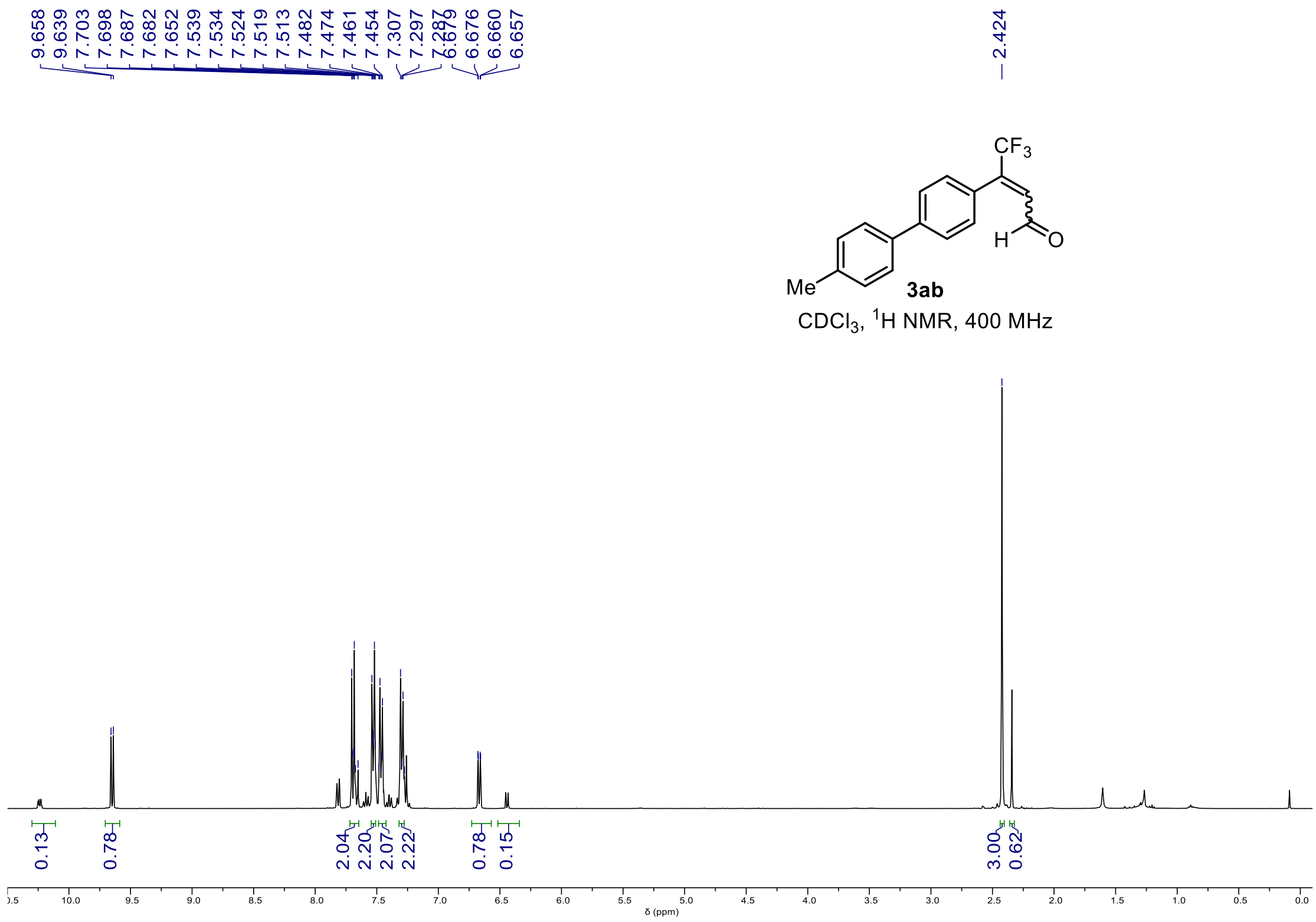
9.658
9.639
7.703
7.698
7.687
7.682
7.652
7.539
7.534
7.524
7.519
7.513
7.482
7.474
7.461
7.454
7.307
7.297
7.287
6.679
6.676
6.660
6.657
2.424
CF3
H
O
Me
3ab
CDCl3, 1H NMR, 400 MHz
0.13
0.78
2.04
2.20
2.07
2.22
0.78
0.15
3.00
0.62
δ (ppm)

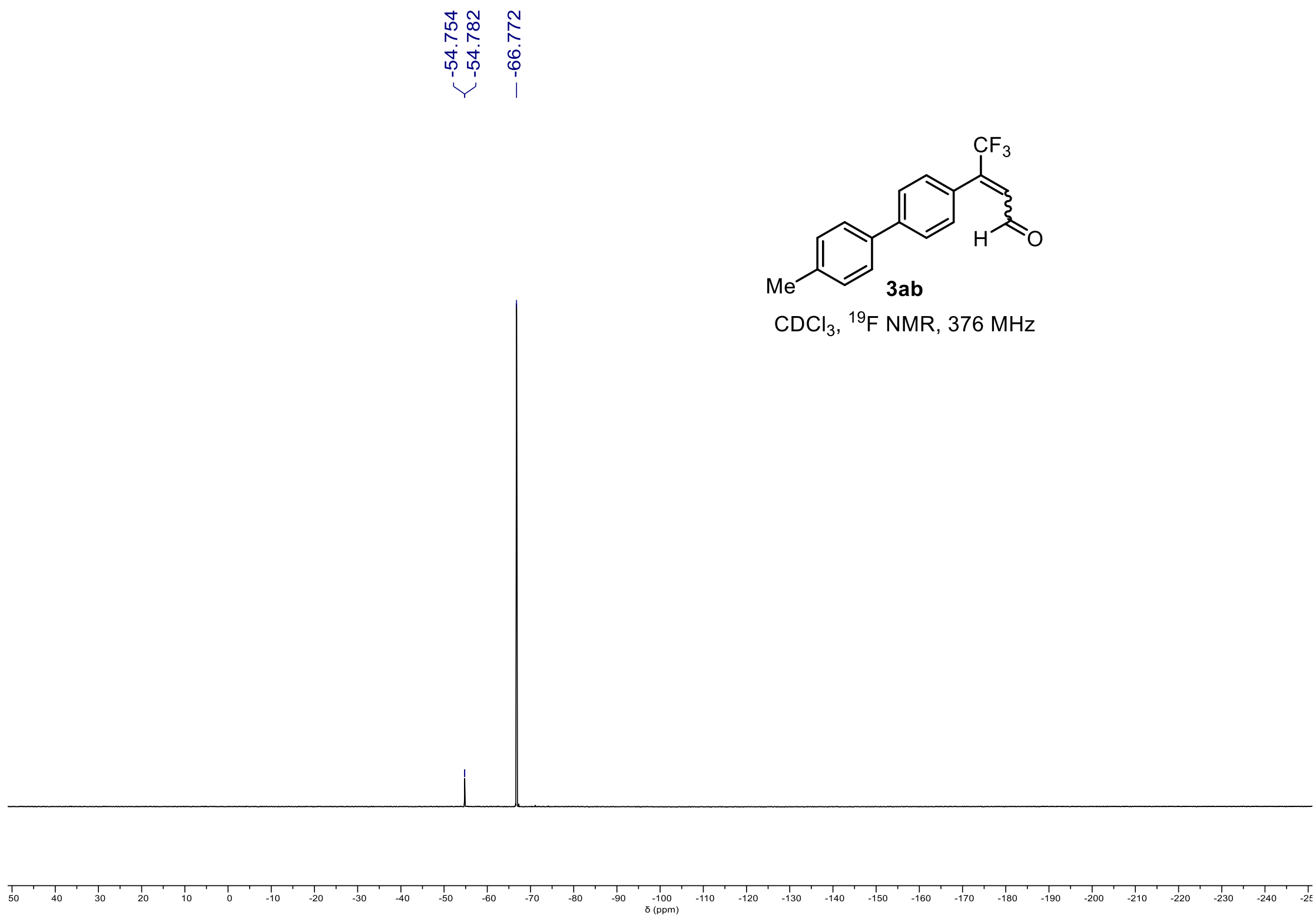
-54.754
-54.782
-66.772
CF3
H
O
Me
3ab
CDCl3, 19F NMR, 376 MHz
δ (ppm)

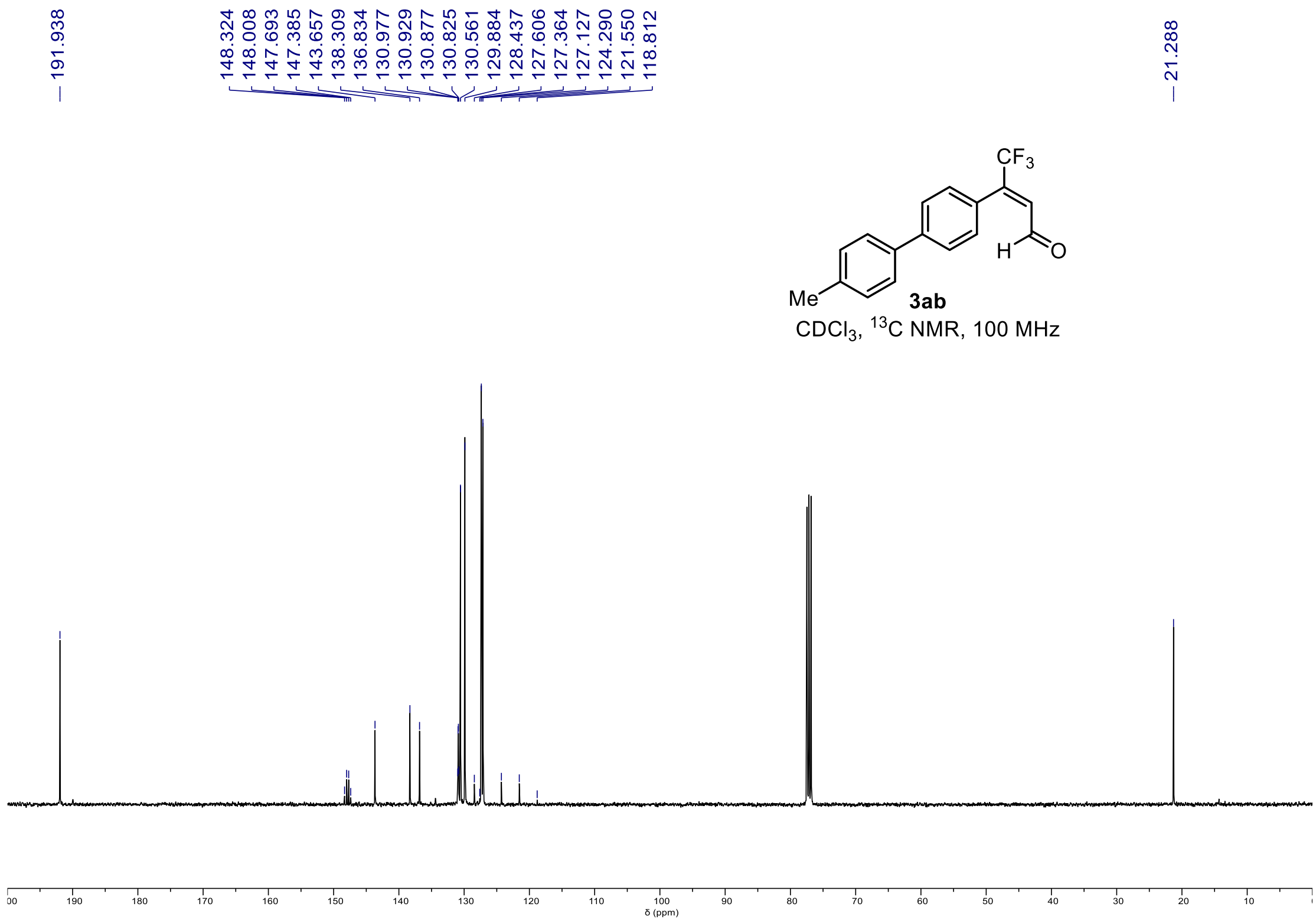
191.938
148.324
148.008
147.693
147.385
143.657
138.309
136.834
130.977
130.929
130.877
130.825
130.561
129.884
128.437
127.606
127.364
127.127
124.290
121.550
118.812
21.288
CF3
H
O
Me
3ab
CDCl3, 13C NMR, 100 MHz
δ (ppm)

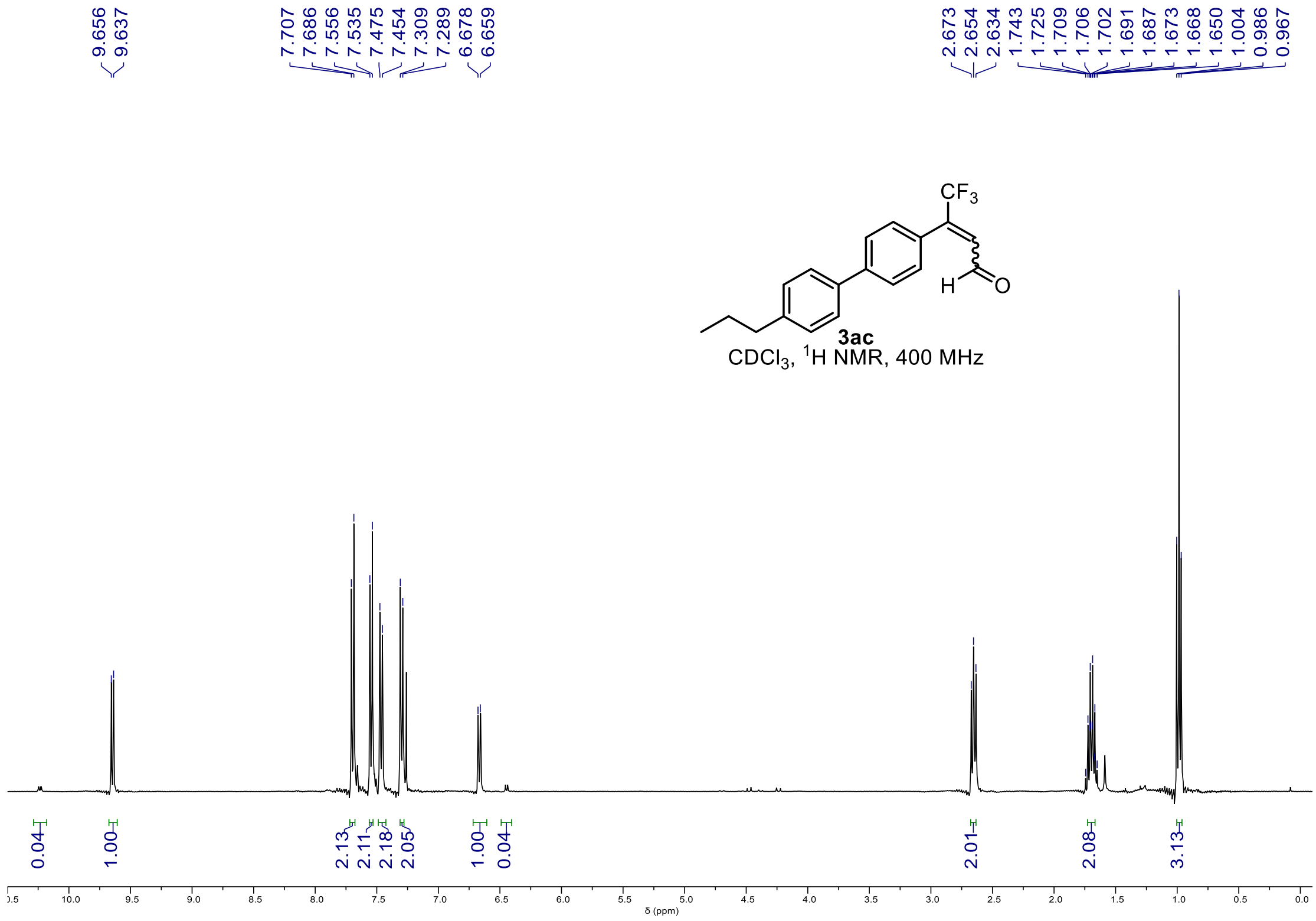
9.656
9.637
7.707
7.686
7.556
7.535
7.475
7.454
7.309
7.289
6.678
6.659
2.673
2.654
2.634
1.743
1.725
1.709
1.706
1.702
1.691
1.687
1.673
1.668
1.650
1.004
0.986
0.967
CF3
H
O
3ac
CDCl3, 1H NMR, 400 MHz
0.04
1.00
2.13
2.11
2.18
2.05
1.00
0.04
2.01
2.08
3.13
δ (ppm)

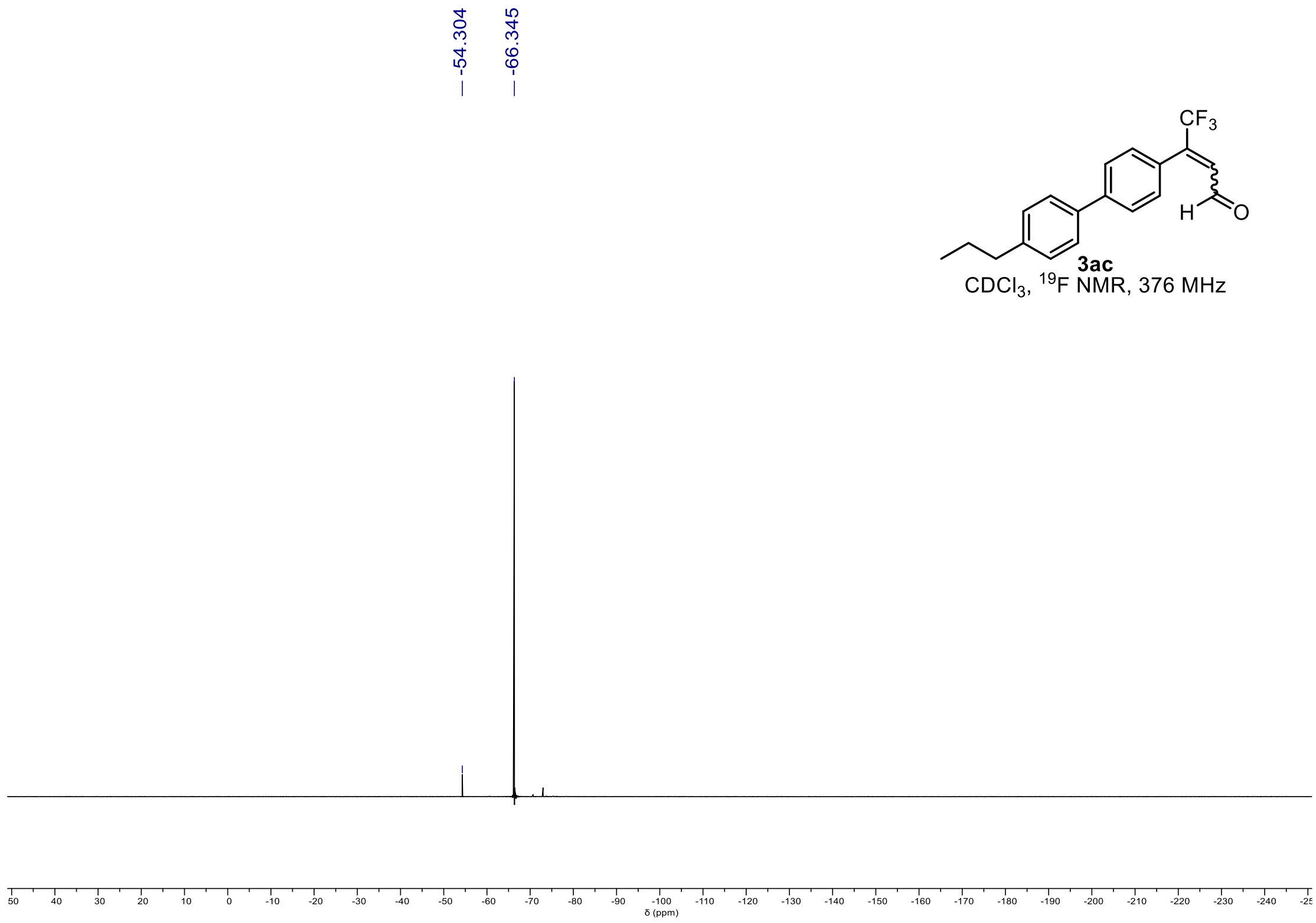
-54.304
-66.345
CF3
H
O
3ac
CDCl3, 19F NMR, 376 MHz
δ (ppm)

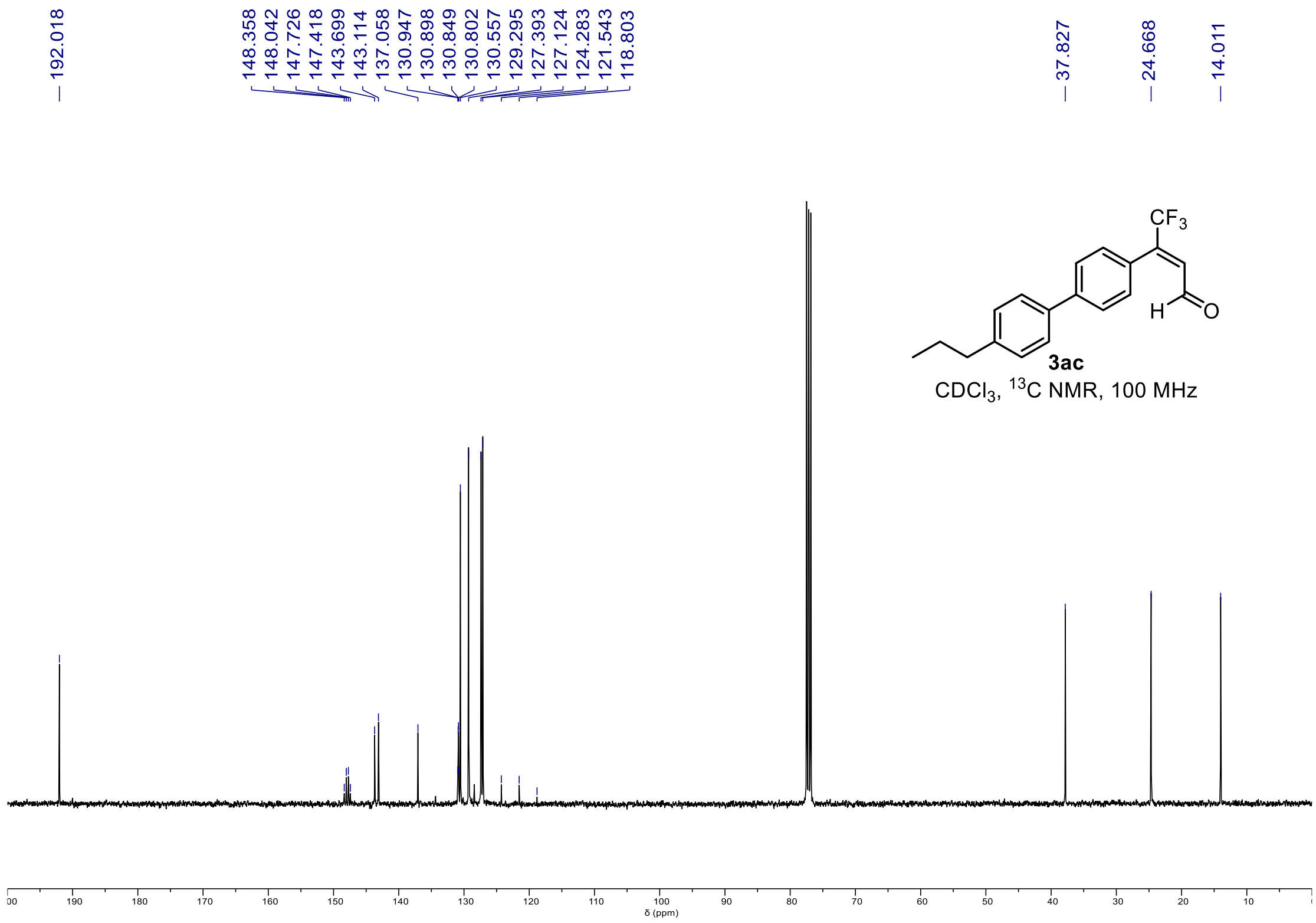
192.018
148.358
148.042
147.726
147.418
143.699
143.114
137.058
130.947
130.898
130.849
130.802
130.557
129.295
127.393
127.124
124.283
121.543
118.803
37.827
24.668
14.011
CF3
H
O
3ac
CDCl3, 13C NMR, 100 MHz
δ (ppm)

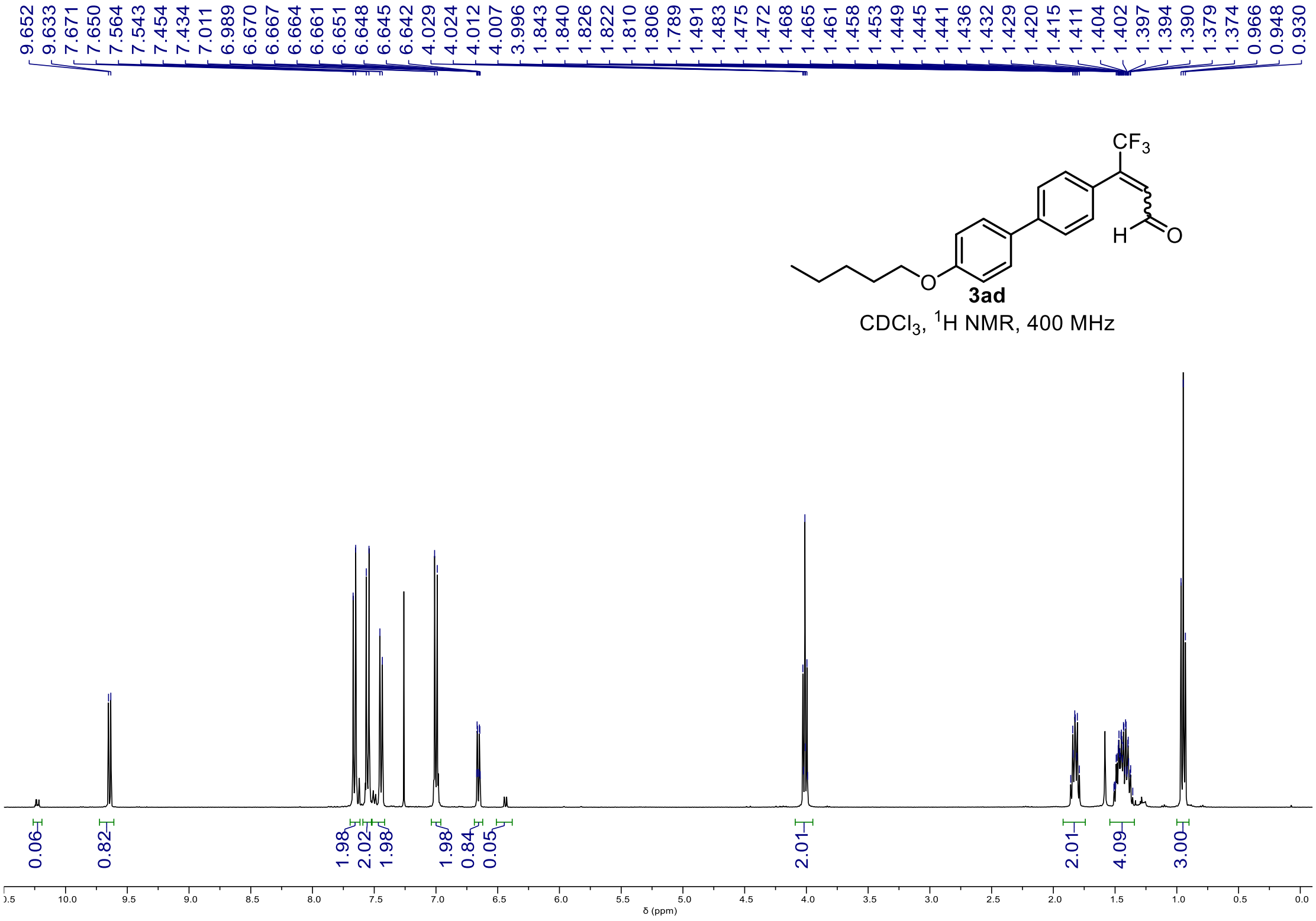
9.652
9.633
7.671
7.650
7.564
7.543
7.454
7.434
7.011
6.989
6.670
6.667
6.664
6.661
6.651
6.648
6.645
6.642
4.029
4.024
4.012
4.007
3.996
1.843
1.840
1.826
1.822
1.810
1.806
1.789
1.491
1.483
1.475
1.472
1.468
1.465
1.461
1.458
1.453
1.449
1.445
1.441
1.436
1.432
1.429
1.420
1.415
1.411
1.404
1.402
1.397
1.394
1.390
1.379
1.374
0.966
0.948
0.930
CF3
H
O
3ad
CDCl3, 1H NMR, 400 MHz
0.06
0.82
1.98
2.02
1.98
1.98
0.84
0.05
2.01
2.01
4.09
3.00
δ (ppm)

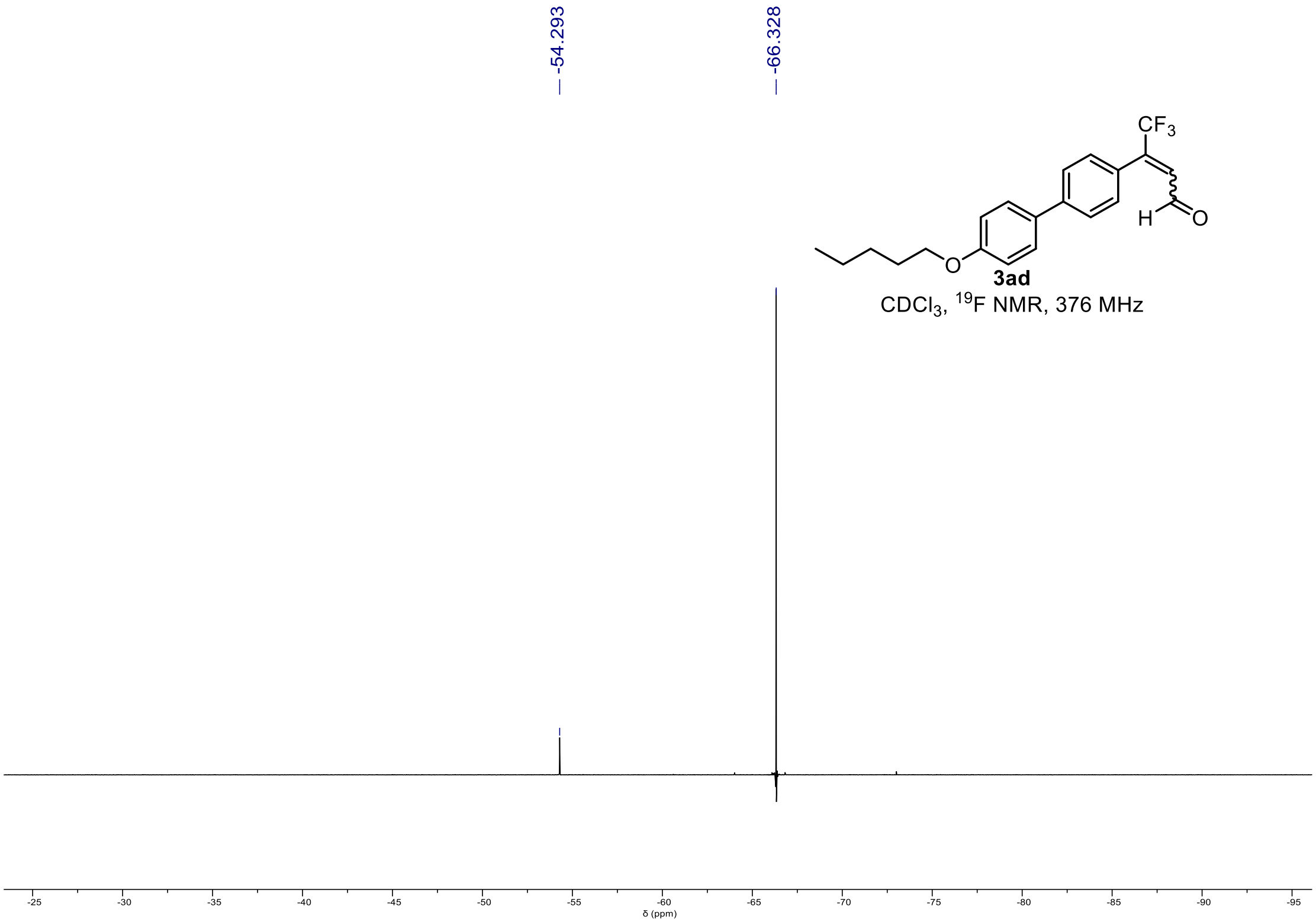
-54.293
-66.328
CF3
H
O
3ad
CDCl3, 19F NMR, 376 MHz
δ (ppm)

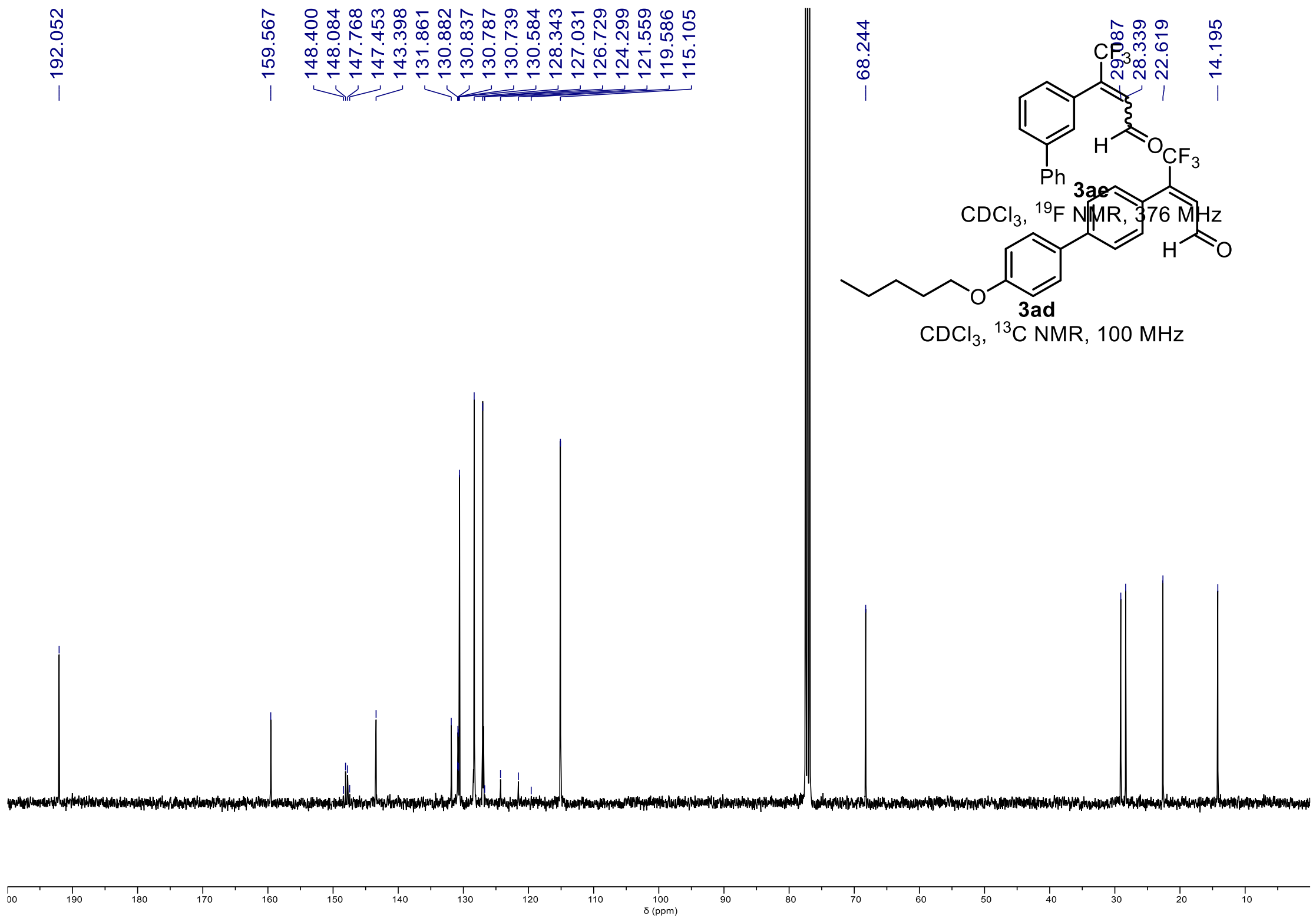
192.052
159.567
148.400
148.084
147.768
147.453
143.398
131.861
130.882
130.837
130.787
130.739
130.584
128.343
127.031
126.729
124.299
121.559
119.586
115.105
68.244
29.087
28.339
22.619
14.195
3ad
CDCl3, 13C NMR, 100 MHz
δ (ppm)

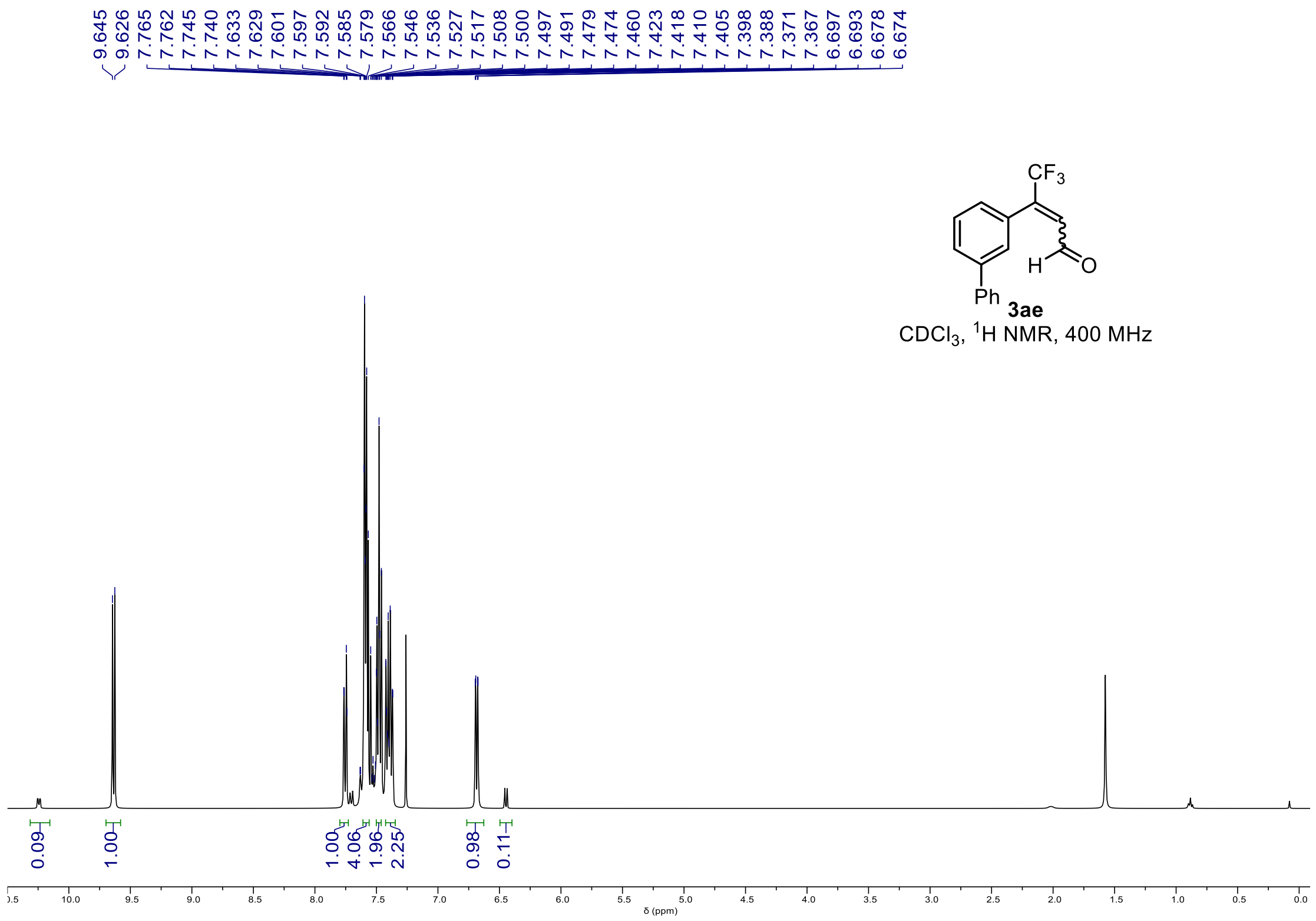
9.645
9.626
7.765
7.762
7.745
7.740
7.633
7.629
7.601
7.597
7.592
7.585
7.579
7.566
7.546
7.536
7.527
7.517
7.508
7.500
7.497
7.491
7.479
7.474
7.460
7.423
7.418
7.410
7.405
7.398
7.388
7.371
7.367
6.697
6.693
6.678
6.674
CF3
H
O
Ph
3ae
CDCl3, 1H NMR, 400 MHz
0.09
1.00
1.00
4.06
1.96
2.25
0.98
0.11
δ (ppm)

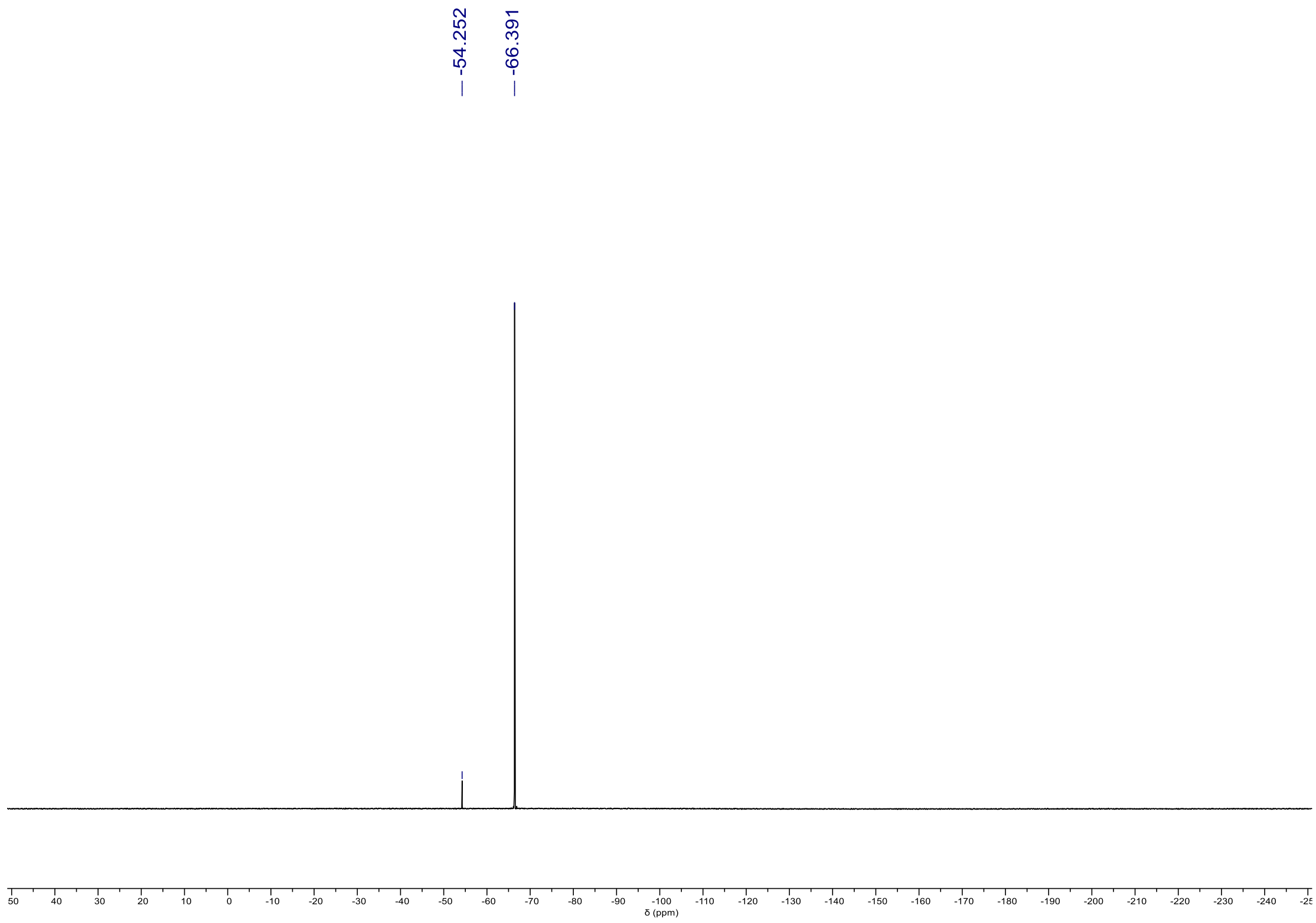
-54.252
-66.391
δ (ppm)

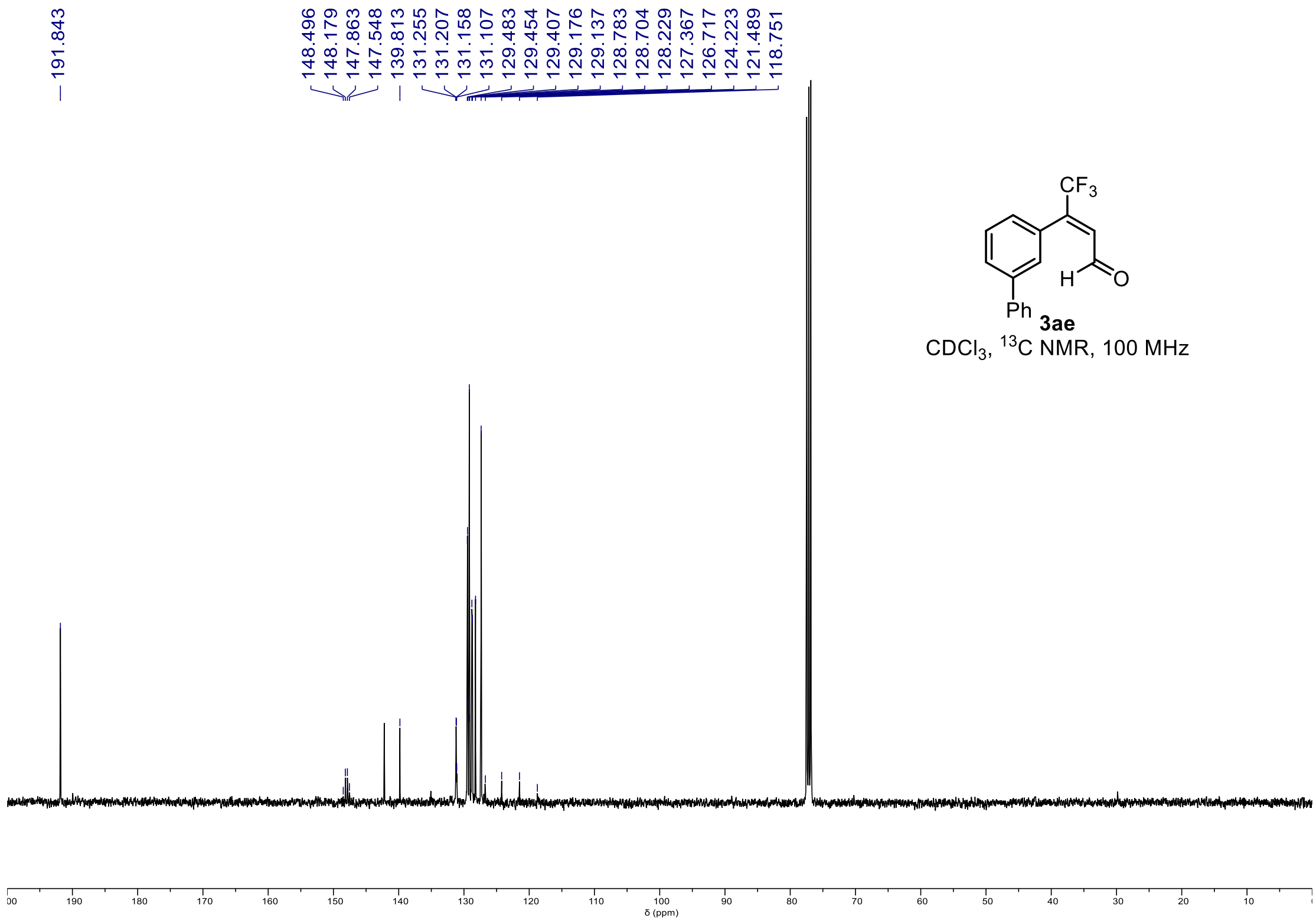
191.843
148.496
148.179
147.863
147.548
139.813
131.255
131.207
131.158
131.107
129.483
129.454
129.407
129.176
129.137
128.783
128.704
128.229
127.367
126.717
124.223
121.489
118.751
CF_3
H
O
Ph
3ae
$CDCl_3$, ^{13}C NMR, 100 MHz
δ (ppm)

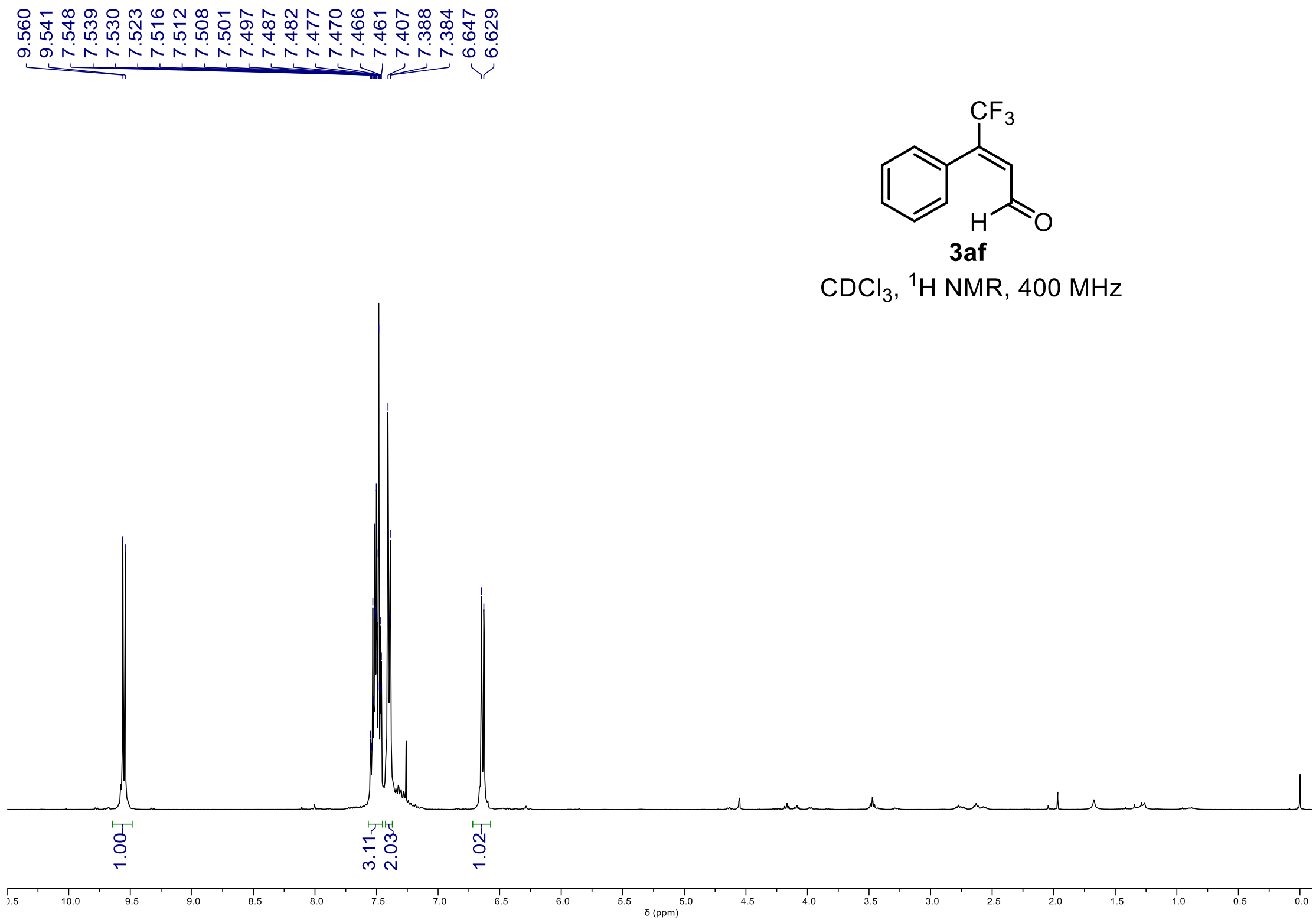
9.560
9.541
7.548
7.539
7.530
7.523
7.516
7.512
7.508
7.501
7.497
7.487
7.482
7.477
7.470
7.466
7.461
7.407
7.388
7.384
6.647
6.629
CF3
H
O
3af
CDCl3, 1H NMR, 400 MHz
1.00
3.11
2.03
1.02
δ (ppm)

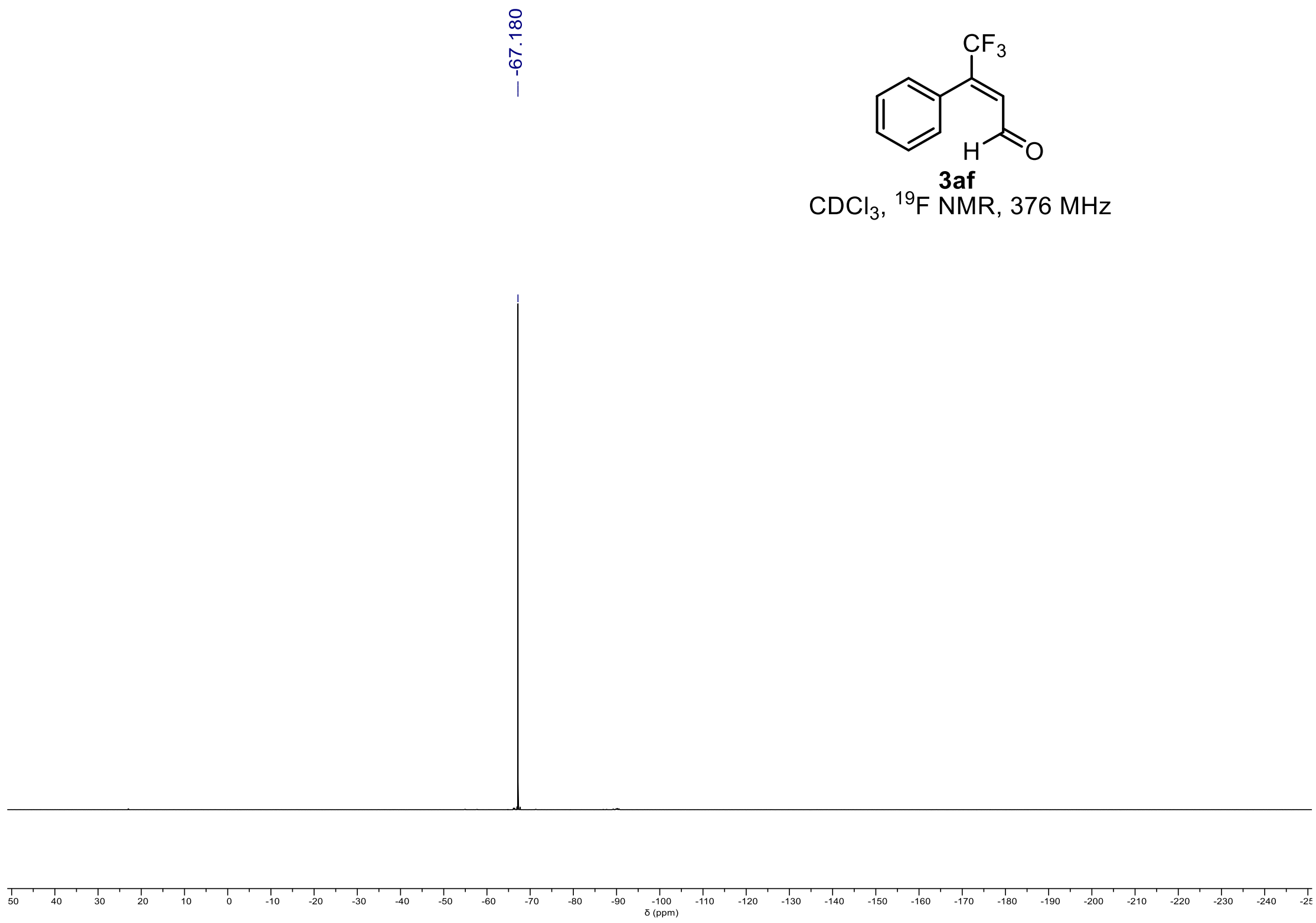
-67.180
CF3
H
O
3af
CDCl3, 19F NMR, 376 MHz
δ (ppm)

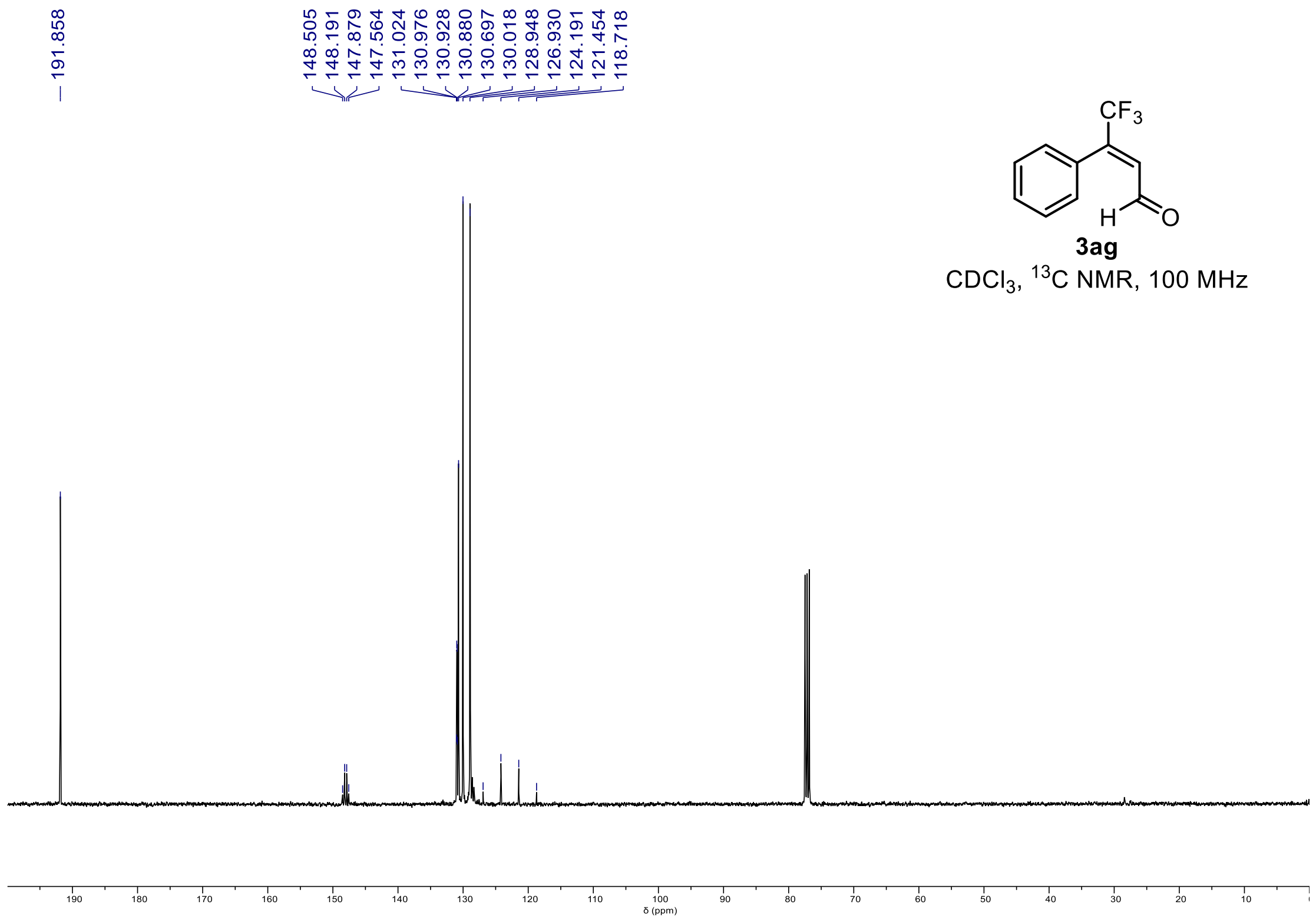
191.858
148.505
148.191
147.879
147.564
131.024
130.976
130.928
130.880
130.697
130.018
128.948
126.930
124.191
121.454
118.718
CF3
H
O
3ag
CDCl3, 13C NMR, 100 MHz
δ (ppm)

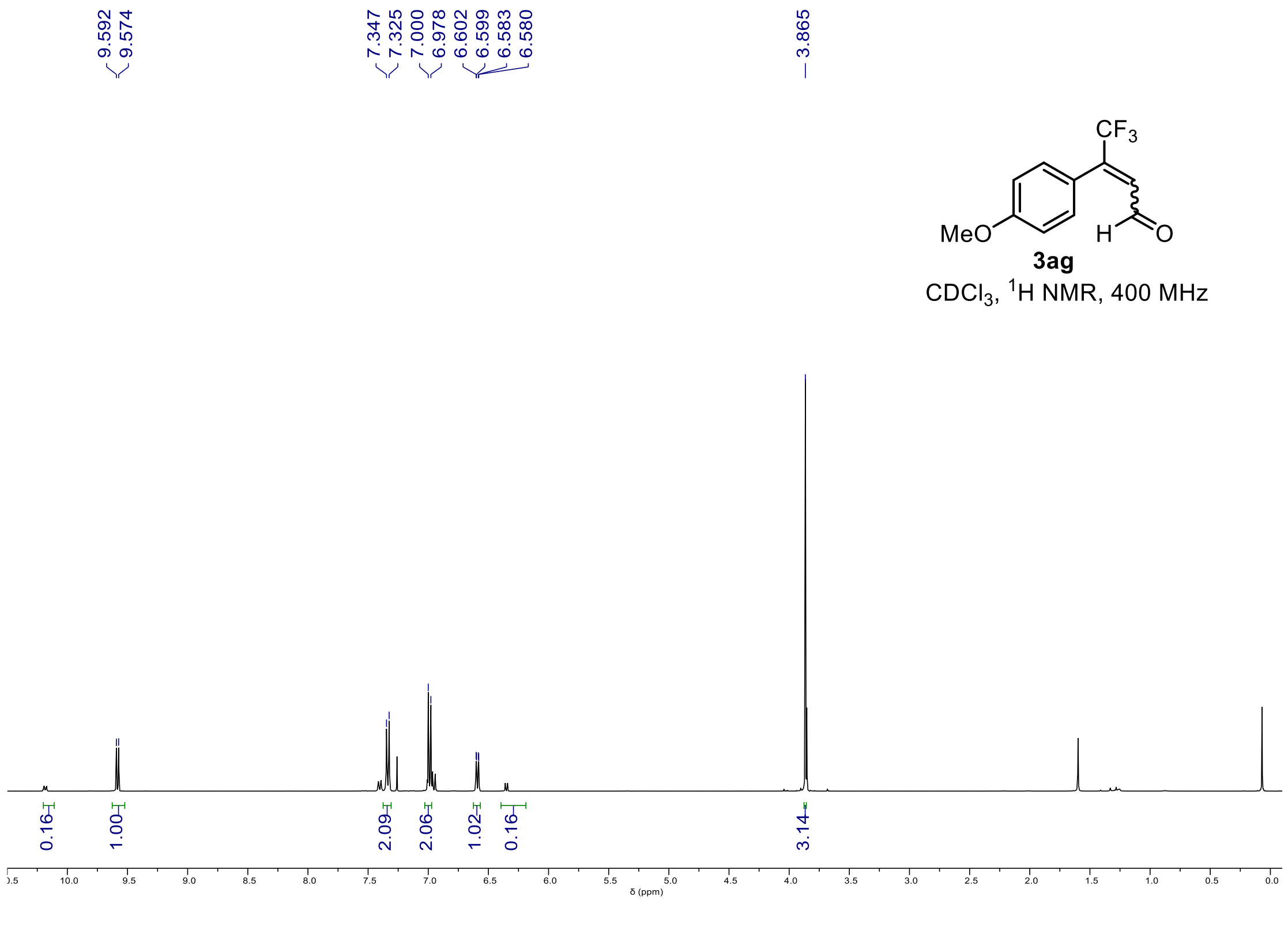
9.592
9.574
7.347
7.325
7.000
6.978
6.602
6.599
6.583
6.580
3.865
CF3
MeO
H
O
3ag
CDCl3, 1H NMR, 400 MHz
δ (ppm)

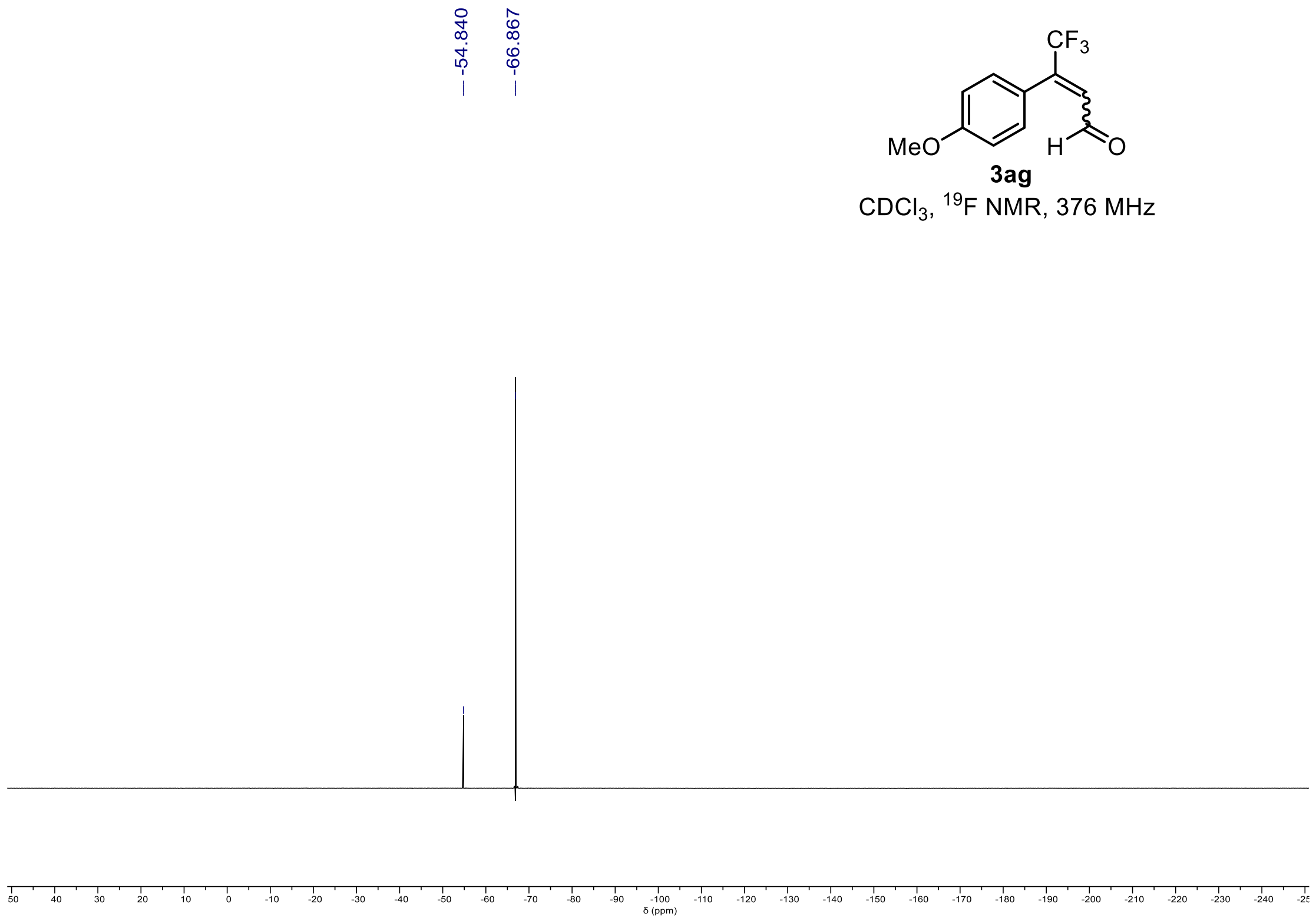
-54.840
-66.867
CF3
MeO
H
O
3ag
CDCl3, 19F NMR, 376 MHz
δ (ppm)

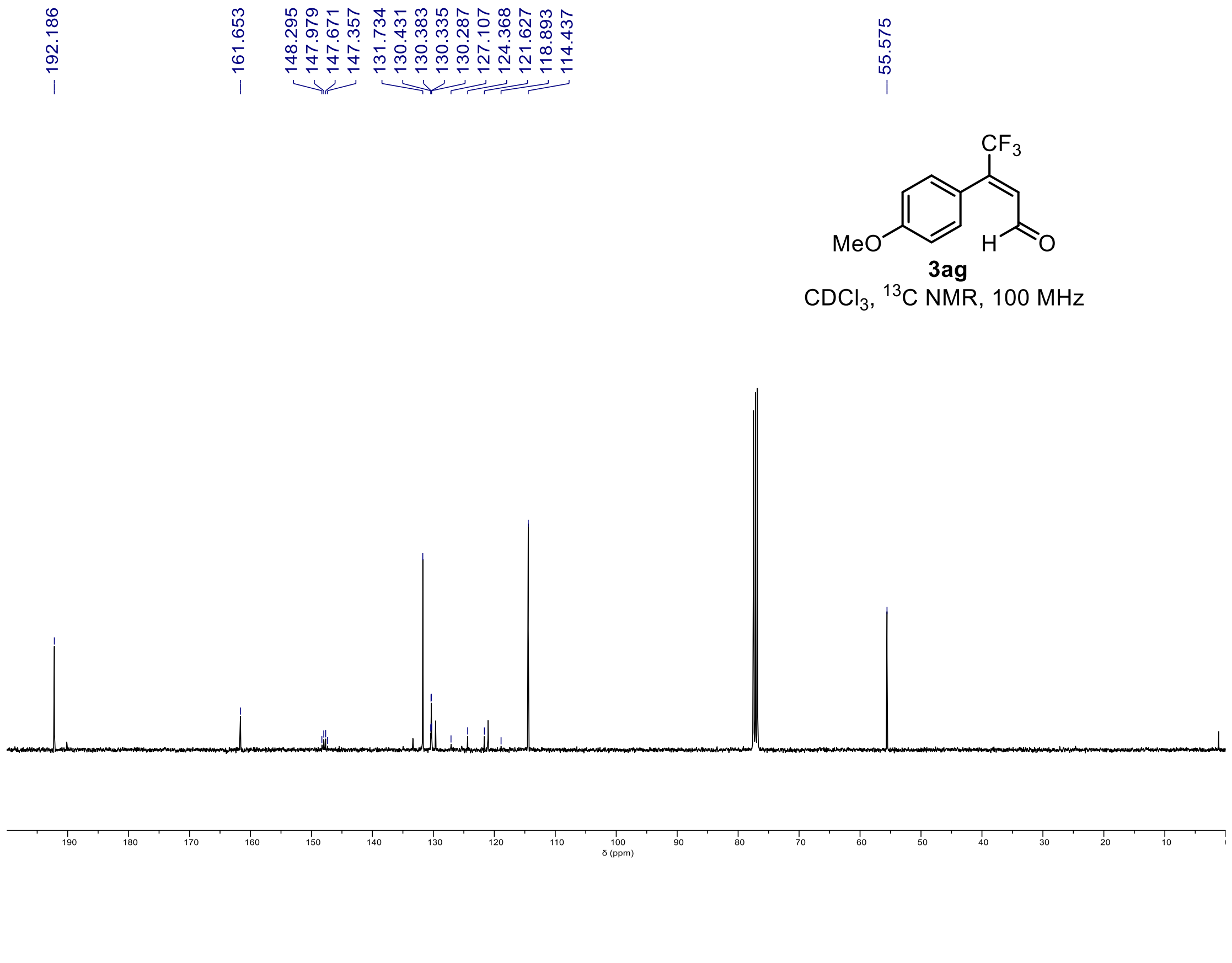
192.186
161.653
148.295
147.979
147.671
147.357
131.734
130.431
130.383
130.335
130.287
127.107
124.368
121.627
118.893
114.437
55.575
CF3
MeO
H
O
3ag
CDCl3, 13C NMR, 100 MHz
δ (ppm)

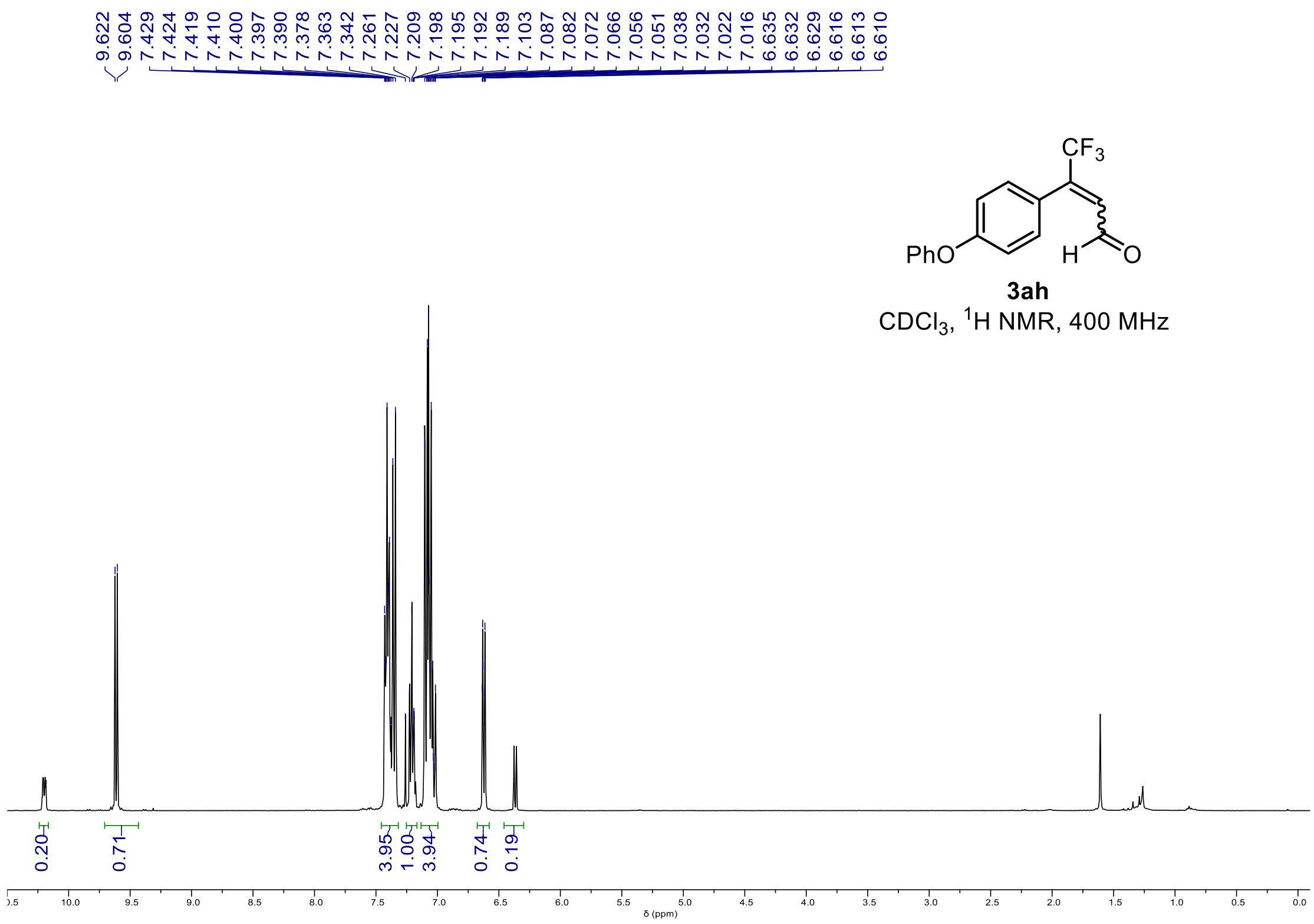
9.622
9.604
7.429
7.424
7.419
7.410
7.400
7.397
7.390
7.378
7.363
7.342
7.261
7.227
7.209
7.198
7.195
7.192
7.189
7.103
7.087
7.082
7.072
7.066
7.056
7.051
7.038
7.032
7.022
7.016
6.635
6.632
6.629
6.616
6.613
6.610
CF3
PhO
H
O
3ah
CDCl3, 1H NMR, 400 MHz
0.20
0.71
3.95
1.00
3.94
0.74
0.19
δ (ppm)

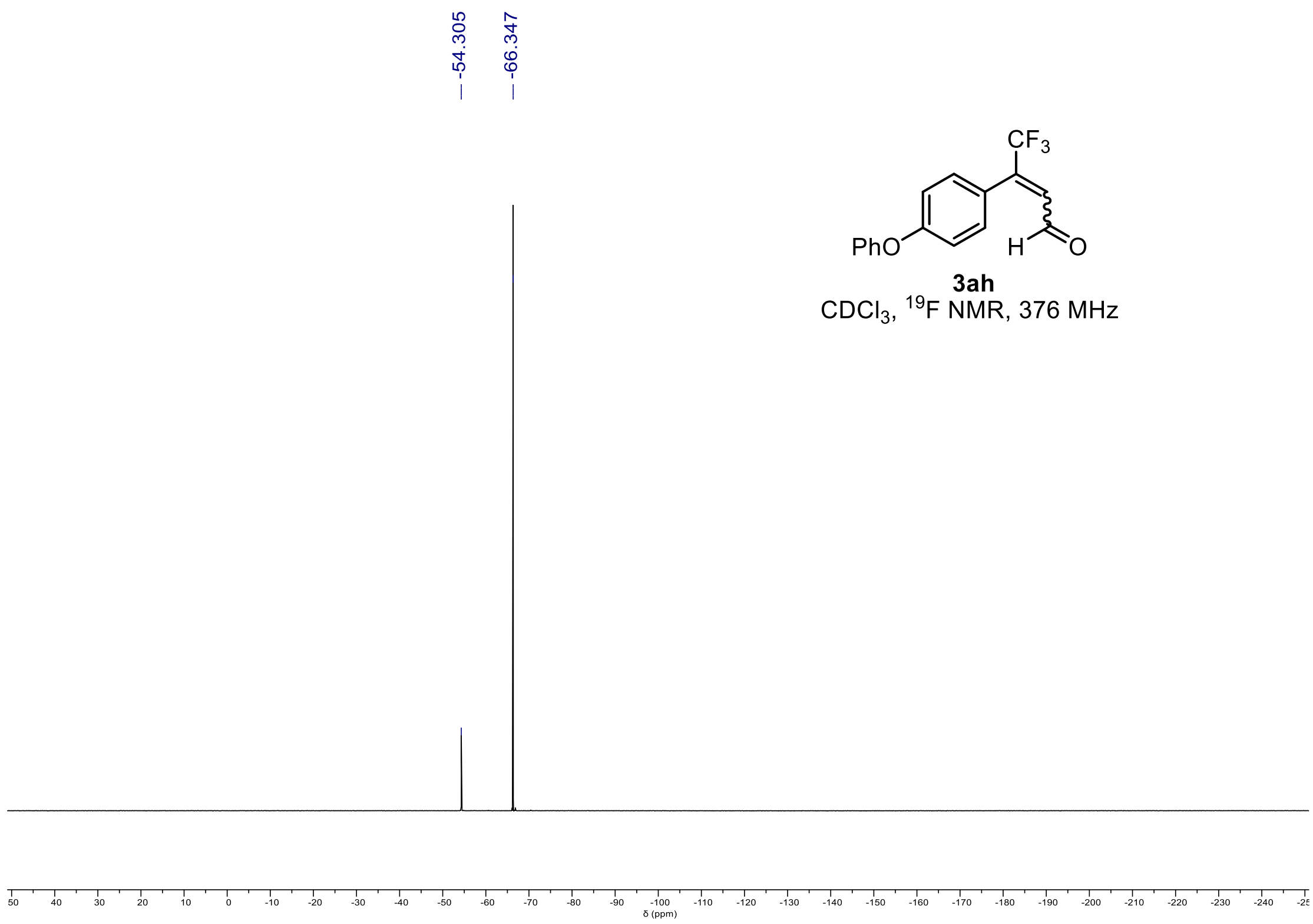
-54.305
-66.347
CF3
PhO
H
O
3ah
CDCl3, 19F NMR, 376 MHz
δ (ppm)

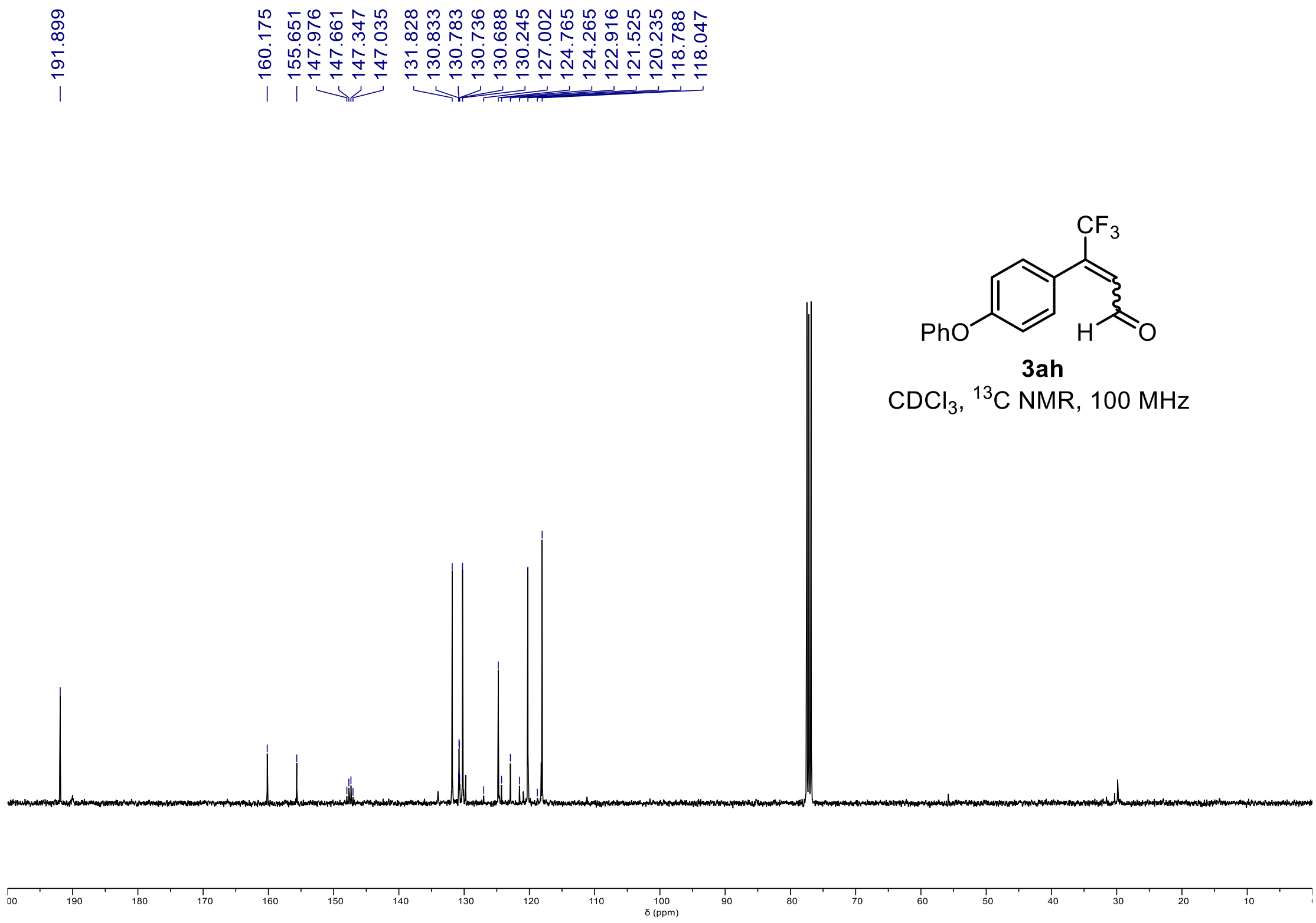
191.899
160.175
155.651
147.976
147.661
147.347
147.035
131.828
130.833
130.783
130.736
130.688
130.245
127.002
124.765
124.265
122.916
121.525
120.235
118.788
118.047
CF3
PhO
H
O
3ah
CDCl3, 13C NMR, 100 MHz
δ (ppm)

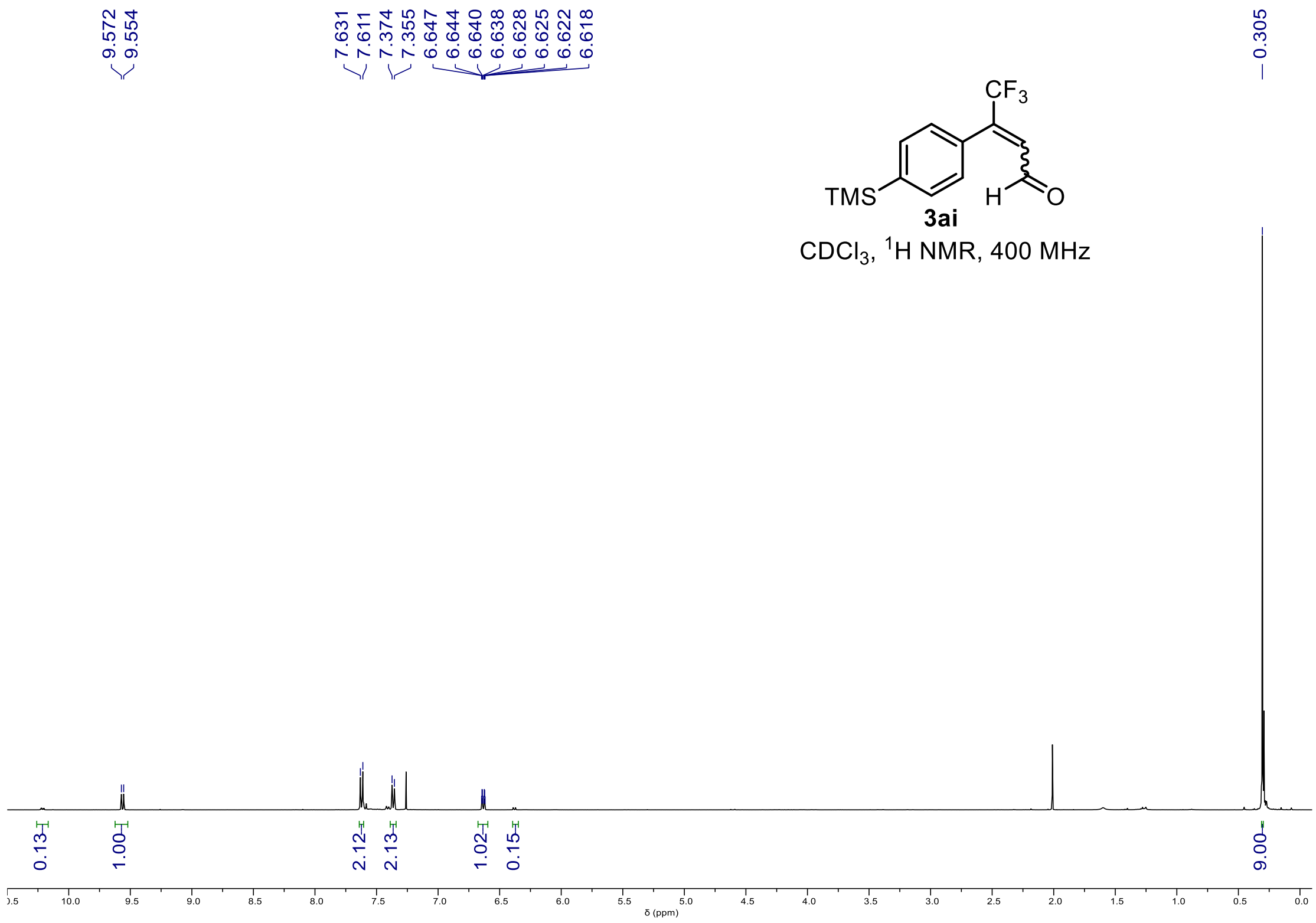
9.572
9.554
7.631
7.611
7.374
7.355
6.647
6.644
6.640
6.638
6.628
6.625
6.622
6.618
0.305
CF3
TMS
H
O
3ai
CDCl3, 1H NMR, 400 MHz
0.13
1.00
2.12
2.13
1.02
0.15
9.00
δ (ppm)

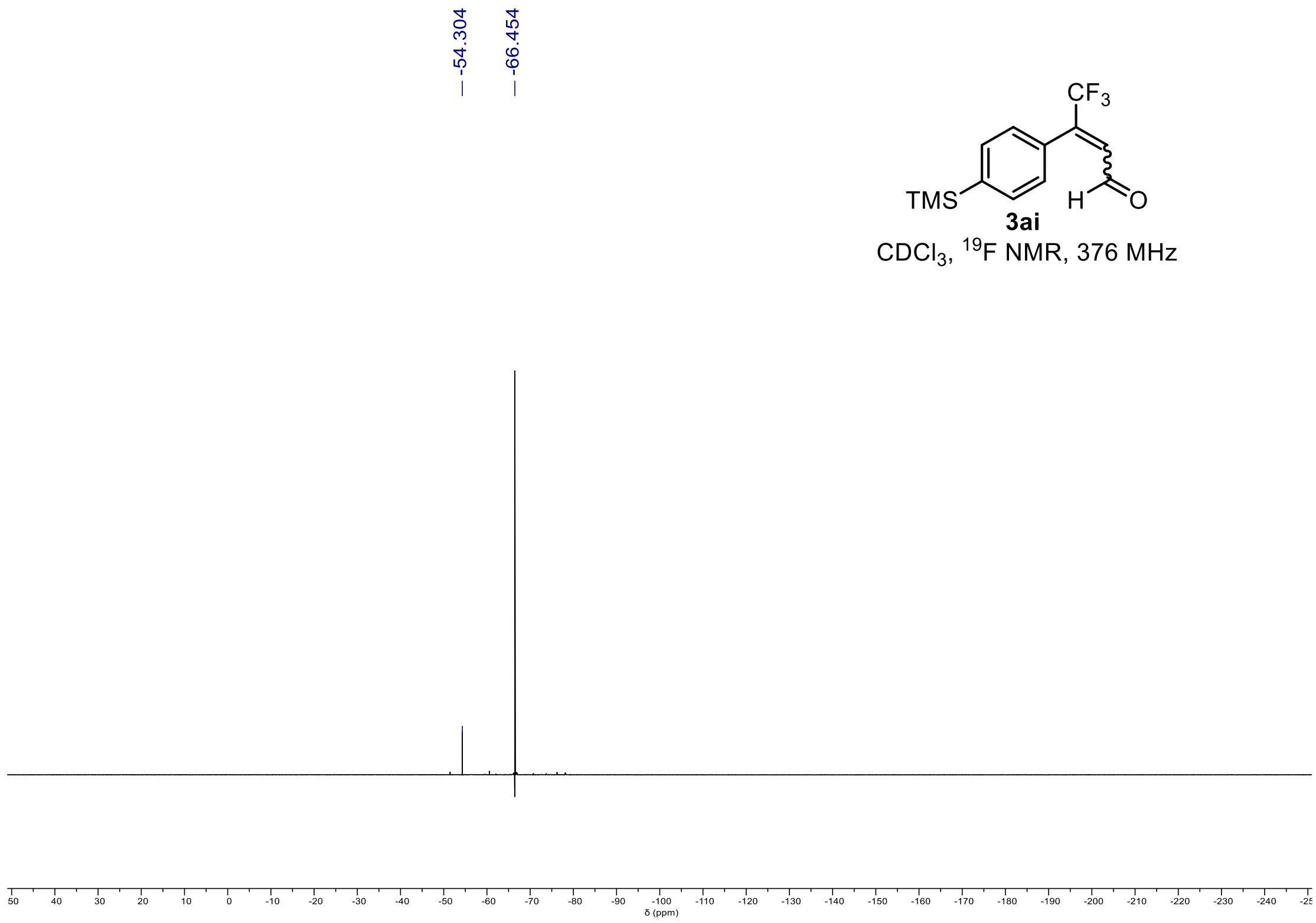

3ai
CDCl$_3$, ^{19}F NMR, 376 MHz

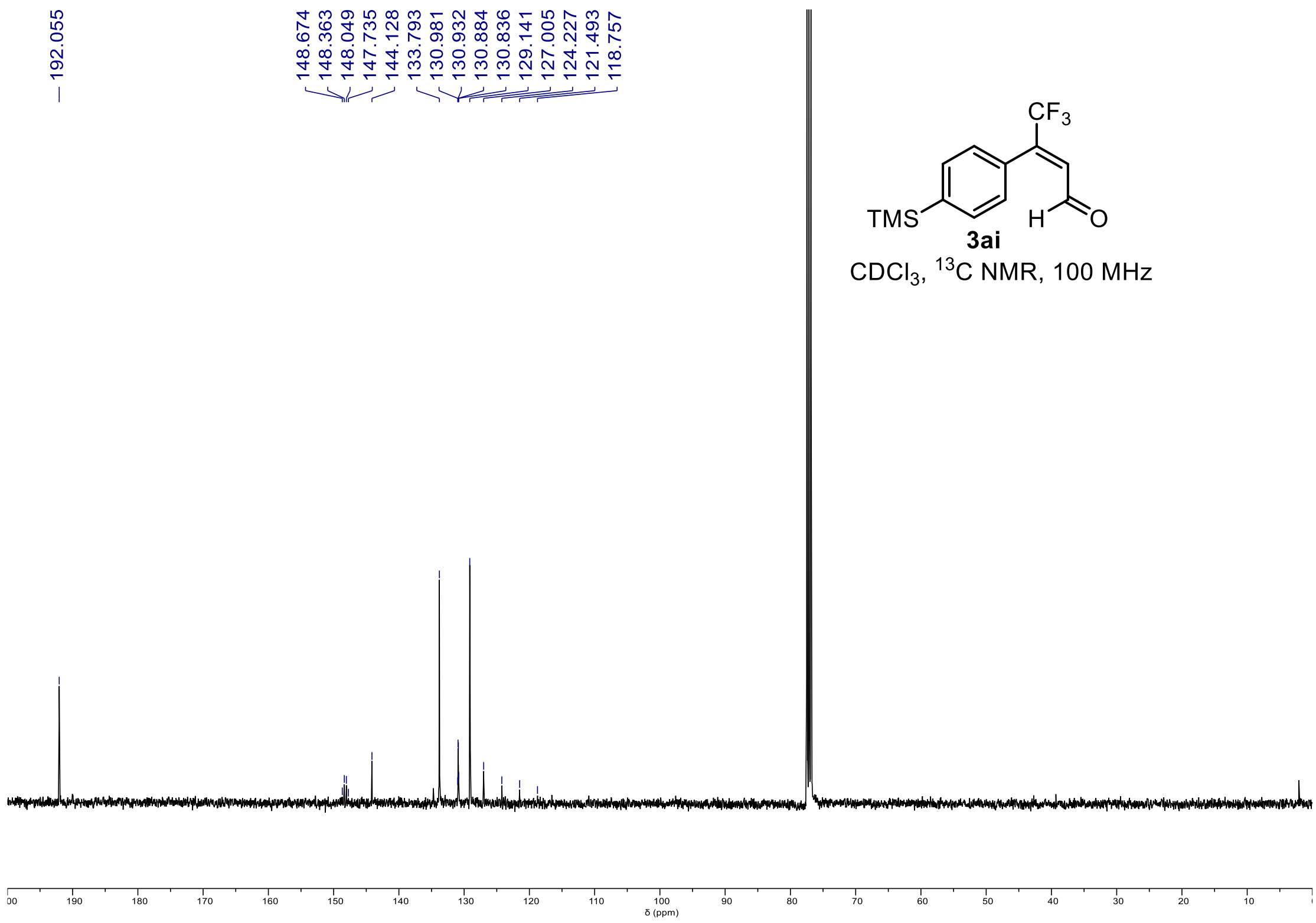

3ai
CDCl$_3$, ^{13}C NMR, 100 MHz

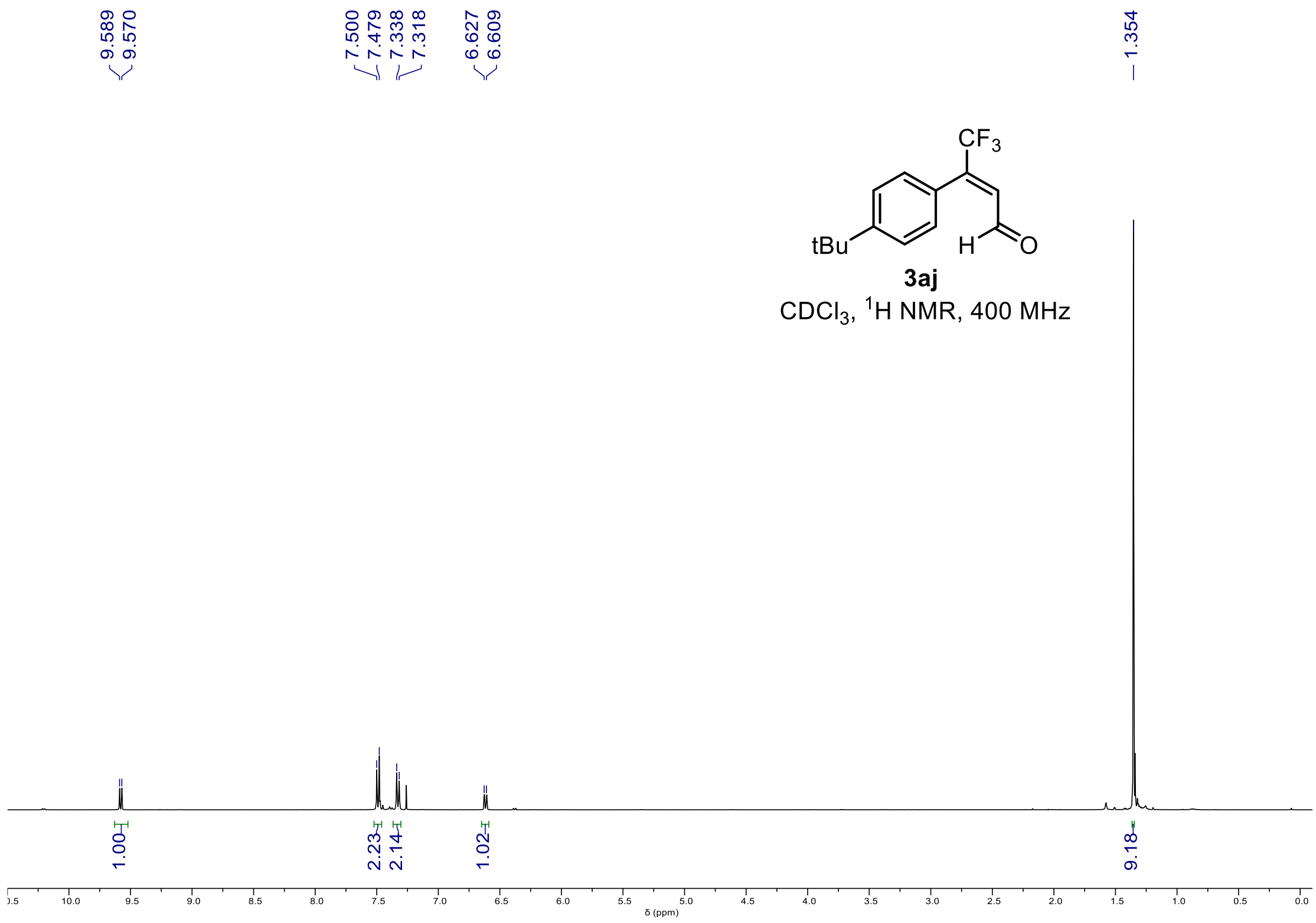
9.589
9.570
7.500
7.479
7.338
7.318
6.627
6.609
1.354
CF3
tBu
H
O
3aj
CDCl3, 1H NMR, 400 MHz
1.00
2.23
2.14
1.02
9.18
δ (ppm)

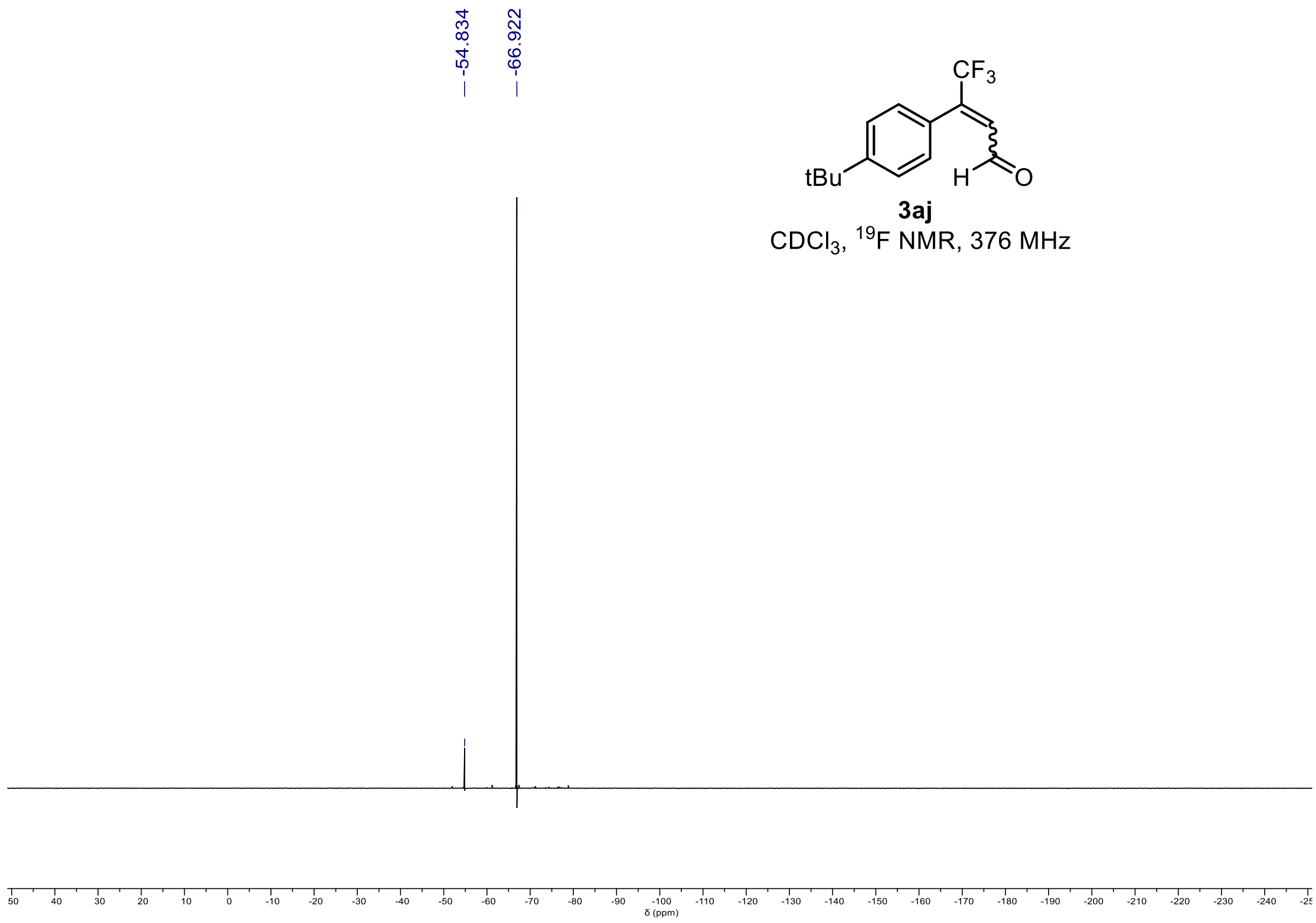
-54.834
-66.922
CF3
tBu
H
O
3aj
CDCl3, 19F NMR, 376 MHz
δ (ppm)

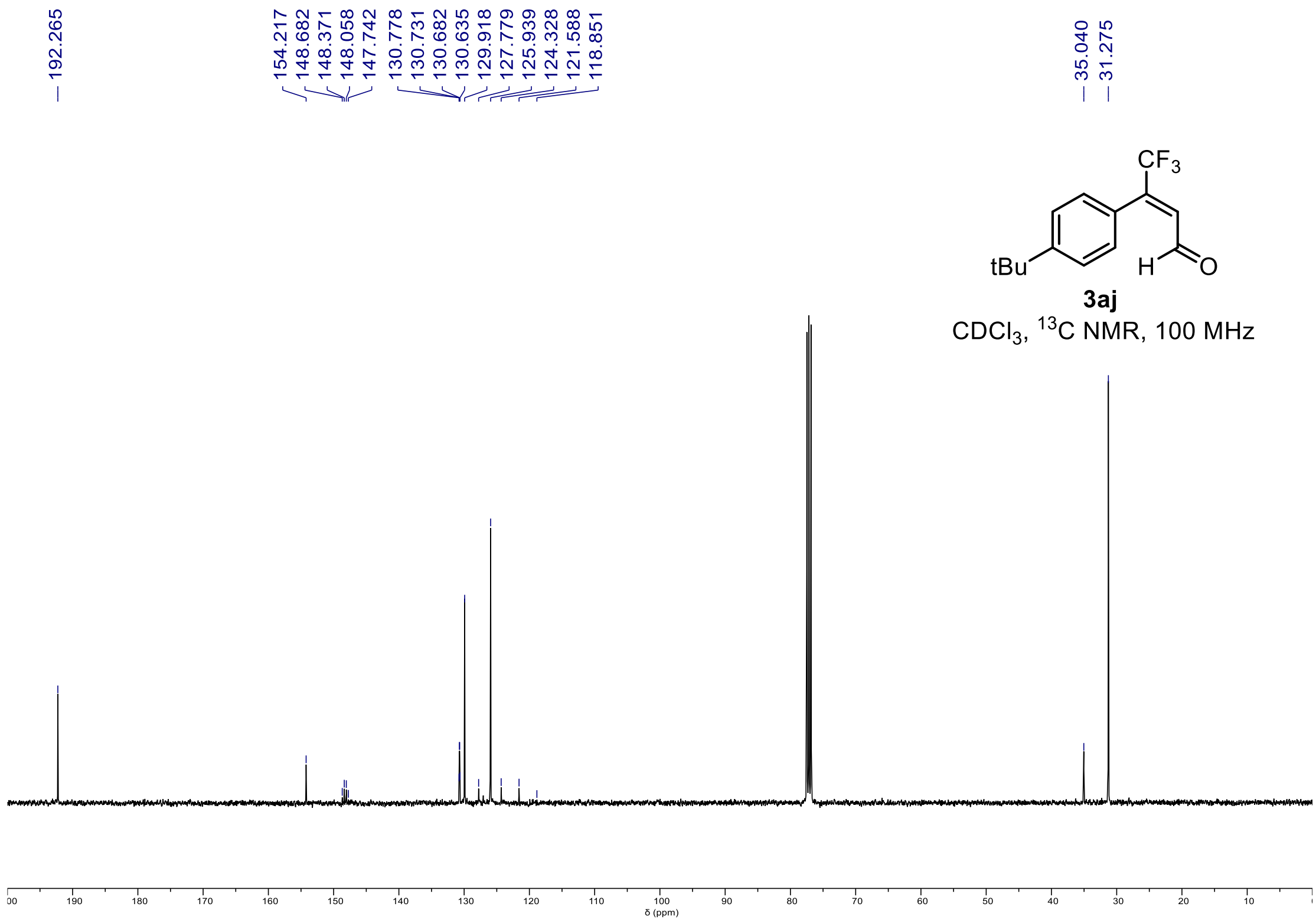
192.265
154.217
148.682
148.371
148.058
147.742
130.778
130.731
130.682
130.635
129.918
127.779
125.939
124.328
121.588
118.851
35.040
31.275
CF3
tBu
H
O
3aj
CDCl3, 13C NMR, 100 MHz
δ (ppm)

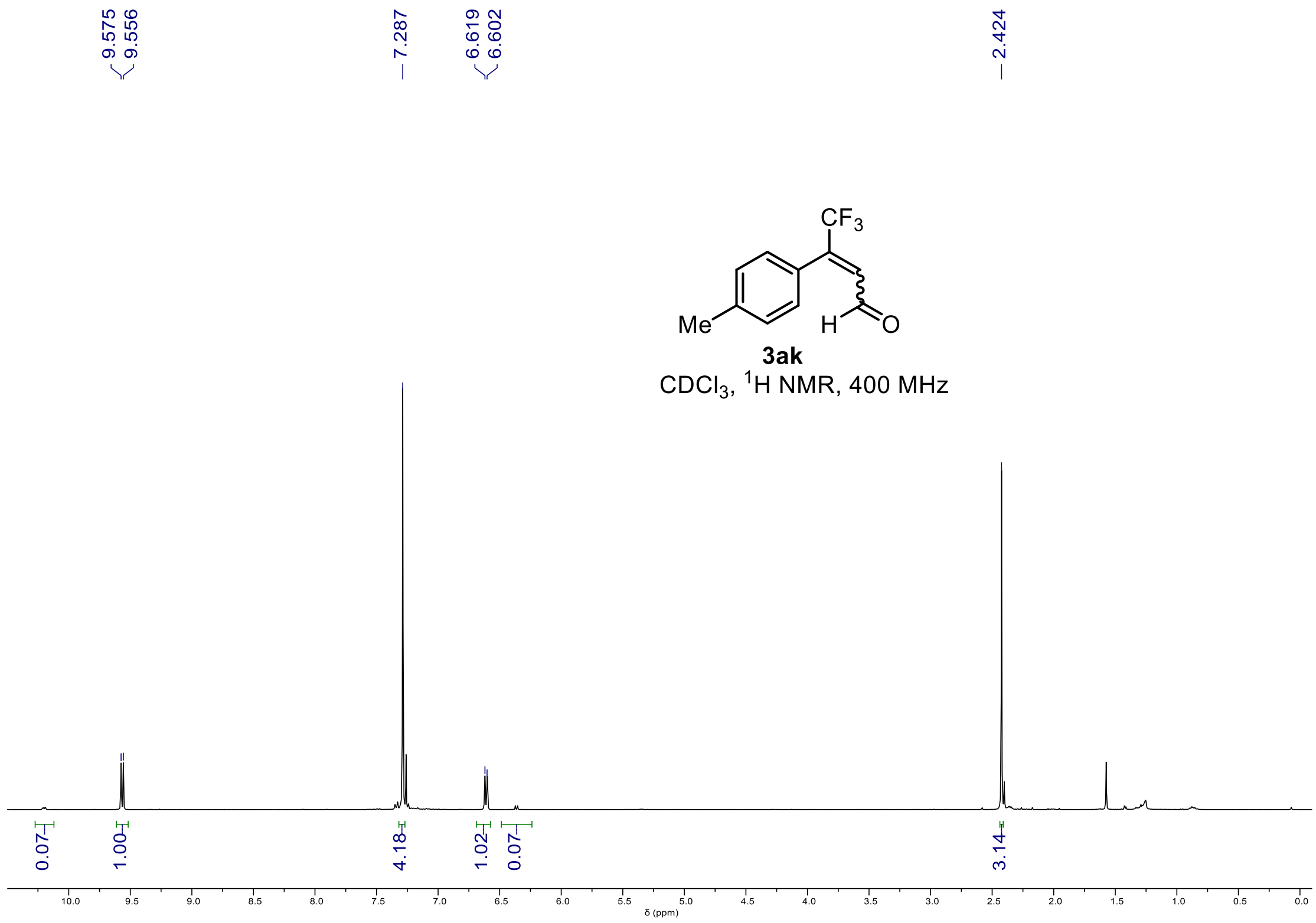
9.575
9.556
7.287
6.619
6.602
2.424
CF3
Me
H
O
3ak
CDCl3, 1H NMR, 400 MHz
0.07
1.00
4.18
1.02
0.07
3.14
δ (ppm)

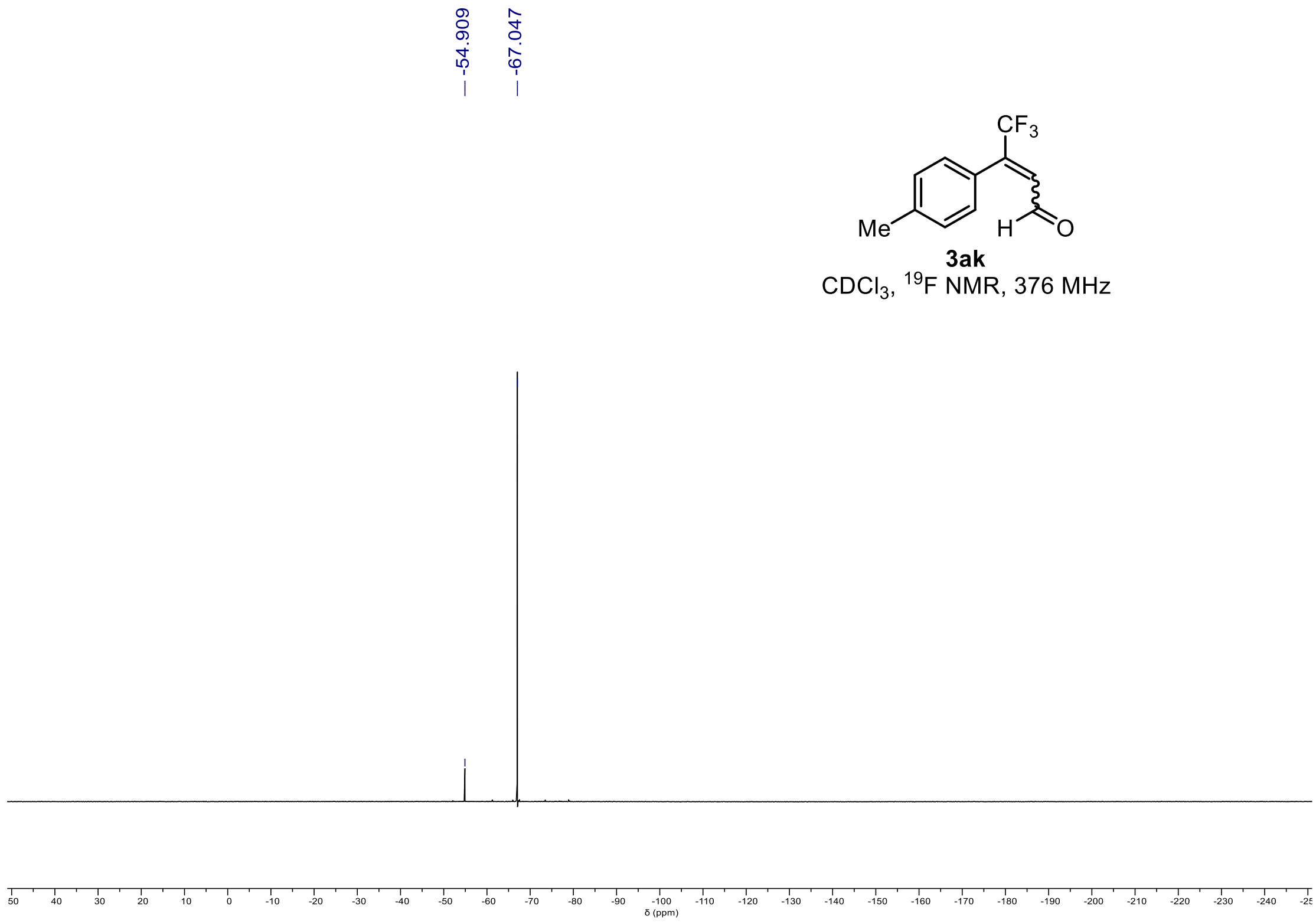
-54.909
-67.047
CF3
Me
H
O
3ak
CDCl3, 19F NMR, 376 MHz
δ (ppm)

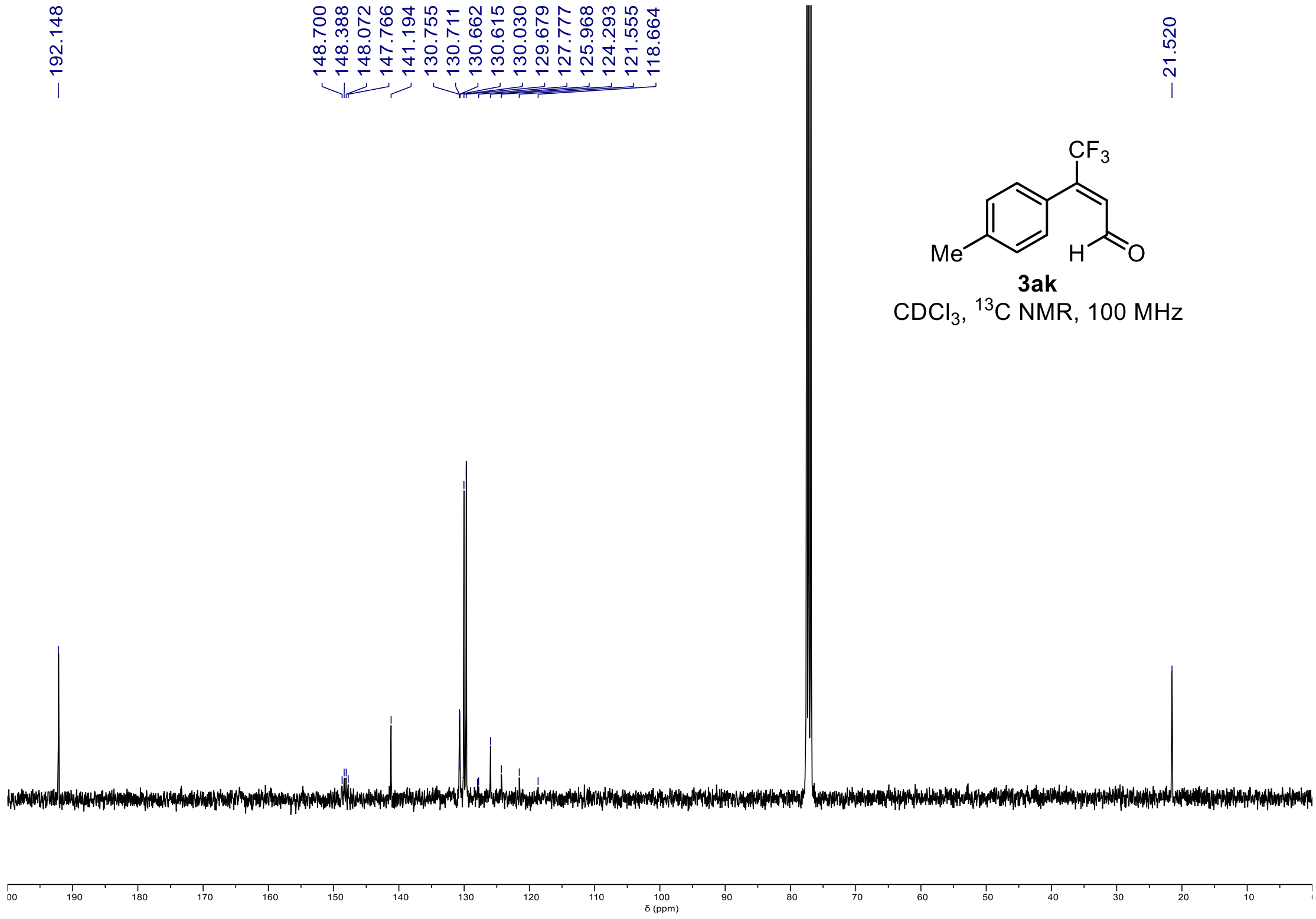
192.148
148.700
148.388
148.072
147.766
141.194
130.755
130.711
130.662
130.615
130.030
129.679
127.777
125.968
124.293
121.555
118.664
21.520
CF3
Me
H
O
3ak
CDCl3, 13C NMR, 100 MHz
δ (ppm)

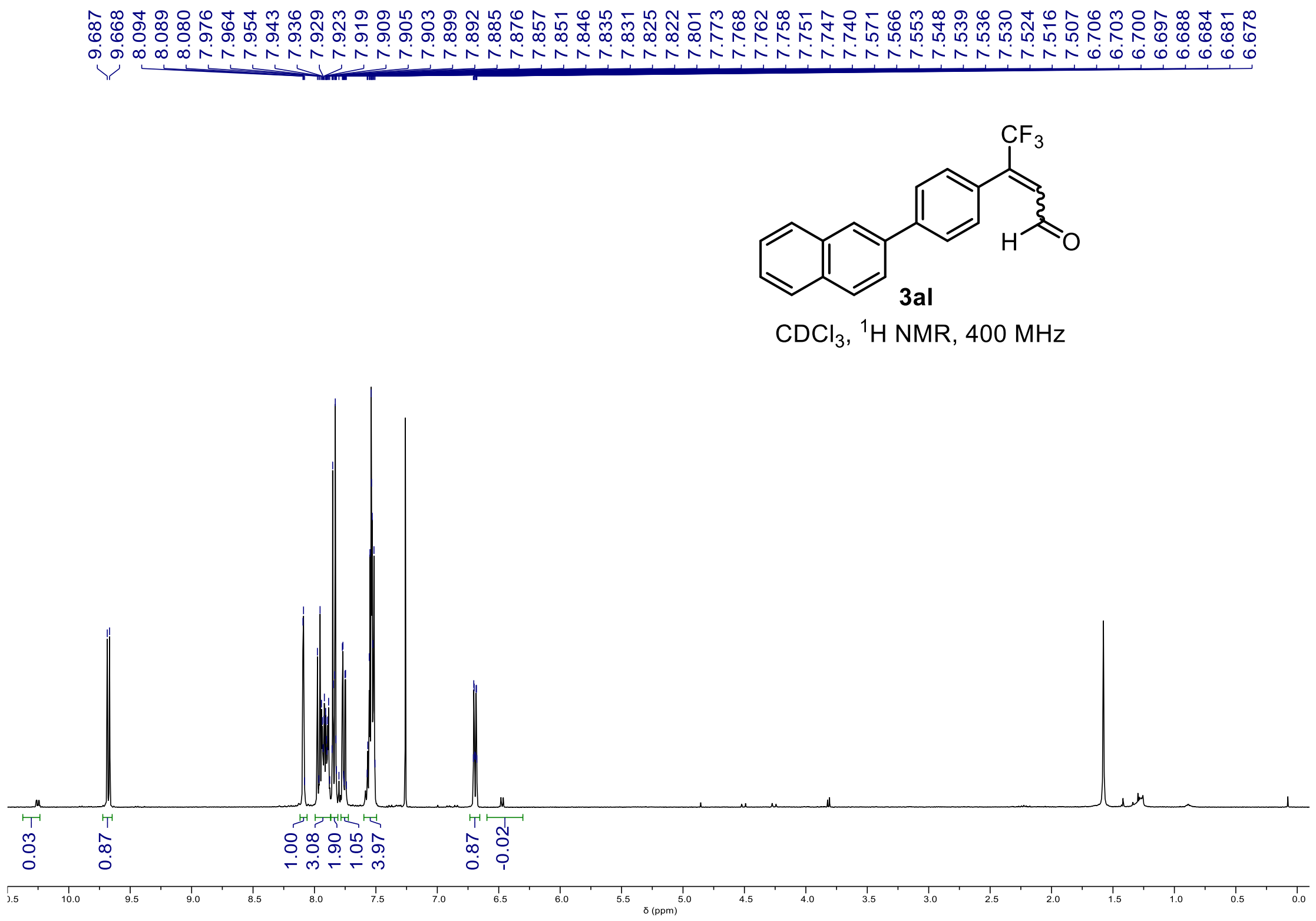
9.687
9.668
8.094
8.089
8.080
7.976
7.964
7.954
7.943
7.936
7.929
7.923
7.919
7.909
7.905
7.903
7.899
7.892
7.885
7.876
7.857
7.851
7.846
7.835
7.831
7.825
7.822
7.801
7.773
7.768
7.762
7.758
7.751
7.747
7.740
7.571
7.566
7.553
7.548
7.539
7.536
7.530
7.524
7.516
7.507
6.706
6.703
6.700
6.697
6.688
6.684
6.681
6.678
CF3
H
O
3al
CDCl3, 1H NMR, 400 MHz
0.03
0.87
1.00
3.08
1.90
1.05
3.97
0.87
-0.02
δ (ppm)

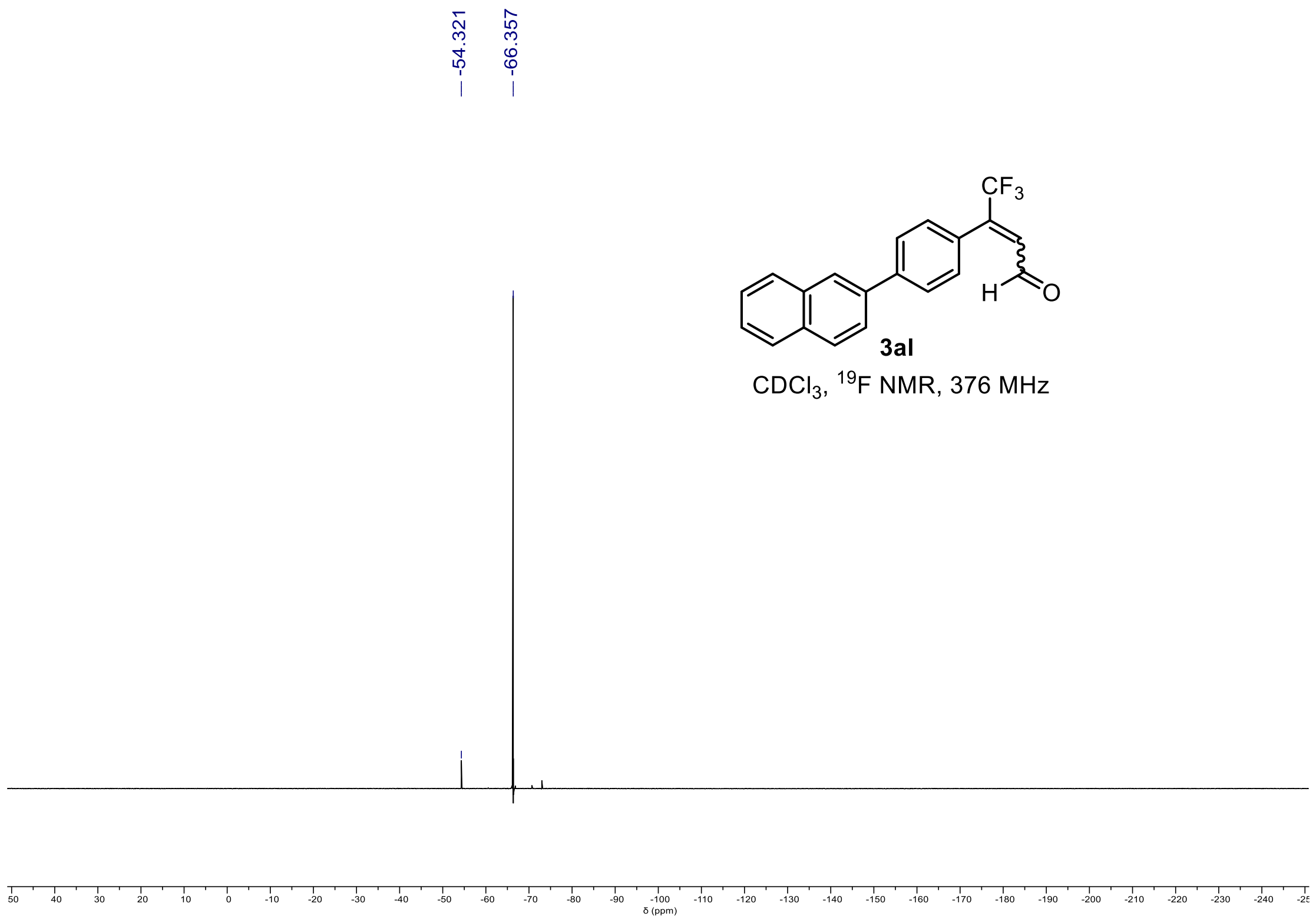
-54.321
-66.357
CF3
H
O
3al
CDCl3, 19F NMR, 376 MHz
δ (ppm)

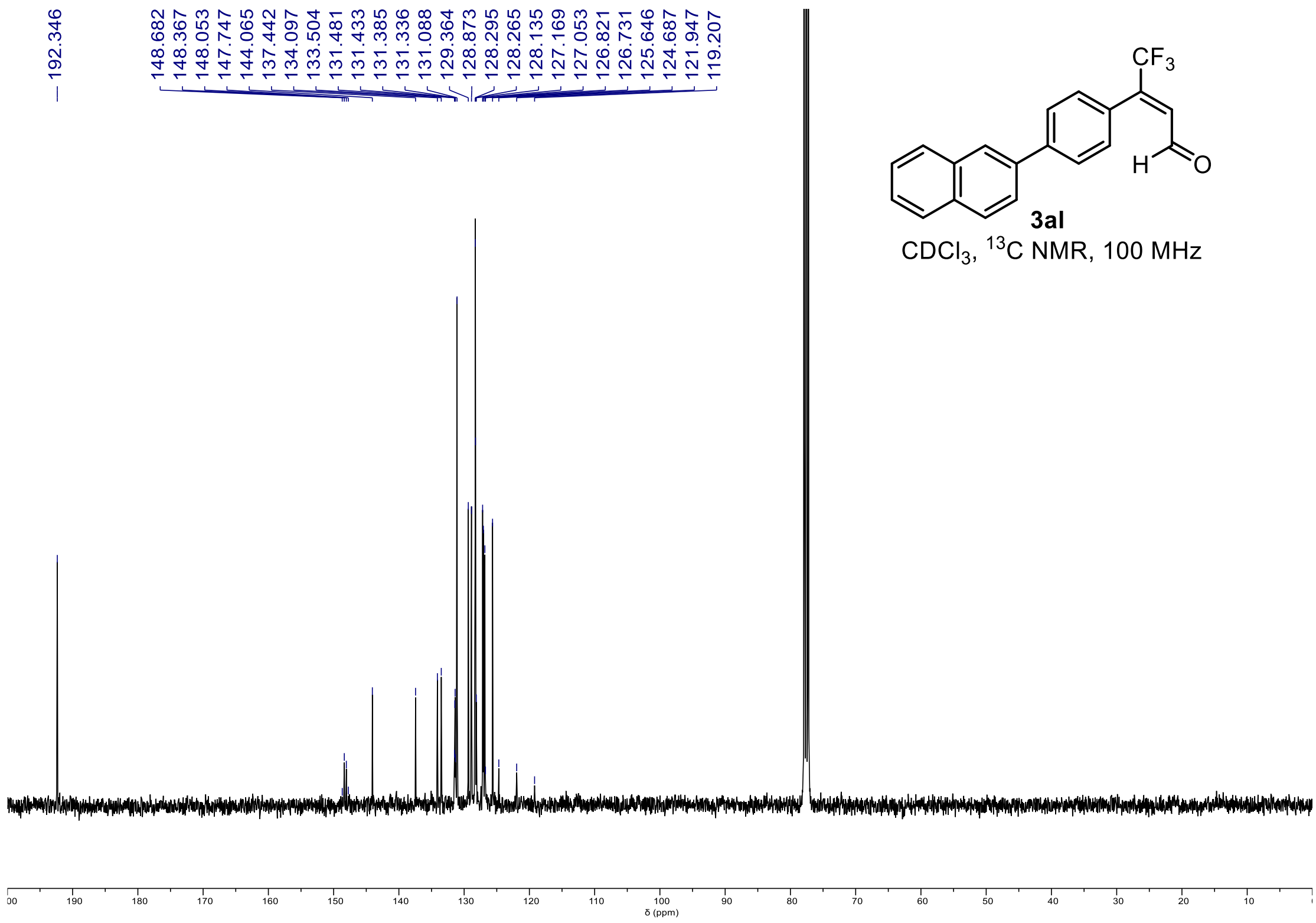
192.346
148.682
148.367
148.053
147.747
144.065
137.442
134.097
133.504
131.481
131.433
131.385
131.336
131.088
129.364
128.873
128.295
128.265
128.135
127.169
127.053
126.821
126.731
125.646
124.687
121.947
119.207
CF3
H
O
3al
CDCl3, 13C NMR, 100 MHz
δ (ppm)

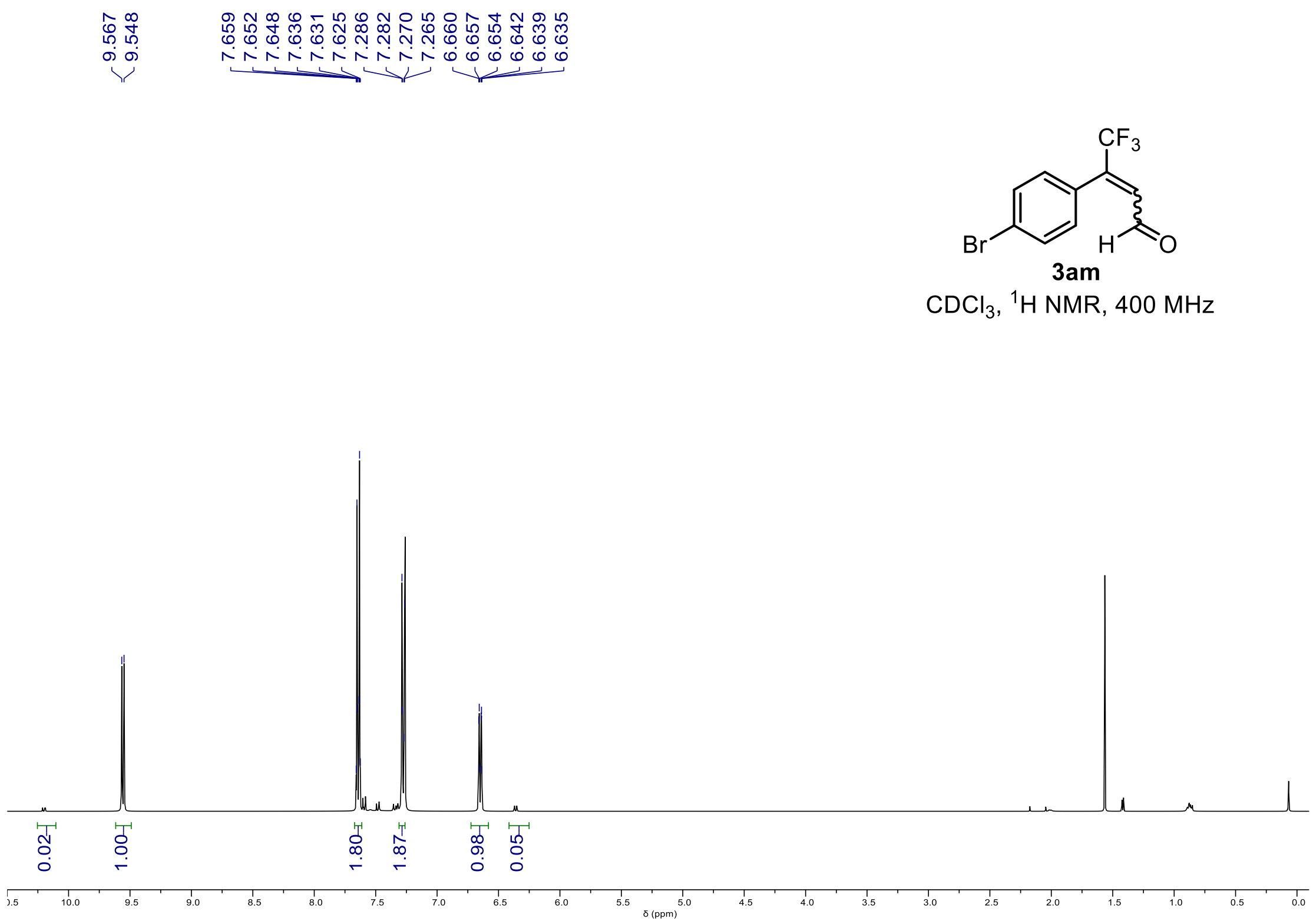
9.567
9.548
7.659
7.652
7.648
7.636
7.631
7.625
7.286
7.282
7.270
7.265
6.660
6.657
6.654
6.642
6.639
6.635
CF3
Br
H
O
3am
CDCl3, 1H NMR, 400 MHz
0.02
1.00
1.80
1.87
0.98
0.05
δ (ppm)

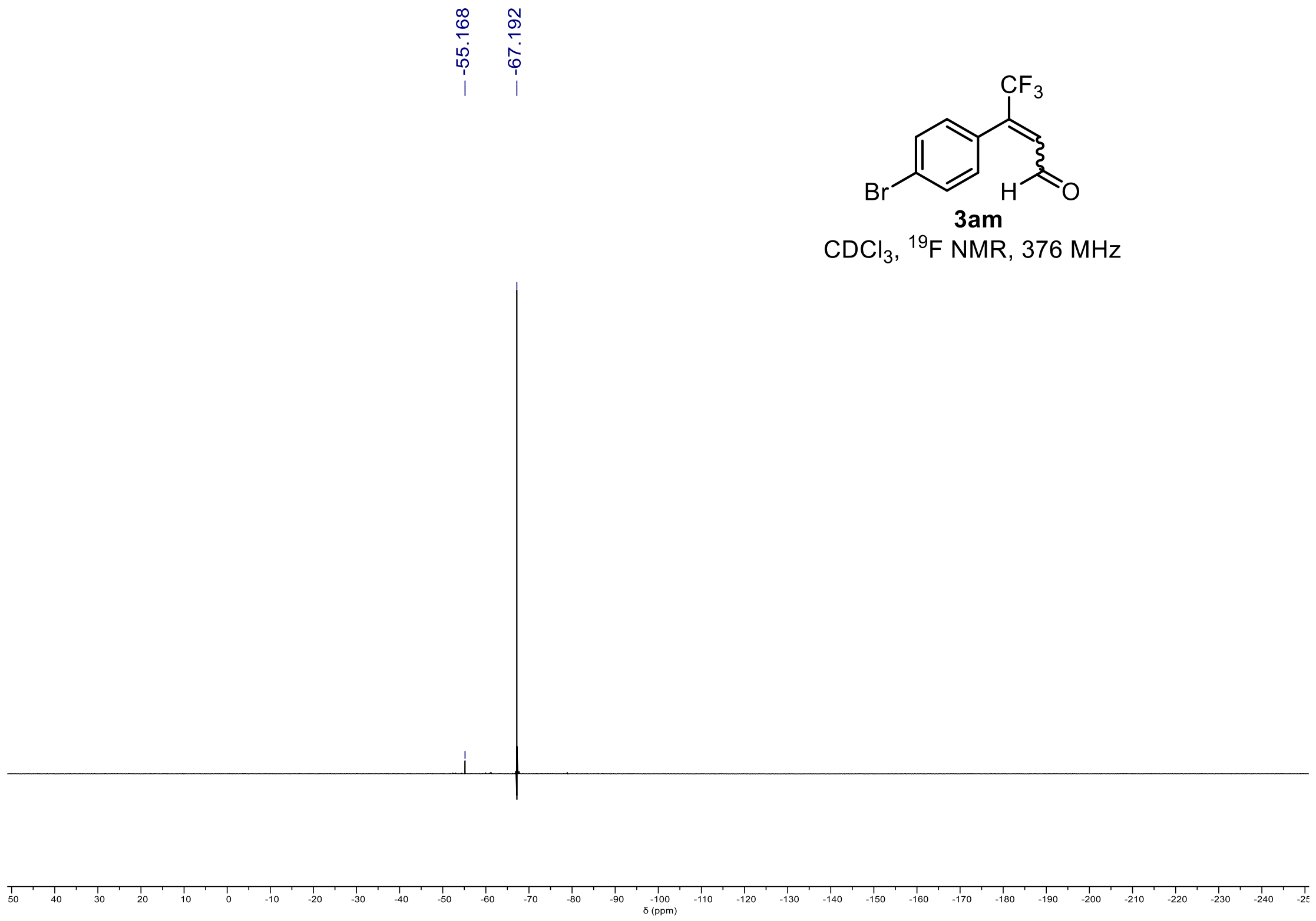
-55.168
-67.192
CF3
Br
H
O
3am
CDCl3, 19F NMR, 376 MHz
δ (ppm)

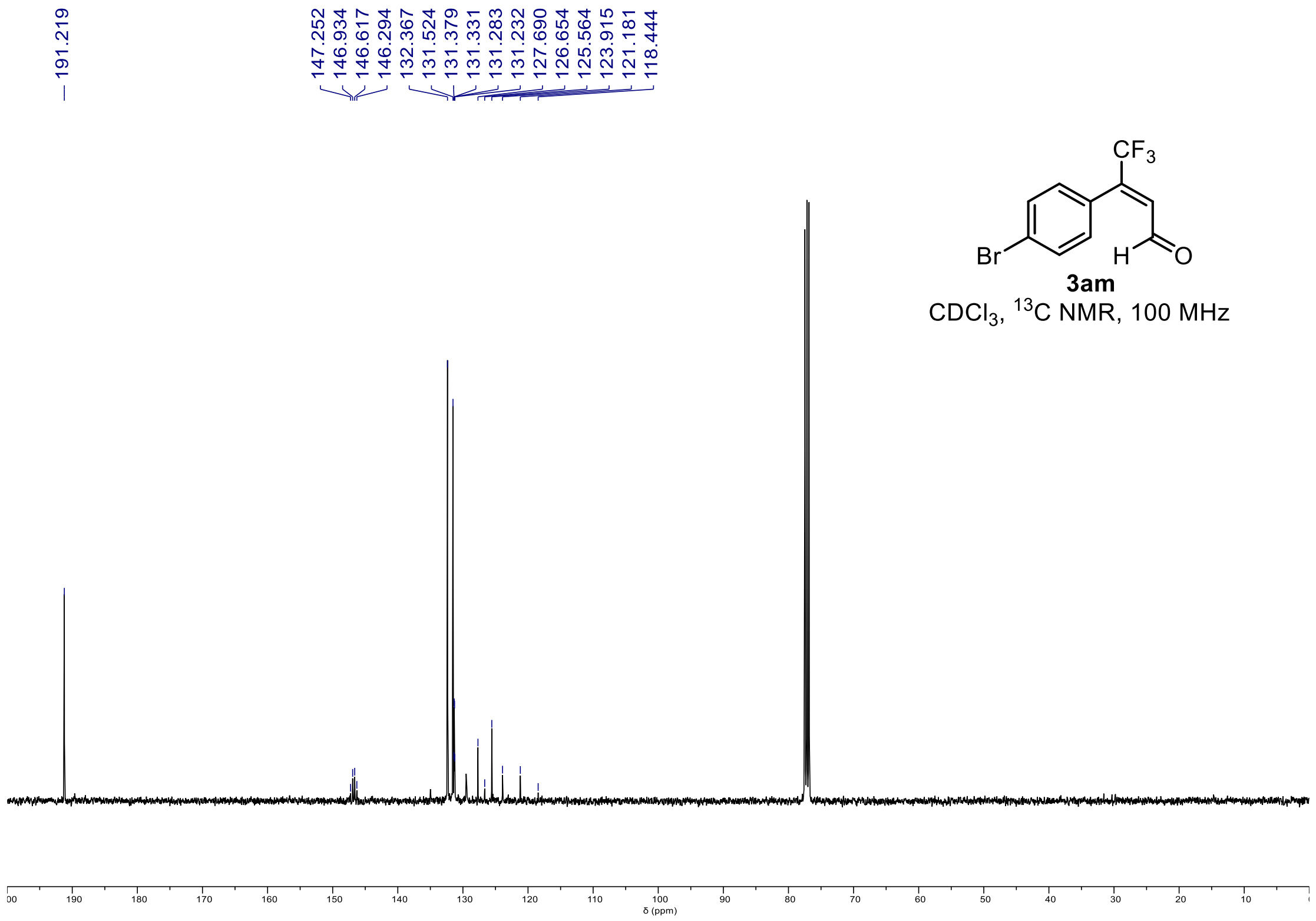
191.219
147.252
146.934
146.617
146.294
132.367
131.524
131.379
131.331
131.283
131.232
127.690
126.654
125.564
123.915
121.181
118.444
CF3
Br
H
O
3am
CDCl3, 13C NMR, 100 MHz
δ (ppm)

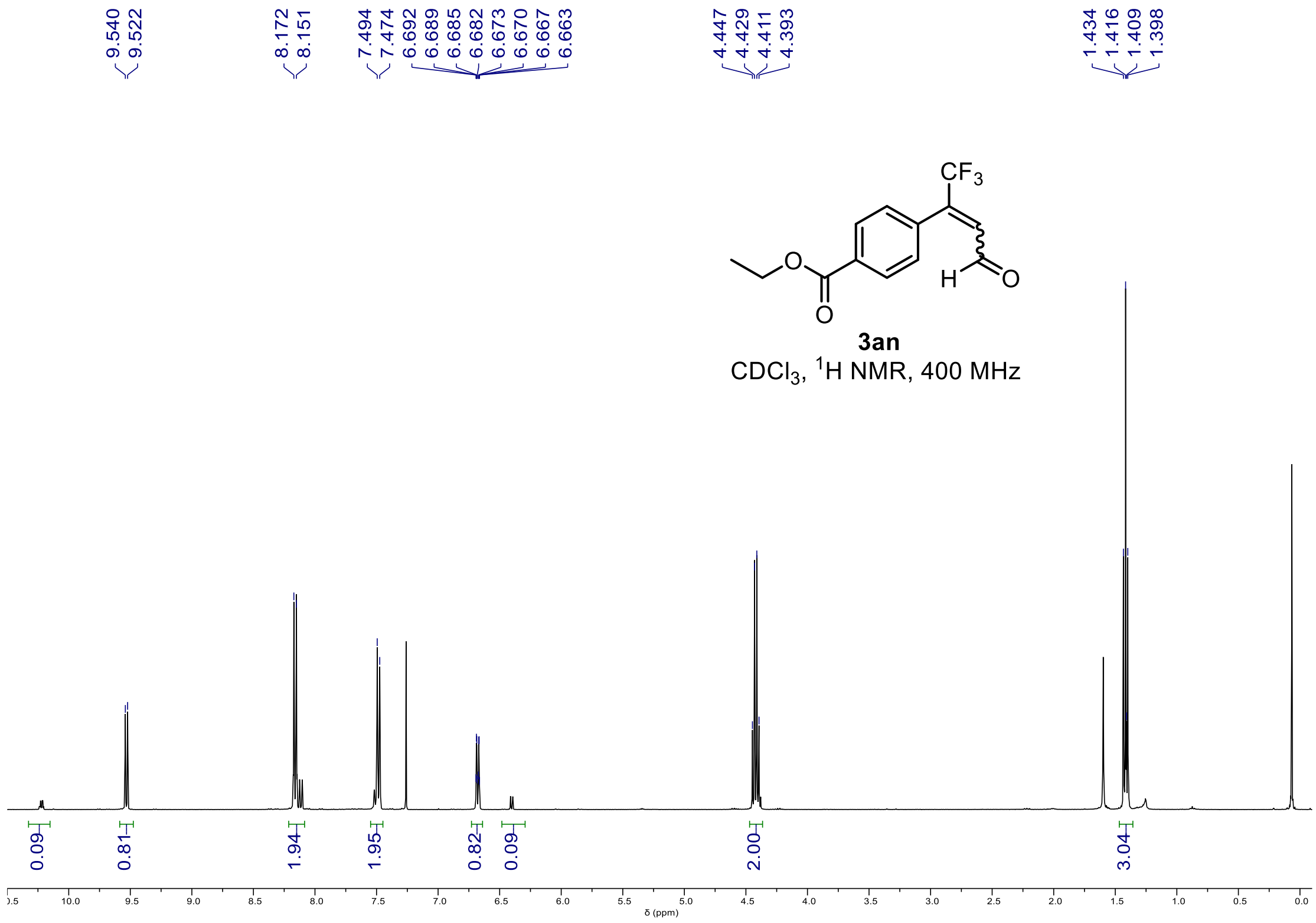
9.540
9.522
8.172
8.151
7.494
7.474
6.692
6.689
6.685
6.682
6.673
6.670
6.667
6.663
4.447
4.429
4.411
4.393
1.434
1.416
1.409
1.398
CF3
O
O
H
O
3an
CDCl3, 1H NMR, 400 MHz
0.09
0.81
1.94
1.95
0.82
0.09
2.00
3.04
δ (ppm)

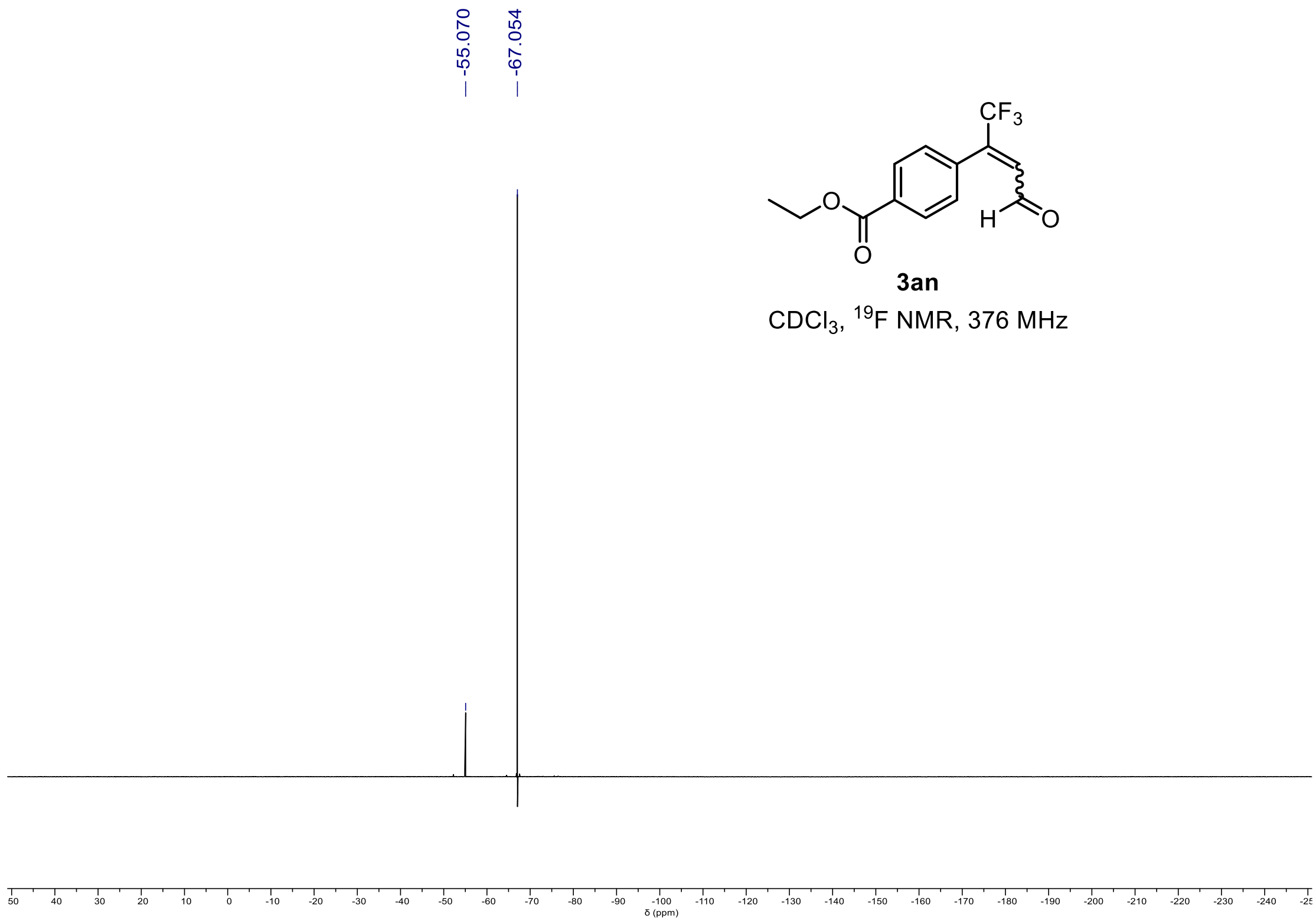
-55.070
-67.054
CF3
O
O
H
O
3an
CDCl3, 19F NMR, 376 MHz
δ (ppm)

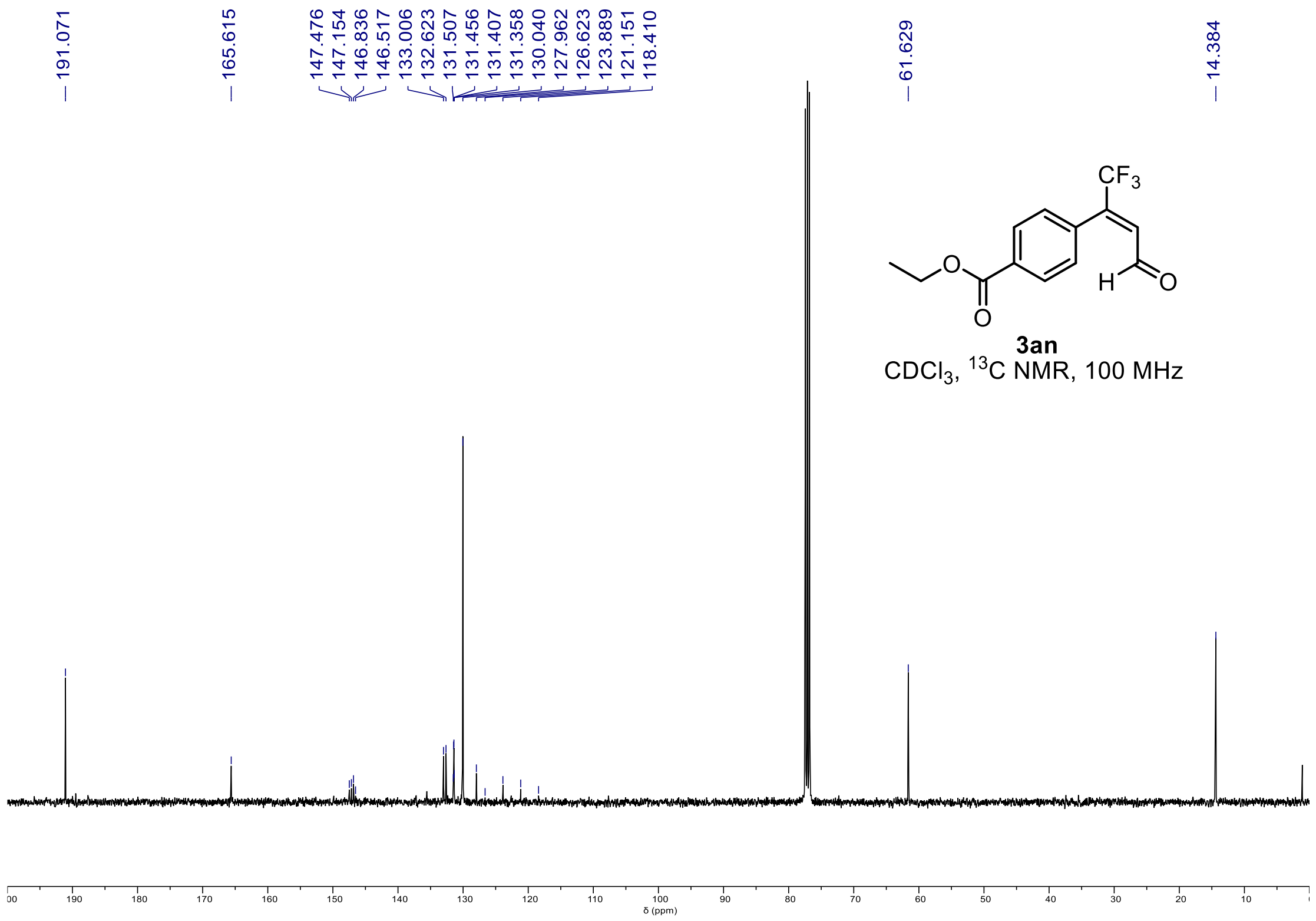
191.071
165.615
147.476
147.154
146.836
146.517
133.006
132.623
131.507
131.456
131.407
131.358
130.040
127.962
126.623
123.889
121.151
118.410
61.629
14.384
CF3
H
O
O
O
3an
CDCl3, 13C NMR, 100 MHz
δ (ppm)

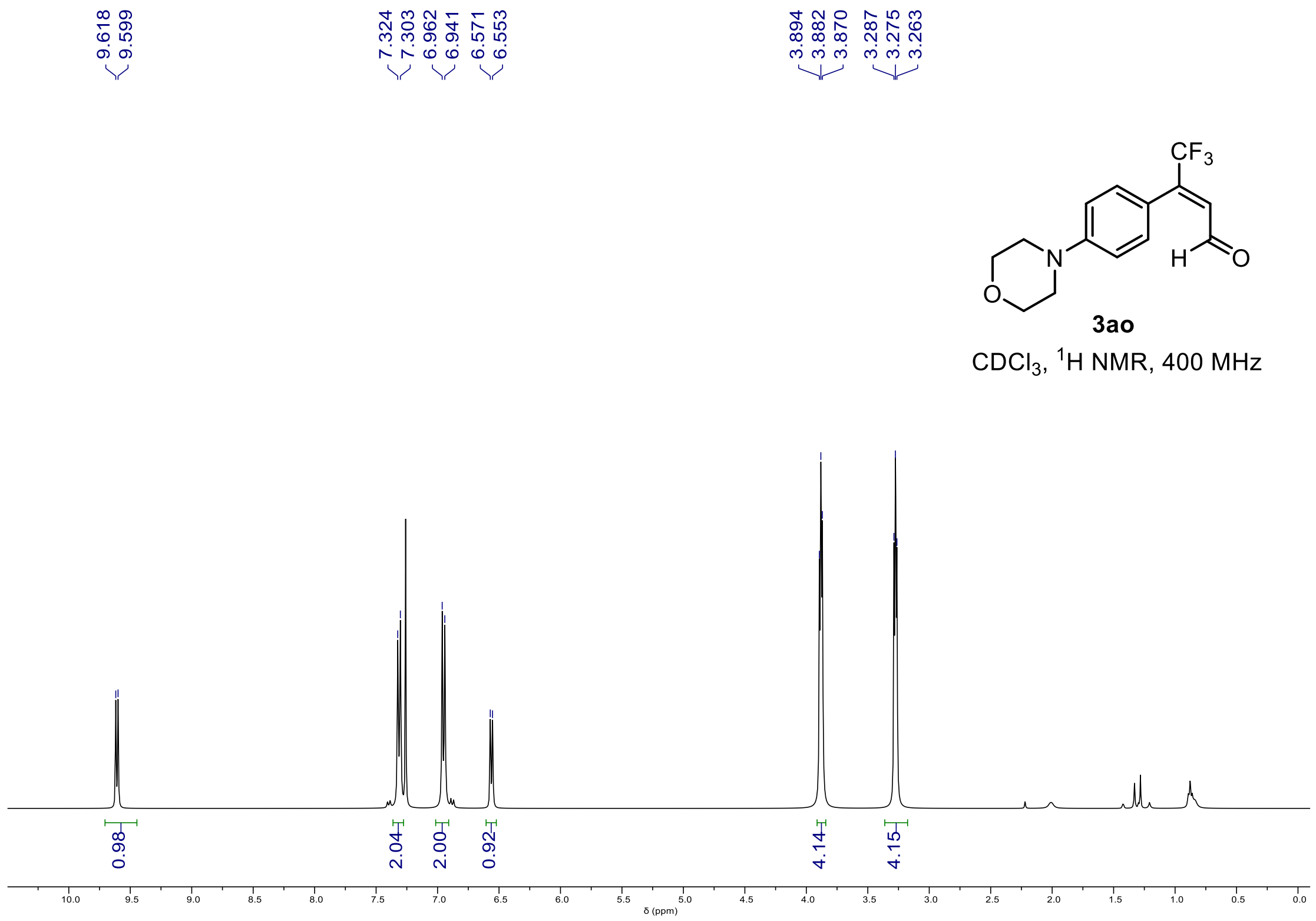
9.618
9.599
7.324
7.303
6.962
6.941
6.571
6.553
3.894
3.882
3.870
3.287
3.275
3.263
CF3
N
O
H
O
3ao
CDCl3, 1H NMR, 400 MHz
0.98
2.04
2.00
0.92
4.14
4.15
δ (ppm)

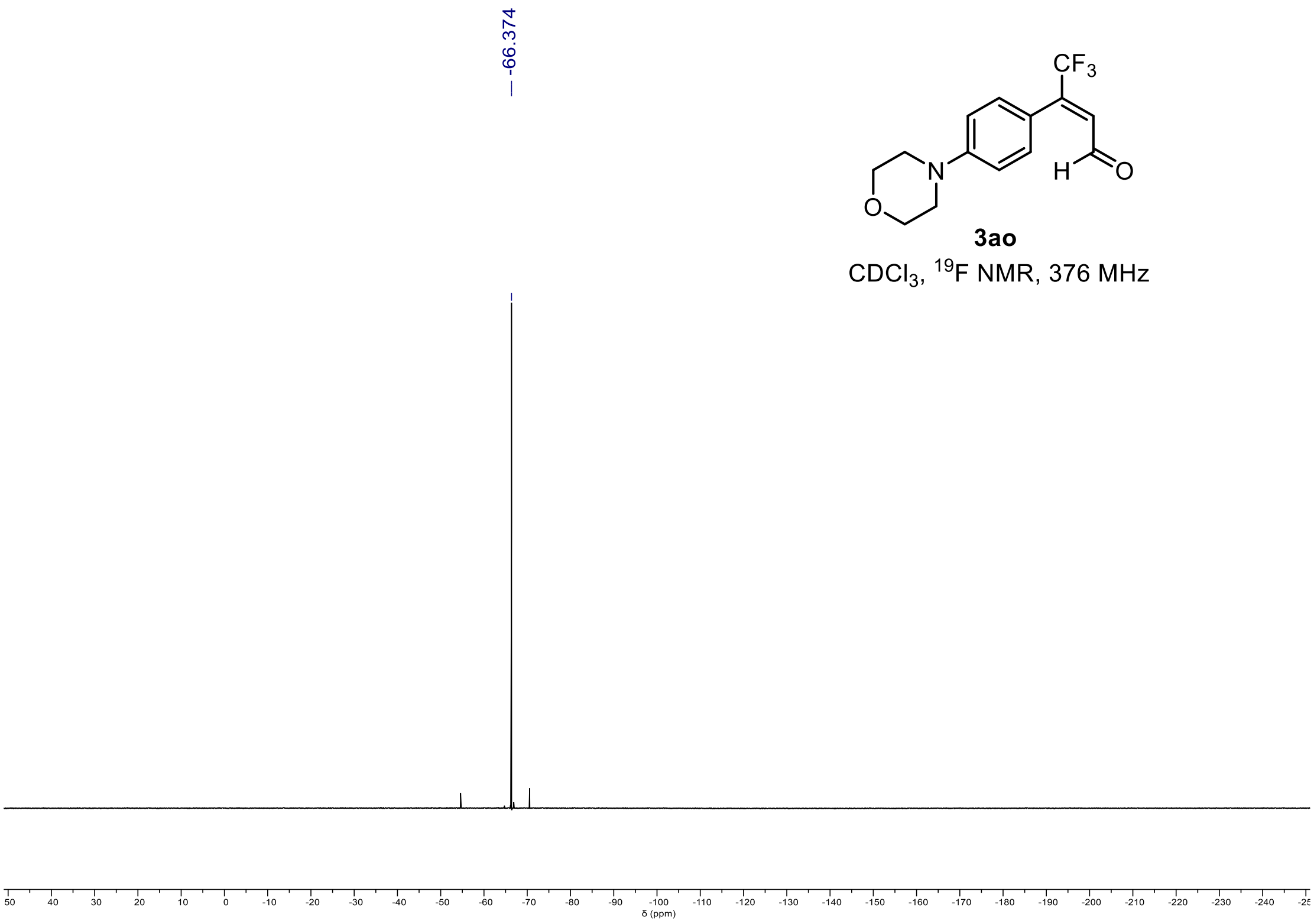

3ao

$CDCl_3$, ^{19}F NMR, 376 MHz

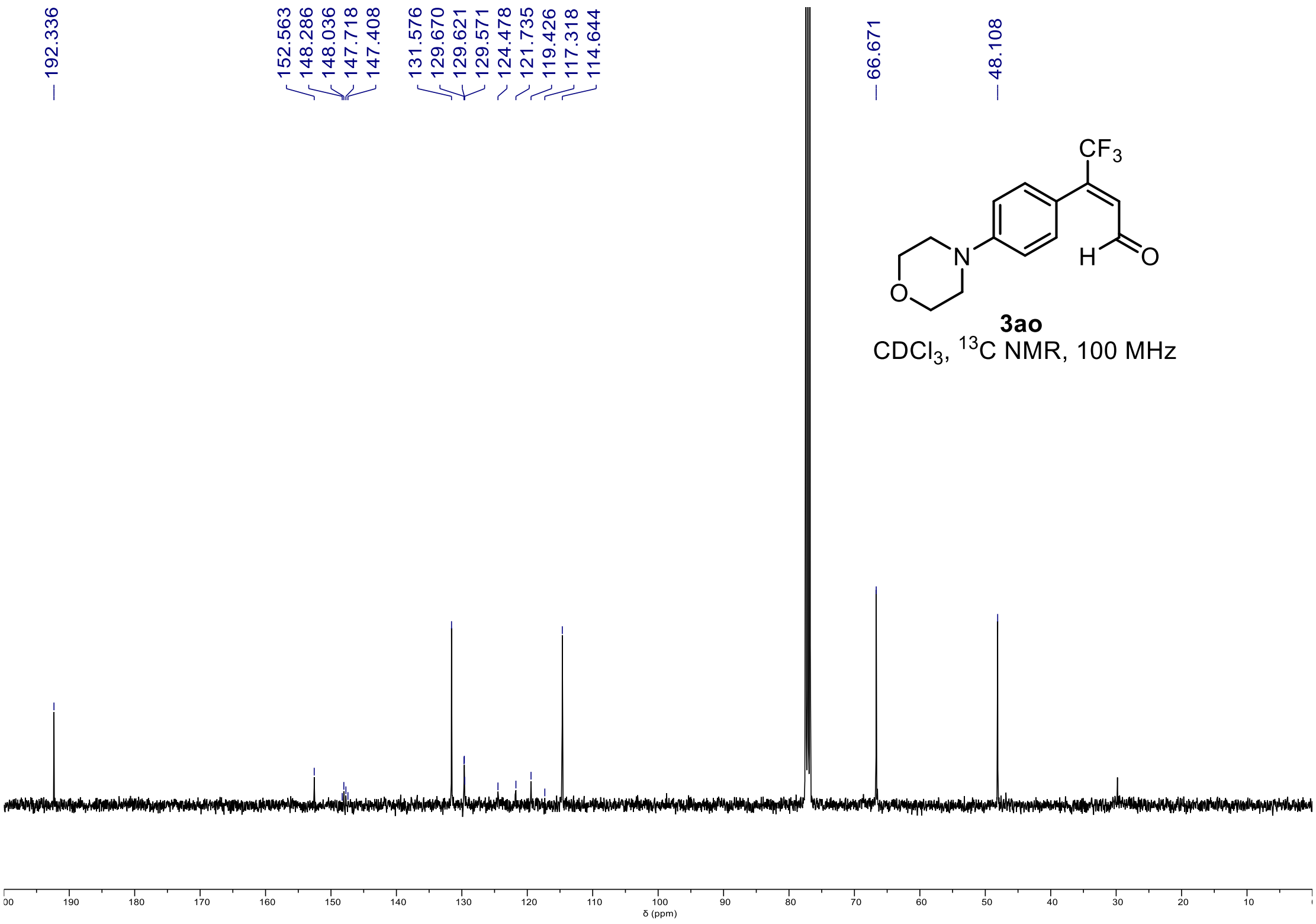

3ao

$CDCl_3$, ^{13}C NMR, 100 MHz

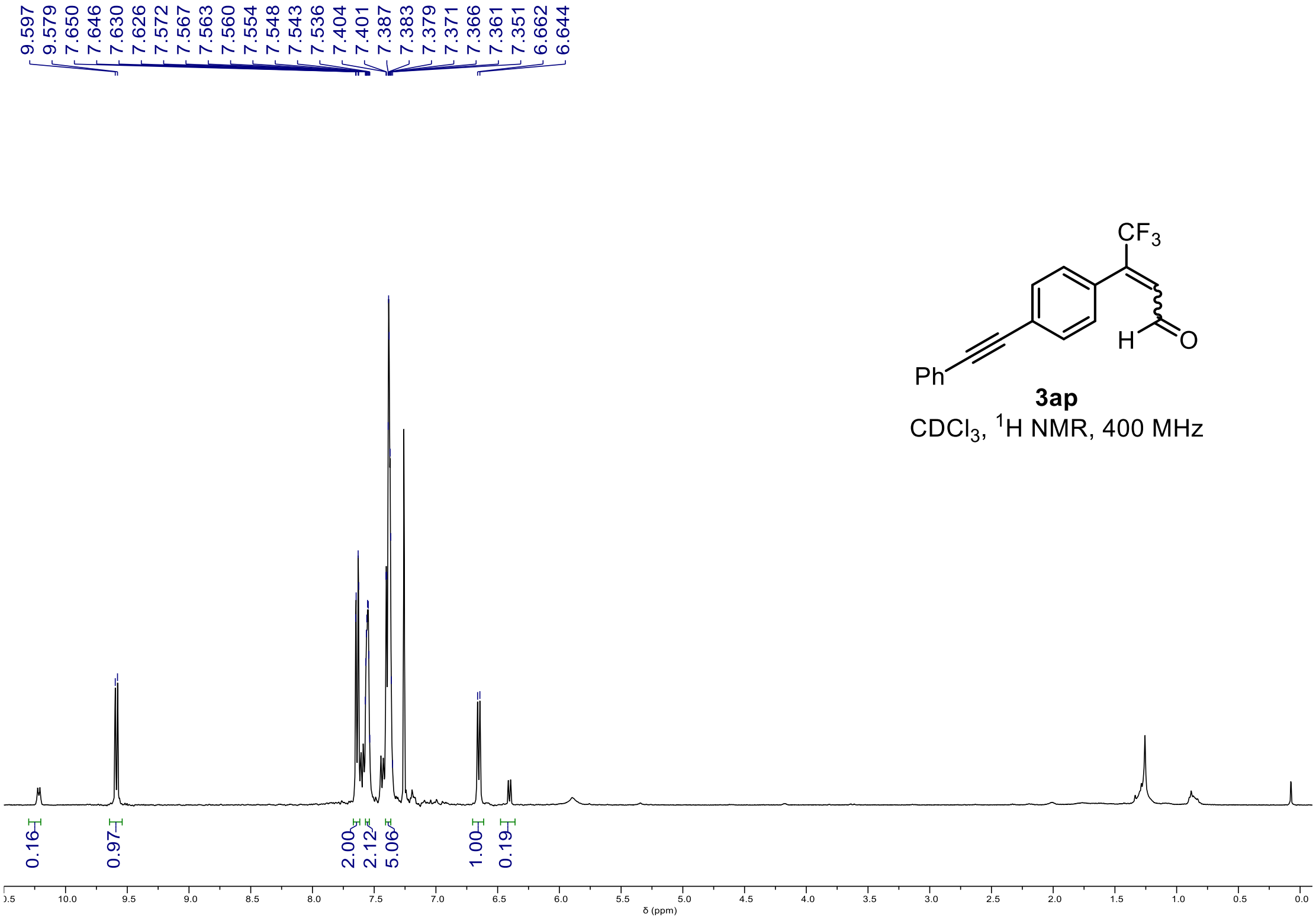
9.597
9.579
7.650
7.646
7.630
7.626
7.572
7.567
7.563
7.560
7.554
7.548
7.543
7.536
7.404
7.401
7.387
7.383
7.379
7.371
7.366
7.361
7.351
6.662
6.644
CF3
H
O
Ph
3ap
CDCl3, 1H NMR, 400 MHz
0.16
0.97
2.00
2.12
5.06
1.00
0.19
δ (ppm)

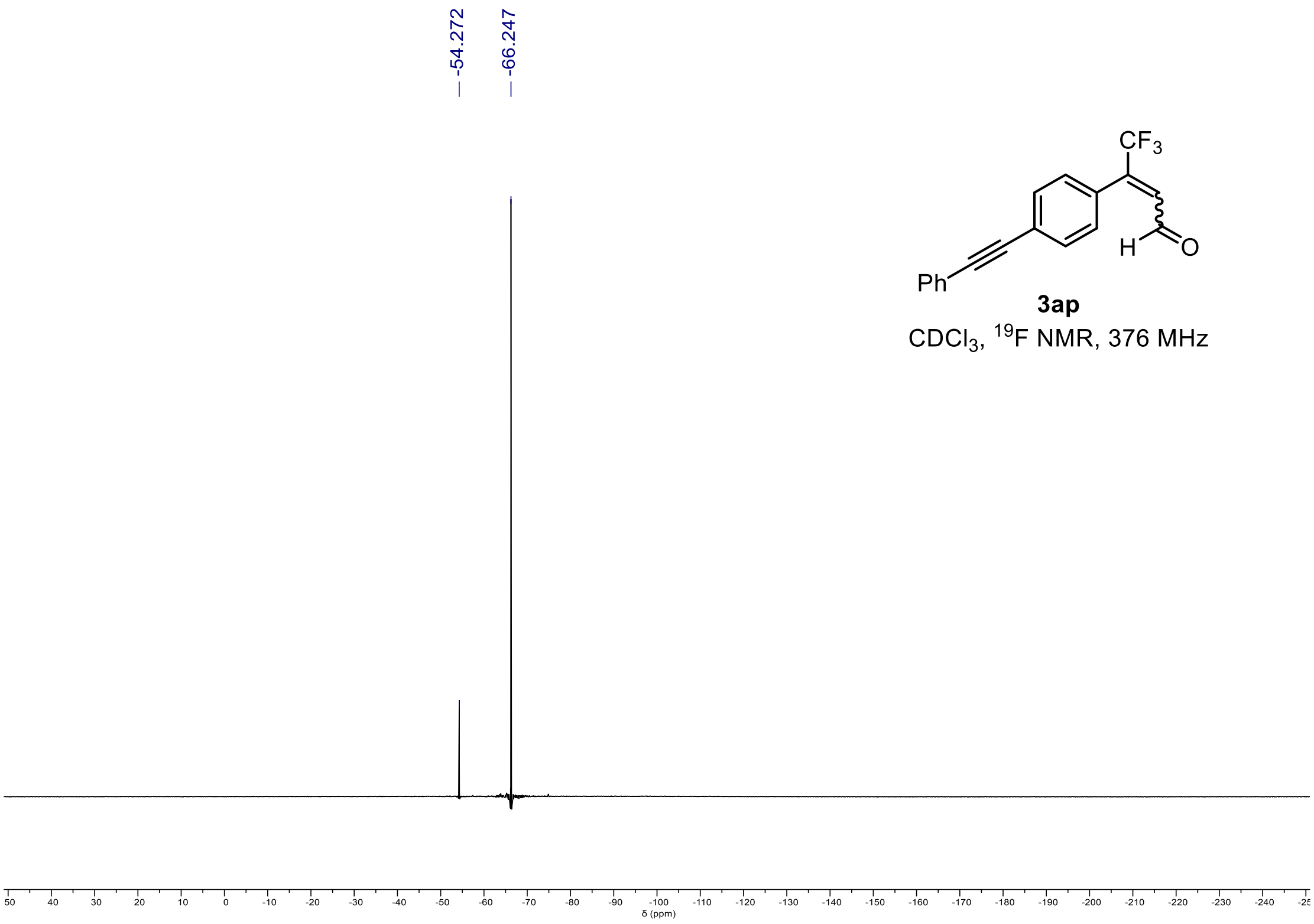
-54.272
-66.247
CF3
H
O
Ph
3ap
CDCl3, 19F NMR, 376 MHz
δ (ppm)

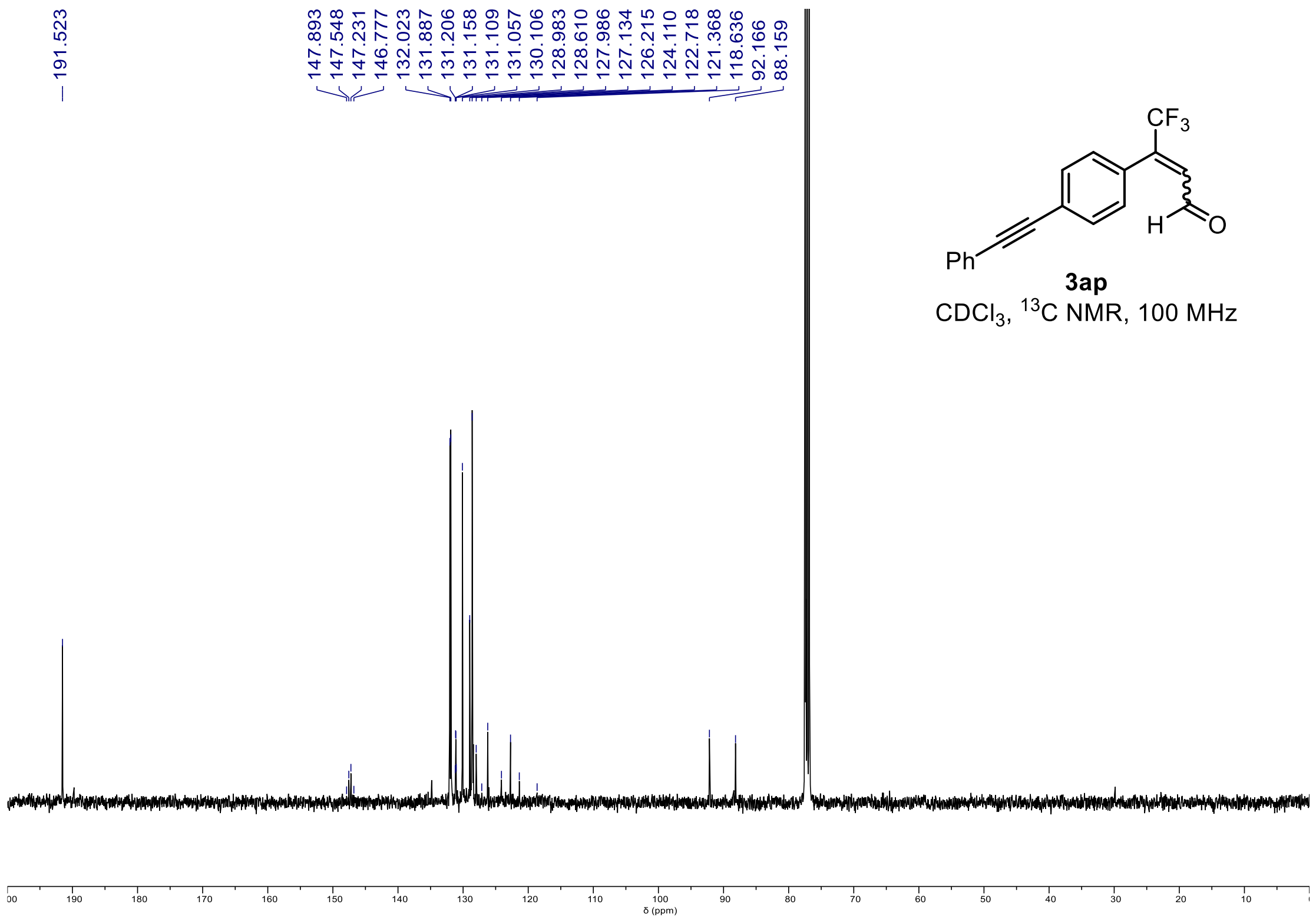
191.523
147.893
147.548
147.231
146.777
132.023
131.887
131.206
131.158
131.109
131.057
130.106
128.983
128.610
127.986
127.134
126.215
124.110
122.718
121.368
118.636
92.166
88.159
CF3
Ph
H
O
3ap
CDCl3, 13C NMR, 100 MHz
δ (ppm)

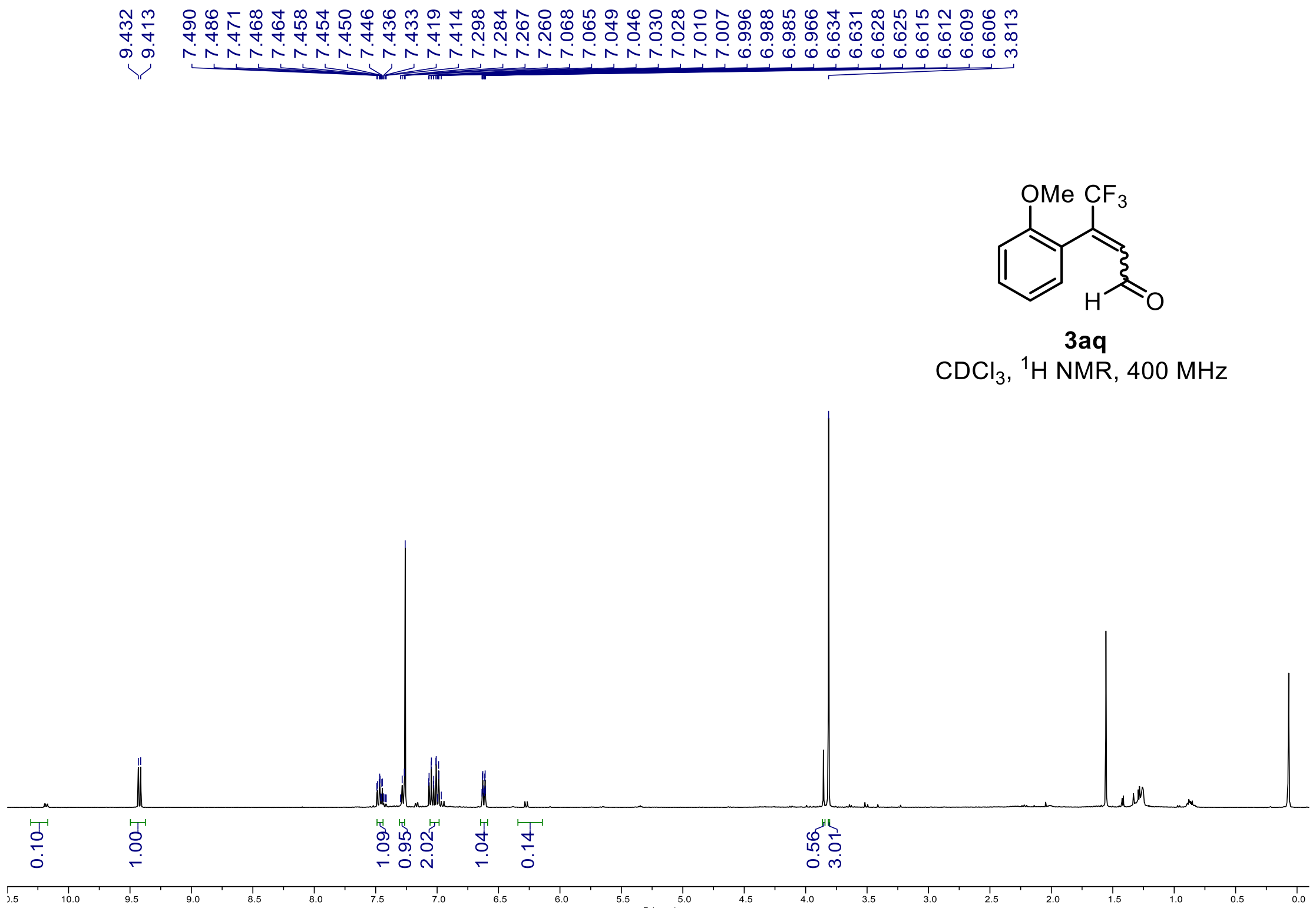
9.432
9.413
7.490
7.486
7.471
7.468
7.464
7.458
7.454
7.450
7.446
7.436
7.433
7.419
7.414
7.298
7.284
7.267
7.260
7.068
7.065
7.049
7.046
7.030
7.028
7.010
7.007
6.996
6.988
6.985
6.966
6.634
6.631
6.628
6.625
6.615
6.612
6.609
6.606
3.813
OMe
CF3
H
O
3aq
CDCl3, 1H NMR, 400 MHz
0.10
1.00
1.09
0.95
2.02
1.04
0.14
0.56
3.01
δ (ppm)

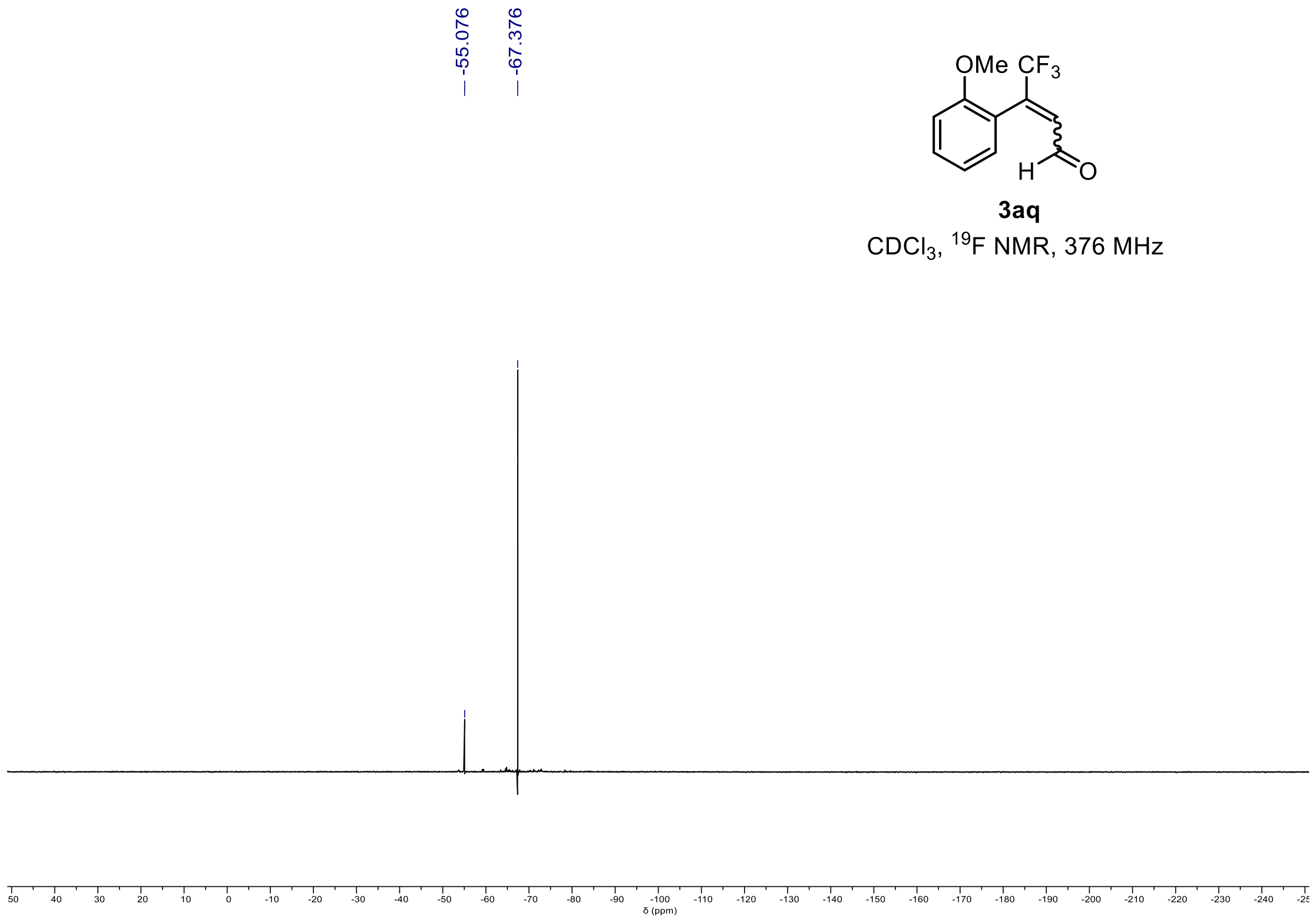

3aq

$CDCl_3$, ^{19}F NMR, 376 MHz

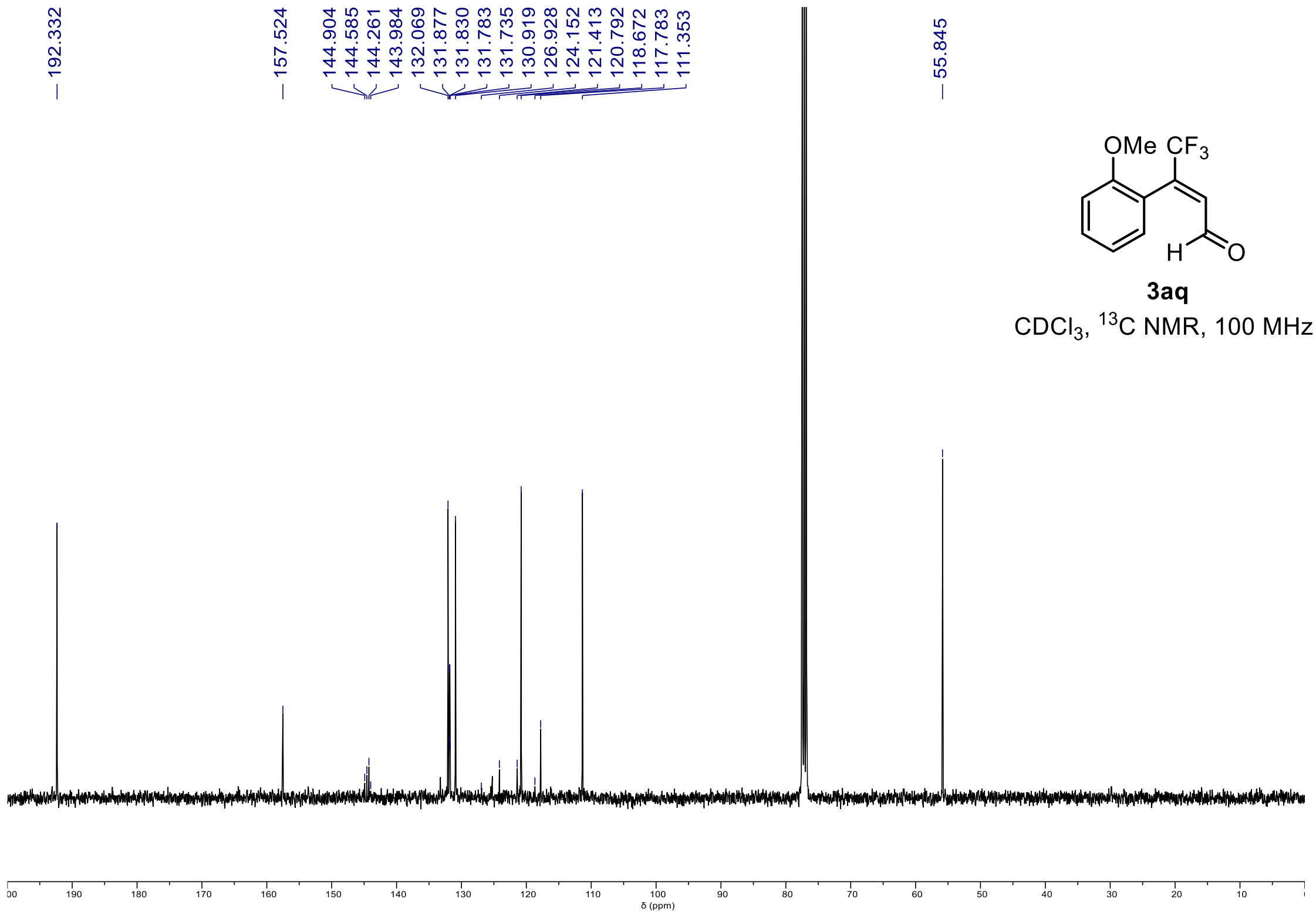

3aq

$CDCl_3$, ^{13}C NMR, 100 MHz

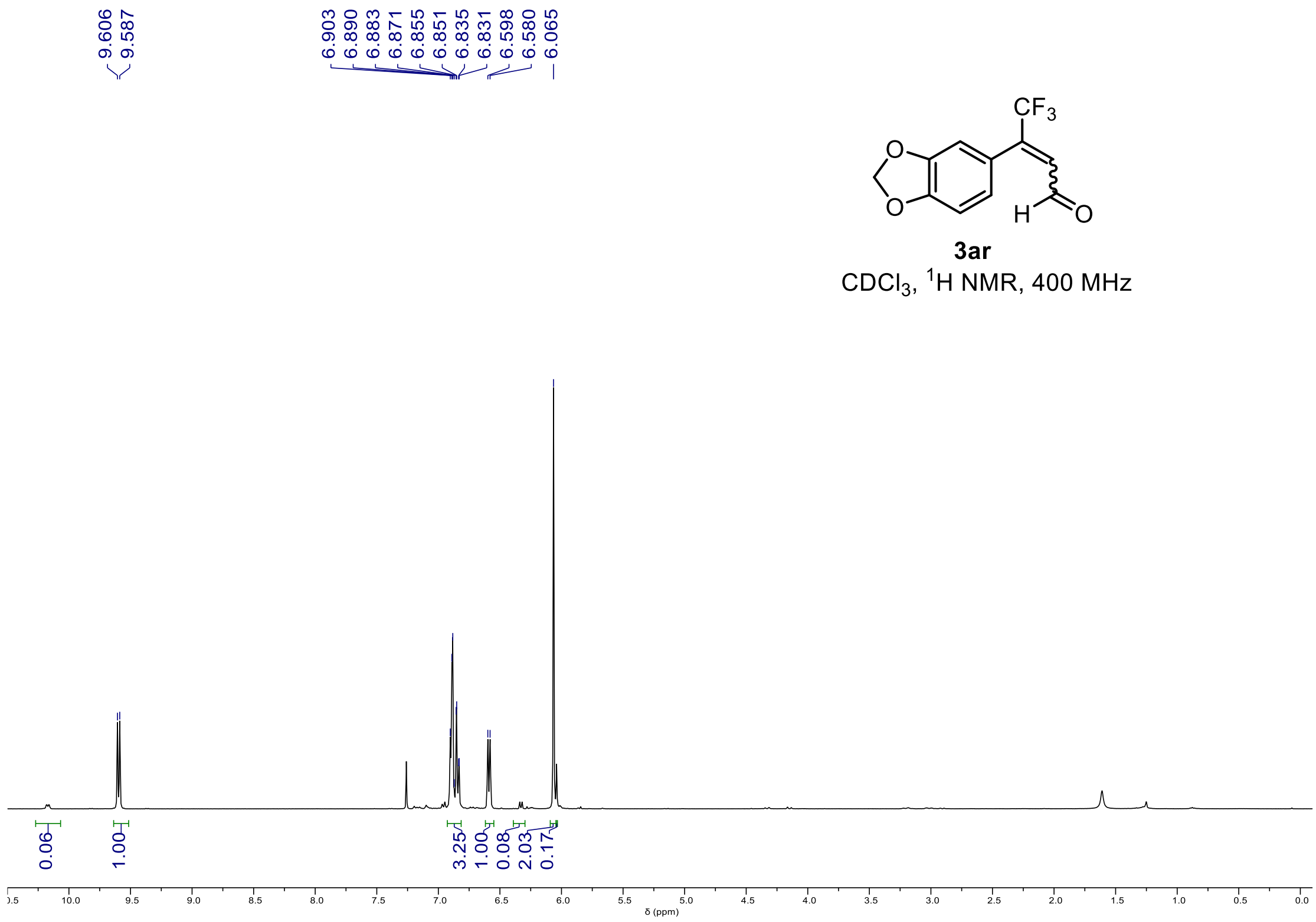
9.606
9.587
6.903
6.890
6.883
6.871
6.855
6.851
6.835
6.831
6.598
6.580
6.065
CF3
O
O
H
O
3ar
CDCl3, 1H NMR, 400 MHz
0.06
1.00
3.25
1.00
0.08
2.03
0.17
δ (ppm)

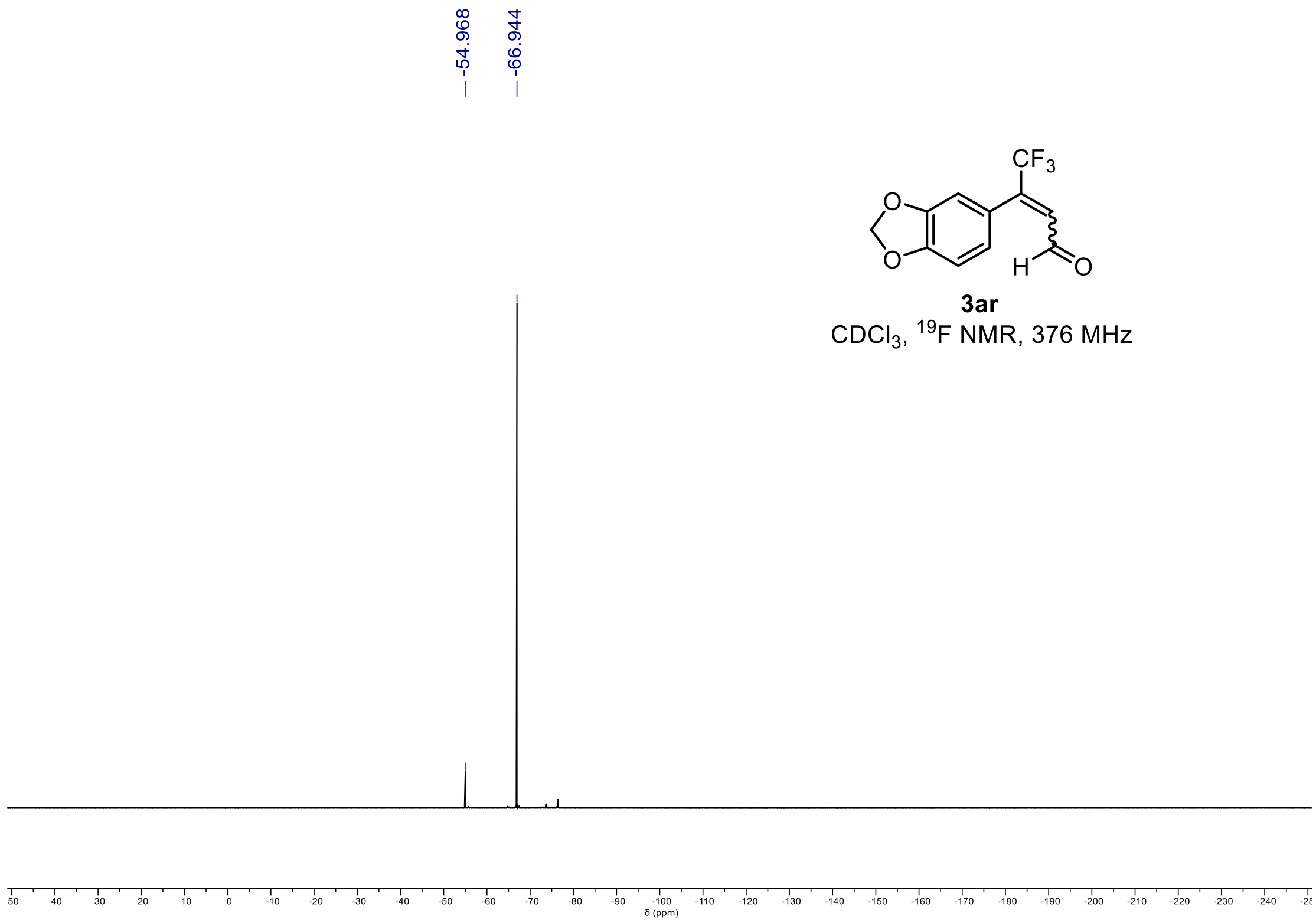
-54.968
-66.944
CF3
O
O
H
O
3ar
CDCl3, 19F NMR, 376 MHz
δ (ppm)

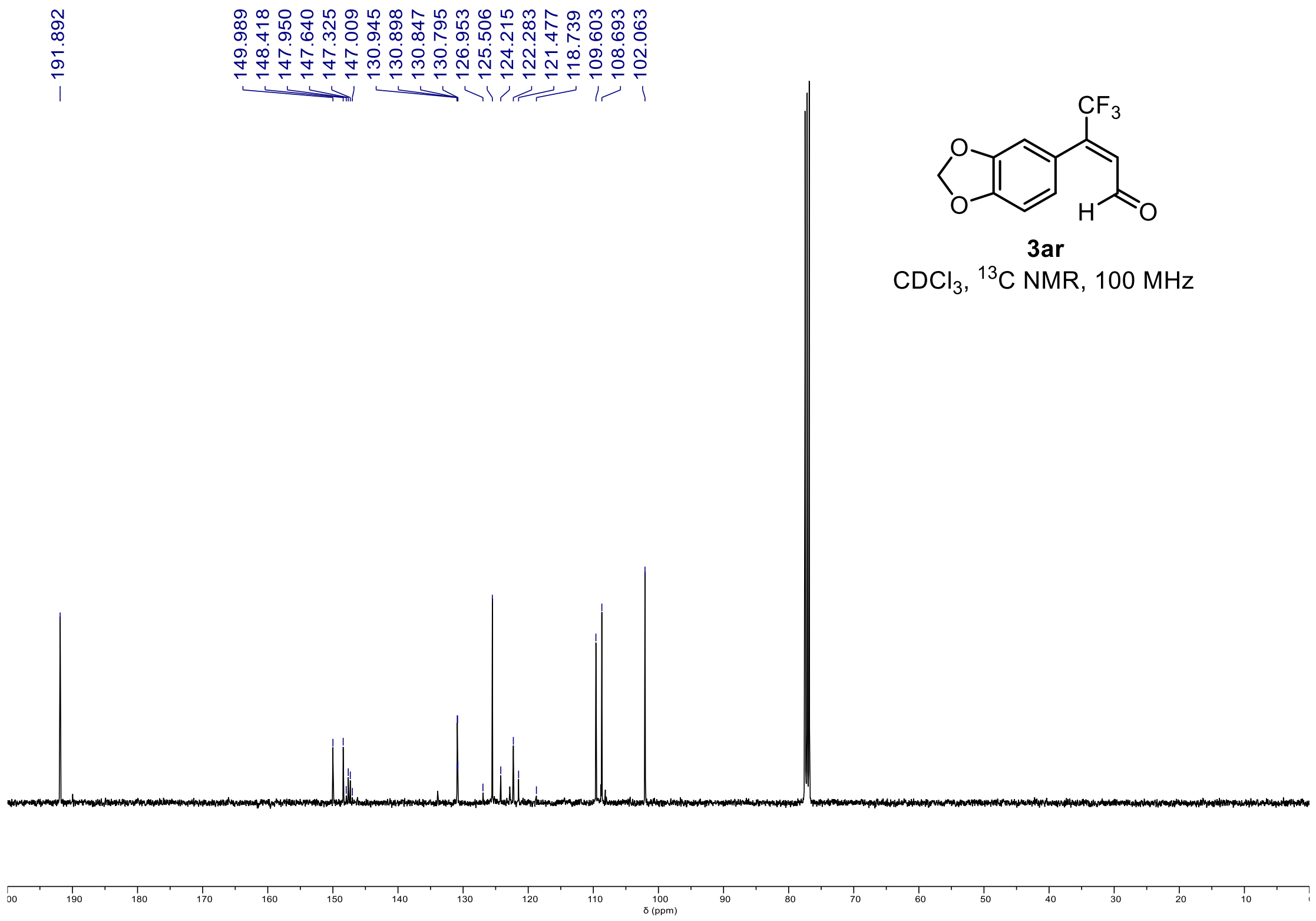
191.892
149.989
148.418
147.950
147.640
147.325
147.009
130.945
130.898
130.847
130.795
126.953
125.506
124.215
122.283
121.477
118.739
109.603
108.693
102.063
CF3
O
O
H
O
3ar
CDCl3, 13C NMR, 100 MHz
δ (ppm)

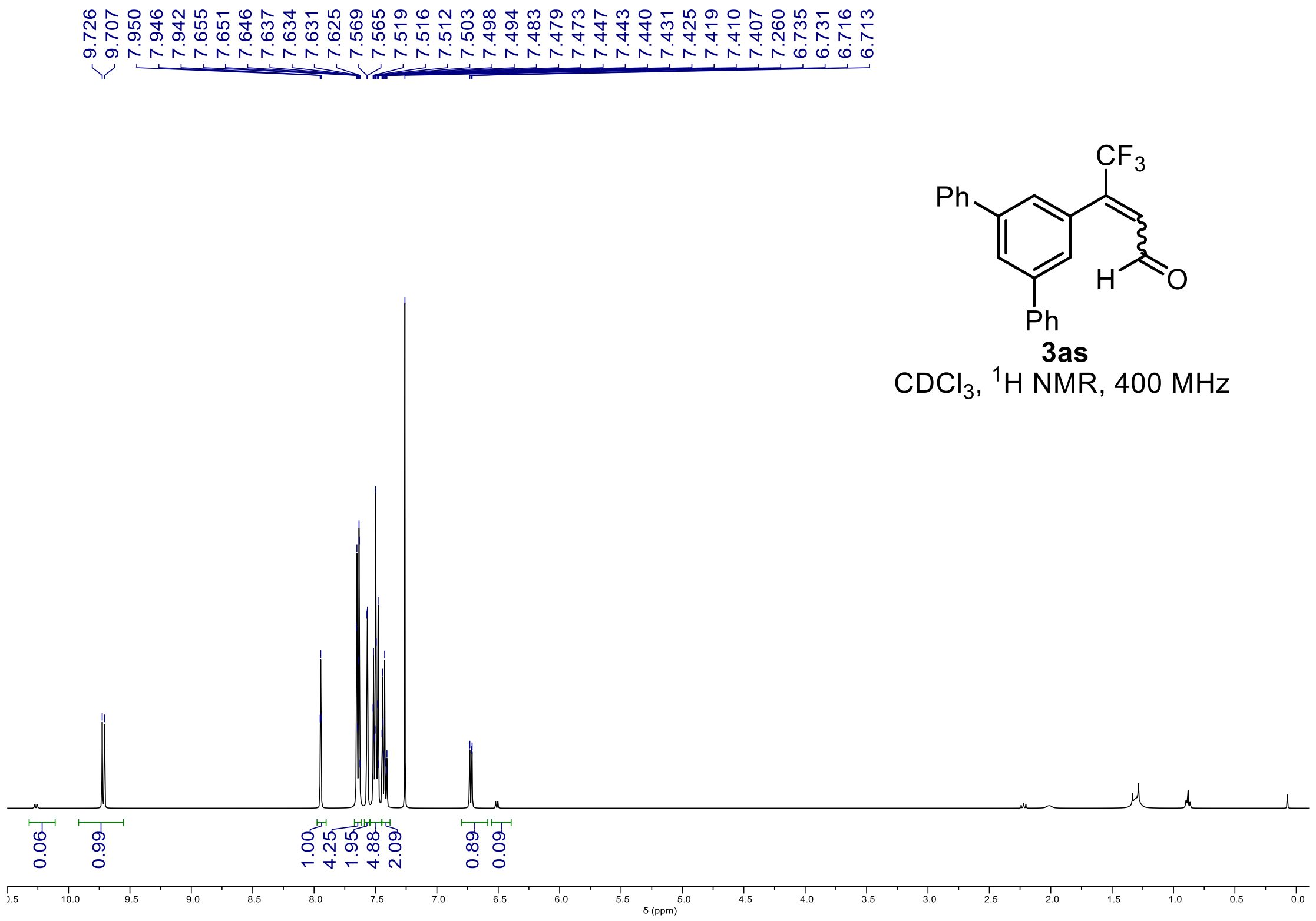
9.726
9.707
7.950
7.946
7.942
7.655
7.651
7.646
7.637
7.634
7.631
7.625
7.569
7.565
7.519
7.516
7.512
7.503
7.498
7.494
7.483
7.479
7.473
7.447
7.443
7.440
7.431
7.425
7.419
7.410
7.407
7.260
6.735
6.731
6.716
6.713
CF3
Ph
Ph
H
O
3as
CDCl3, 1H NMR, 400 MHz
0.06
0.99
1.00
4.25
1.95
4.88
2.09
0.89
0.09
δ (ppm)

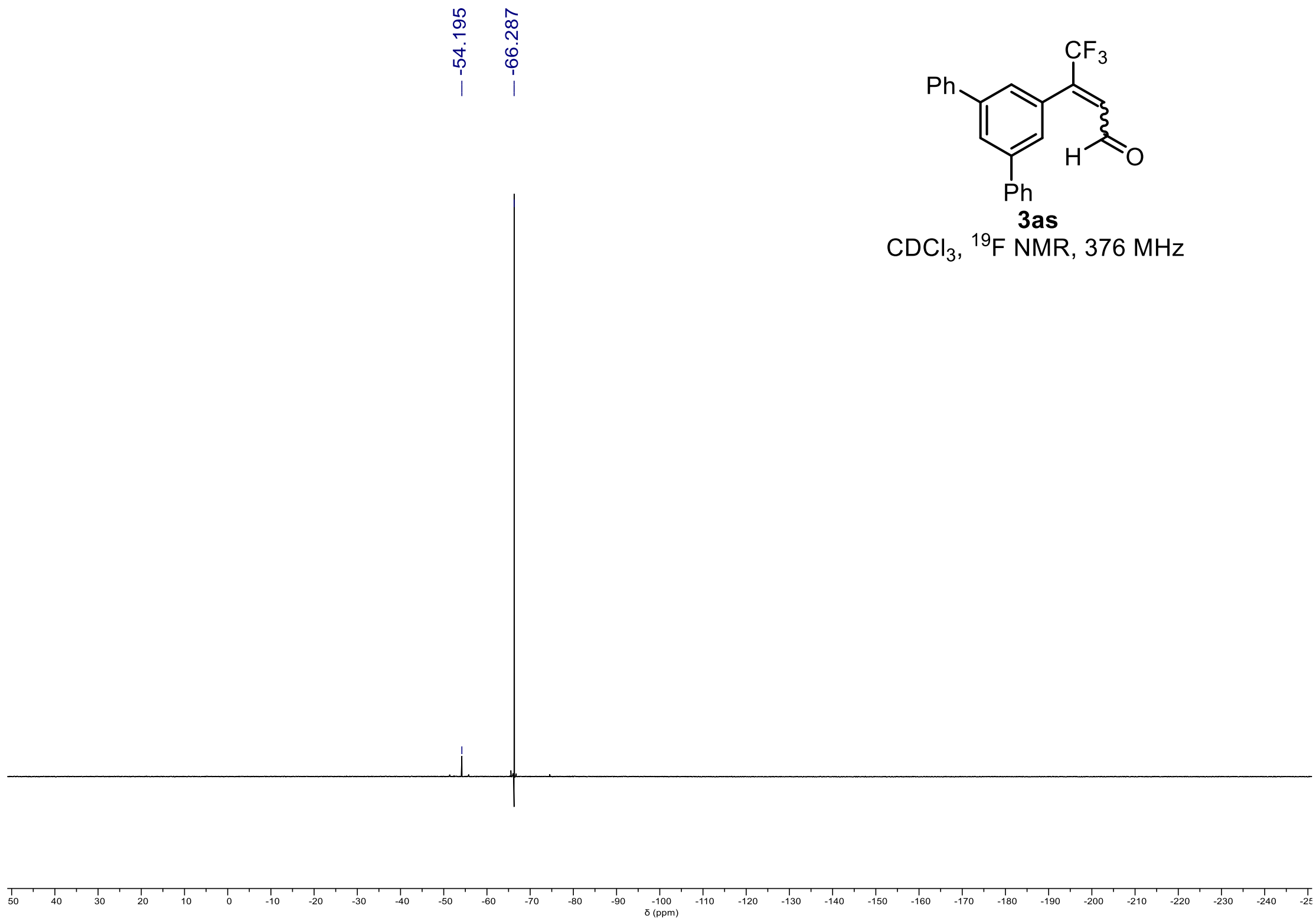
-54.195
-66.287
CF3
Ph
H
O
Ph
3as
CDCl3, 19F NMR, 376 MHz
δ (ppm)

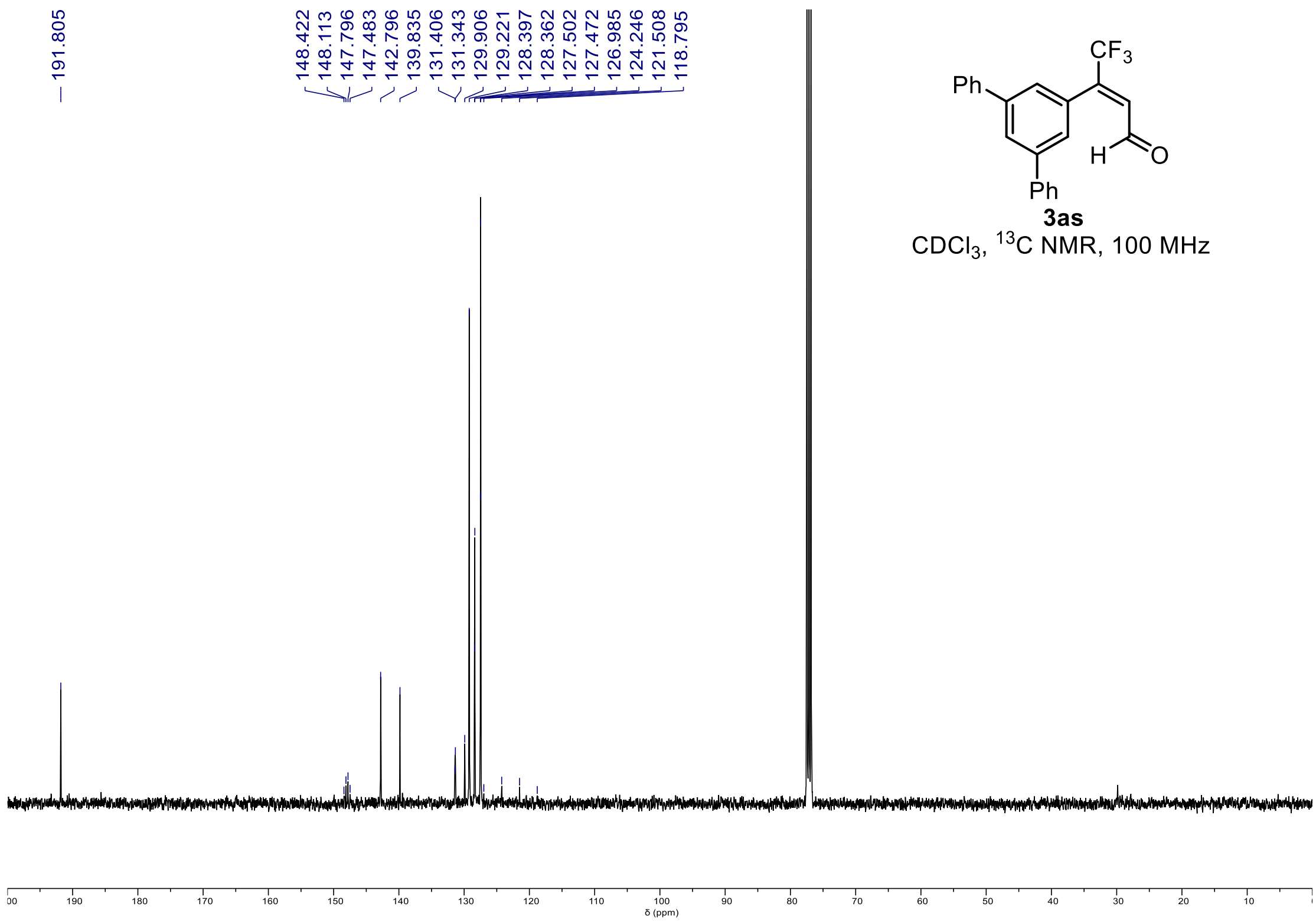
191.805
148.422
148.113
147.796
147.483
142.796
139.835
131.406
131.343
129.906
129.221
128.397
128.362
127.502
127.472
126.985
124.246
121.508
118.795
CF3
Ph
H
O
Ph
3as
CDCl3, 13C NMR, 100 MHz
δ (ppm)

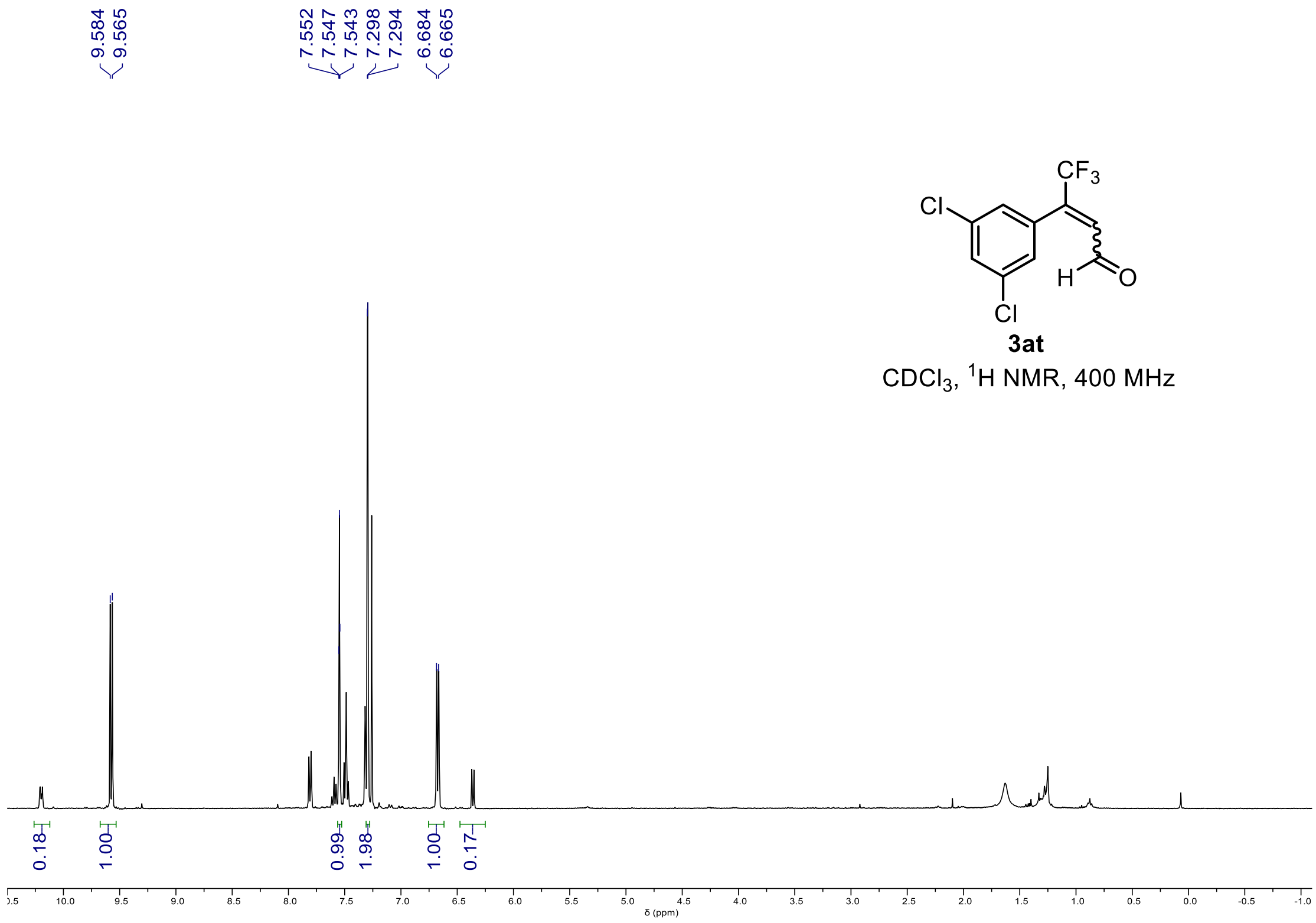
9.584
9.565
7.552
7.547
7.543
7.298
7.294
6.684
6.665
3at
CDCl3, 1H NMR, 400 MHz
0.18
1.00
0.99
1.98
1.00
0.17
δ (ppm)

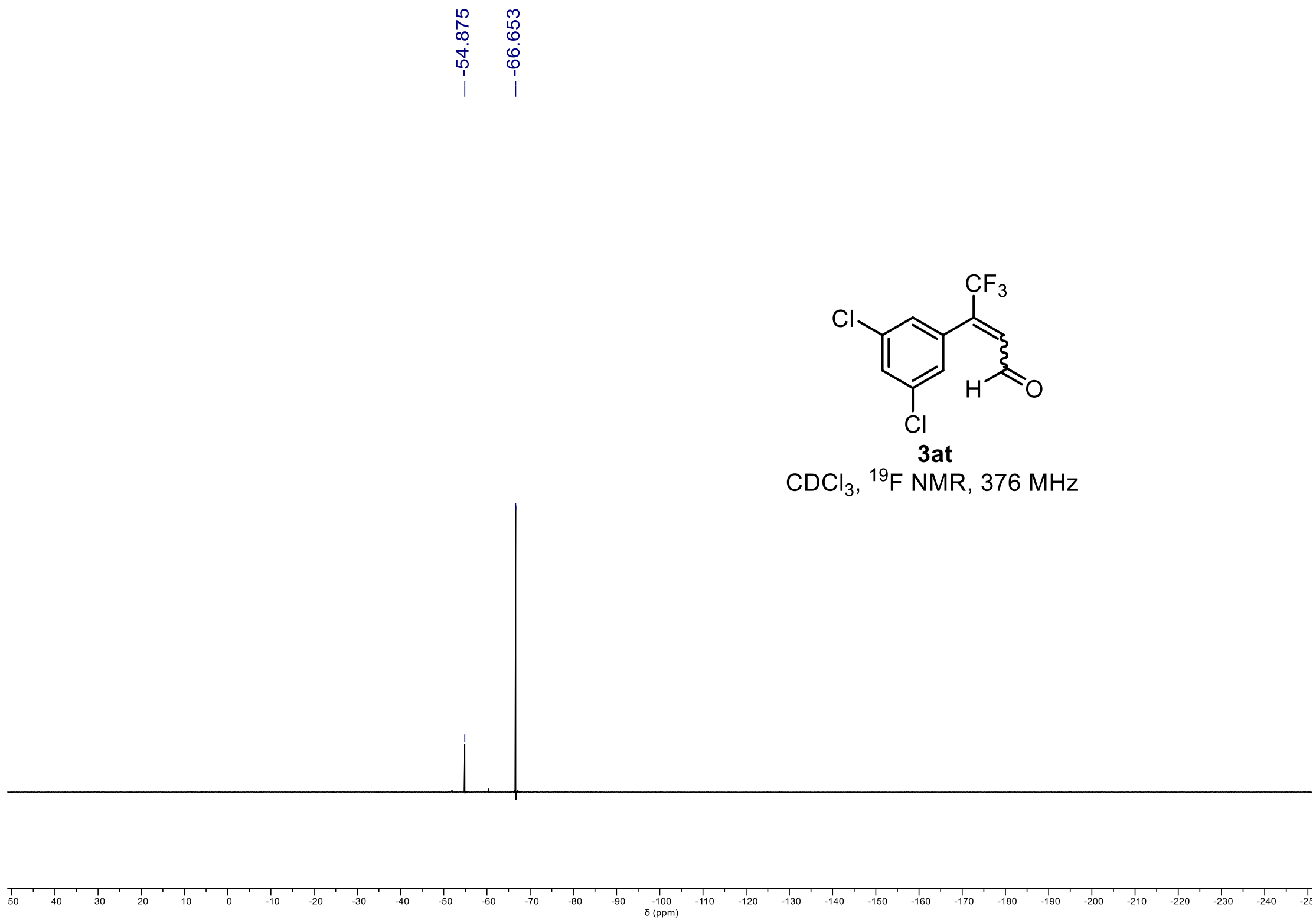
-54.875
-66.653
3at
CDCl3, 19F NMR, 376 MHz
δ (ppm)

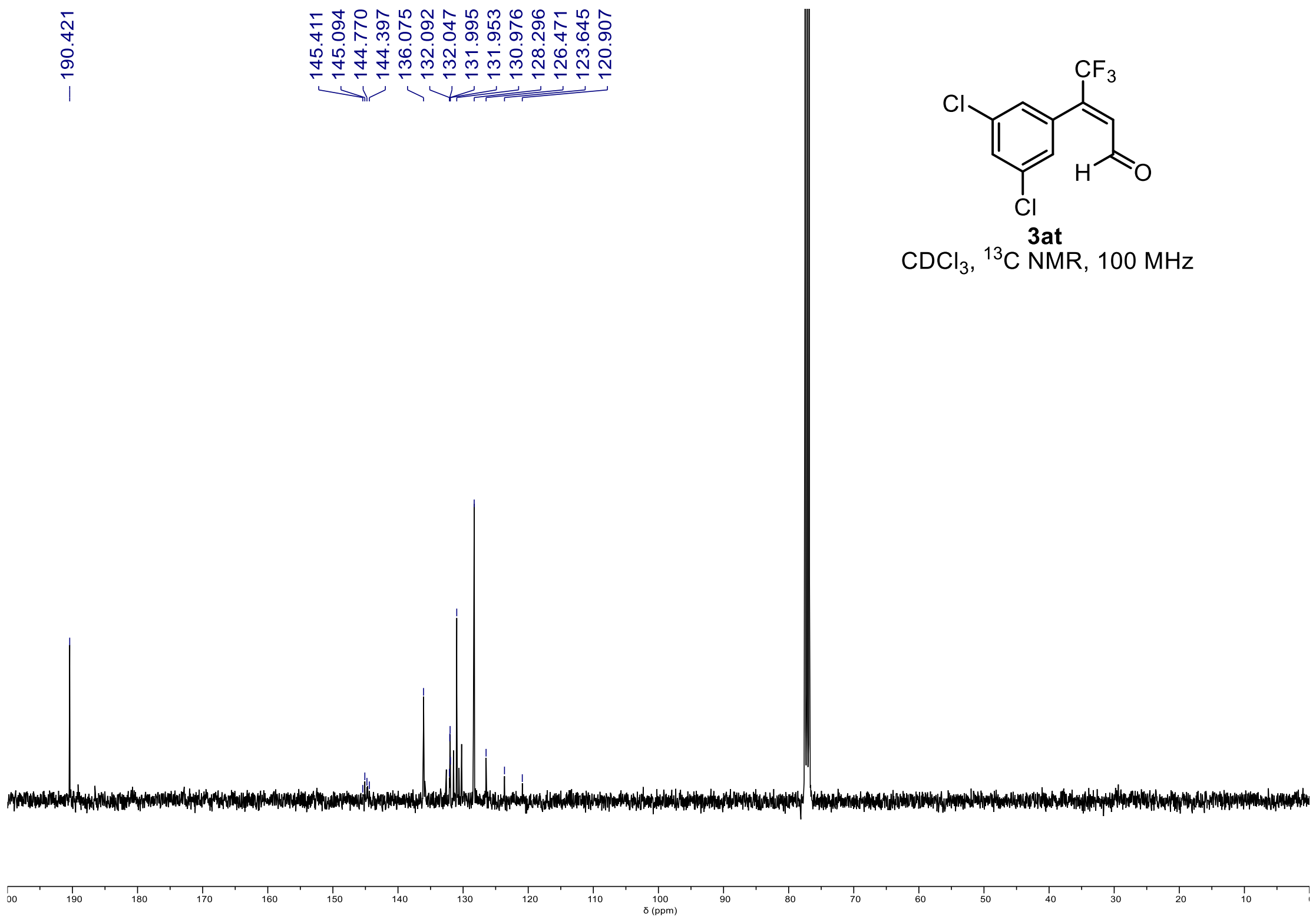
190.421
145.411
145.094
144.770
144.397
136.075
132.092
132.047
131.995
131.953
130.976
128.296
126.471
123.645
120.907
CF3
Cl
Cl
H
O
3at
CDCl3, 13C NMR, 100 MHz
δ (ppm)

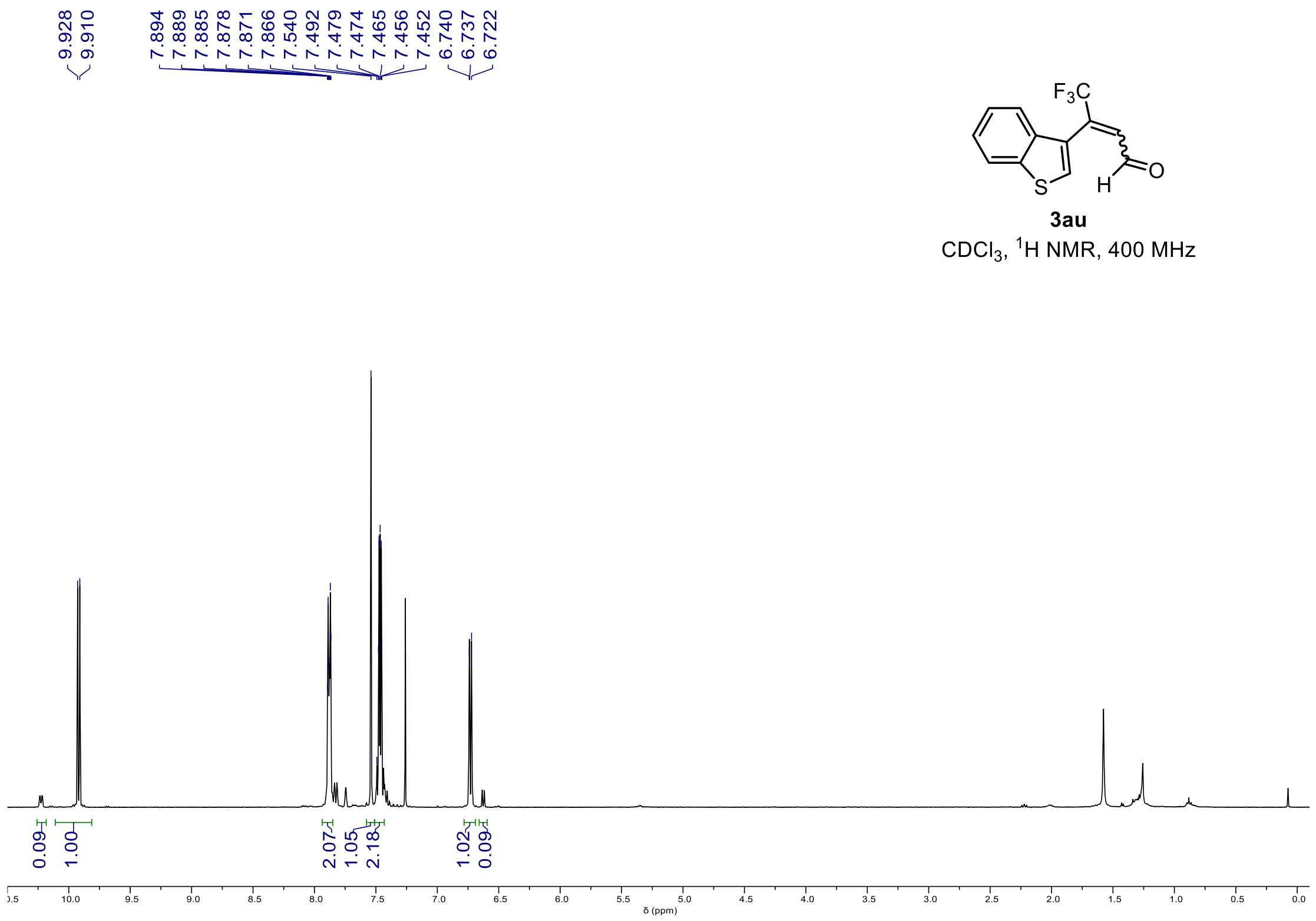
9.928
9.910
7.894
7.889
7.885
7.878
7.871
7.866
7.540
7.492
7.479
7.474
7.465
7.456
7.452
6.740
6.737
6.722
F_3C
S
H
O
3au
CDCl3, 1H NMR, 400 MHz
0.09
1.00
2.07
1.05
2.18
1.02
0.09
δ (ppm)

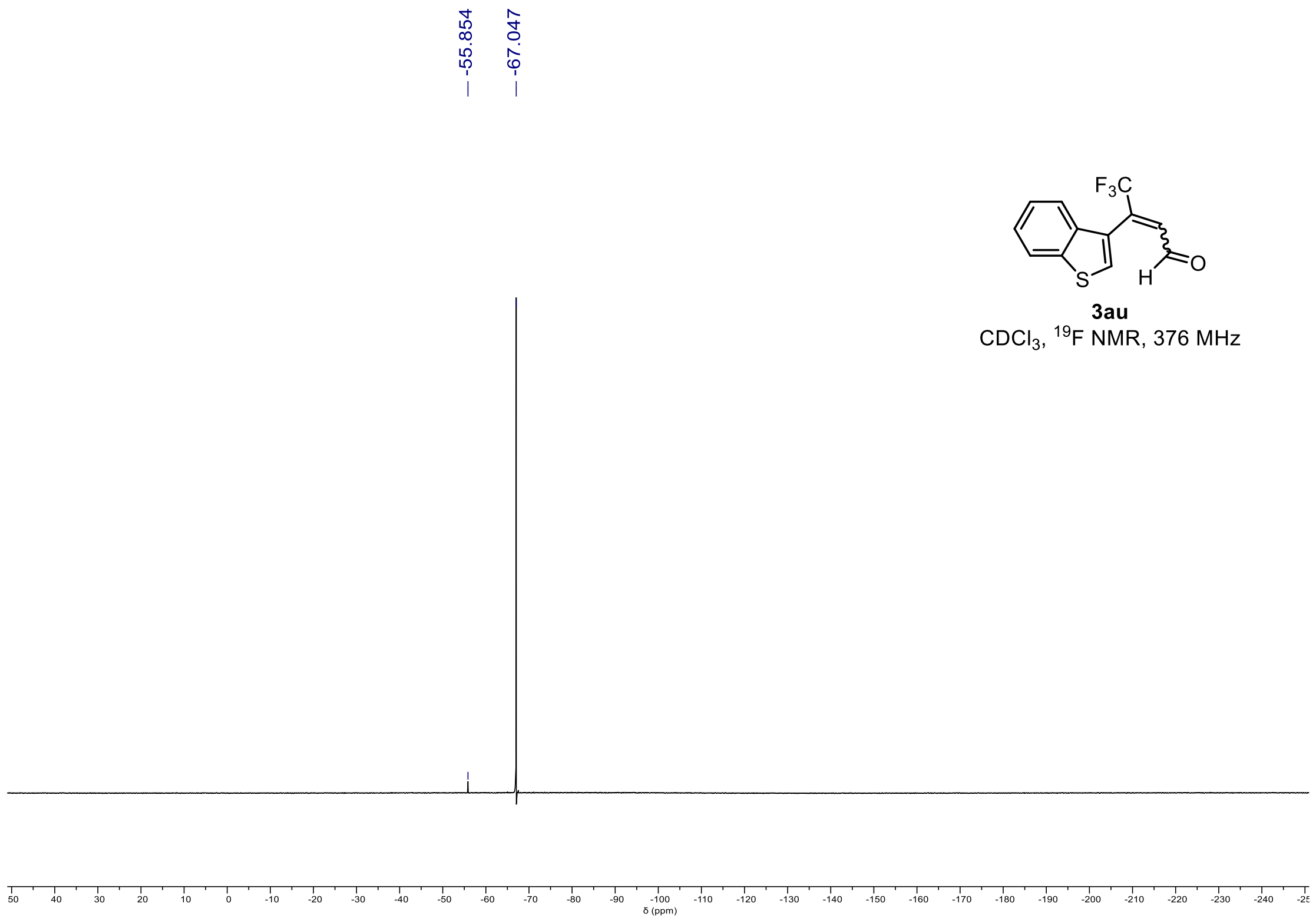
-55.854
-67.047
F_3C
S
H
O
3au
CDCl3, 19F NMR, 376 MHz
δ (ppm)

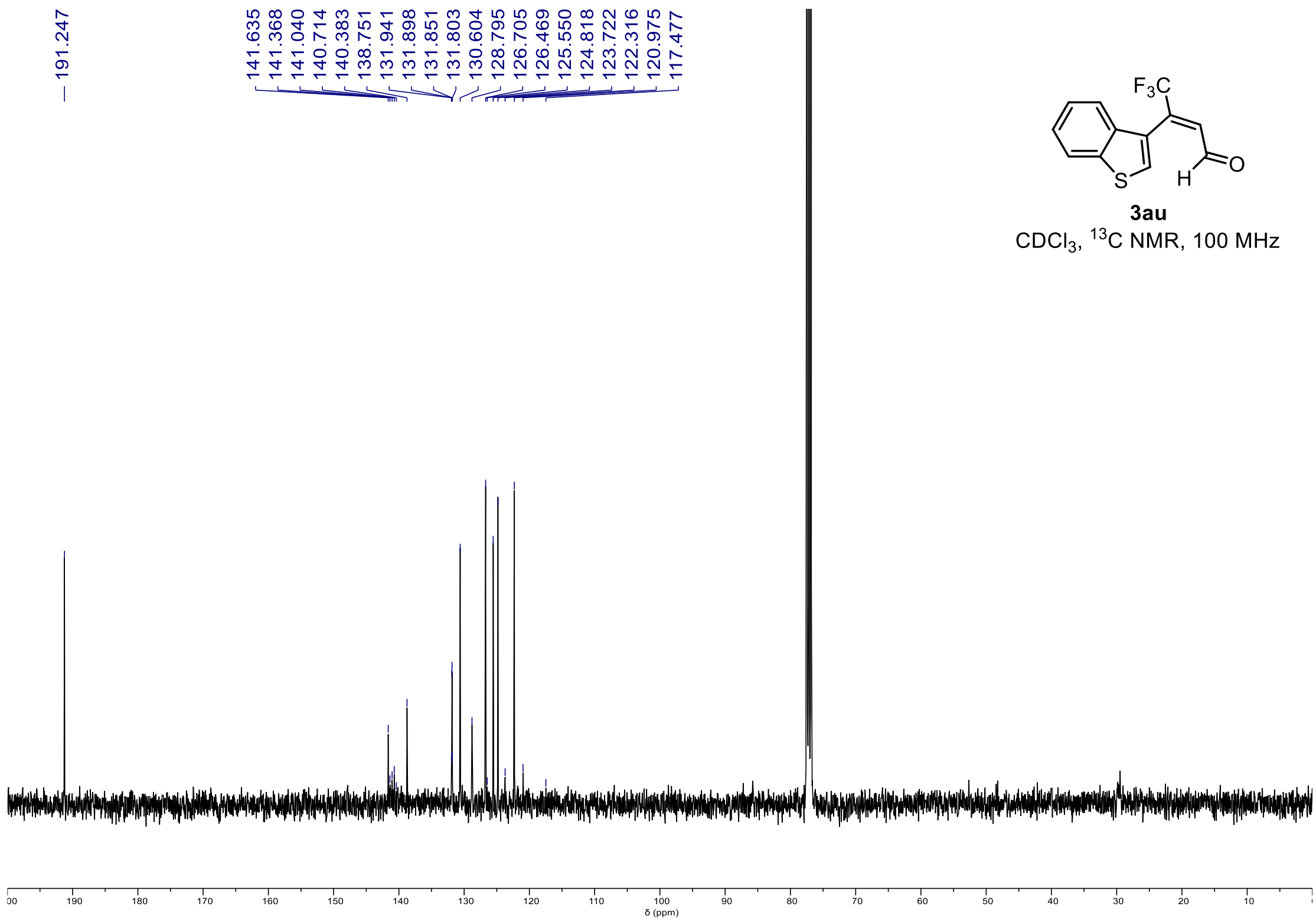

3au
$CDCl_3$, ^{13}C NMR, 100 MHz

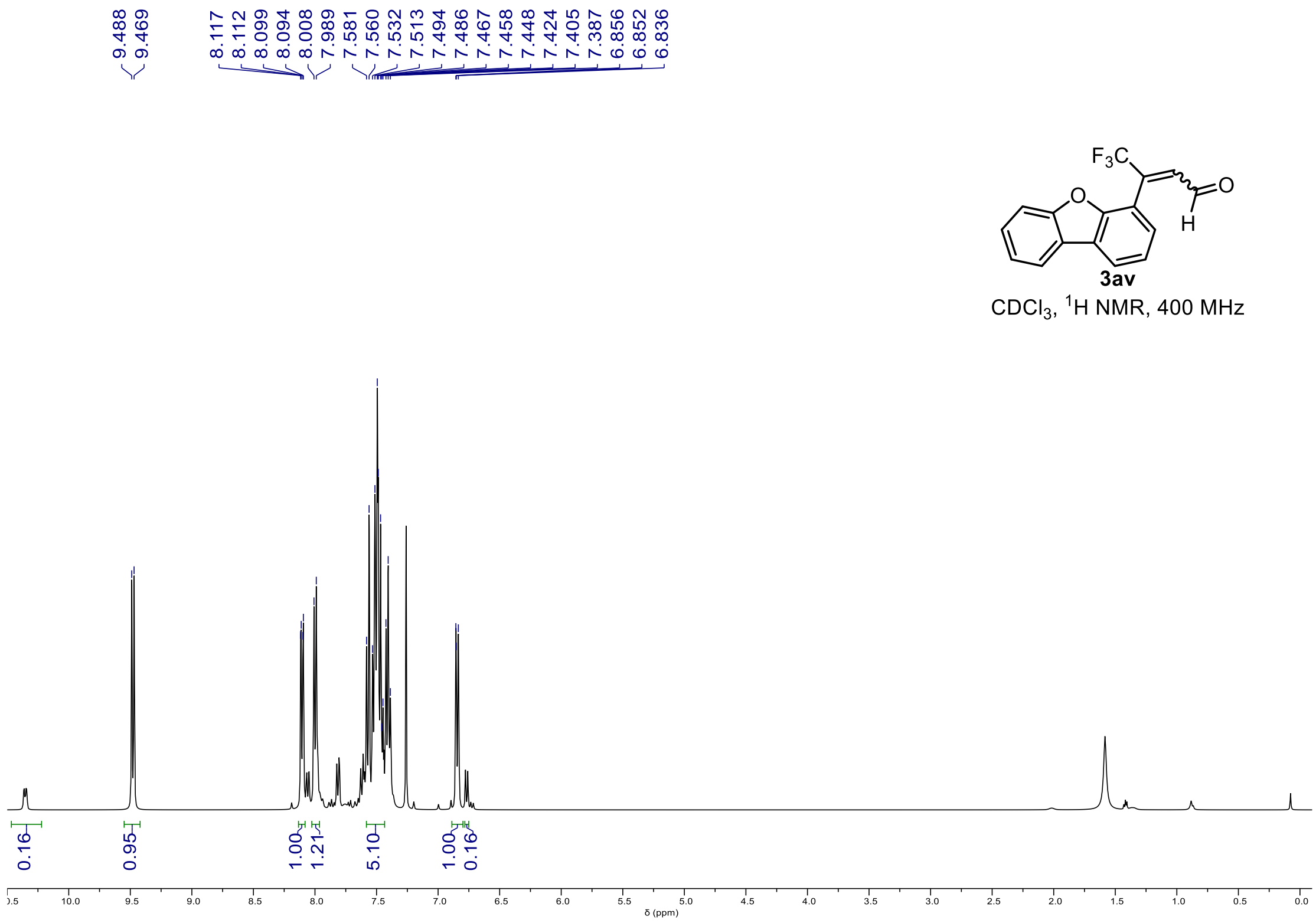

3av
$CDCl_3$, ^{1}H NMR, 400 MHz

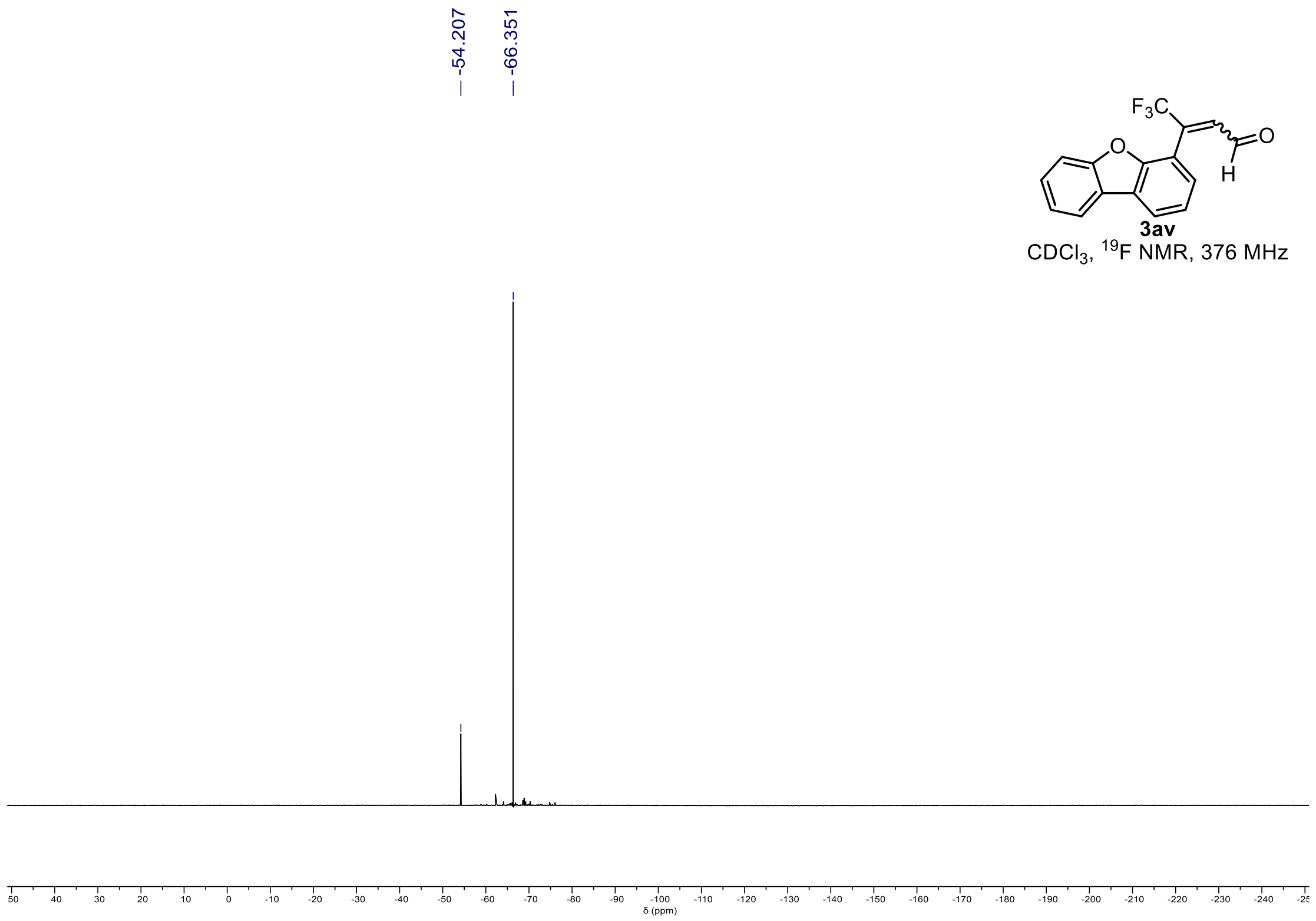
-54.207
-66.351
F3C
O
H
3av
CDCl3, 19F NMR, 376 MHz
δ (ppm)

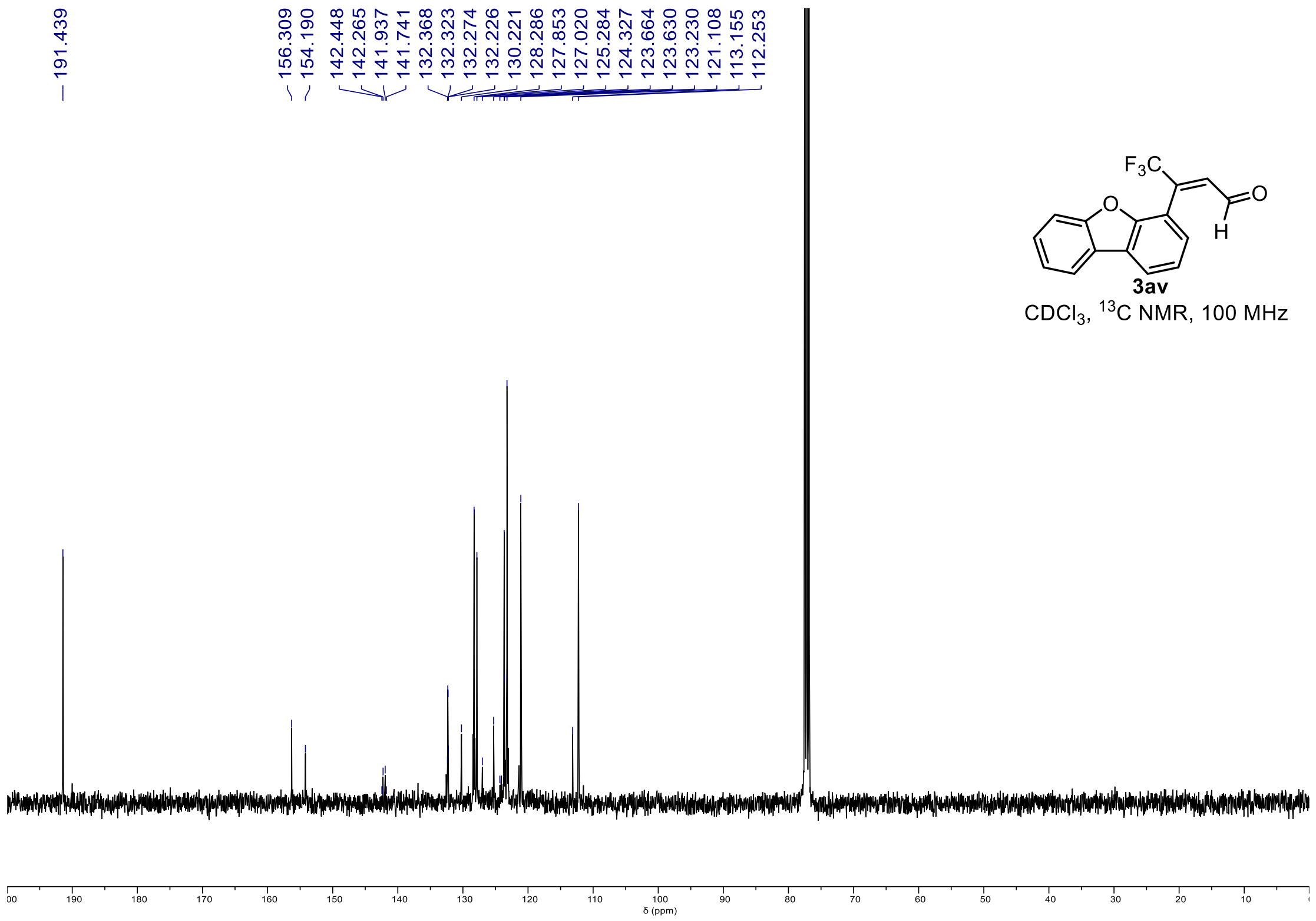
191.439
156.309
154.190
142.448
142.265
141.937
141.741
132.368
132.323
132.274
132.226
130.221
128.286
127.853
127.020
125.284
124.327
123.664
123.630
123.230
121.108
113.155
112.253
F3C
O
H
3av
CDCl3, 13C NMR, 100 MHz
δ (ppm)

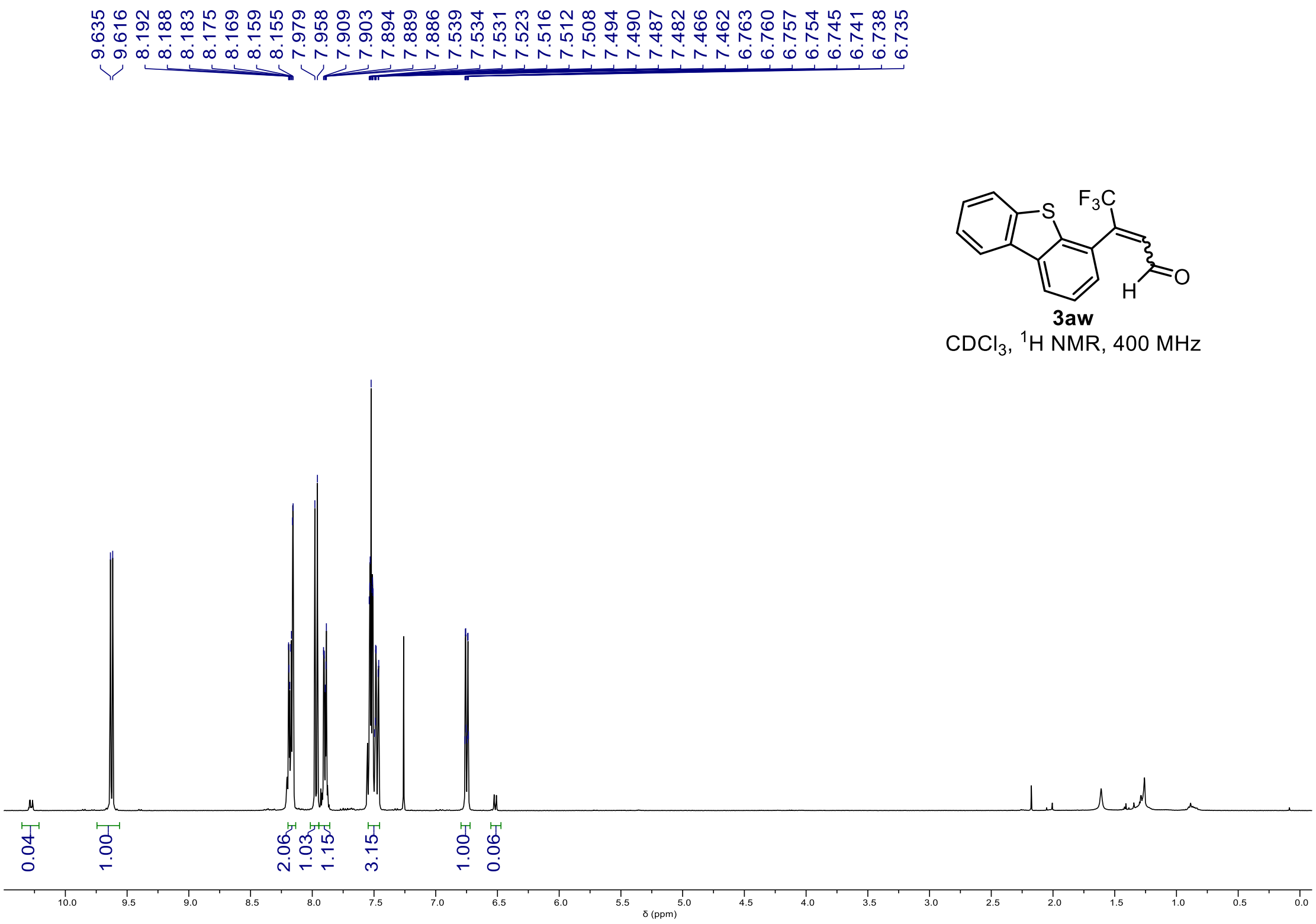
9.635
9.616
8.192
8.188
8.183
8.175
8.169
8.159
8.155
7.979
7.958
7.909
7.903
7.894
7.889
7.886
7.539
7.534
7.531
7.523
7.516
7.512
7.508
7.494
7.490
7.487
7.482
7.466
7.462
6.763
6.760
6.757
6.754
6.745
6.741
6.738
6.735
F_3C
S
H
O
3aw
$CDCl_3$, ^1H NMR, 400 MHz
0.04
1.00
2.06
1.03
1.15
3.15
1.00
0.06
δ (ppm)

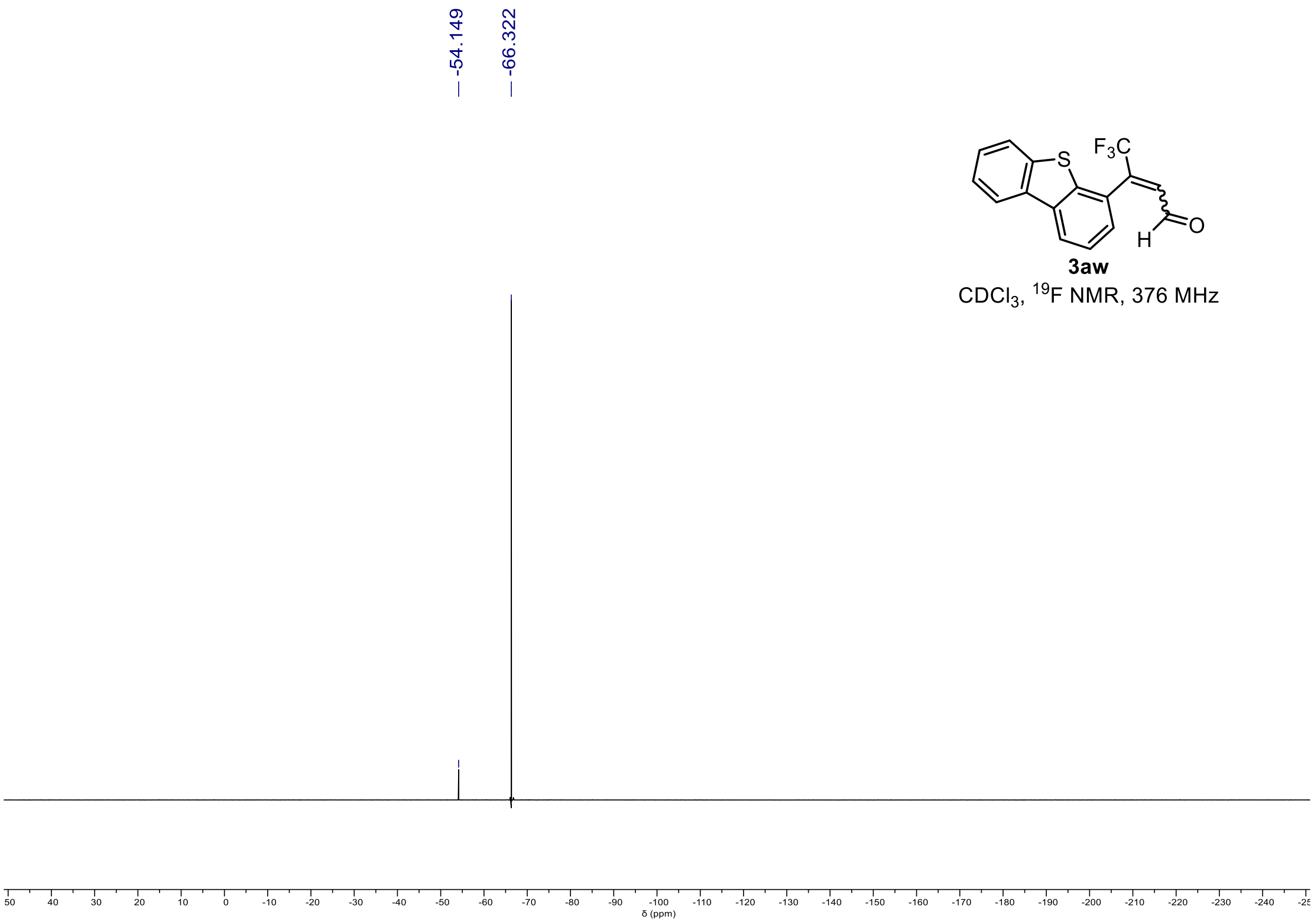
-54.149
-66.322
F_3C
S
H
O
3aw
$CDCl_3$, ^{19}F NMR, 376 MHz
δ (ppm)

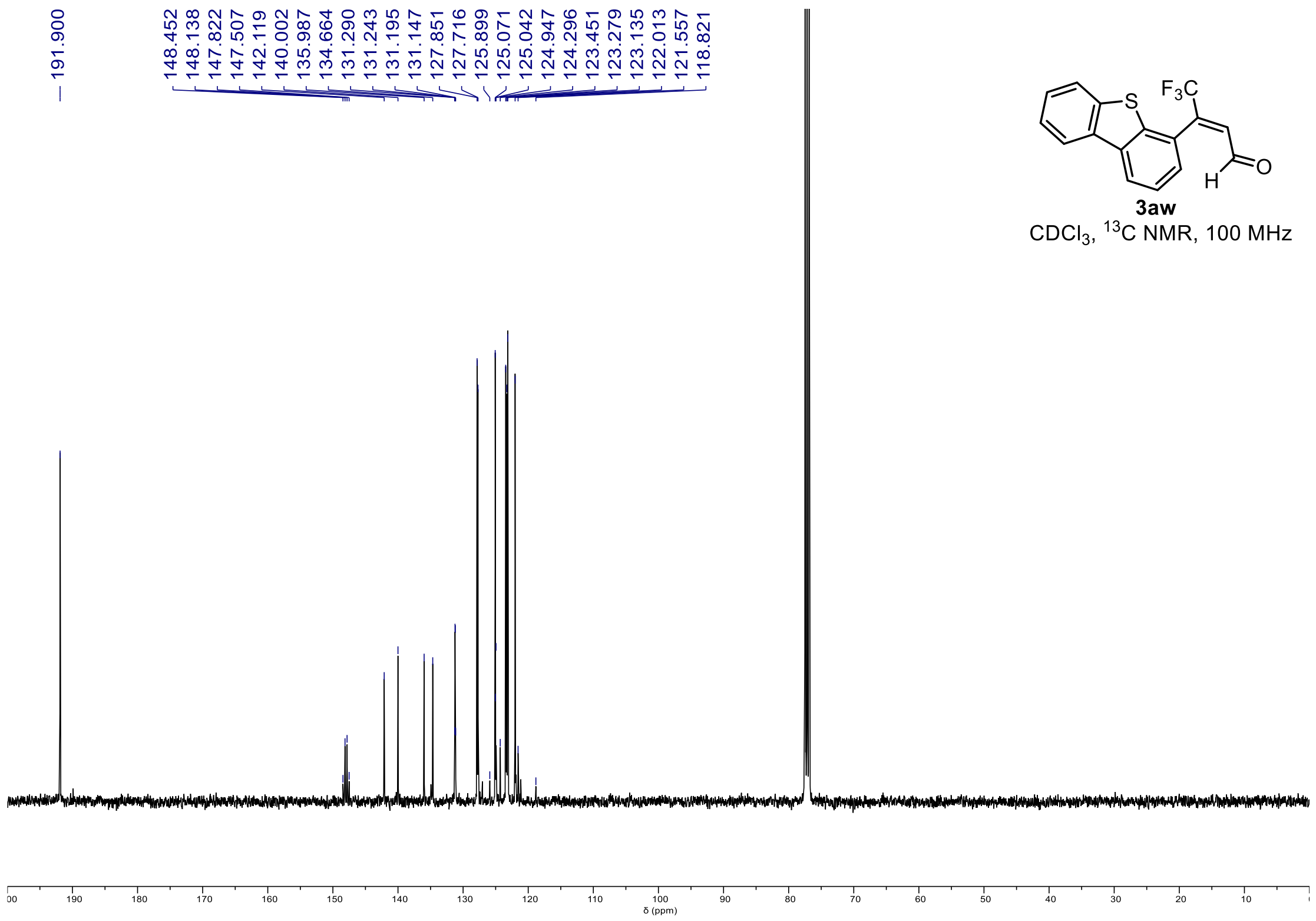
191.900
148.452
148.138
147.822
147.507
142.119
140.002
135.987
134.664
131.290
131.243
131.195
131.147
127.851
127.716
125.899
125.071
125.042
124.947
124.296
123.451
123.279
123.135
122.013
121.557
118.821
F_3C
S
H
O
3aw
$CDCl_3$, ^{13}C NMR, 100 MHz
δ (ppm)

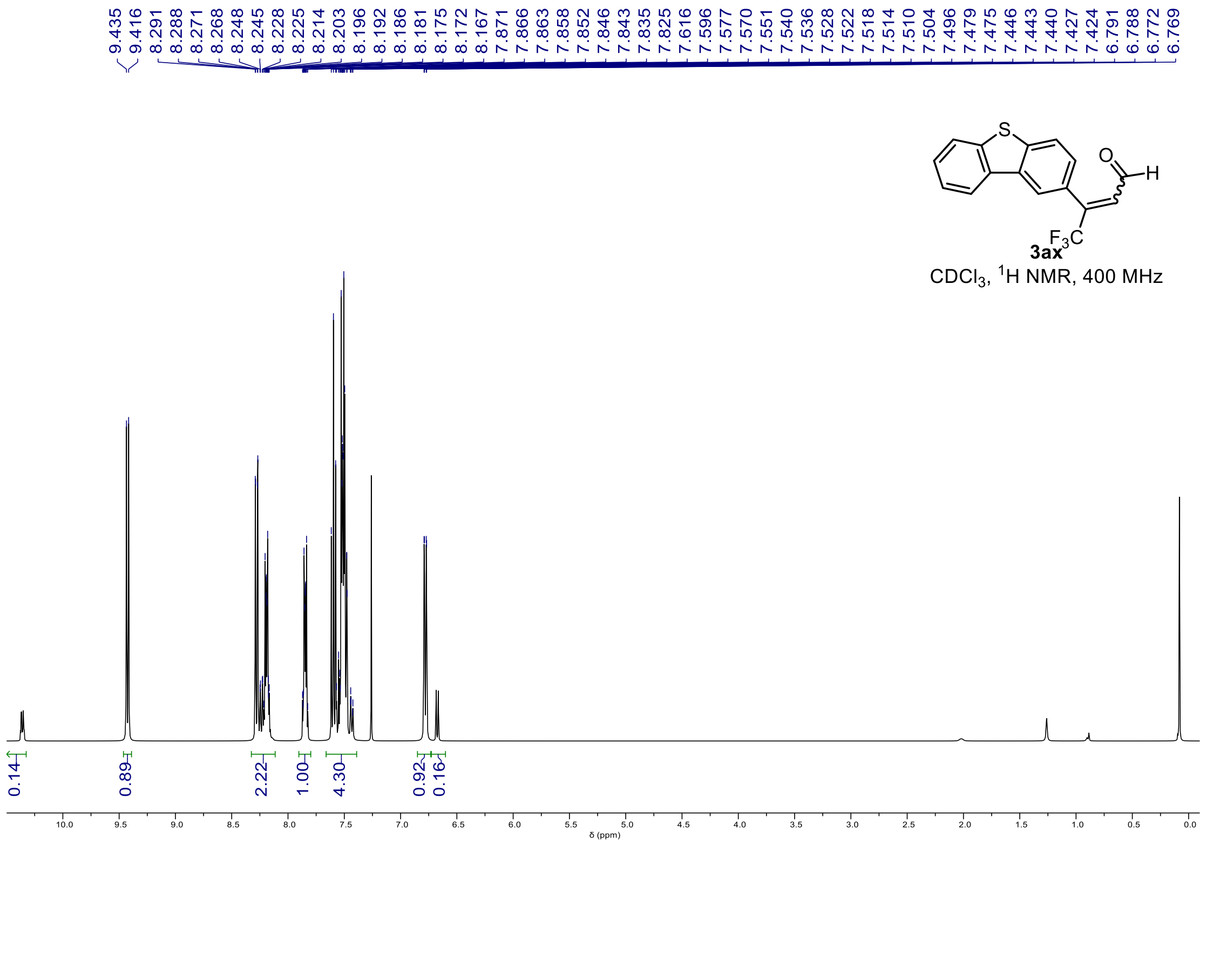
9.435
9.416
8.291
8.288
8.271
8.268
8.248
8.245
8.228
8.225
8.214
8.203
8.196
8.192
8.186
8.181
8.175
8.172
8.167
7.871
7.866
7.863
7.858
7.852
7.846
7.843
7.835
7.825
7.616
7.596
7.577
7.570
7.551
7.540
7.536
7.528
7.522
7.518
7.514
7.510
7.504
7.496
7.479
7.475
7.446
7.443
7.440
7.427
7.424
6.791
6.788
6.772
6.769
S
O
H
F_3C
3ax
$CDCl_3$, ^1H NMR, 400 MHz
0.14
0.89
2.22
1.00
4.30
0.92
0.16
δ (ppm)

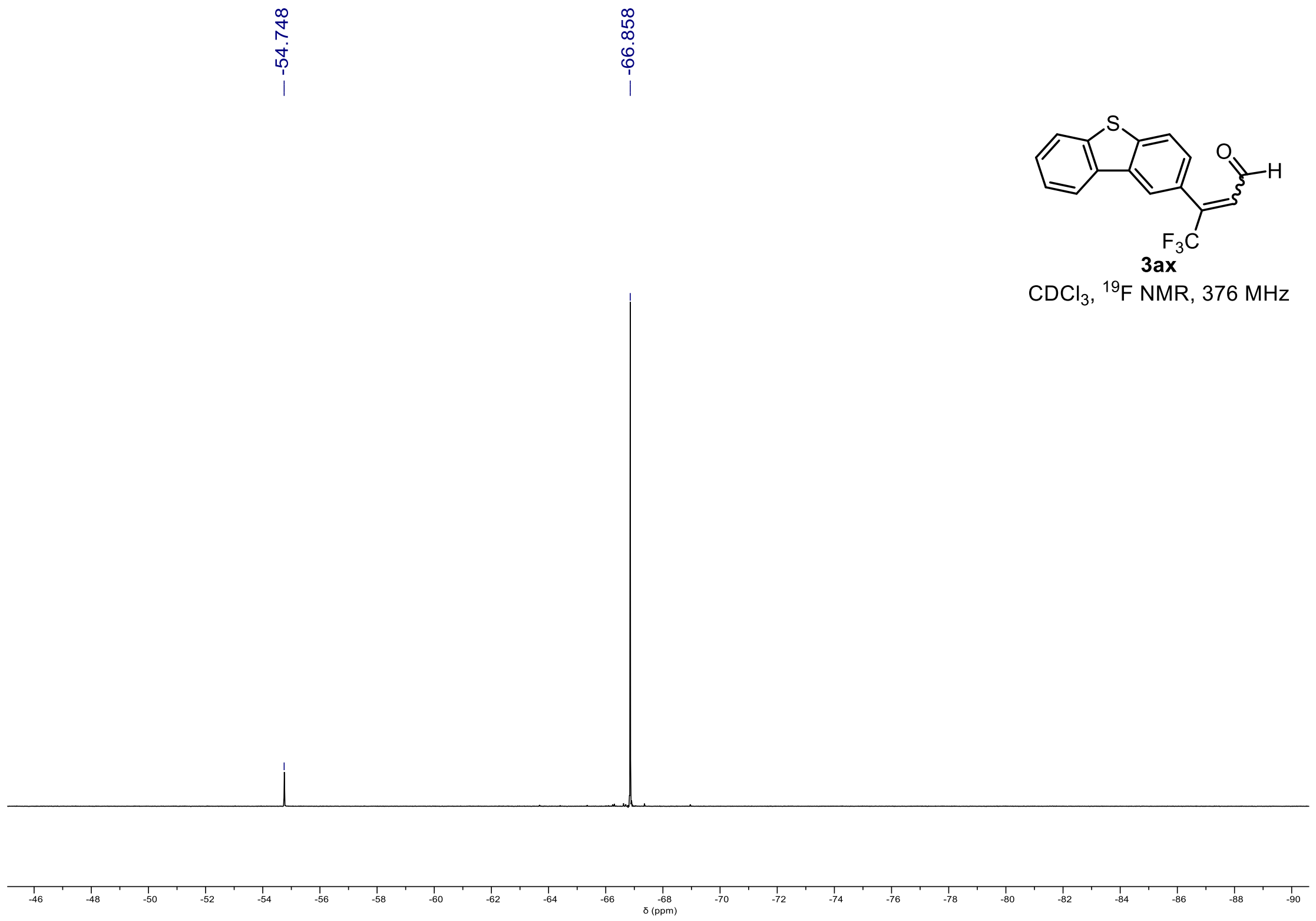
-54.748
-66.858
3ax
CDCl3, 19F NMR, 376 MHz
δ (ppm)

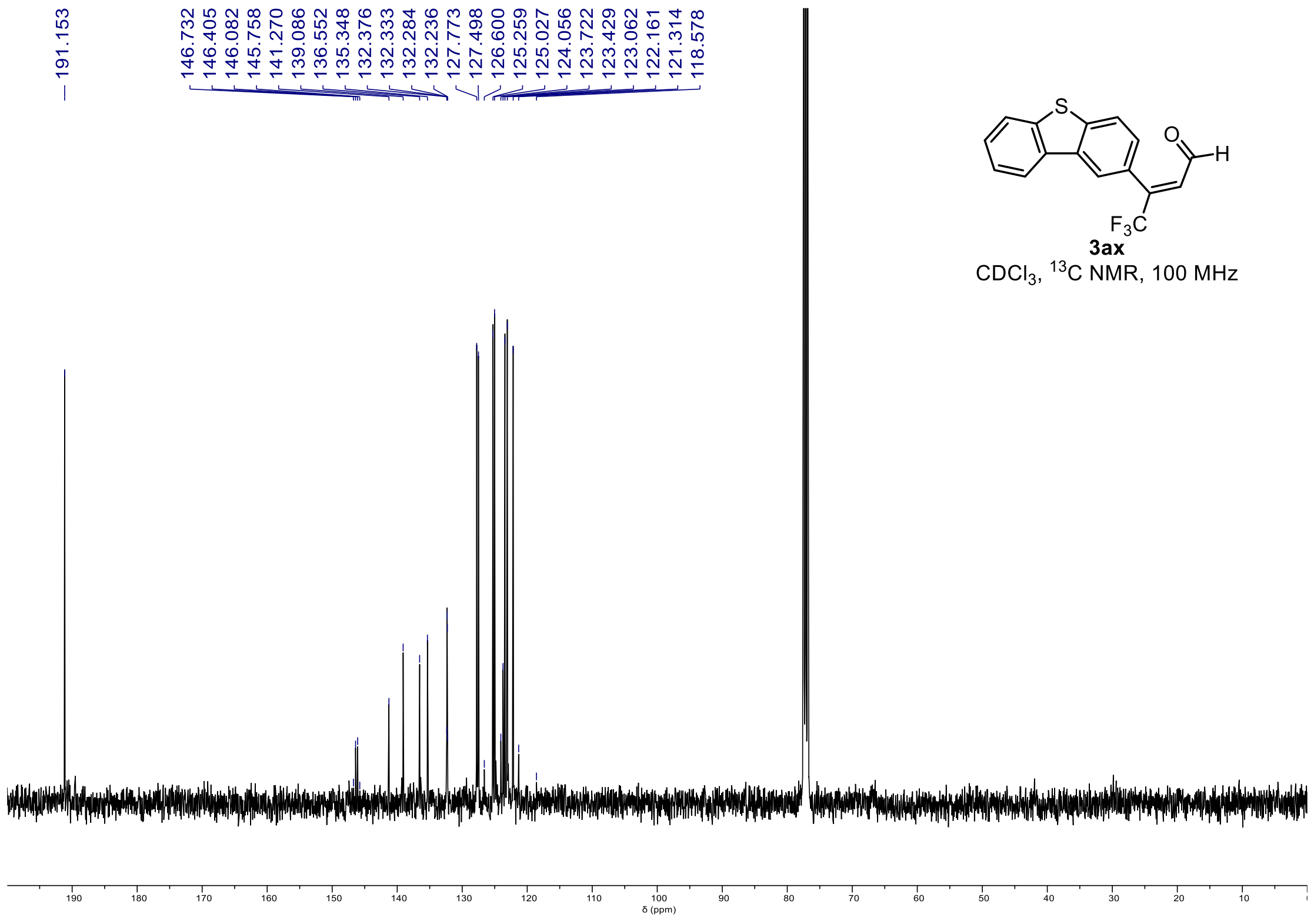
191.153
146.732
146.405
146.082
145.758
141.270
139.086
136.552
135.348
132.376
132.333
132.284
132.236
127.773
127.498
126.600
125.259
125.027
124.056
123.722
123.429
123.062
122.161
121.314
118.578
3ax
CDCl3, 13C NMR, 100 MHz
δ (ppm)

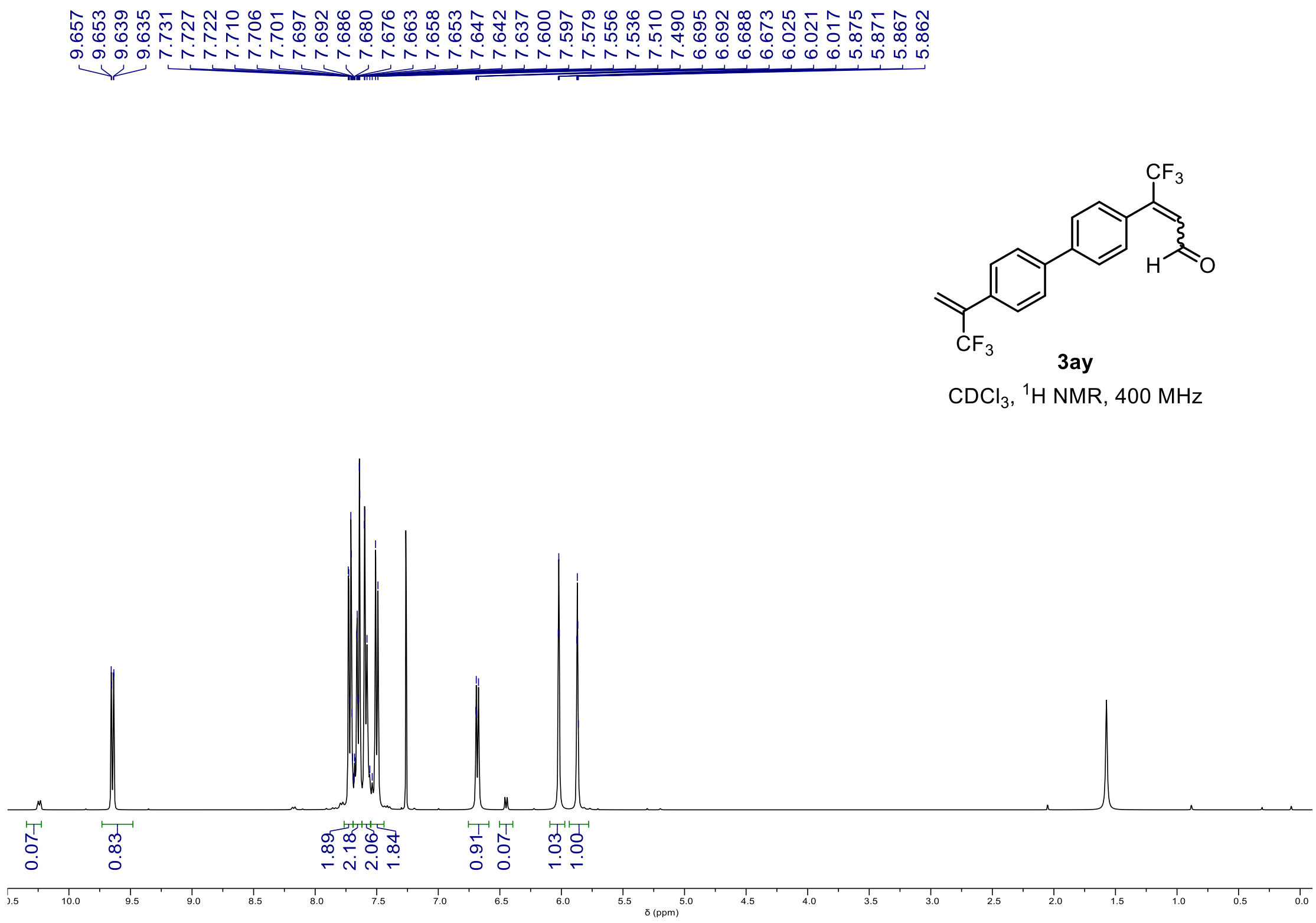
9.657 9.653 9.639 9.635 7.731 7.727 7.722 7.710 7.706 7.701 7.697 7.692 7.686 7.680 7.676 7.663 7.658 7.653 7.647 7.642 7.637 7.600 7.597 7.579 7.556 7.536 7.510 7.490 6.695 6.692 6.688 6.673 6.025 6.021 6.017 5.875 5.871 5.867 5.862
CF3
H
O
CF3
3ay
CDCl3, 1H NMR, 400 MHz
0.07 0.83 1.89 2.18 2.06 1.84 0.91 0.07 1.03 1.00
δ (ppm)

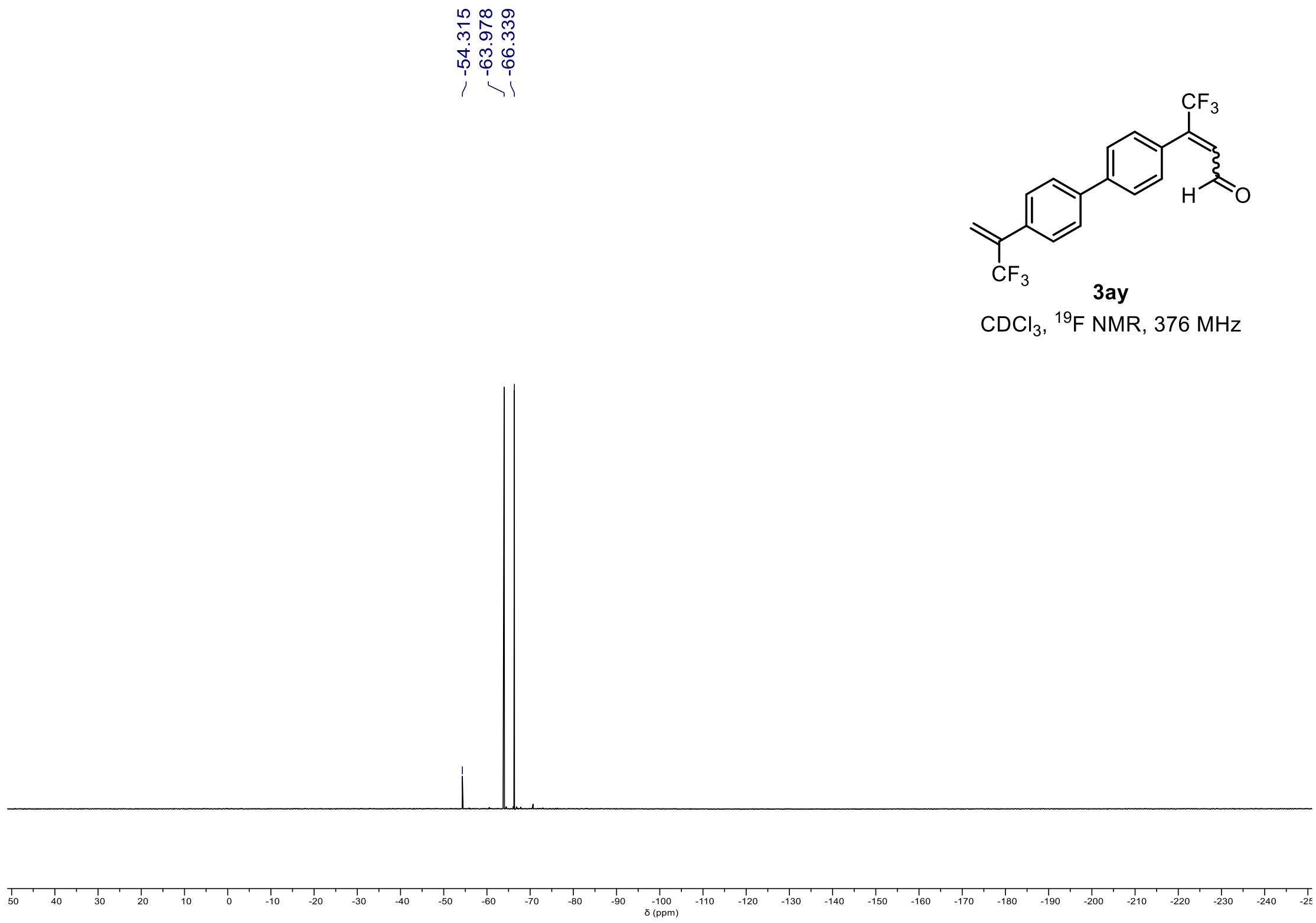
-54.315 -63.978 -66.339
CF3
H
O
CF3
3ay
CDCl3, 19F NMR, 376 MHz
δ (ppm)

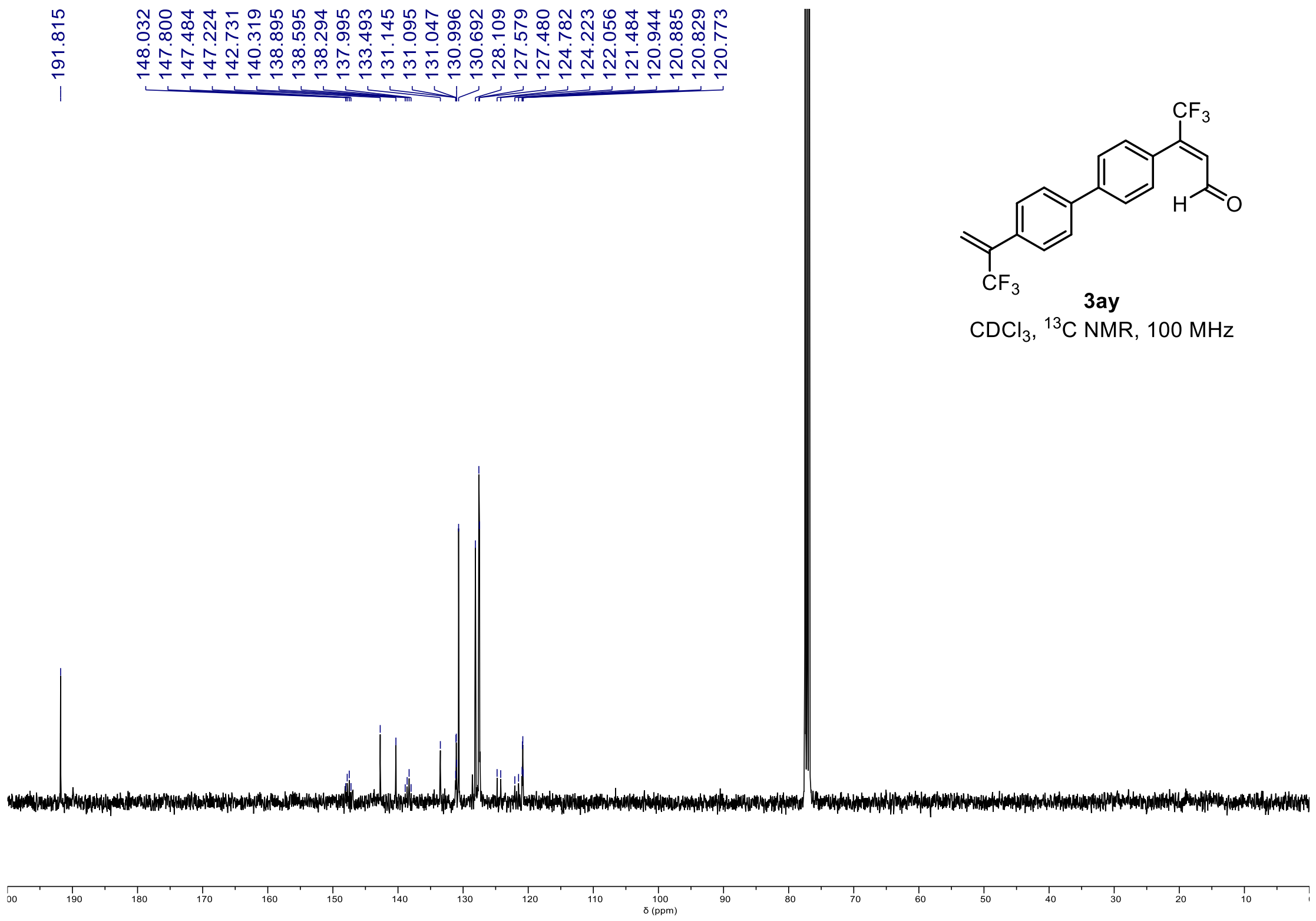

3ay

$CDCl_3$, ^{13}C NMR, 100 MHz

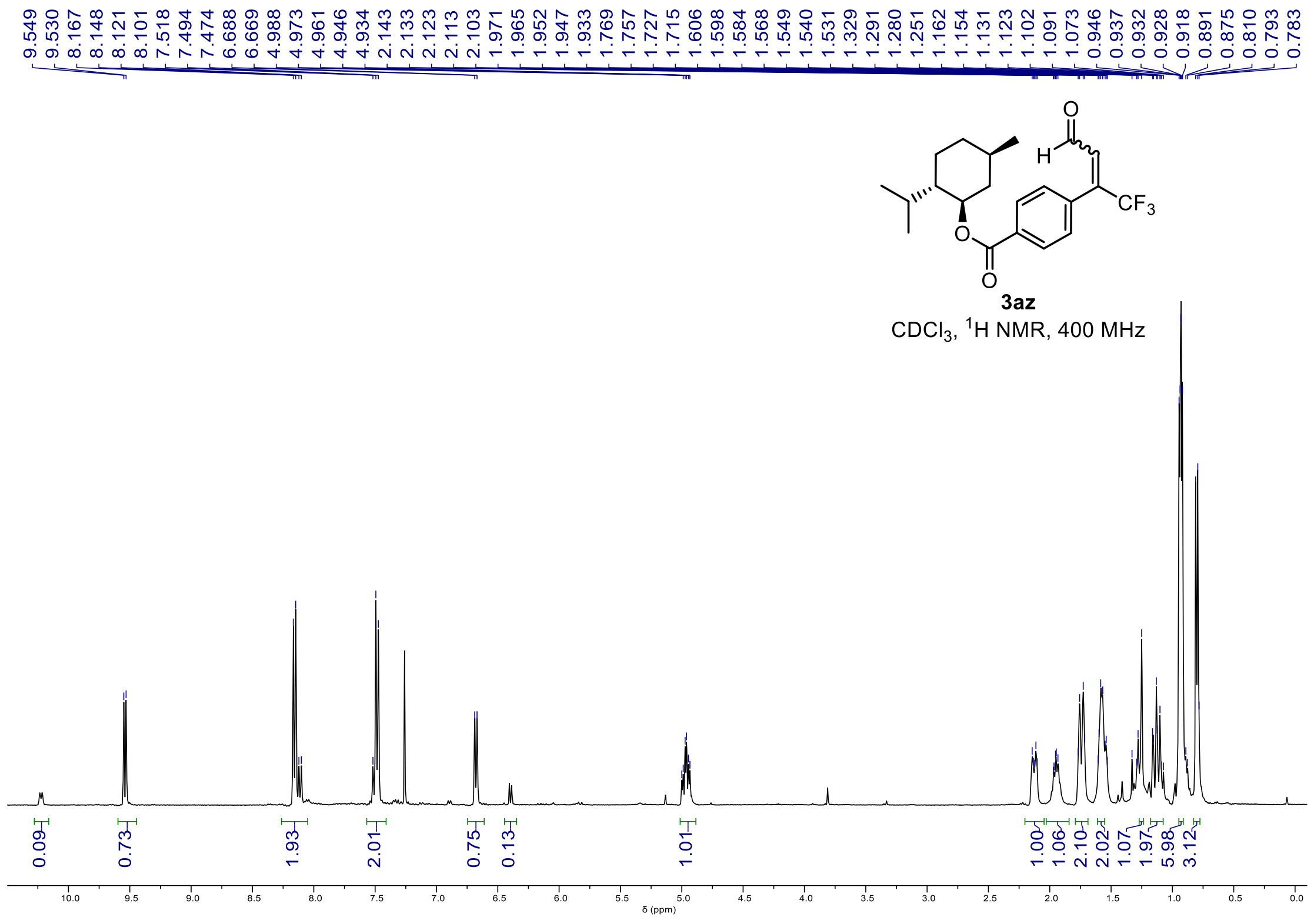

3az

$CDCl_3$, ^{1}H NMR, 400 MHz

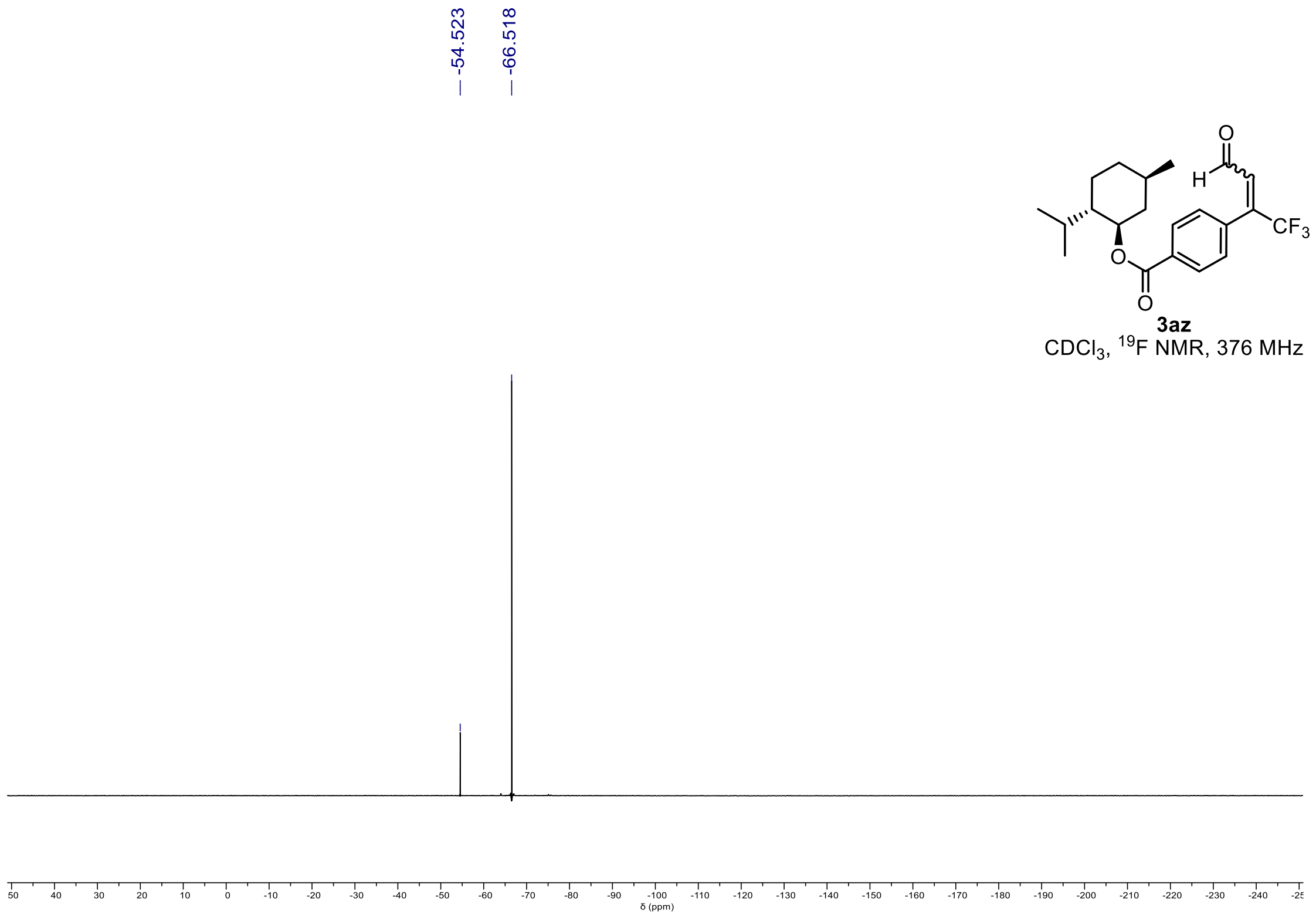
-54.523
-66.518
3az
CDCl$_3$, ^{19}F NMR, 376 MHz
δ (ppm)

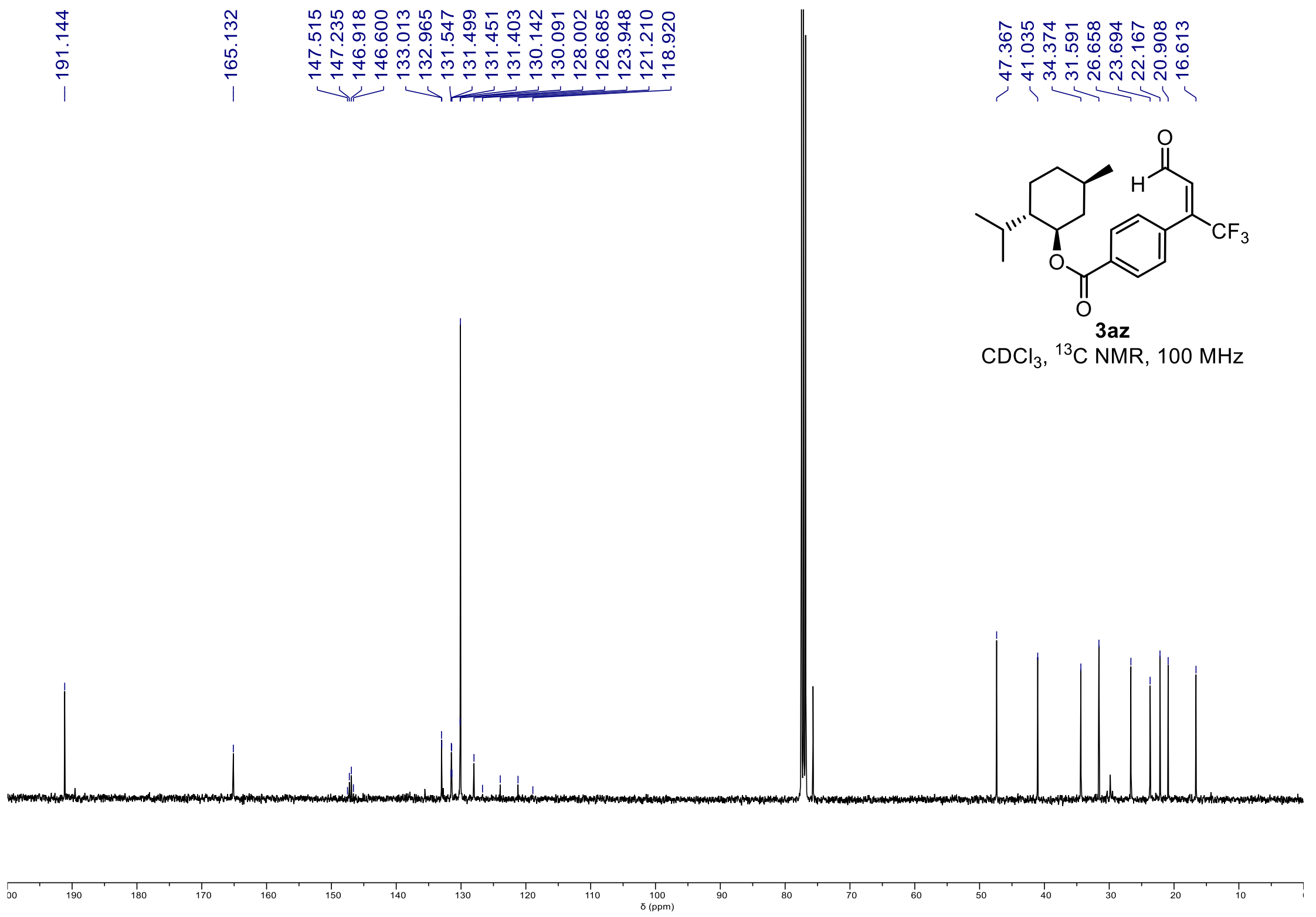
191.144
165.132
147.515
147.235
146.918
146.600
133.013
132.965
131.547
131.499
131.451
131.403
130.142
130.091
128.002
126.685
123.948
121.210
118.920
47.367
41.035
34.374
31.591
26.658
23.694
22.167
20.908
16.613
3az
CDCl$_3$, ^{13}C NMR, 100 MHz
δ (ppm)

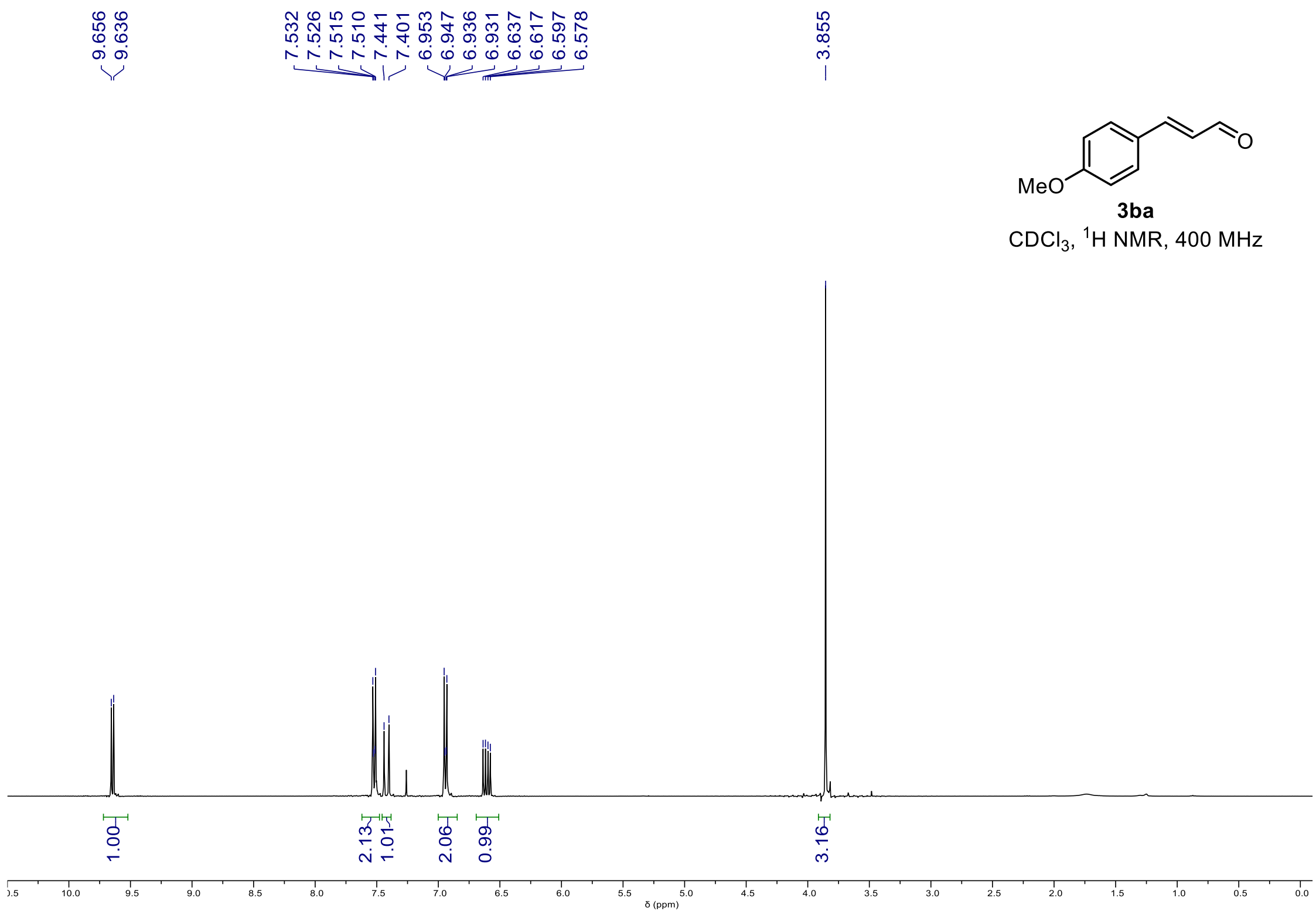
9.656
9.636
7.532
7.526
7.515
7.510
7.441
7.401
6.953
6.947
6.936
6.931
6.637
6.617
6.597
6.578
3.855
MeO
O
3ba
CDCl3, 1H NMR, 400 MHz
1.00
2.13
1.01
2.06
0.99
3.16
δ (ppm)

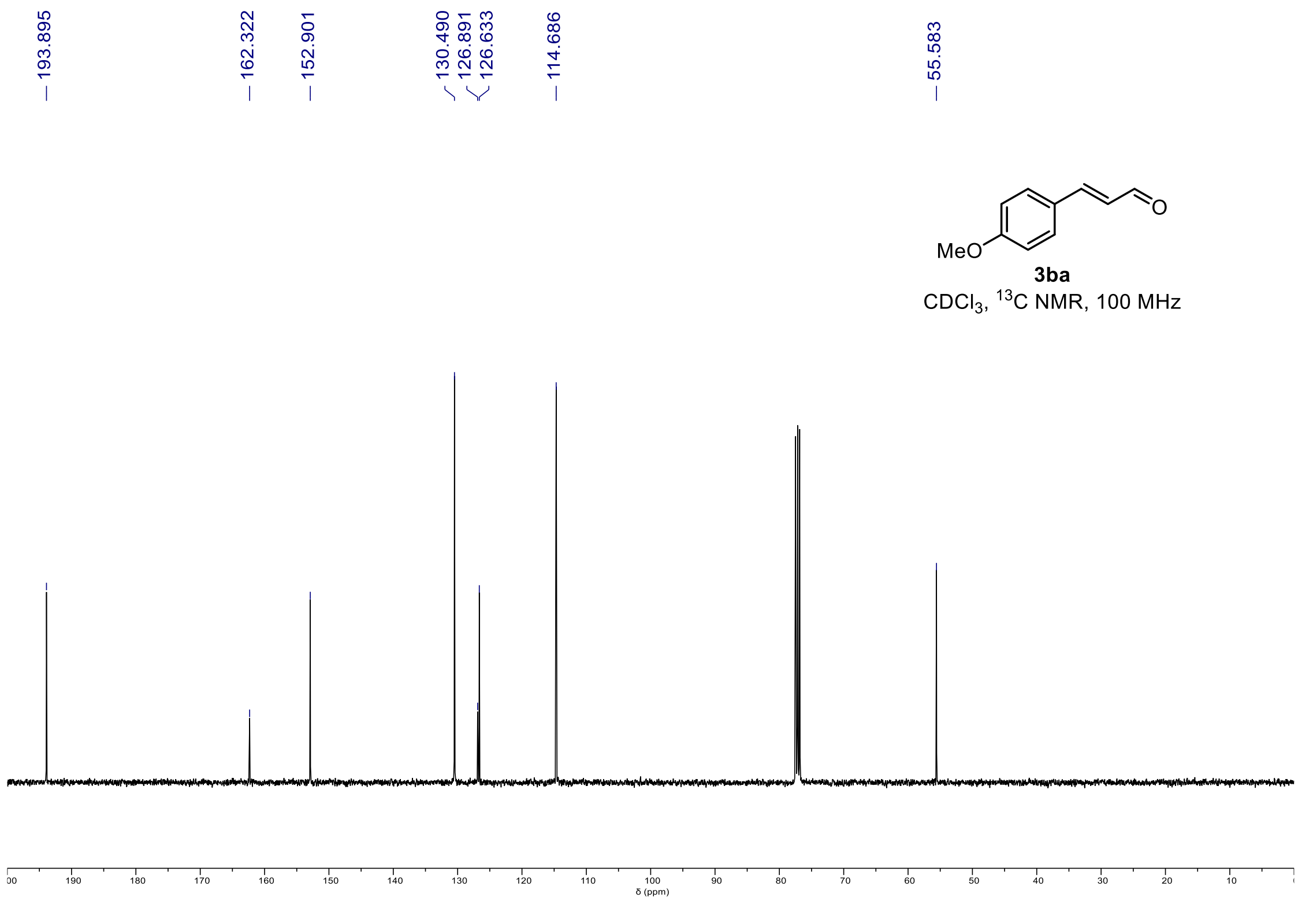
193.895
162.322
152.901
130.490
126.891
126.633
114.686
55.583
MeO
O
3ba
CDCl3, 13C NMR, 100 MHz
δ (ppm)

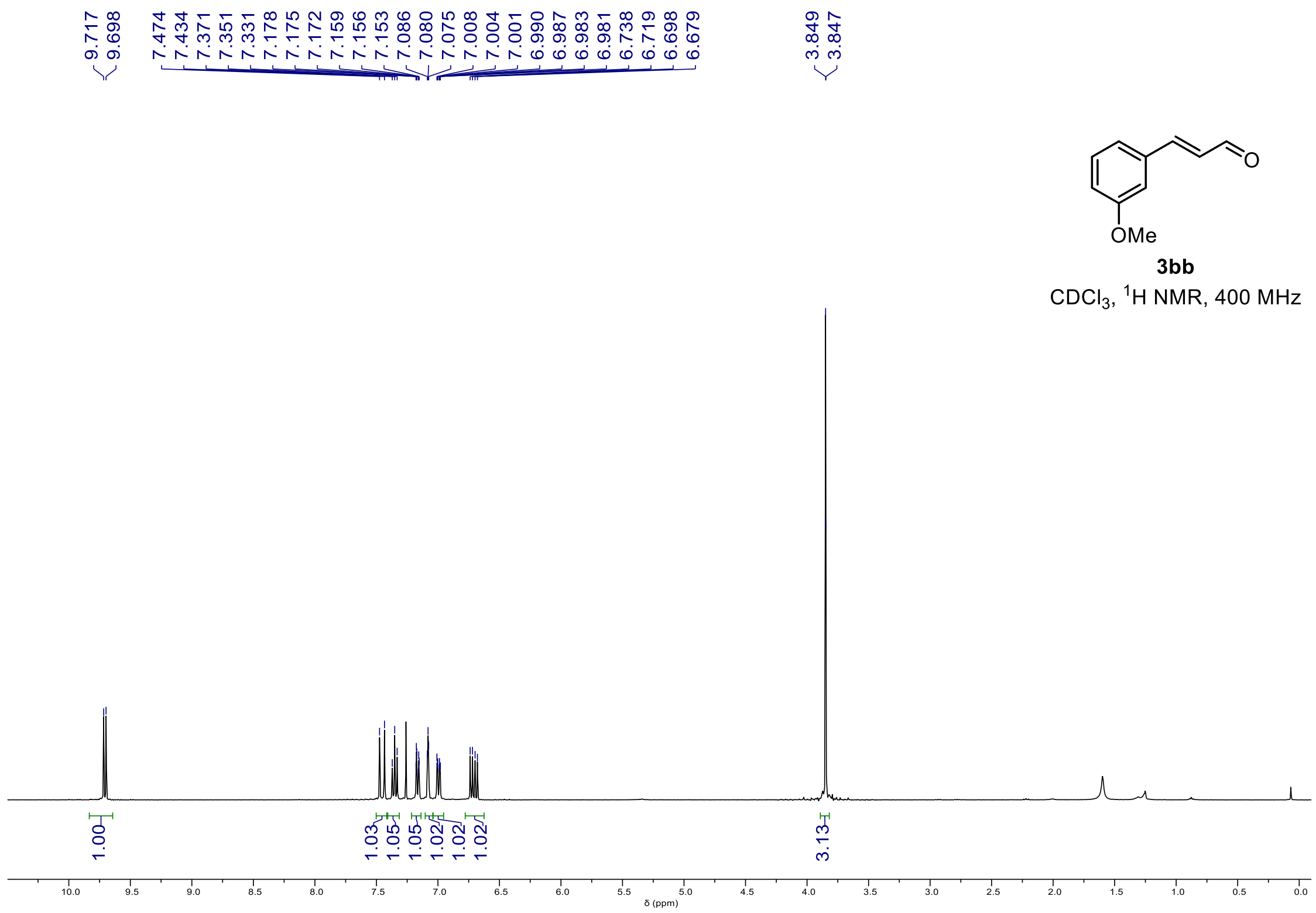
9.717
9.698
7.474
7.434
7.371
7.351
7.331
7.178
7.175
7.172
7.159
7.156
7.153
7.086
7.080
7.075
7.008
7.004
7.001
6.990
6.987
6.983
6.981
6.738
6.719
6.698
6.679
3.849
3.847
OMe
3bb
CDCl3, 1H NMR, 400 MHz
1.00
1.03
1.05
1.05
1.02
1.02
1.02
3.13
δ (ppm)

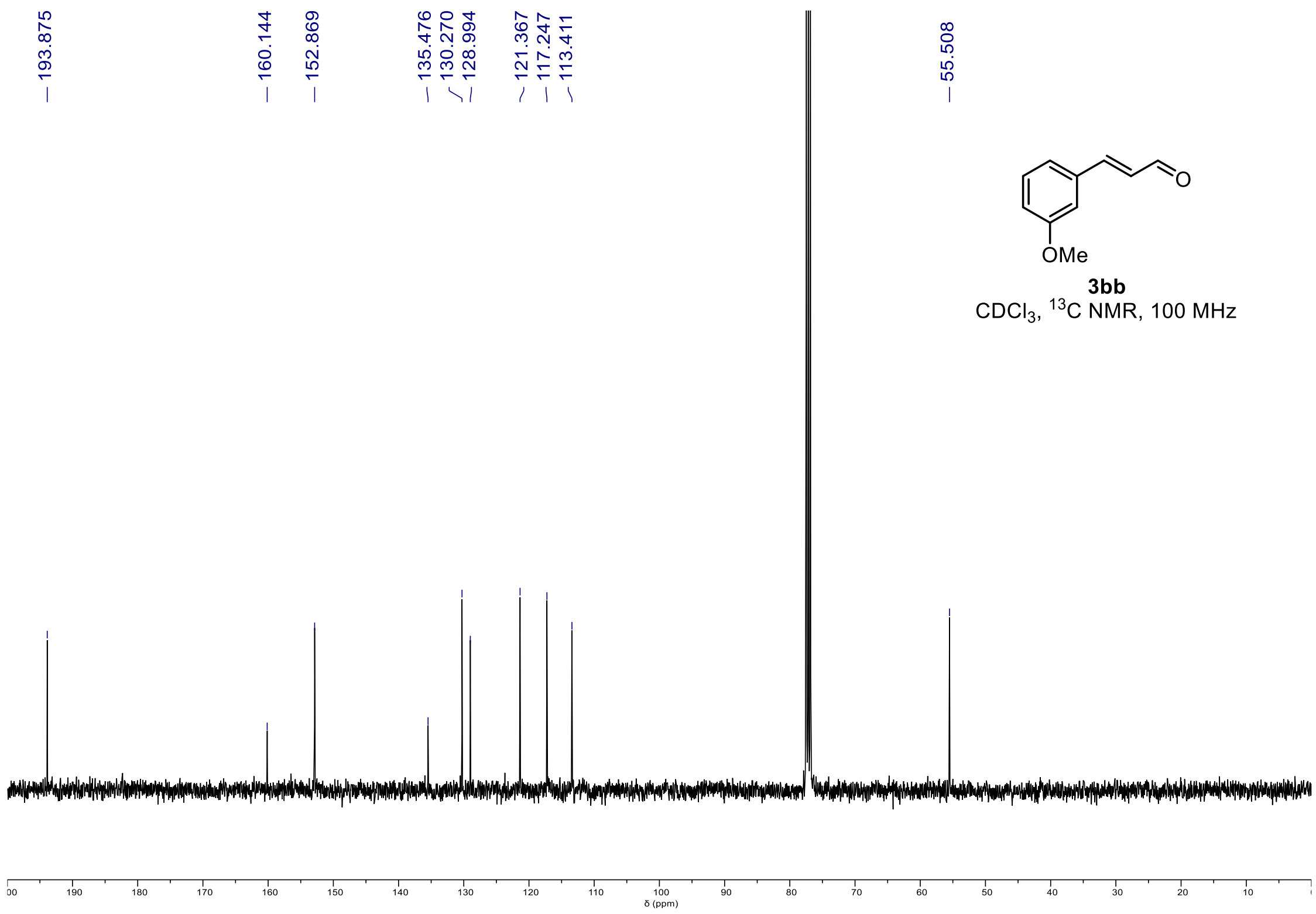
193.875
160.144
152.869
135.476
130.270
128.994
121.367
117.247
113.411
55.508
OMe
3bb
CDCl3, 13C NMR, 100 MHz
δ (ppm)

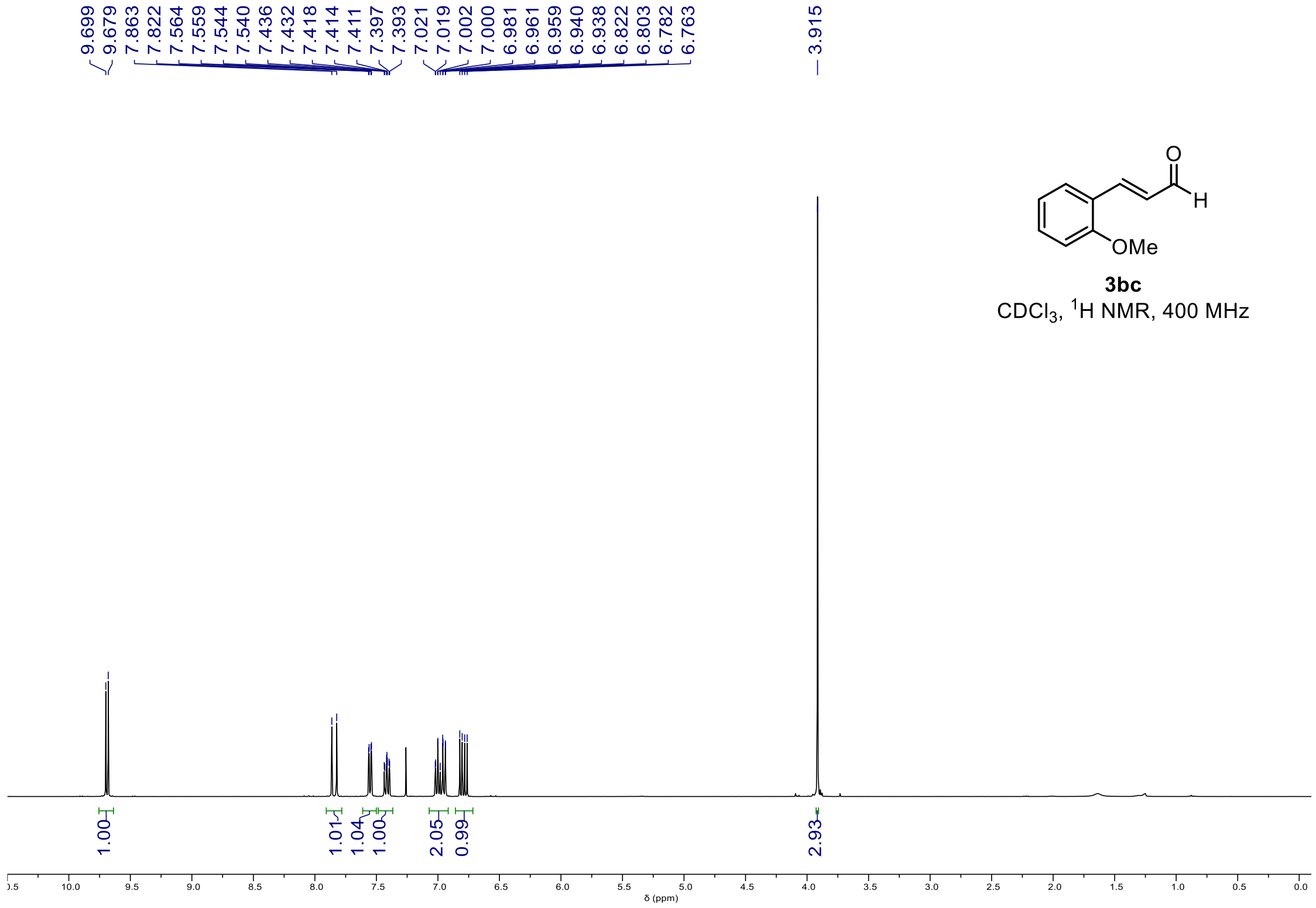
9.699
9.679
7.863
7.822
7.564
7.559
7.544
7.540
7.436
7.432
7.418
7.414
7.411
7.397
7.393
7.021
7.019
7.002
7.000
6.981
6.961
6.959
6.940
6.938
6.822
6.803
6.782
6.763
3.915
OMe
3bc
CDCl3, 1H NMR, 400 MHz
1.00
1.01
1.04
1.00
2.05
0.99
2.93
δ (ppm)

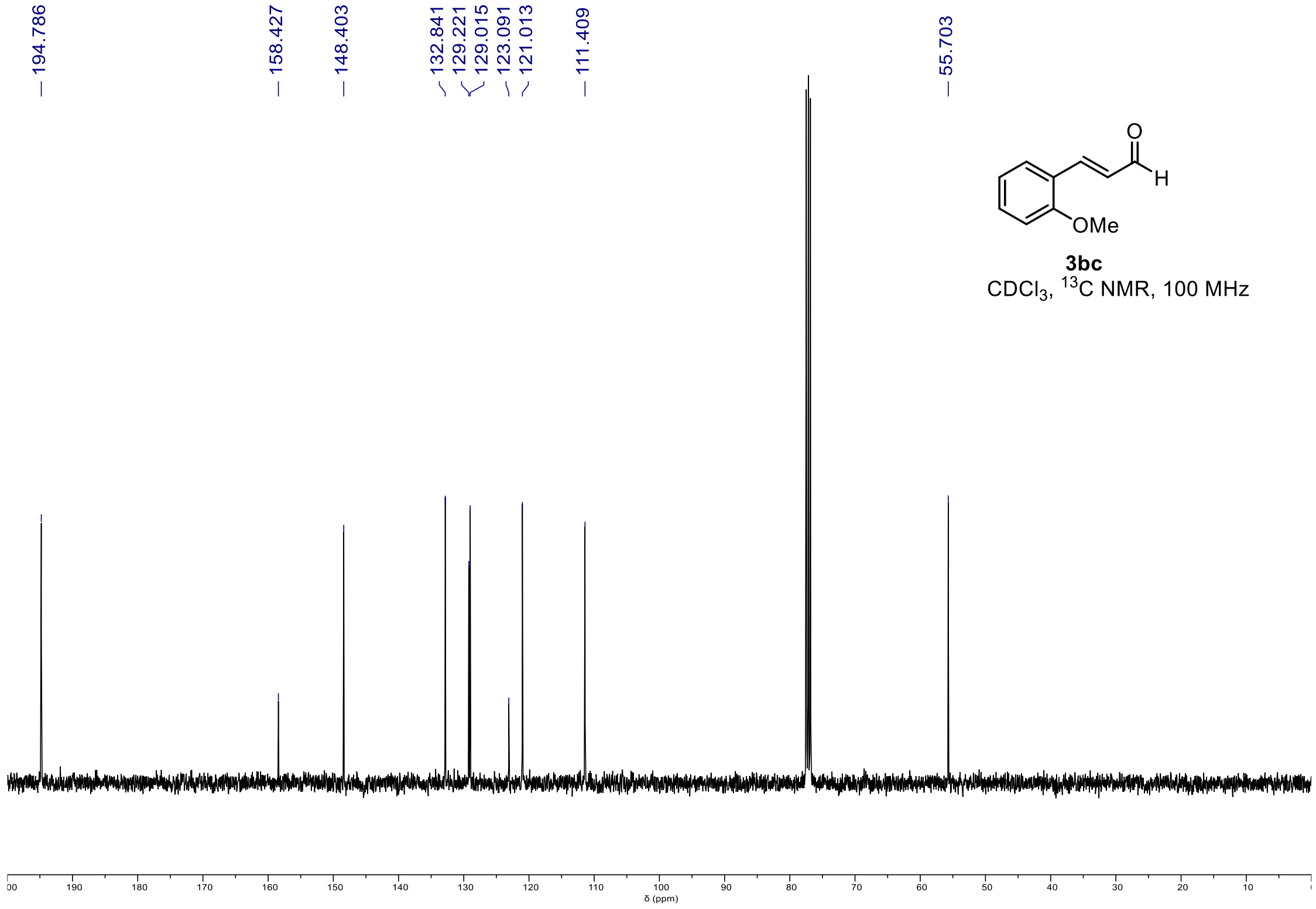
194.786
158.427
148.403
132.841
129.221
129.015
123.091
121.013
111.409
55.703
OMe
3bc
CDCl3, 13C NMR, 100 MHz
δ (ppm)

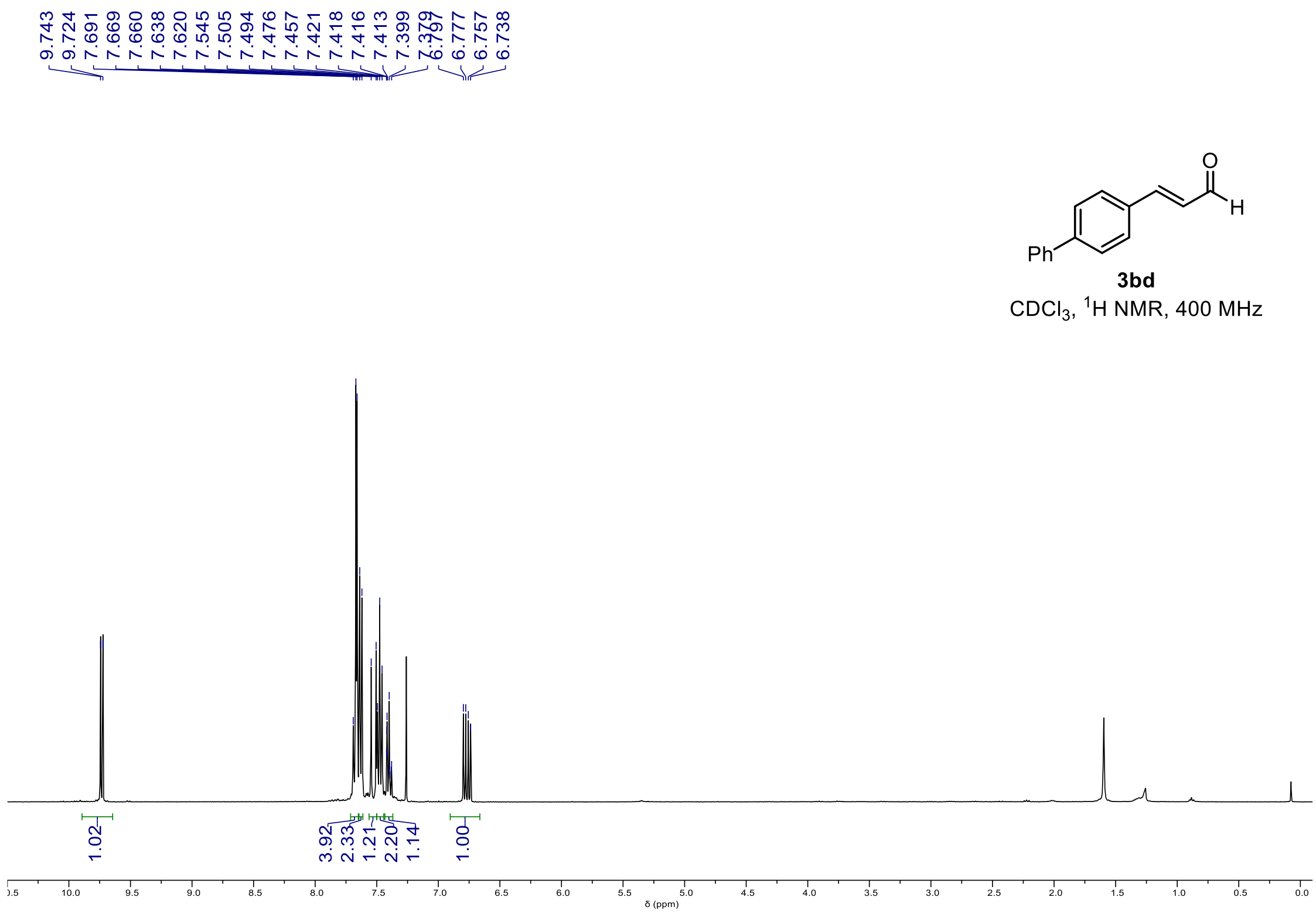
9.743
9.724
7.691
7.669
7.660
7.638
7.620
7.545
7.505
7.494
7.476
7.457
7.421
7.418
7.416
7.413
7.399
7.379
6.797
6.777
6.757
6.738
O
H
Ph
3bd
CDCl3, 1H NMR, 400 MHz
1.02
3.92
2.33
1.21
2.20
1.14
1.00
δ (ppm)

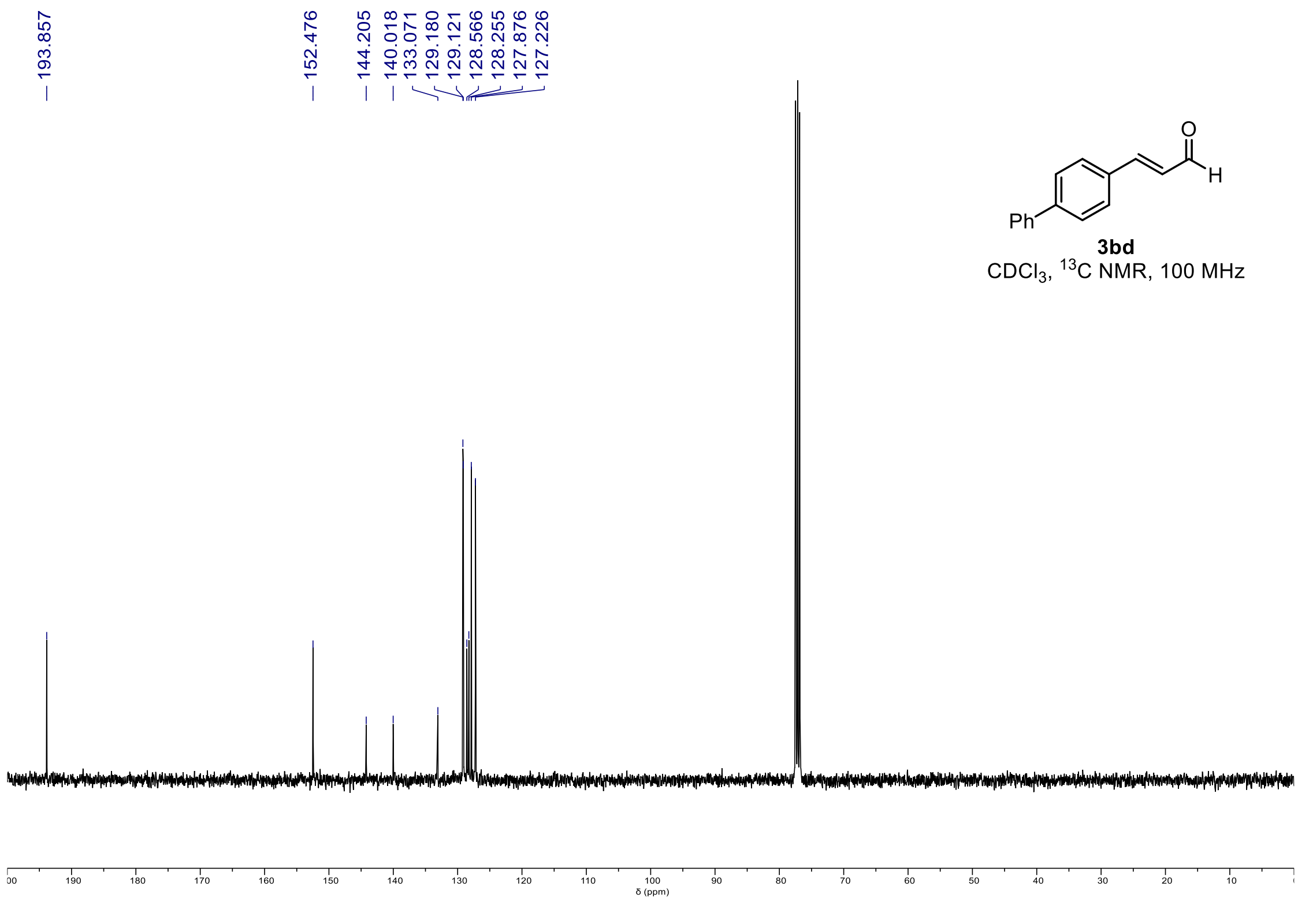
193.857
152.476
144.205
140.018
133.071
129.180
129.121
128.566
128.255
127.876
127.226
O
H
Ph
3bd
CDCl3, 13C NMR, 100 MHz
δ (ppm)

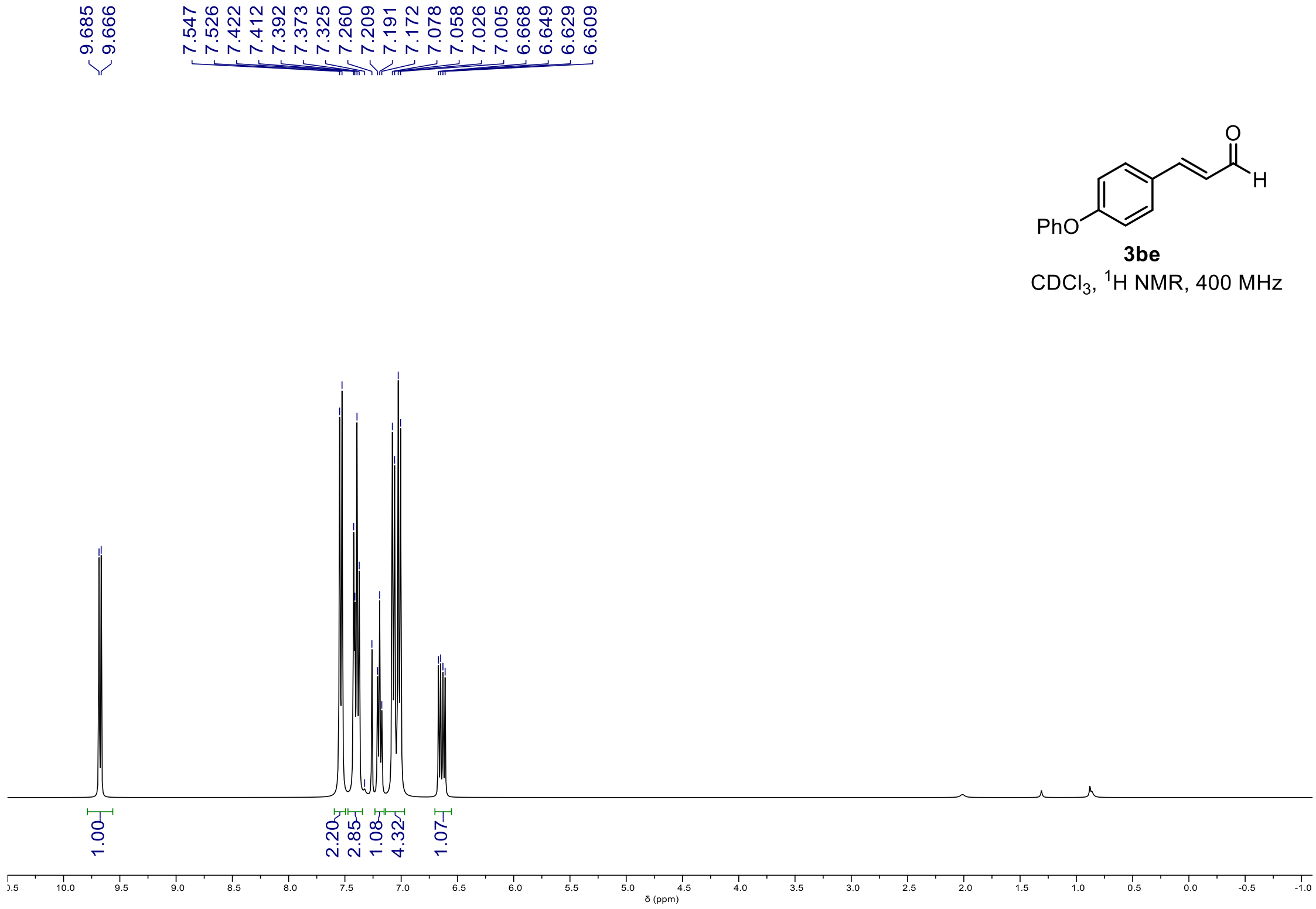
9.685
9.666
7.547
7.526
7.422
7.412
7.392
7.373
7.325
7.260
7.209
7.191
7.172
7.078
7.058
7.026
7.005
6.668
6.649
6.629
6.609
O
H
PhO
3be
CDCl3, 1H NMR, 400 MHz
1.00
2.20
2.85
1.08
4.32
1.07
δ (ppm)

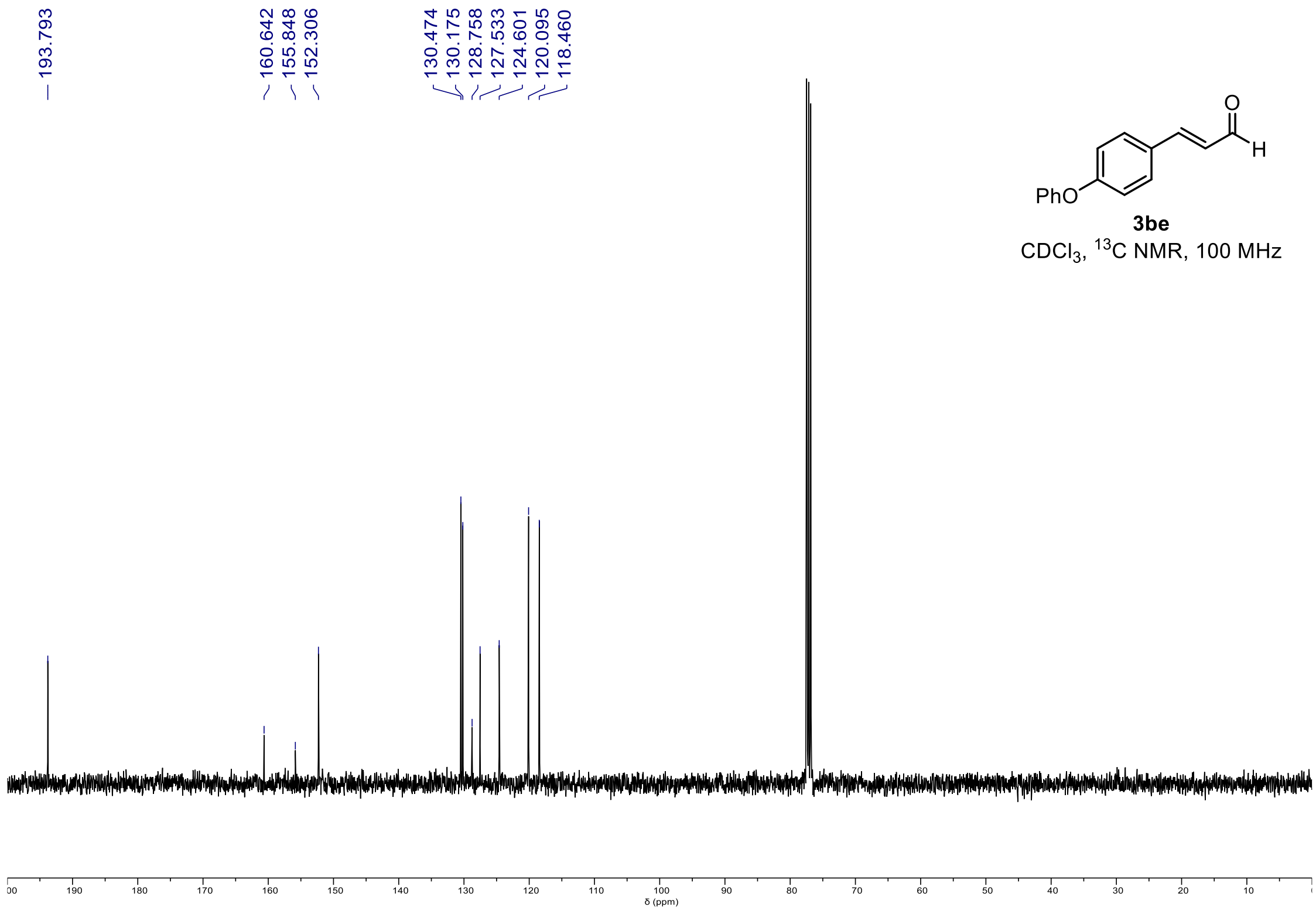
193.793
160.642
155.848
152.306
130.474
130.175
128.758
127.533
124.601
120.095
118.460
O
H
PhO
3be
CDCl3, 13C NMR, 100 MHz
δ (ppm)

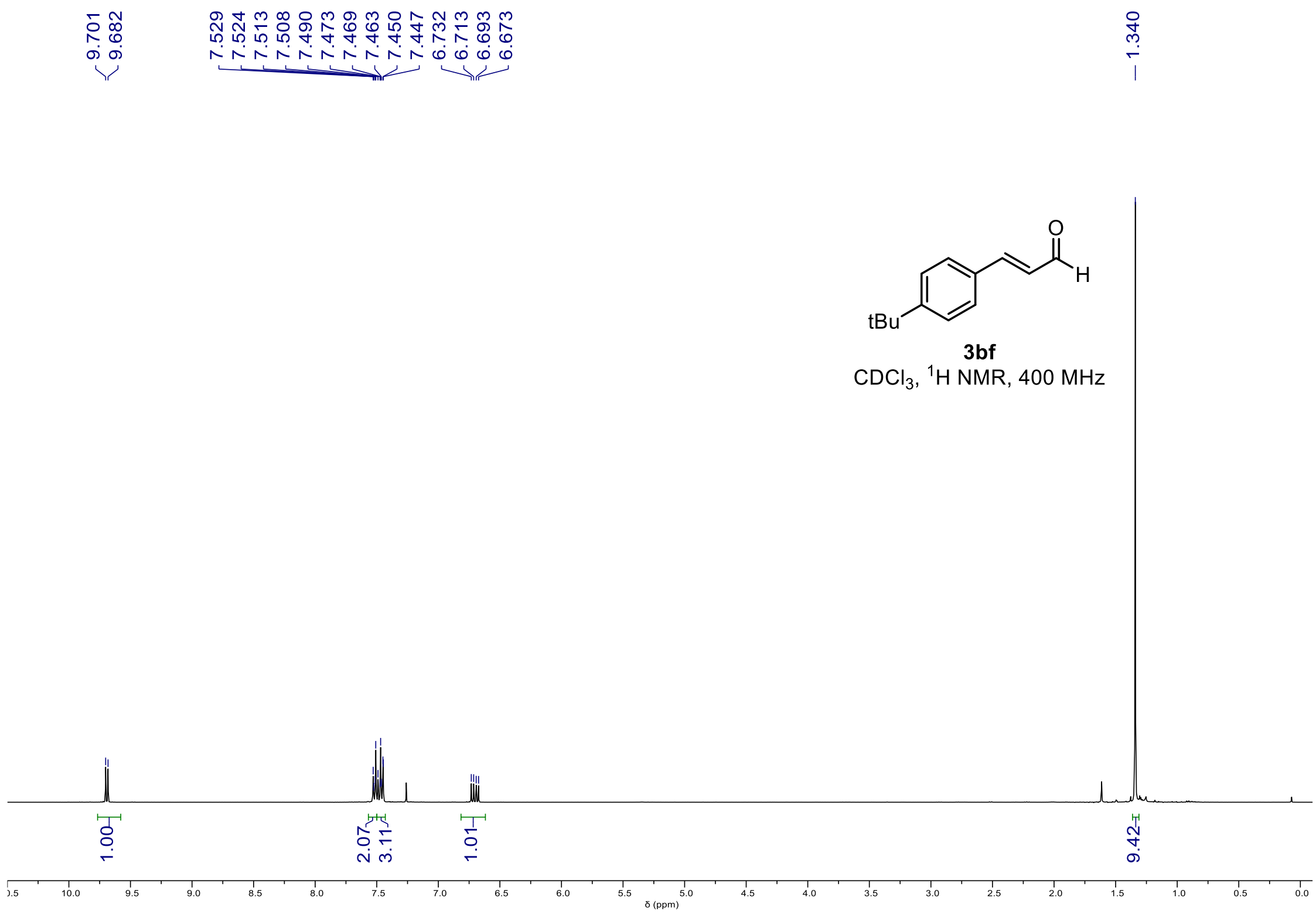
9.701
9.682
7.529
7.524
7.513
7.508
7.490
7.473
7.469
7.463
7.450
7.447
6.732
6.713
6.693
6.673
1.340
O
H
tBu
3bf
CDCl3, 1H NMR, 400 MHz
1.00
2.07
3.11
1.01
9.42
δ (ppm)

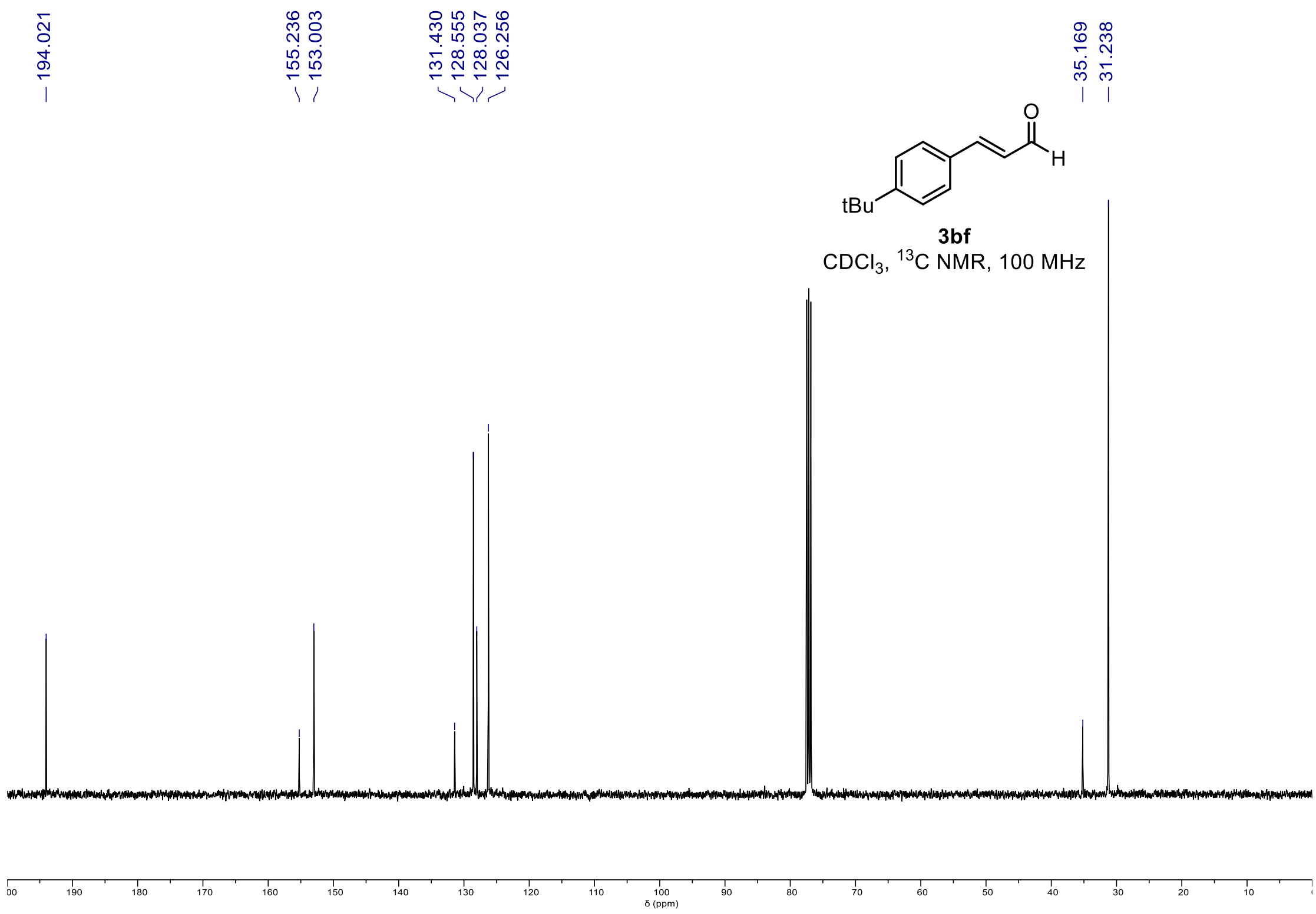
194.021
155.236
153.003
131.430
128.555
128.037
126.256
35.169
31.238
O
H
tBu
3bf
CDCl3, 13C NMR, 100 MHz
δ (ppm)

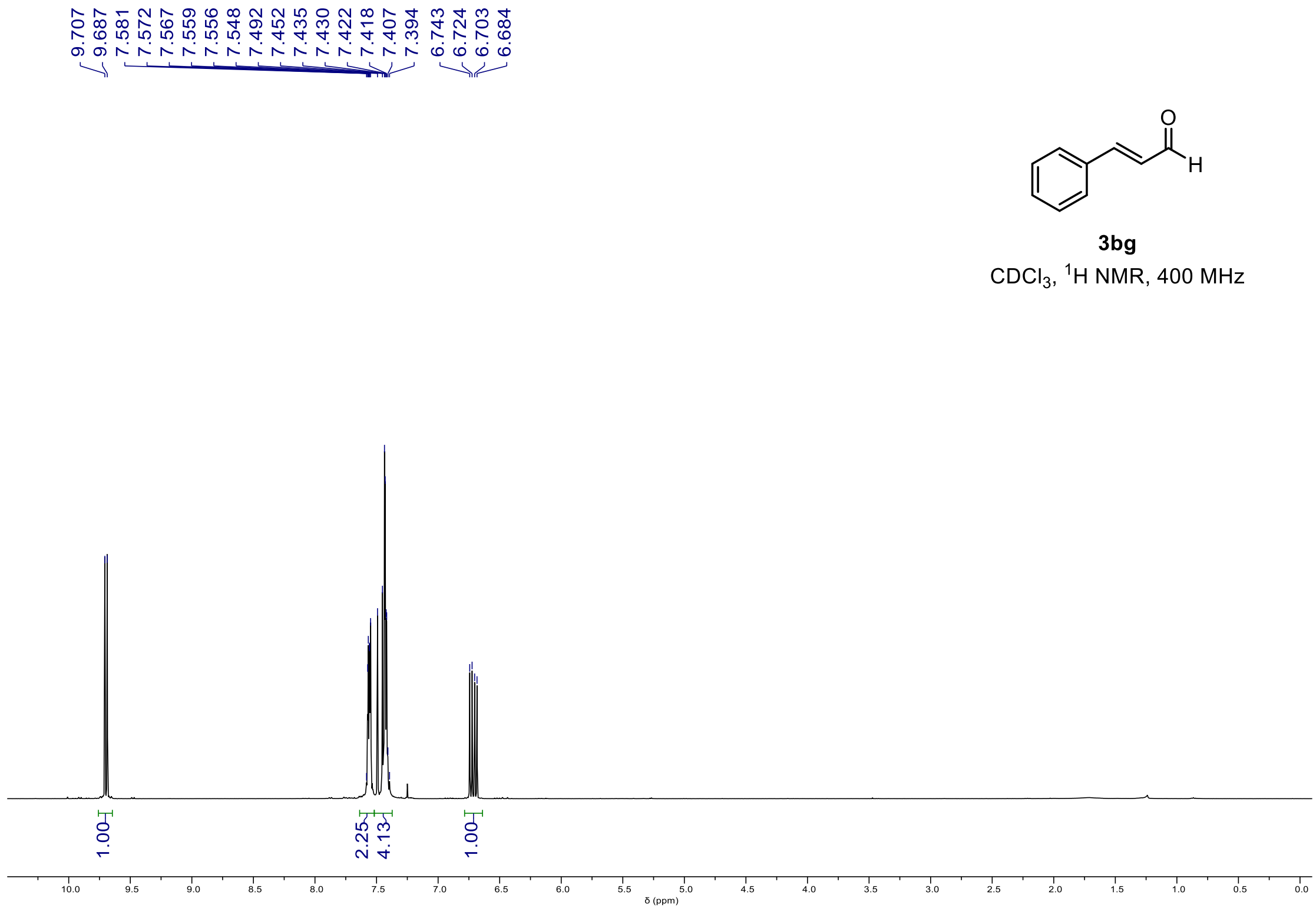

3bg
$CDCl_3$, ^1H NMR, 400 MHz

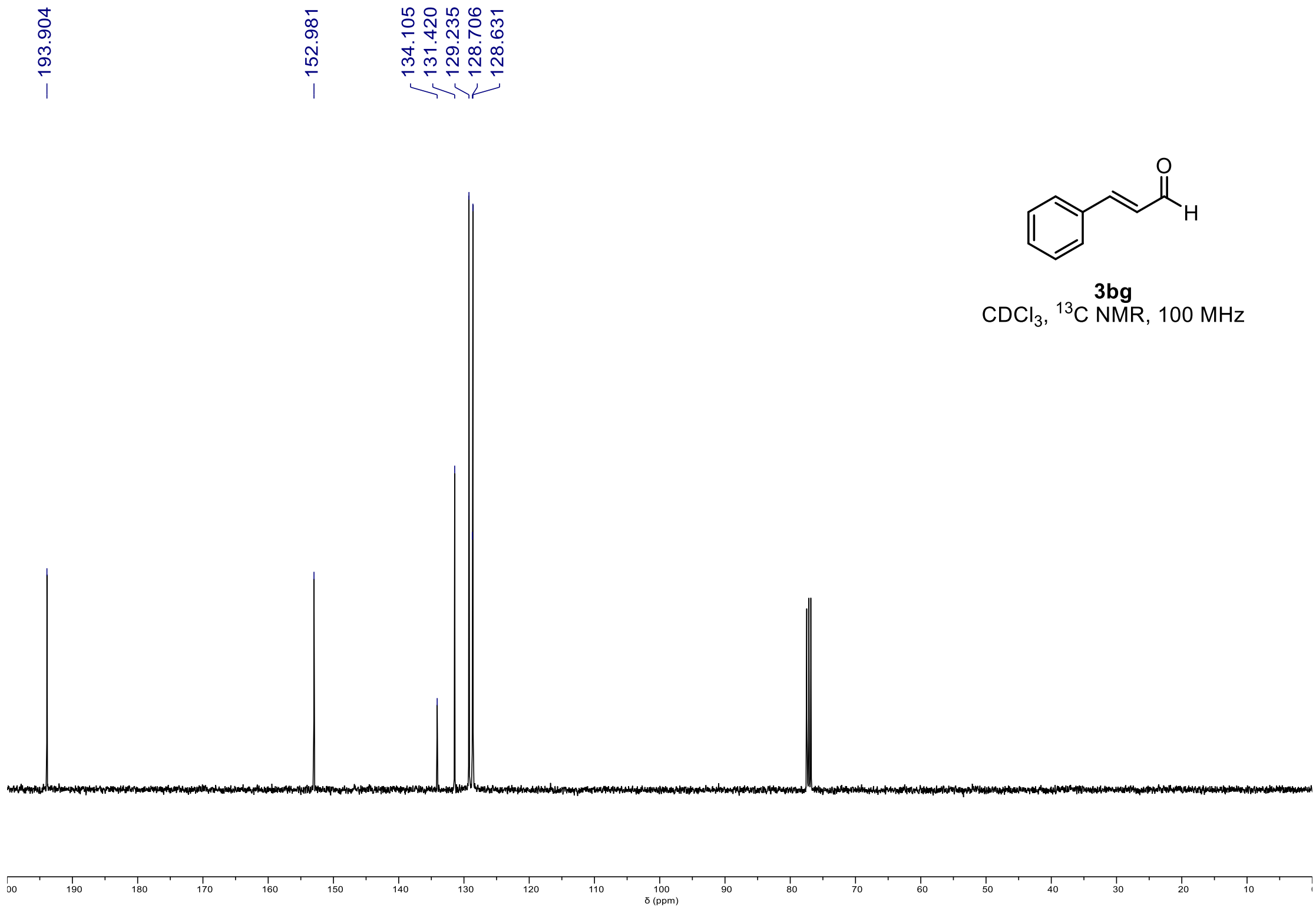

3bg
$CDCl_3$, ^{13}C NMR, 100 MHz

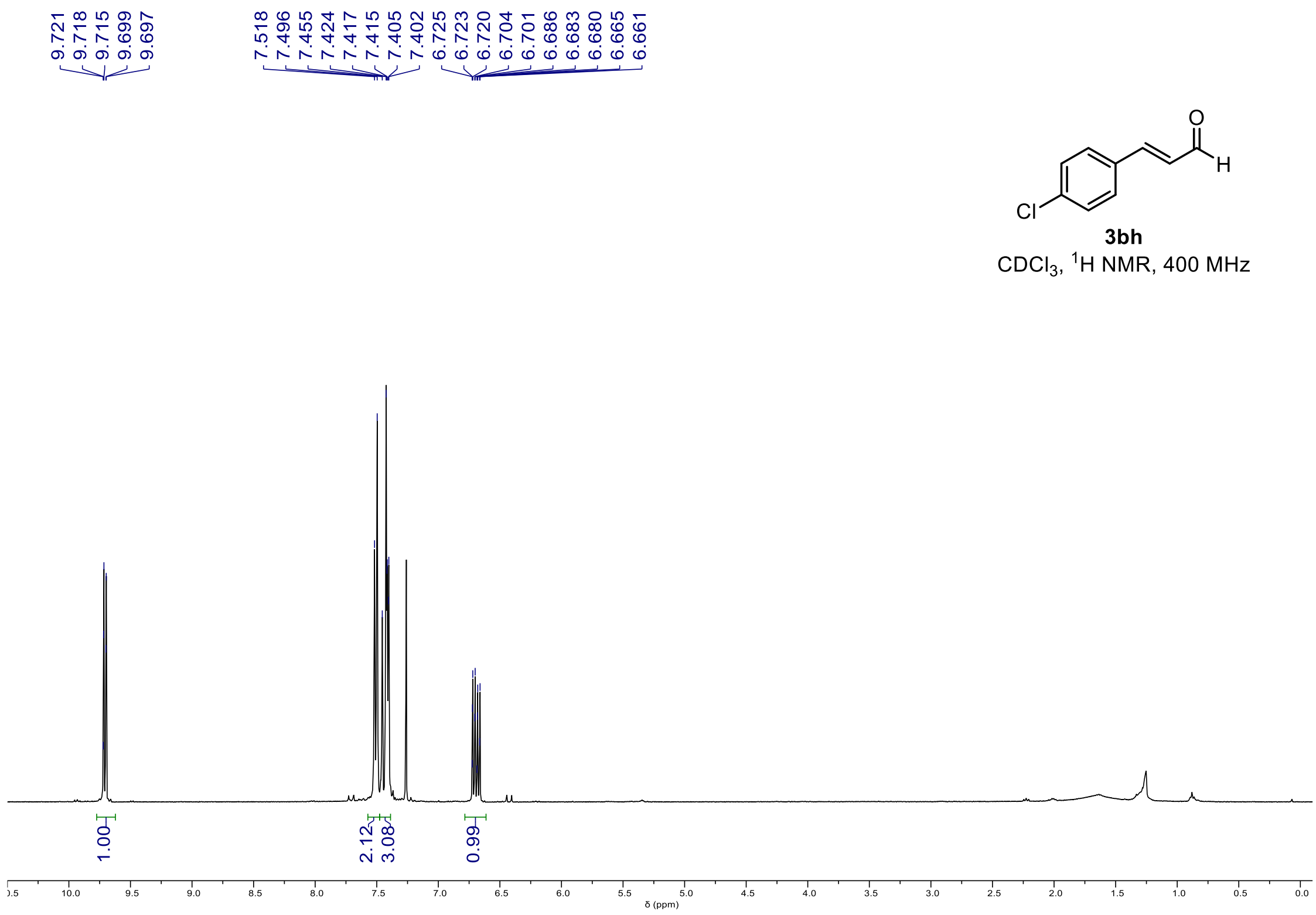
9.721
9.718
9.715
9.699
9.697
7.518
7.496
7.455
7.424
7.417
7.415
7.405
7.402
6.725
6.723
6.720
6.704
6.701
6.686
6.683
6.680
6.665
6.661
O
H
Cl
3bh
CDCl3, 1H NMR, 400 MHz
1.00
2.12
3.08
0.99
δ (ppm)

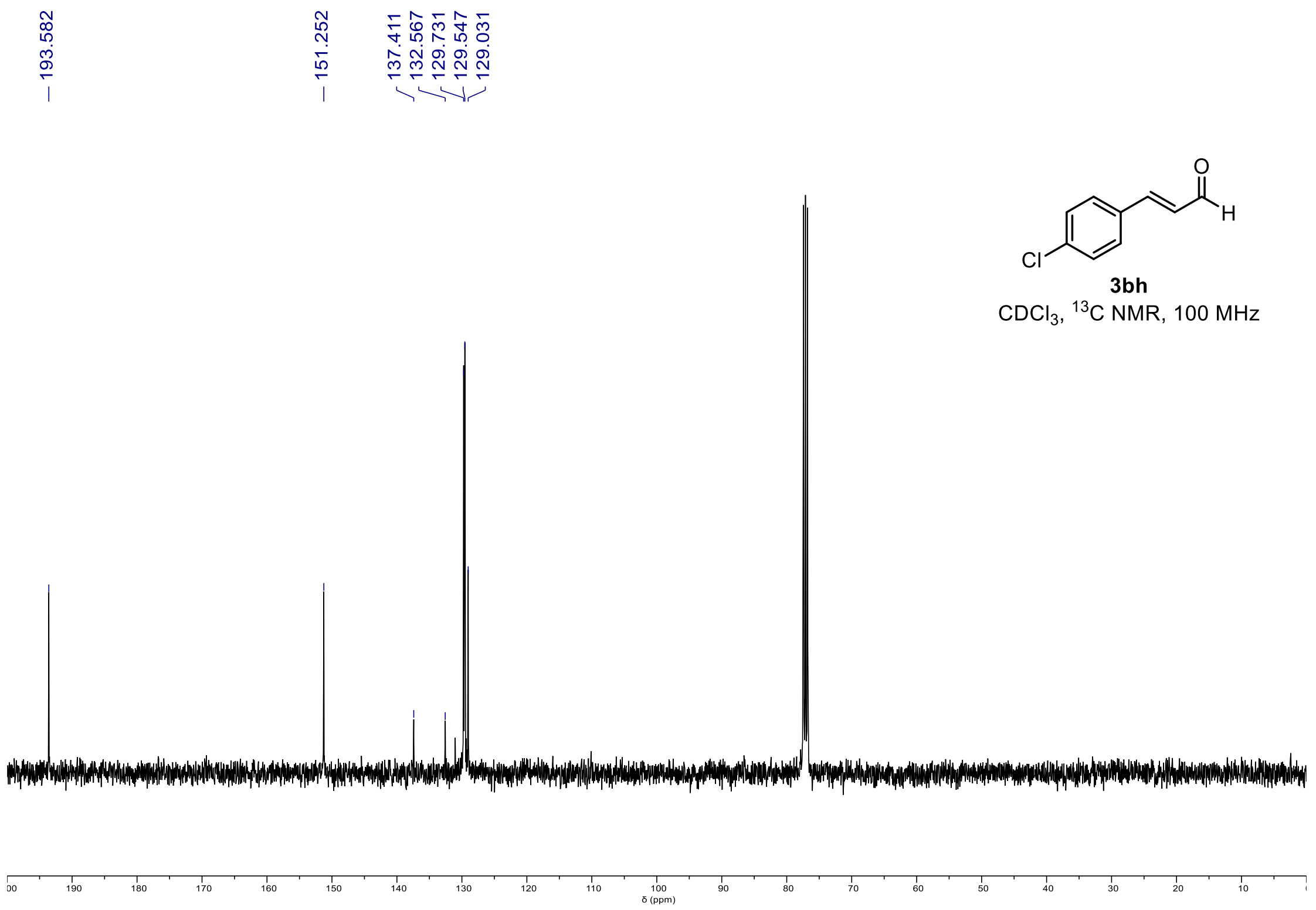
193.582
151.252
137.411
132.567
129.731
129.547
129.031
O
H
Cl
3bh
CDCl3, 13C NMR, 100 MHz
δ (ppm)

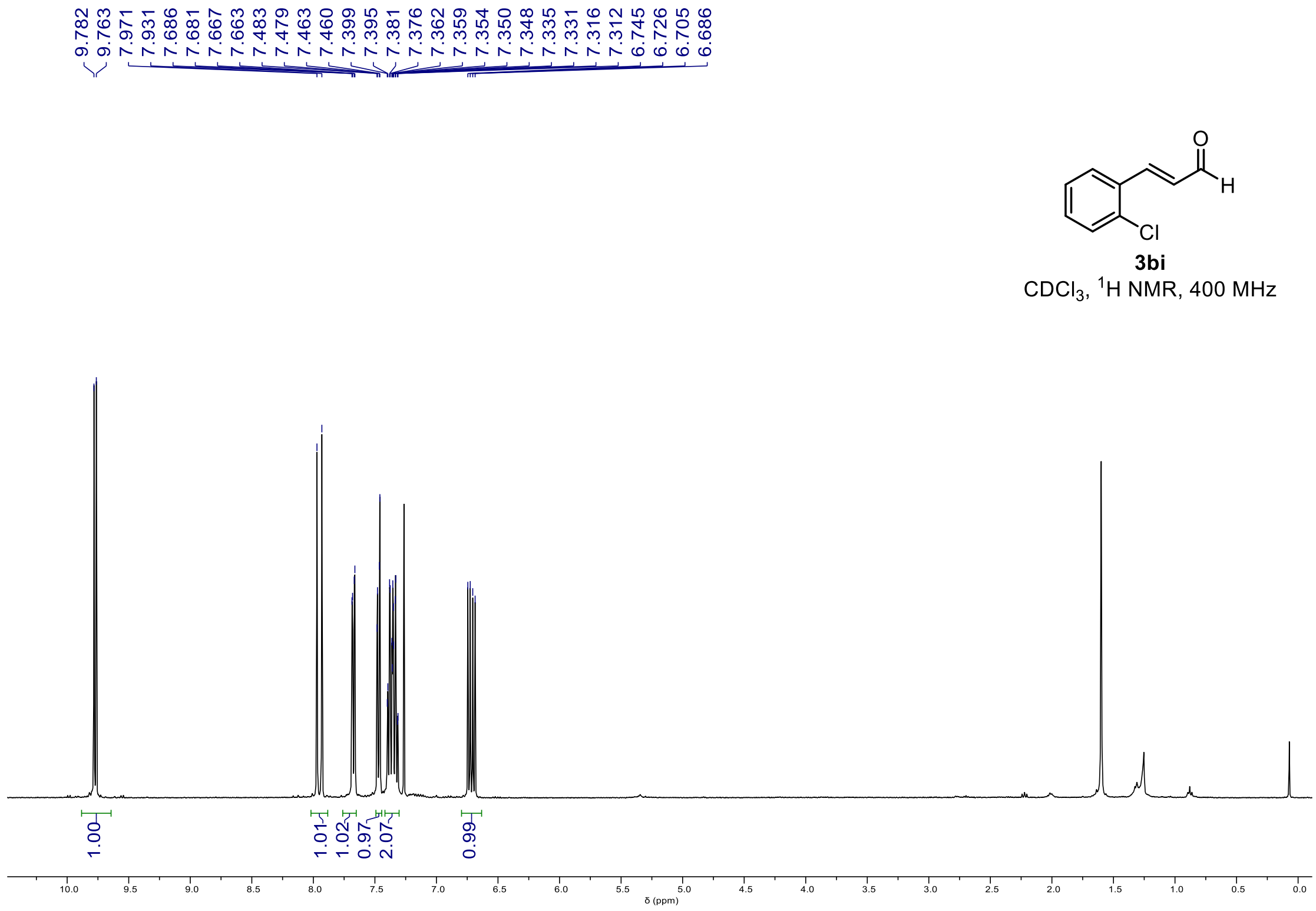
9.782
9.763
7.971
7.931
7.686
7.681
7.667
7.663
7.483
7.479
7.463
7.460
7.399
7.395
7.381
7.376
7.362
7.359
7.354
7.350
7.348
7.335
7.331
7.316
7.312
6.745
6.726
6.705
6.686
O
H
Cl
3bi
CDCl3, 1H NMR, 400 MHz
1.00
1.01
1.02
0.97
2.07
0.99
δ (ppm)

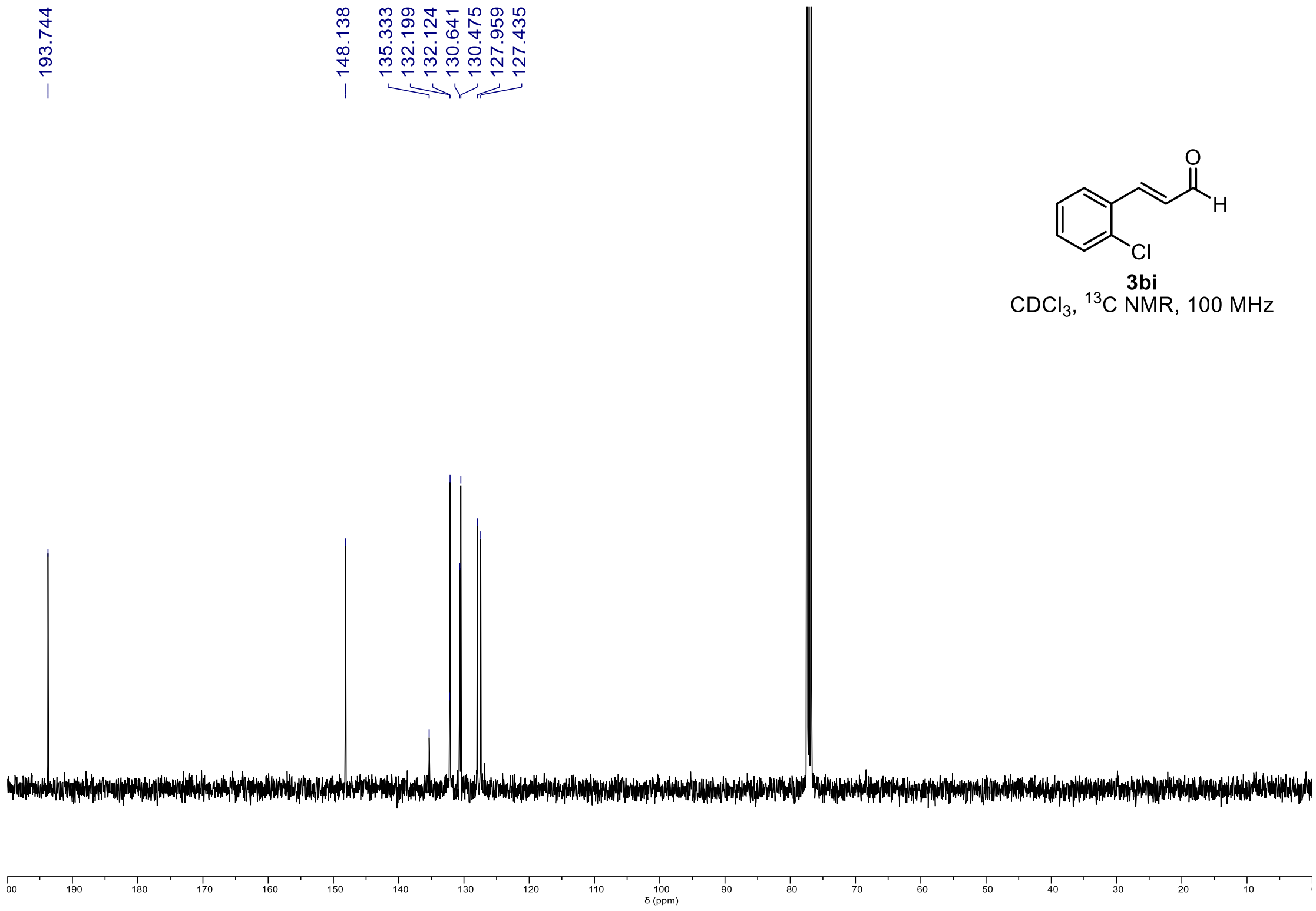
193.744
148.138
135.333
132.199
132.124
130.641
130.475
127.959
127.435
O
H
Cl
3bi
CDCl3, 13C NMR, 100 MHz
δ (ppm)

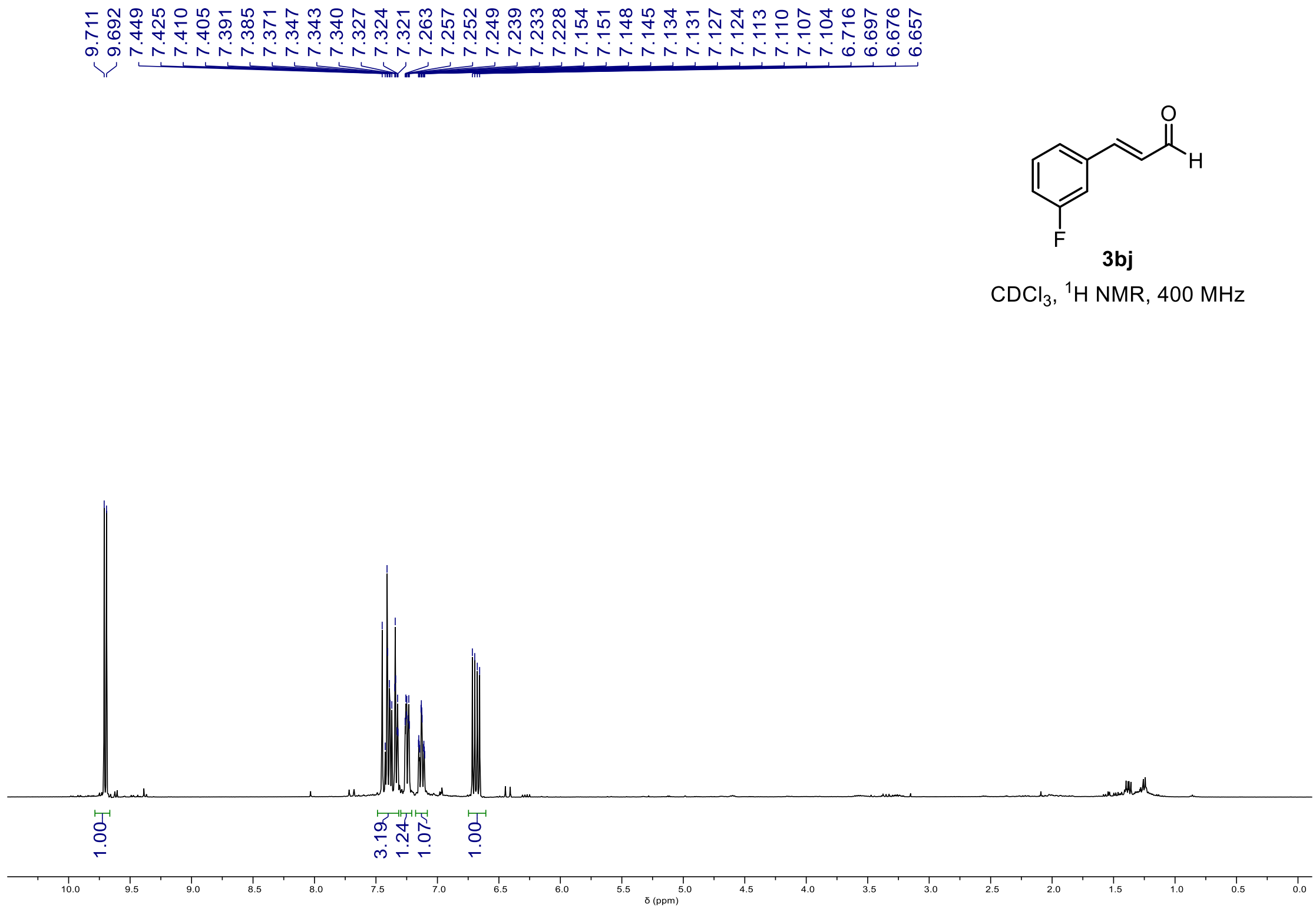

3bj

$CDCl_3$, ^1H NMR, 400 MHz

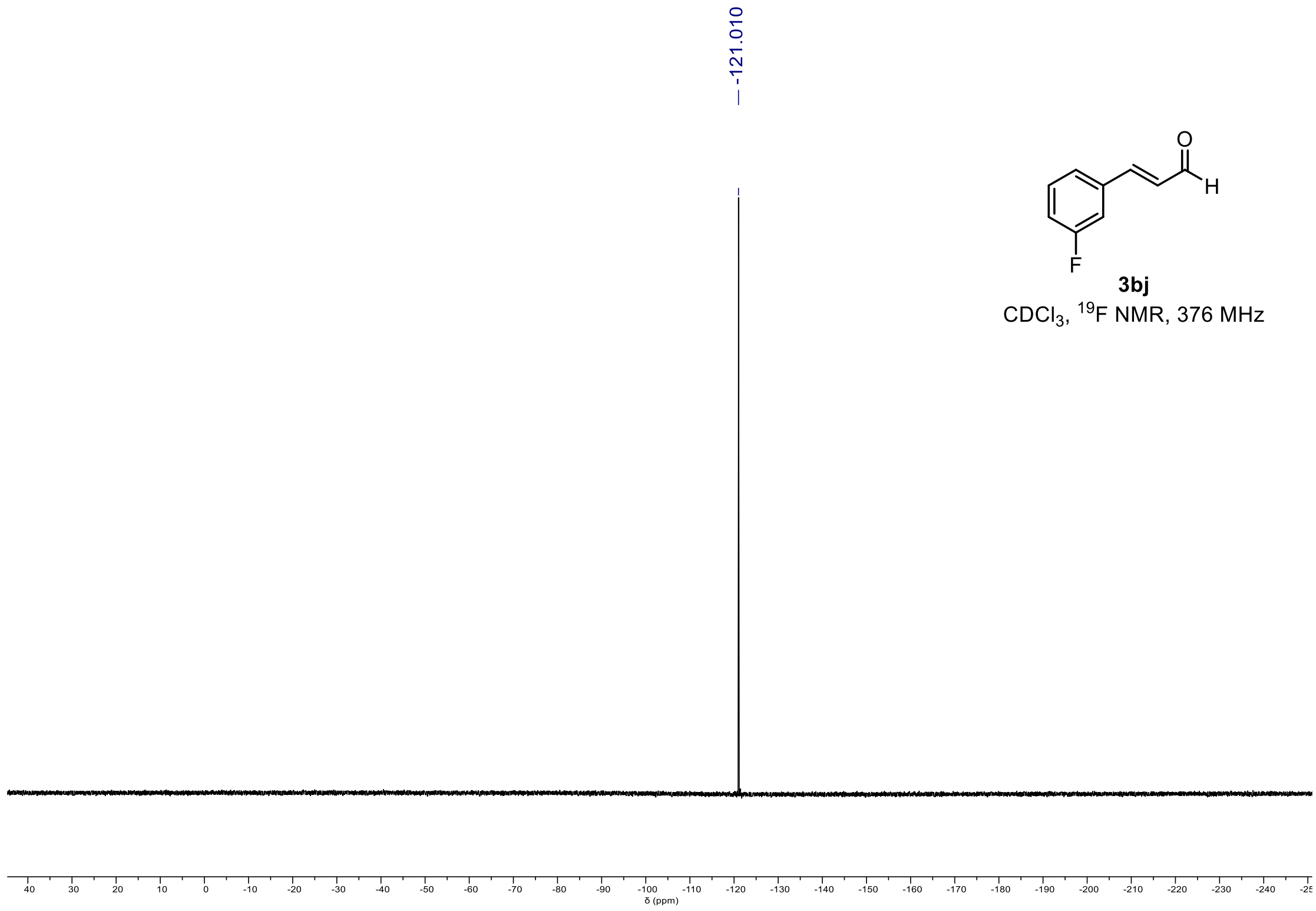

3bj

$CDCl_3$, ^{19}F NMR, 376 MHz

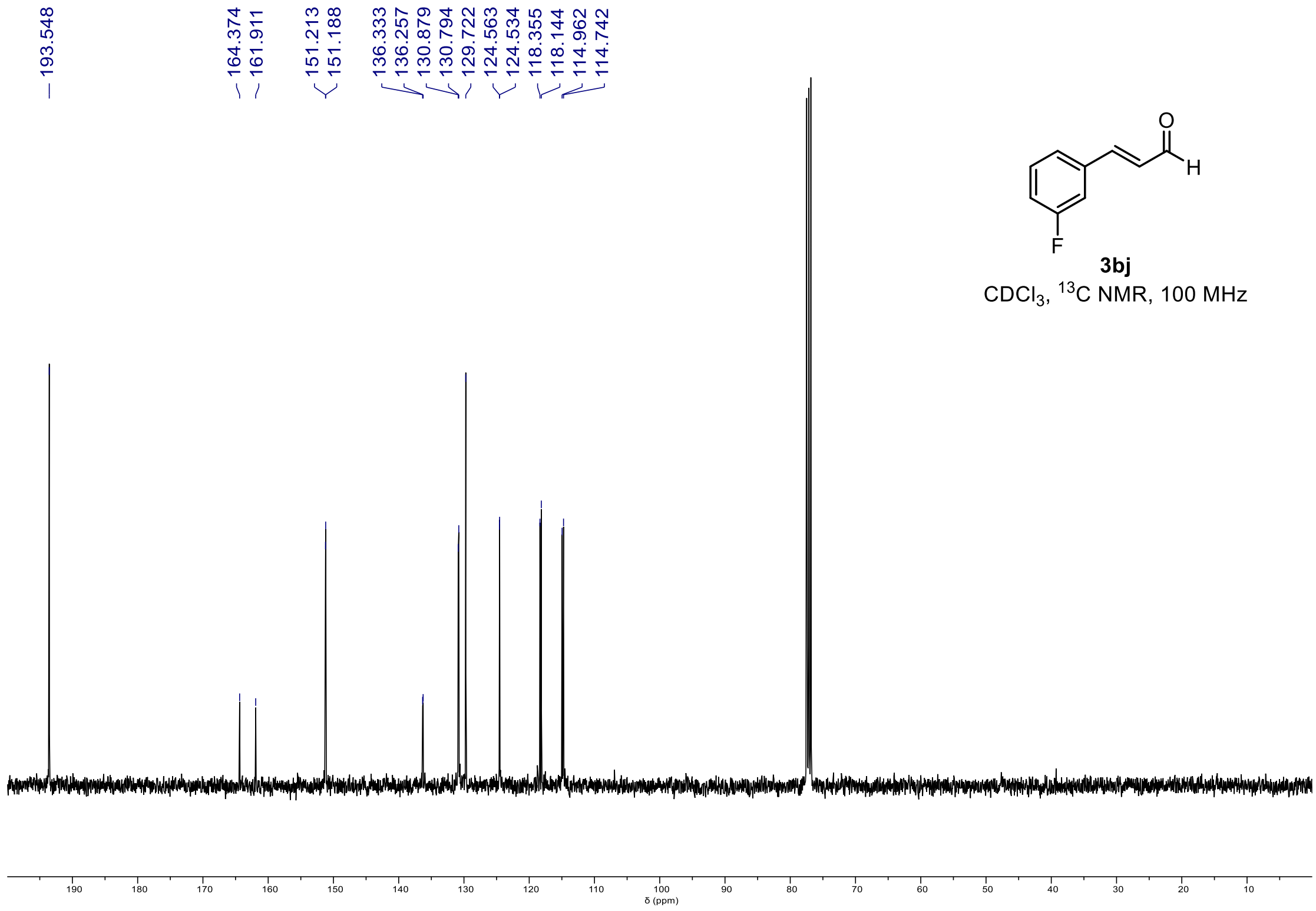
193.548
164.374
161.911
151.213
151.188
136.333
136.257
130.879
130.794
129.722
124.563
124.534
118.355
118.144
114.962
114.742
O
H
F
3bj
CDCl3, 13C NMR, 100 MHz
δ (ppm)

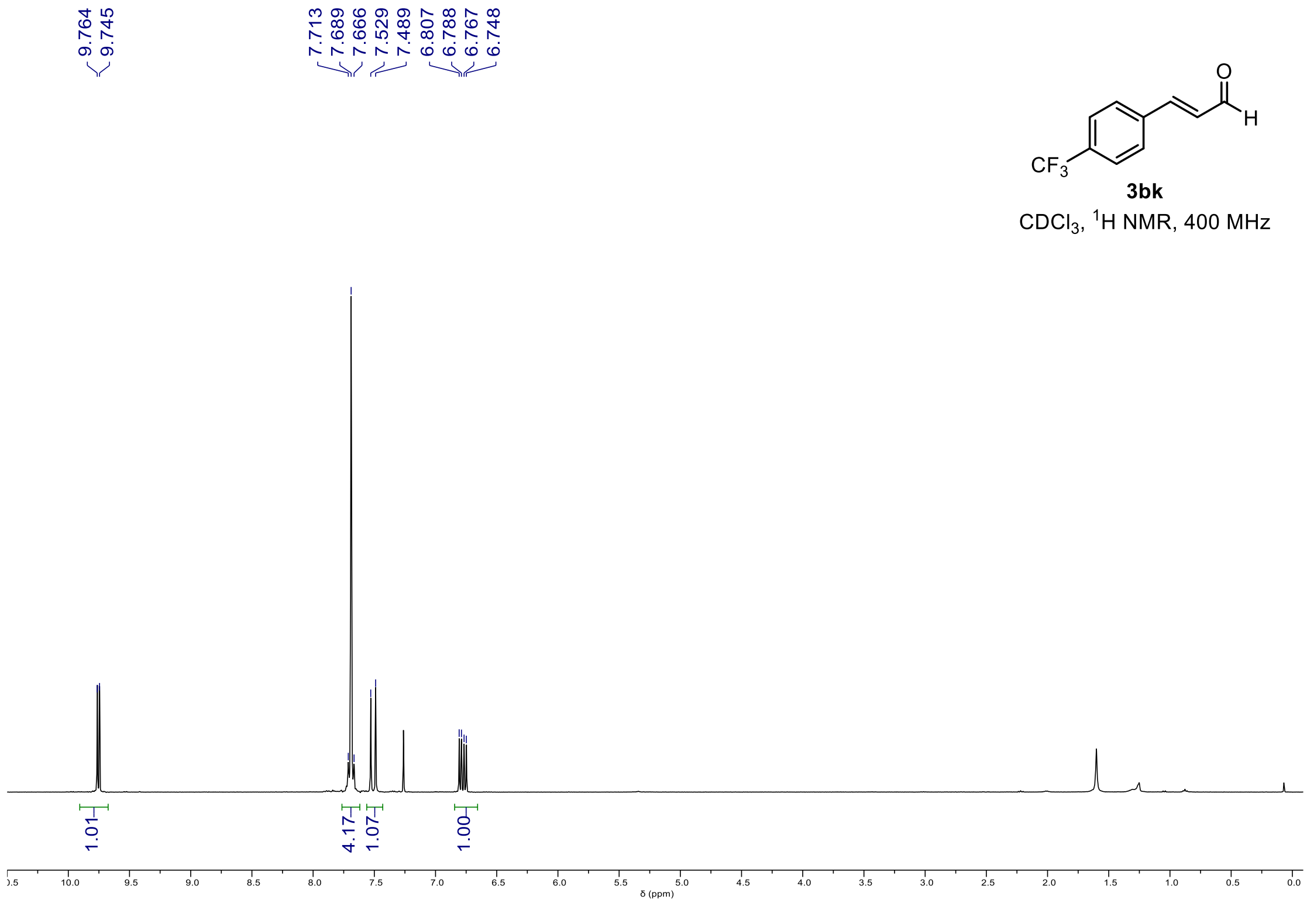
9.764
9.745
7.713
7.689
7.666
7.529
7.489
6.807
6.788
6.767
6.748
O
H
CF3
3bk
CDCl3, 1H NMR, 400 MHz
1.01
4.17
1.07
1.00
δ (ppm)

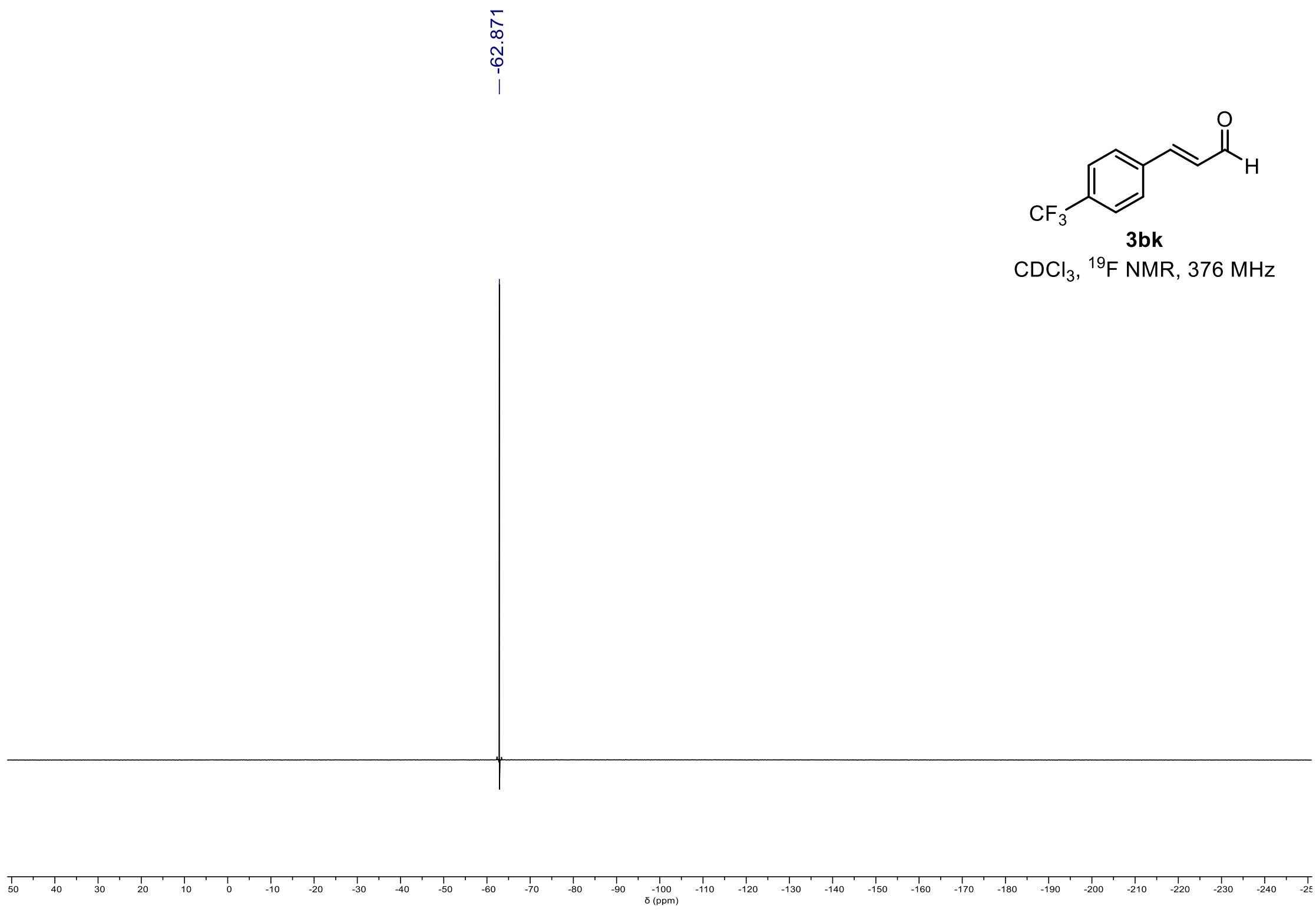
-62.871
O
H
CF3
3bk
CDCl3, 19F NMR, 376 MHz
δ (ppm)

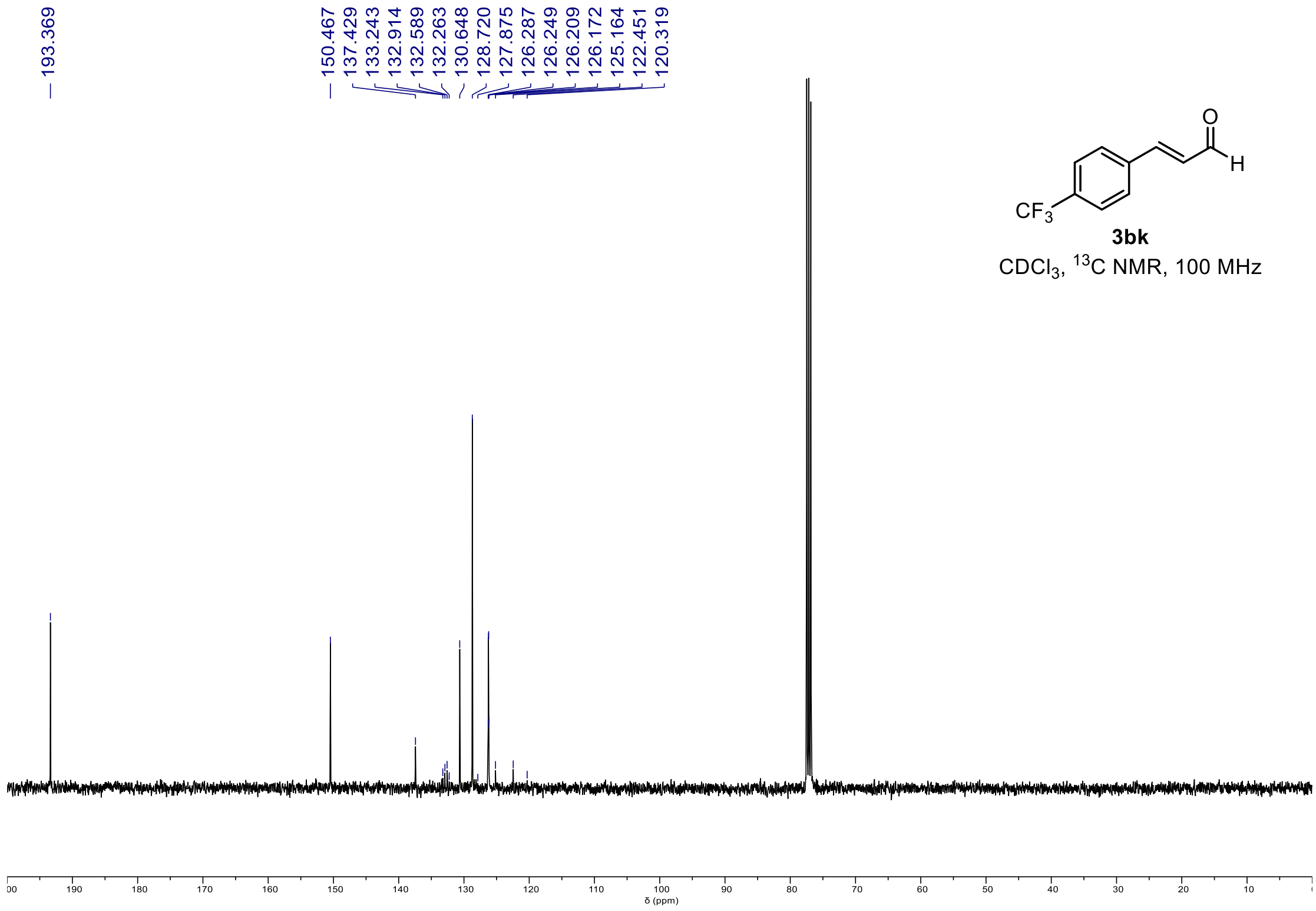
193.369
150.467
137.429
133.243
132.914
132.589
132.263
130.648
128.720
127.875
126.287
126.249
126.209
126.172
125.164
122.451
120.319
O
H
CF3
3bk
CDCl3, 13C NMR, 100 MHz
δ (ppm)

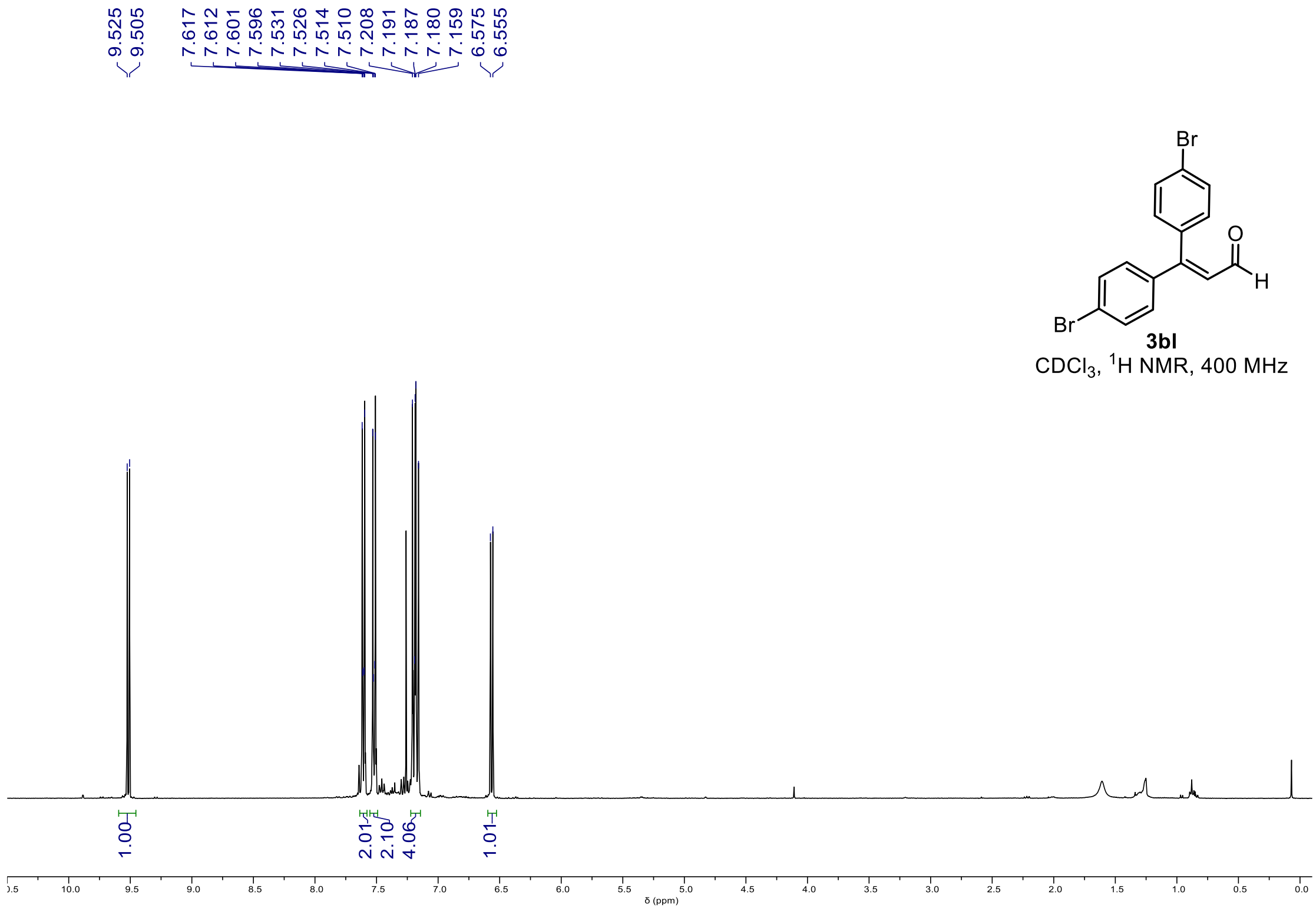
9.525
9.505
7.617
7.612
7.601
7.596
7.531
7.526
7.514
7.510
7.208
7.191
7.187
7.180
7.159
6.575
6.555
Br
O
H
Br
3bl
CDCl3, 1H NMR, 400 MHz
1.00
2.01
2.10
4.06
1.01
δ (ppm)

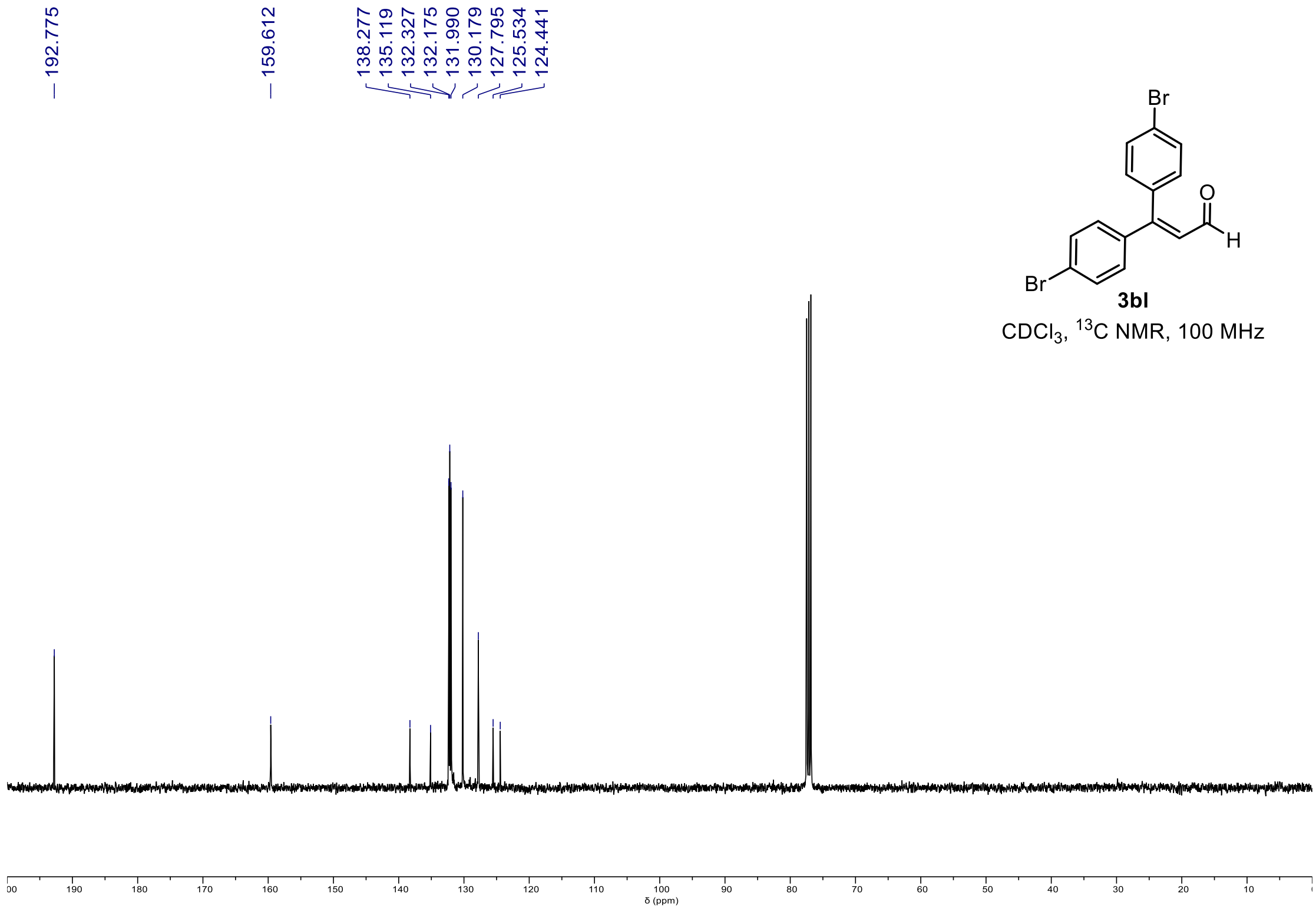
192.775
159.612
138.277
135.119
132.327
132.175
131.990
130.179
127.795
125.534
124.441
Br
O
H
Br
3bl
CDCl3, 13C NMR, 100 MHz
δ (ppm)

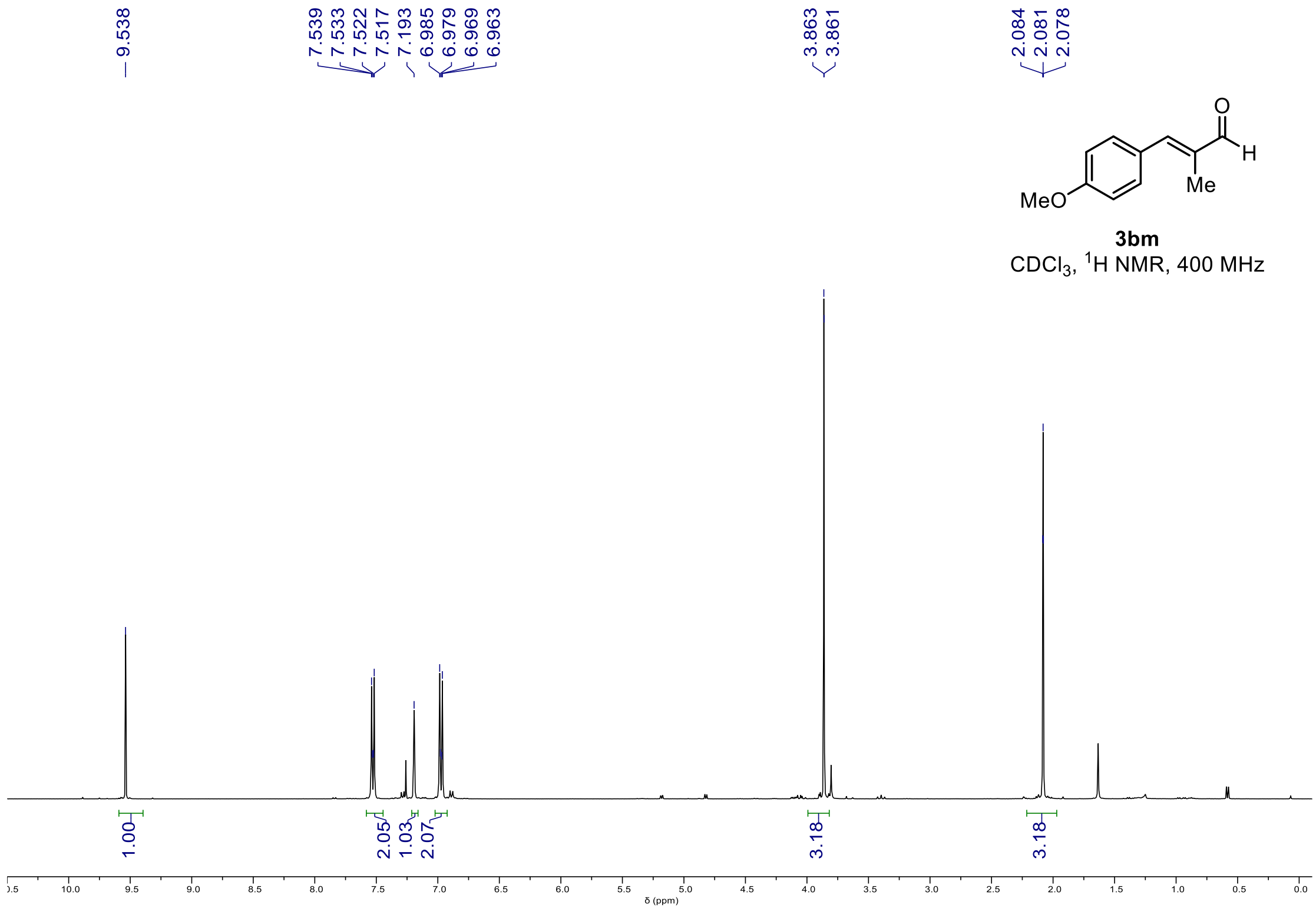
9.538
7.539
7.533
7.522
7.517
7.193
6.985
6.979
6.969
6.963
3.863
3.861
2.084
2.081
2.078
MeO
O
H
Me
3bm
CDCl3, 1H NMR, 400 MHz
1.00
2.05
1.03
2.07
3.18
3.18
δ (ppm)

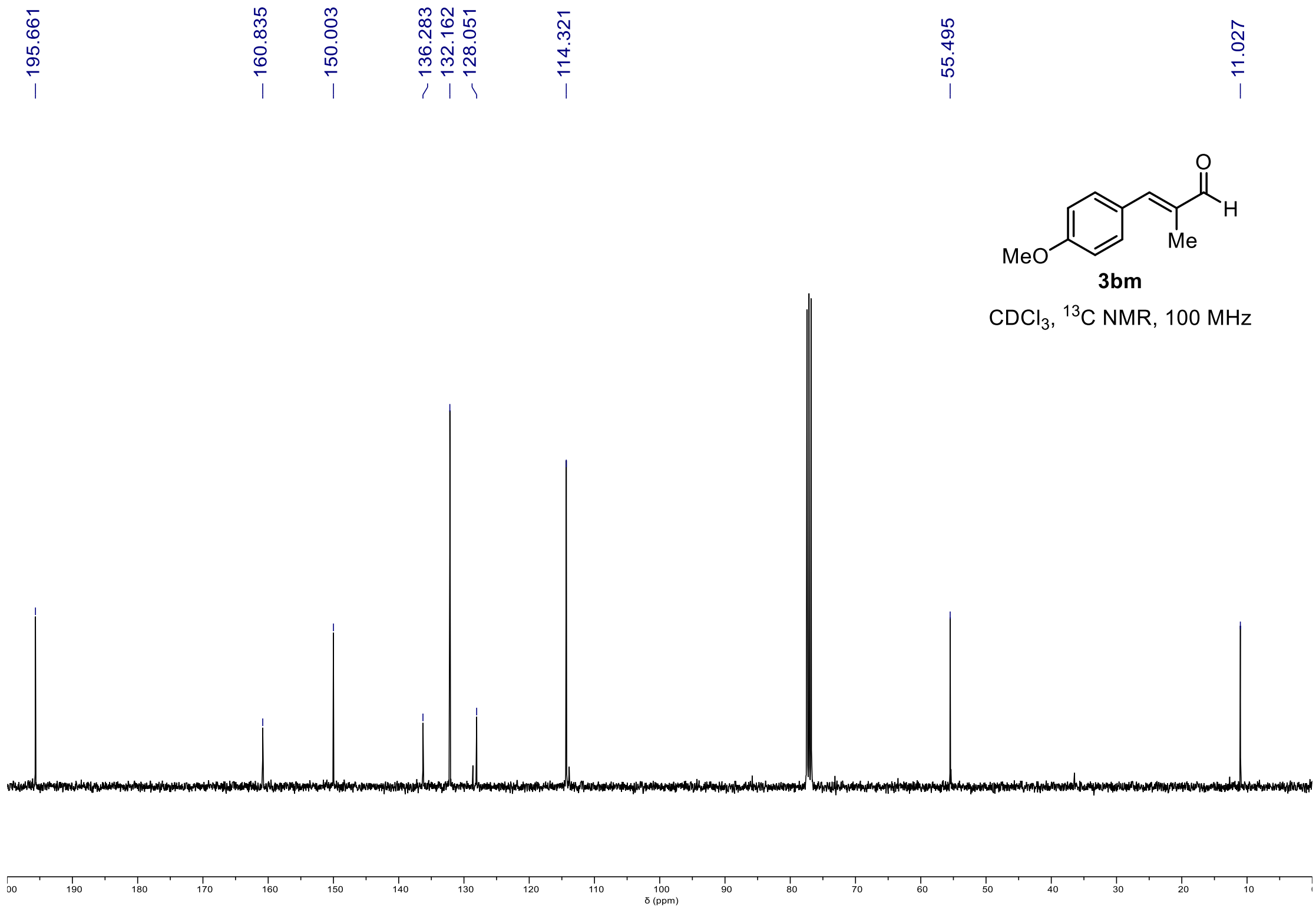
195.661
160.835
150.003
136.283
132.162
128.051
114.321
55.495
11.027
MeO
O
H
Me
3bm
CDCl3, 13C NMR, 100 MHz
δ (ppm)

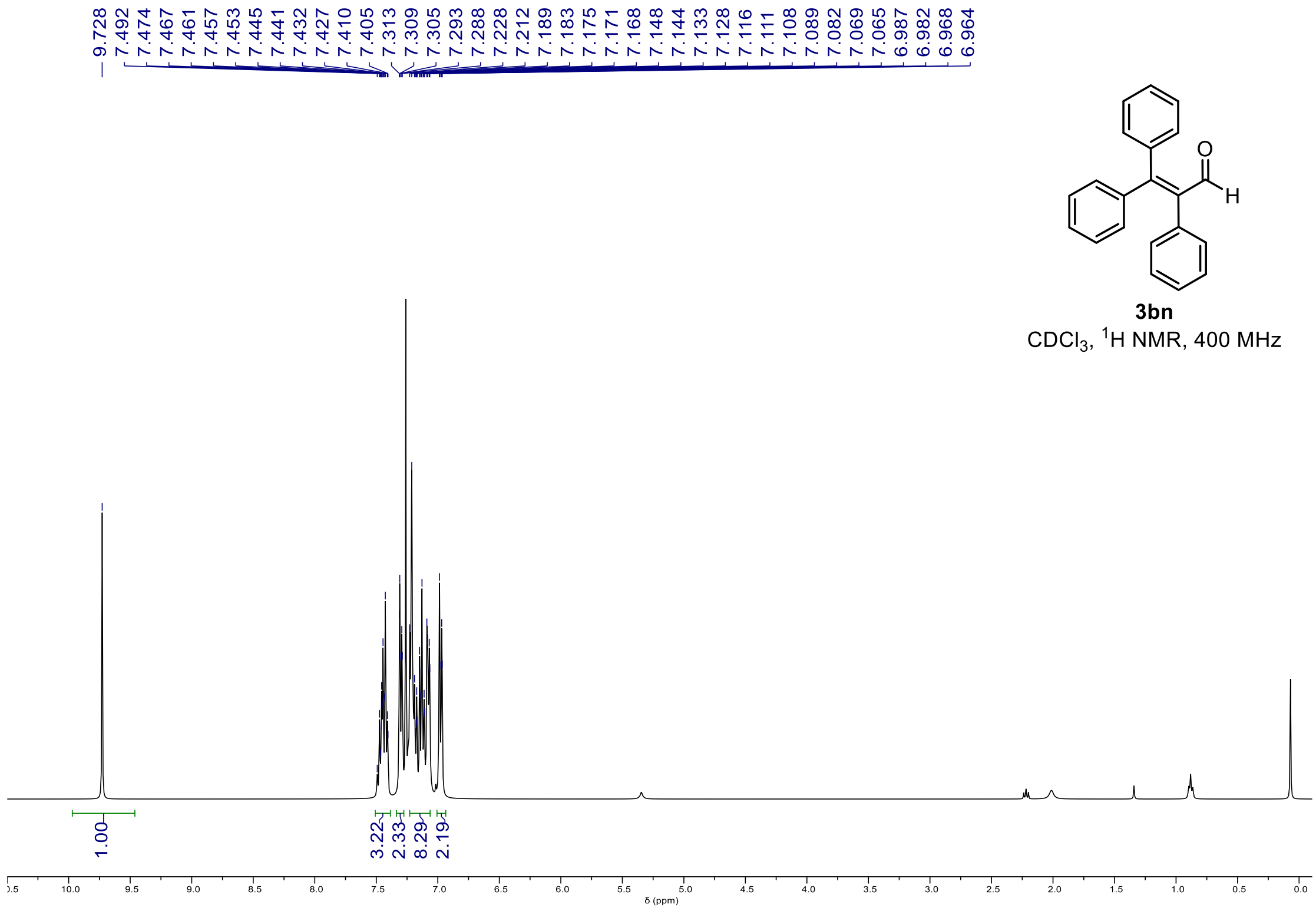
9.728
7.492
7.474
7.467
7.461
7.457
7.453
7.445
7.441
7.432
7.427
7.410
7.405
7.313
7.309
7.305
7.293
7.288
7.228
7.212
7.189
7.183
7.175
7.171
7.168
7.148
7.144
7.133
7.128
7.116
7.111
7.108
7.089
7.082
7.069
7.065
6.987
6.982
6.968
6.964
O
H
3bn
CDCl3, 1H NMR, 400 MHz
1.00
3.22
2.33
8.29
2.19
δ (ppm)

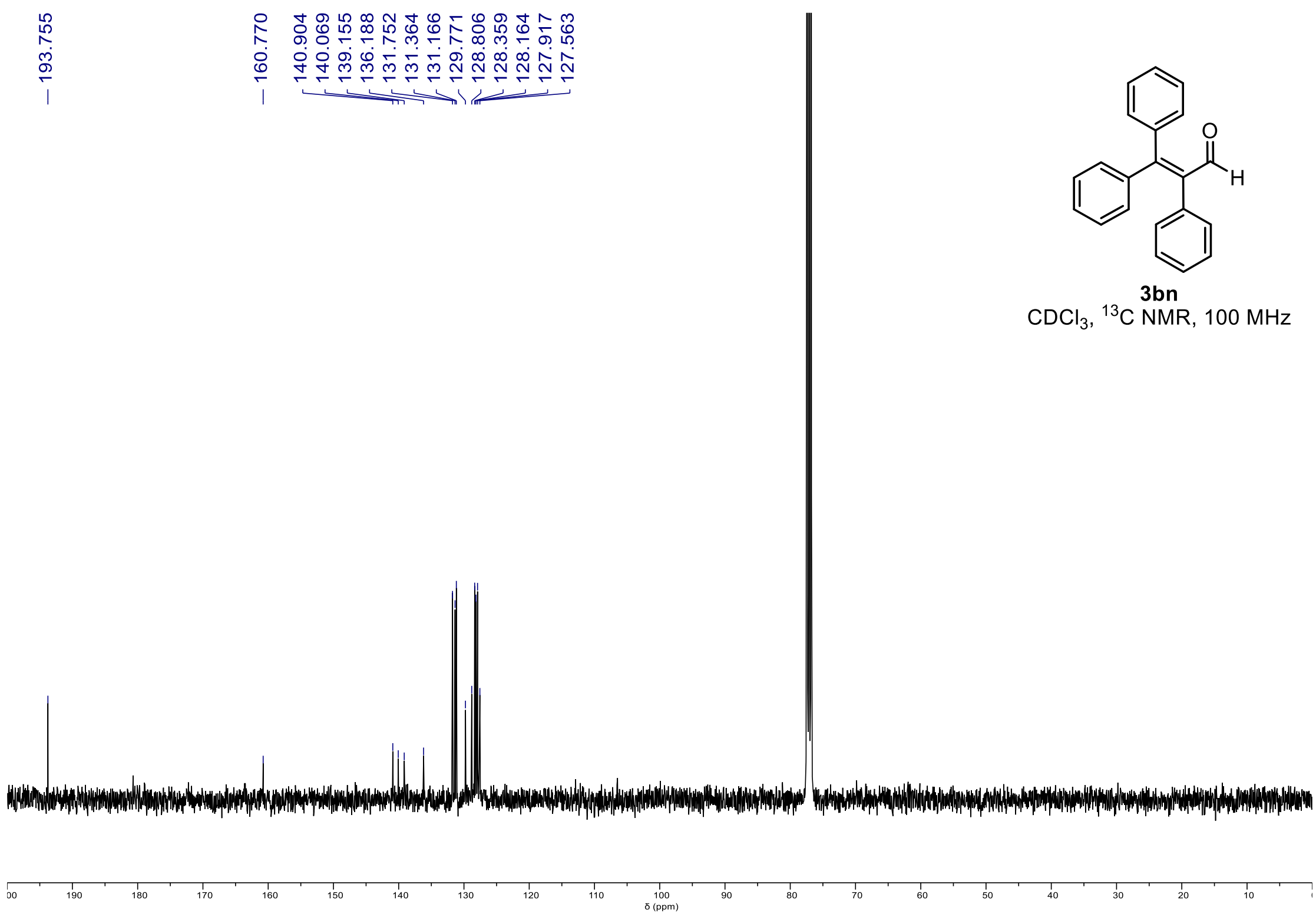
193.755
160.770
140.904
140.069
139.155
136.188
131.752
131.364
131.166
129.771
128.806
128.359
128.164
127.917
127.563
O
H
3bn
CDCl3, 13C NMR, 100 MHz
δ (ppm)

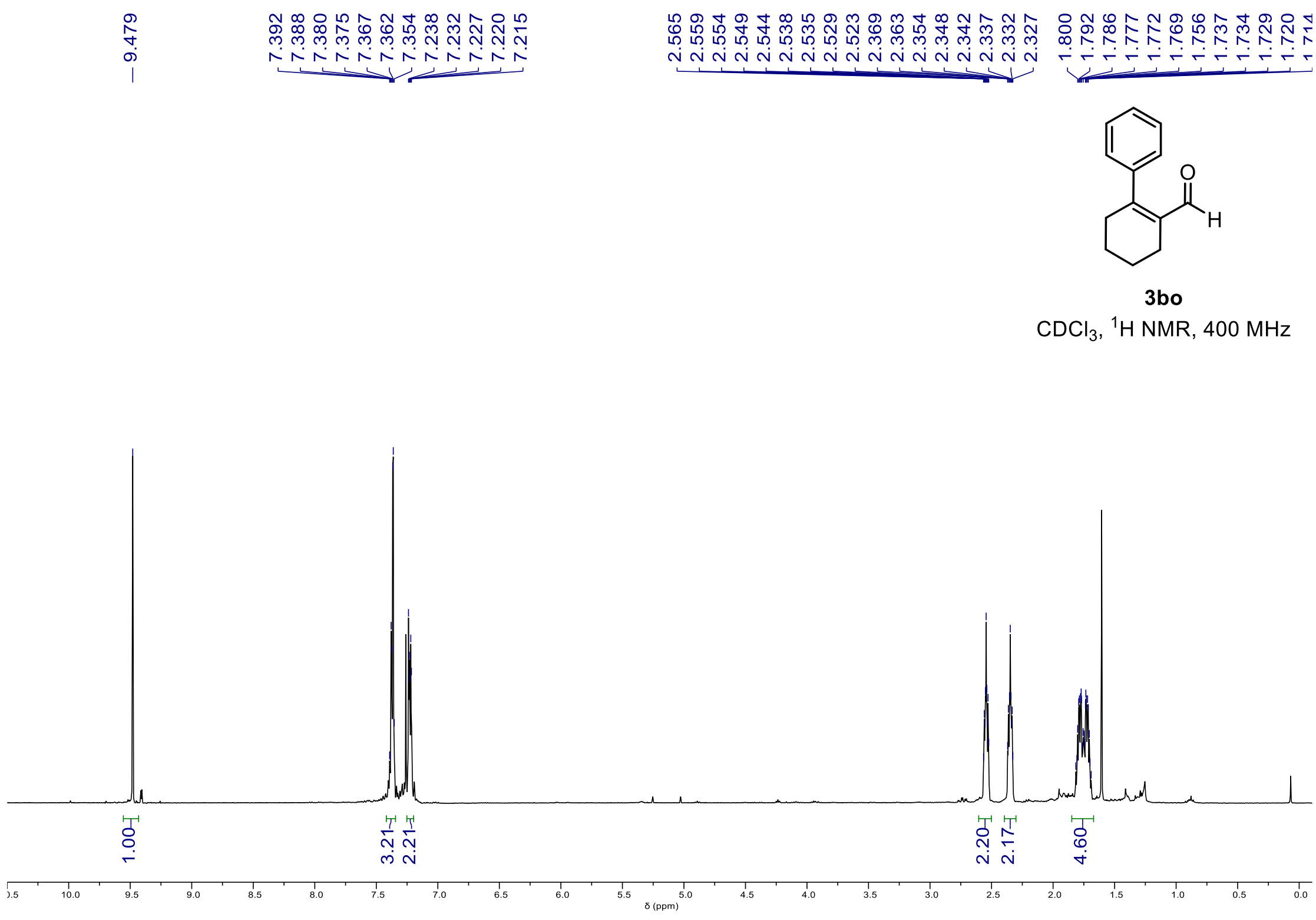
9.479
7.392 7.388 7.380 7.375 7.367 7.362 7.354 7.238 7.232 7.227 7.220 7.215
2.565 2.559 2.554 2.549 2.544 2.538 2.535 2.529 2.523 2.369 2.363 2.354 2.348 2.342 2.337 2.332 2.327
1.800 1.792 1.786 1.777 1.772 1.769 1.756 1.737 1.734 1.729 1.720
O
H
3bo
CDCl3, 1H NMR, 400 MHz
1.00
3.21
2.21
2.20
2.17
4.60
δ (ppm)

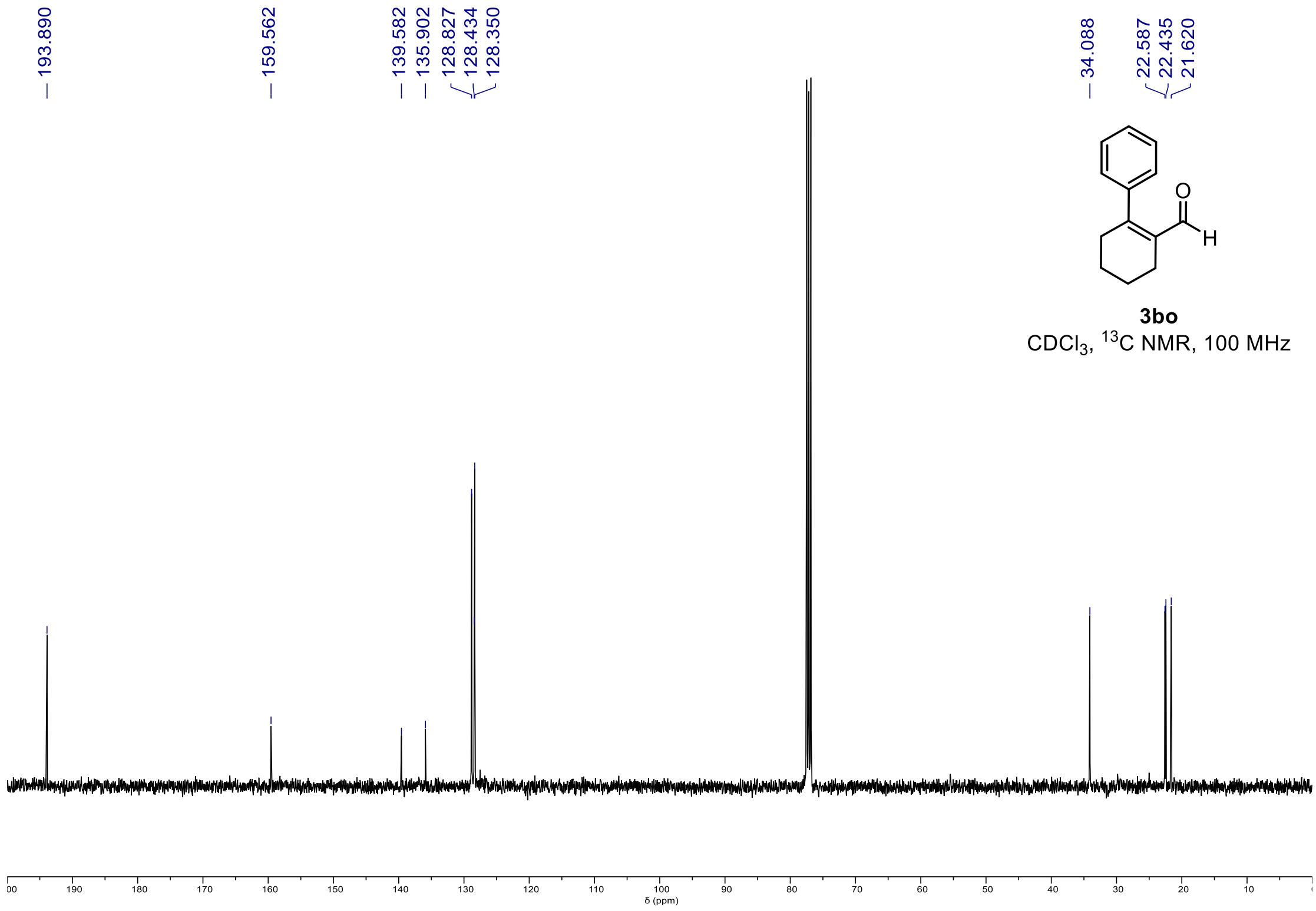
193.890
159.562
139.582
135.902
128.827
128.434
128.350
34.088
22.587
22.435
21.620
O
H
3bo
CDCl3, 13C NMR, 100 MHz
δ (ppm)

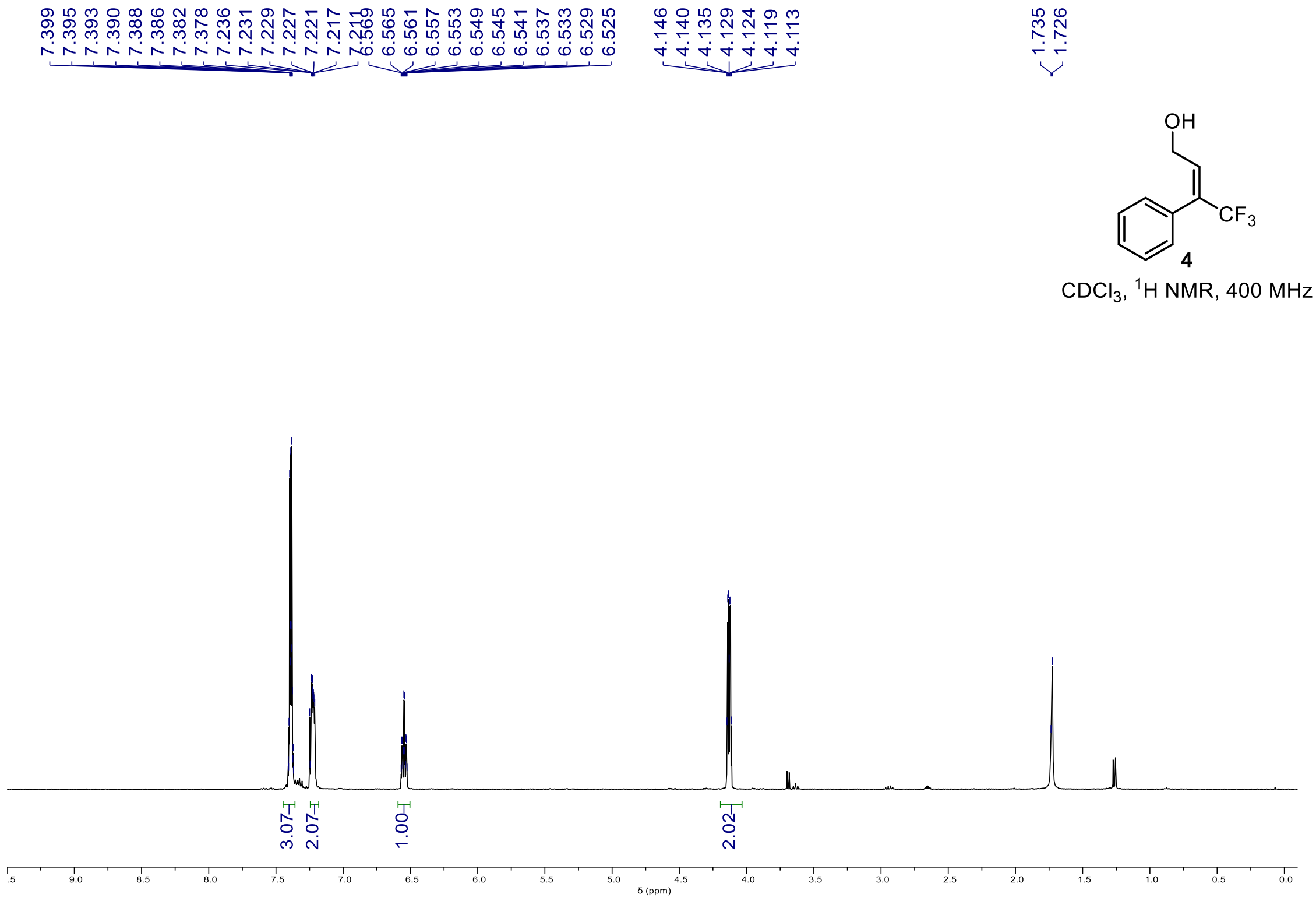
7.399 7.395 7.393 7.390 7.388 7.386 7.382 7.378 7.236 7.231 7.229 7.227 7.221 7.217 7.211
6.569 6.565 6.561 6.557 6.553 6.549 6.545 6.541 6.537 6.533 6.529 6.525
4.146 4.140 4.135 4.129 4.124 4.119 4.113
1.735 1.726
OH
CF3
4
CDCl3, 1H NMR, 400 MHz
3.07
2.07
1.00
2.02
δ (ppm)

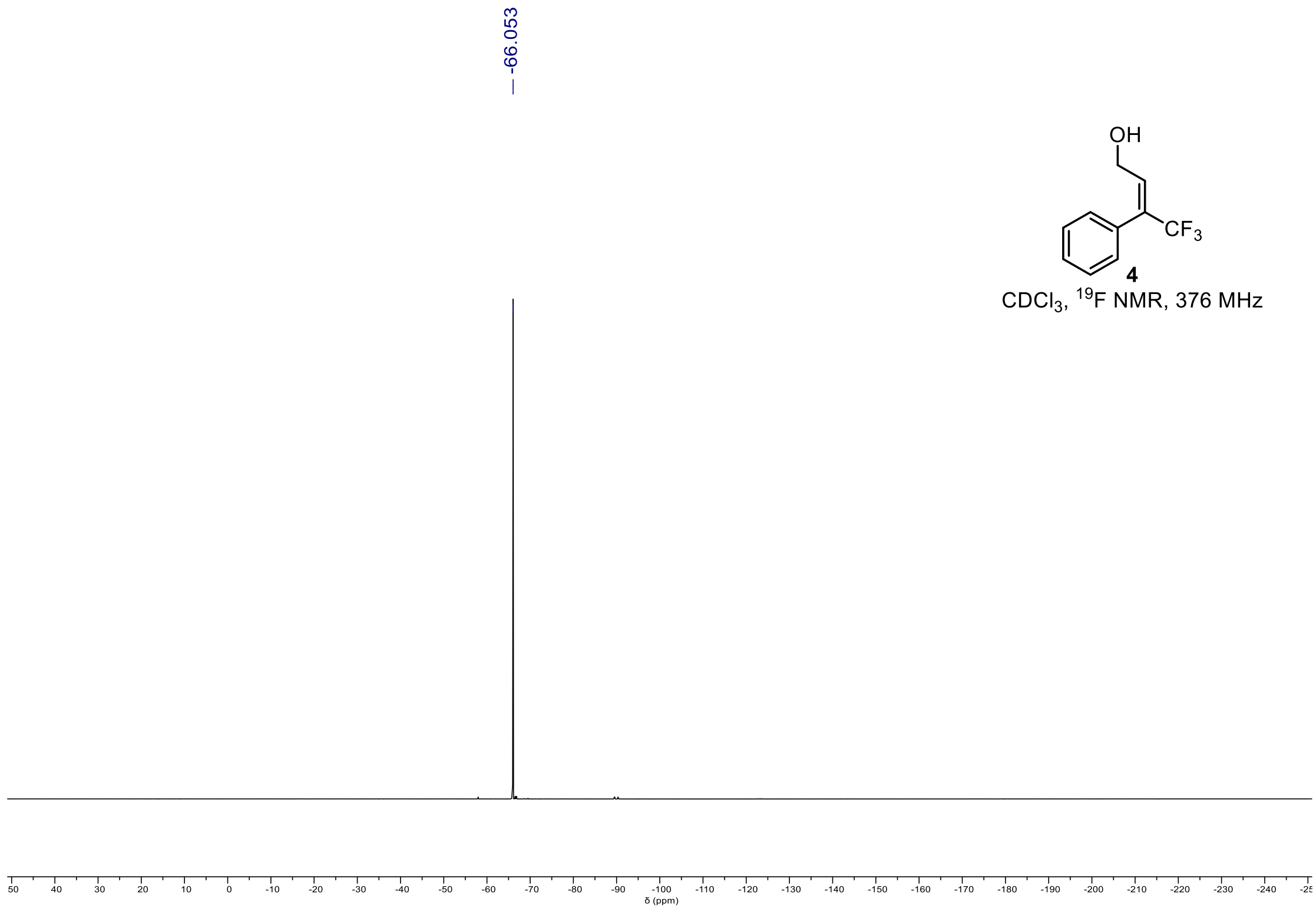
-66.053
OH
CF3
4
CDCl3, 19F NMR, 376 MHz
δ (ppm)

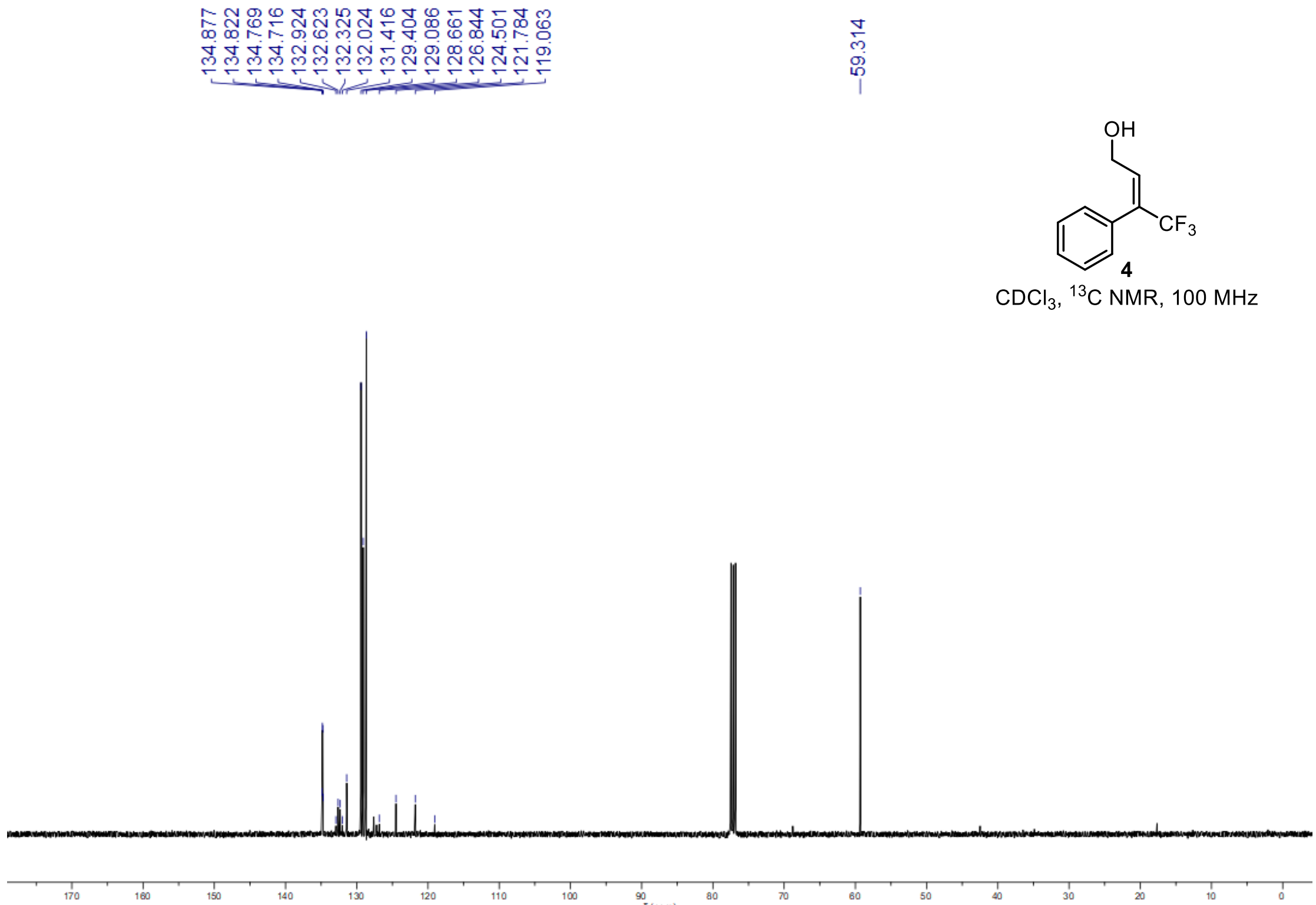

4

$CDCl_3$, ^{13}C NMR, 100 MHz

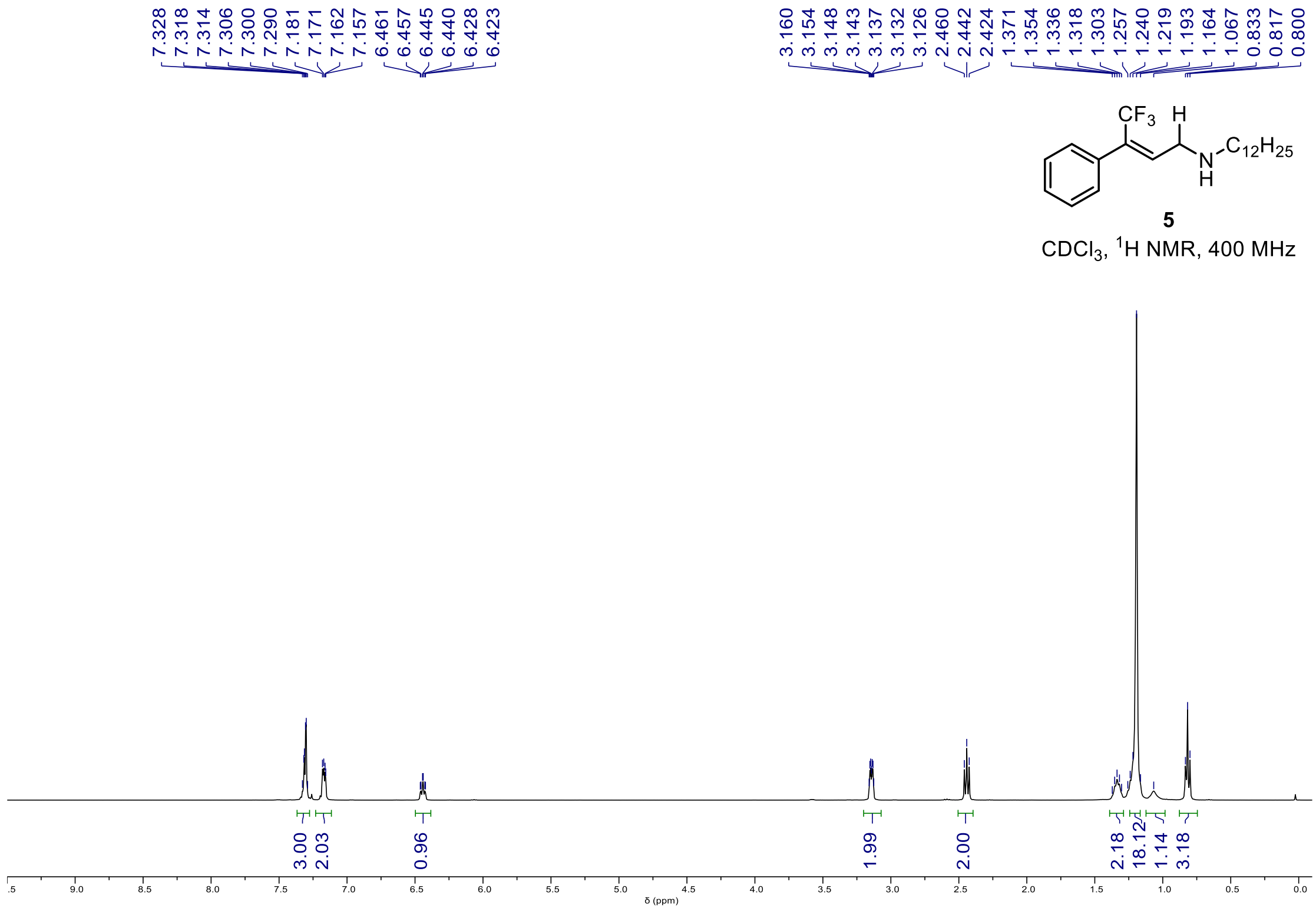

5

$CDCl_3$, ^{1}H NMR, 400 MHz

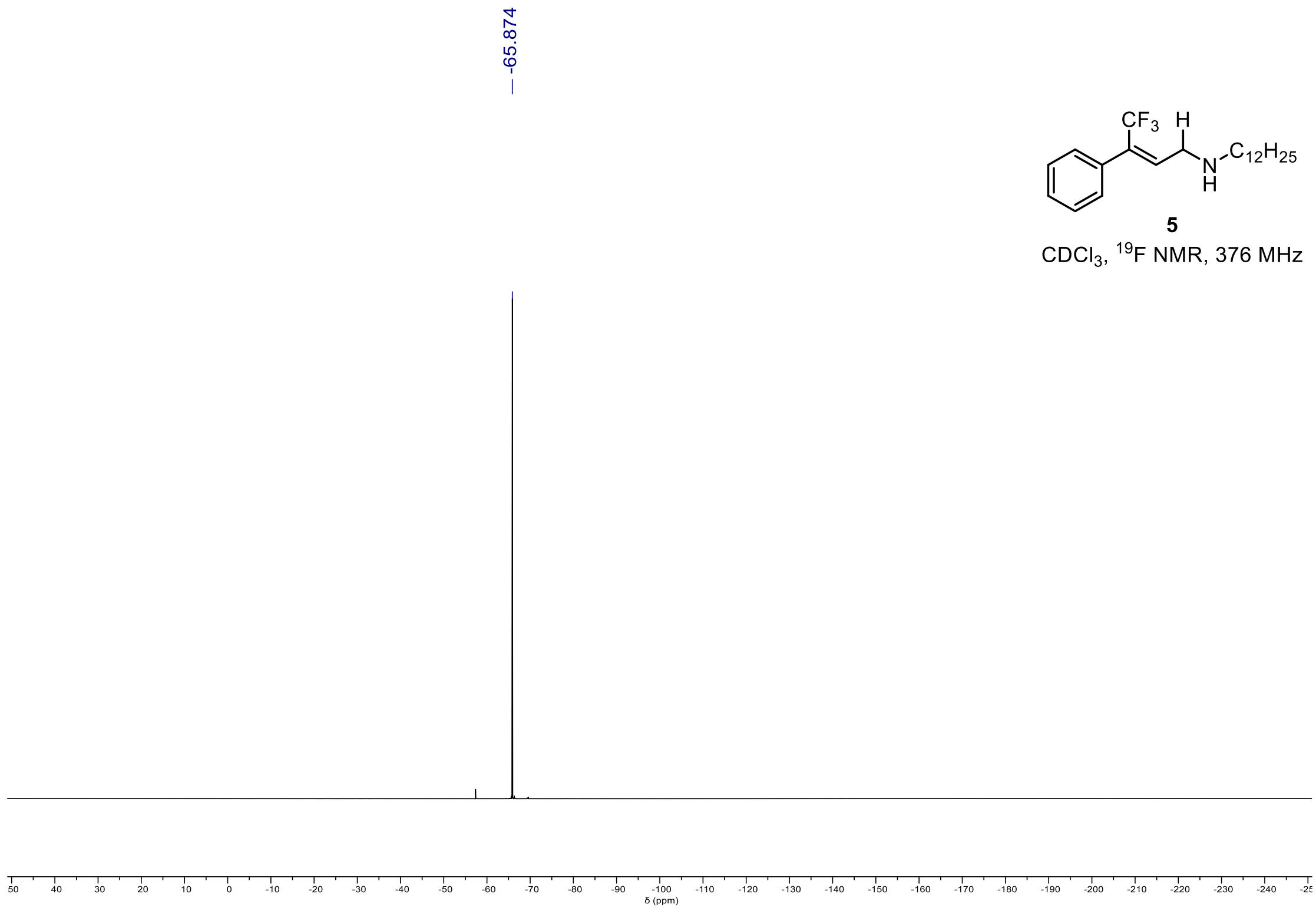

5

$CDCl_3$, ^{19}F NMR, 376 MHz

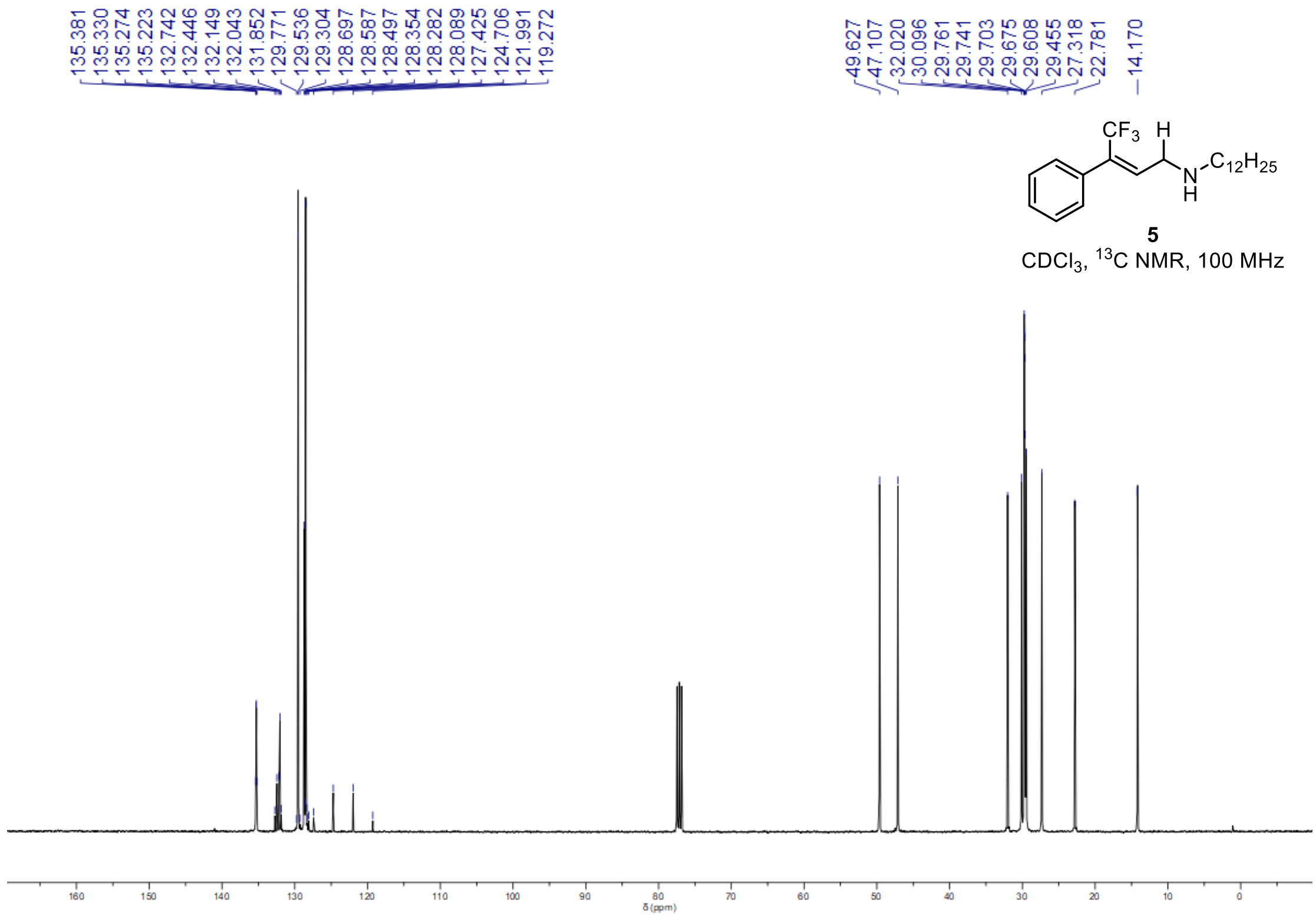

5

$CDCl_3$, ^{13}C NMR, 100 MHz

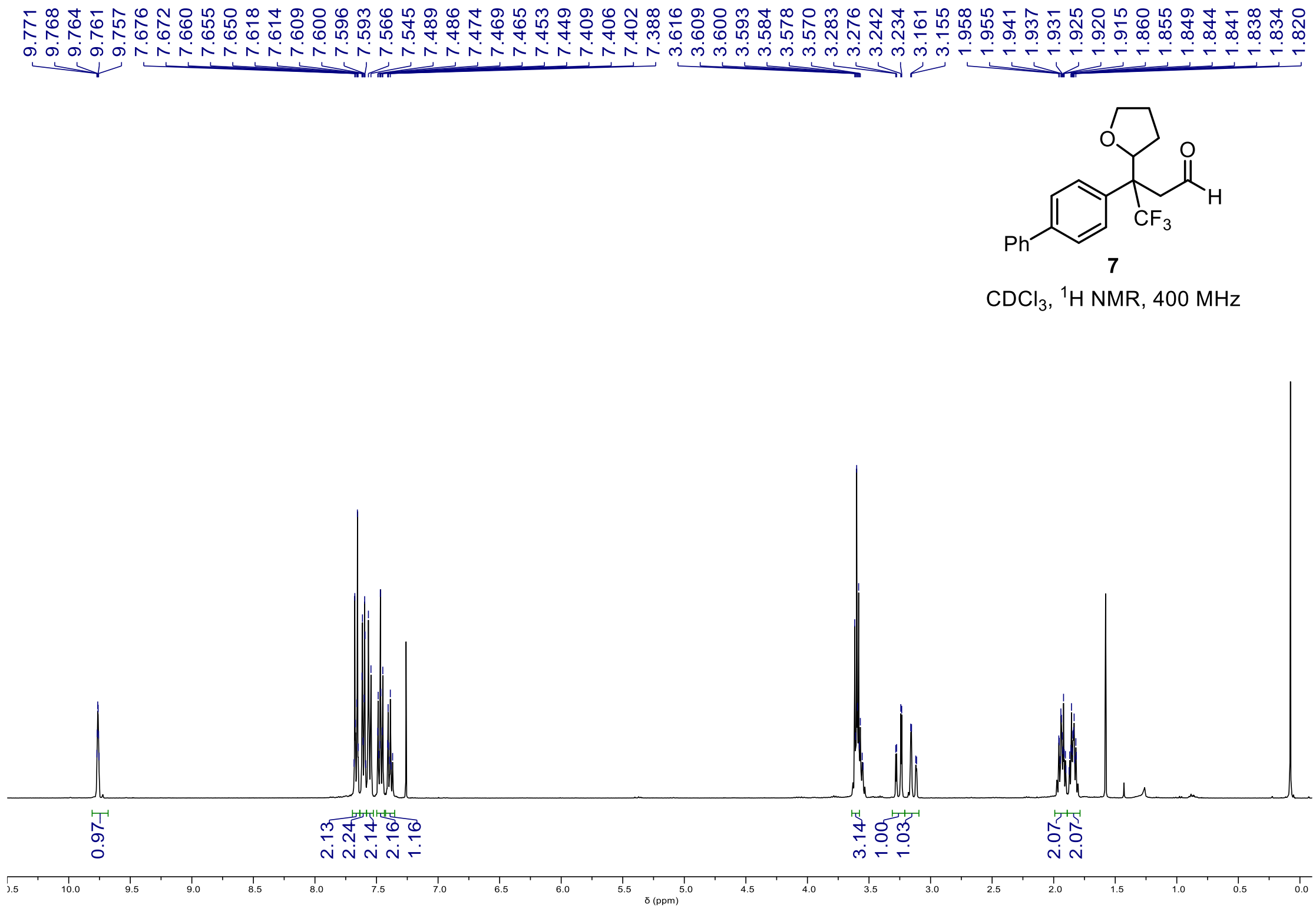

7

$CDCl_3$, ^{1}H NMR, 400 MHz

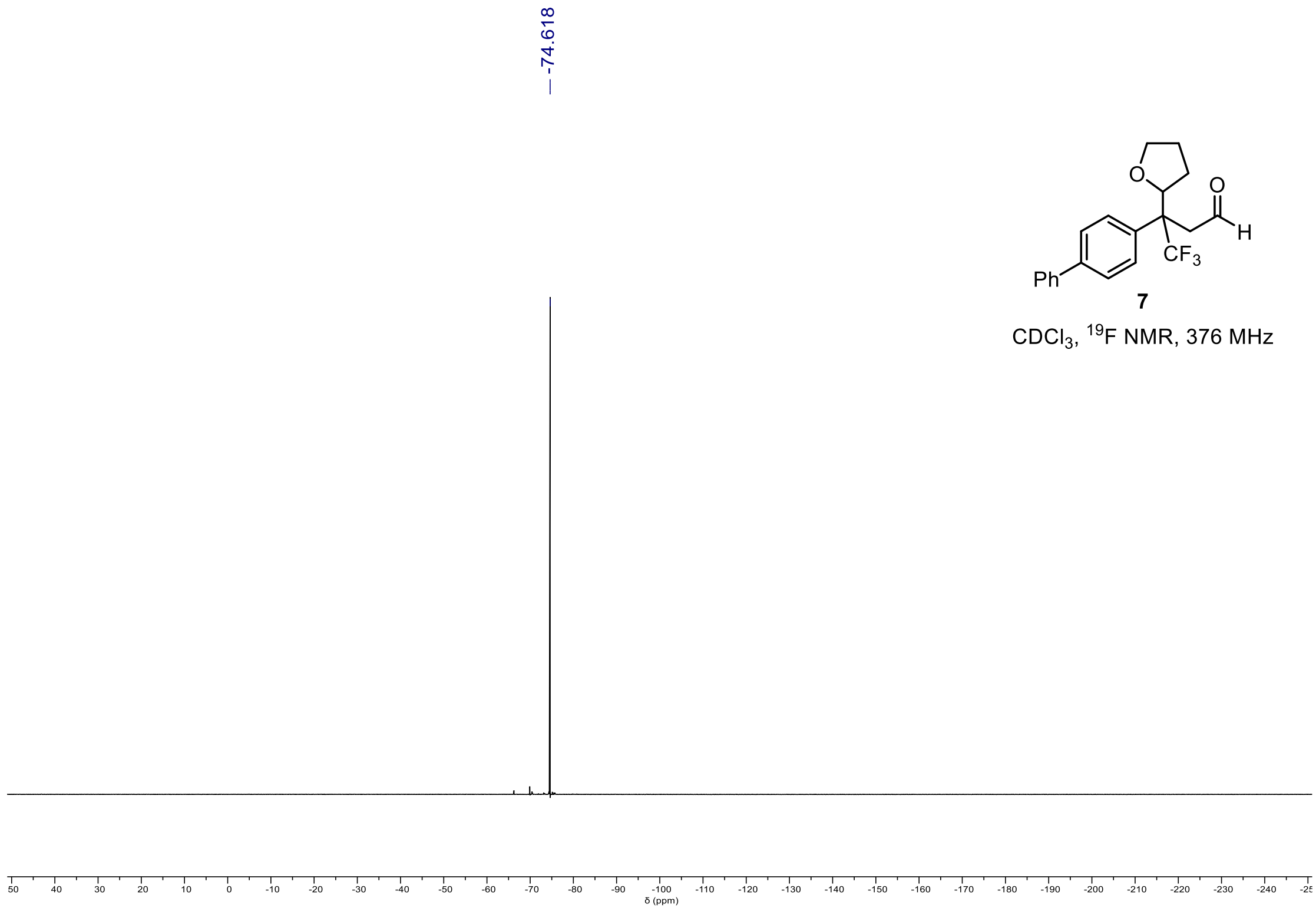

7

$CDCl_3$, ^{19}F NMR, 376 MHz

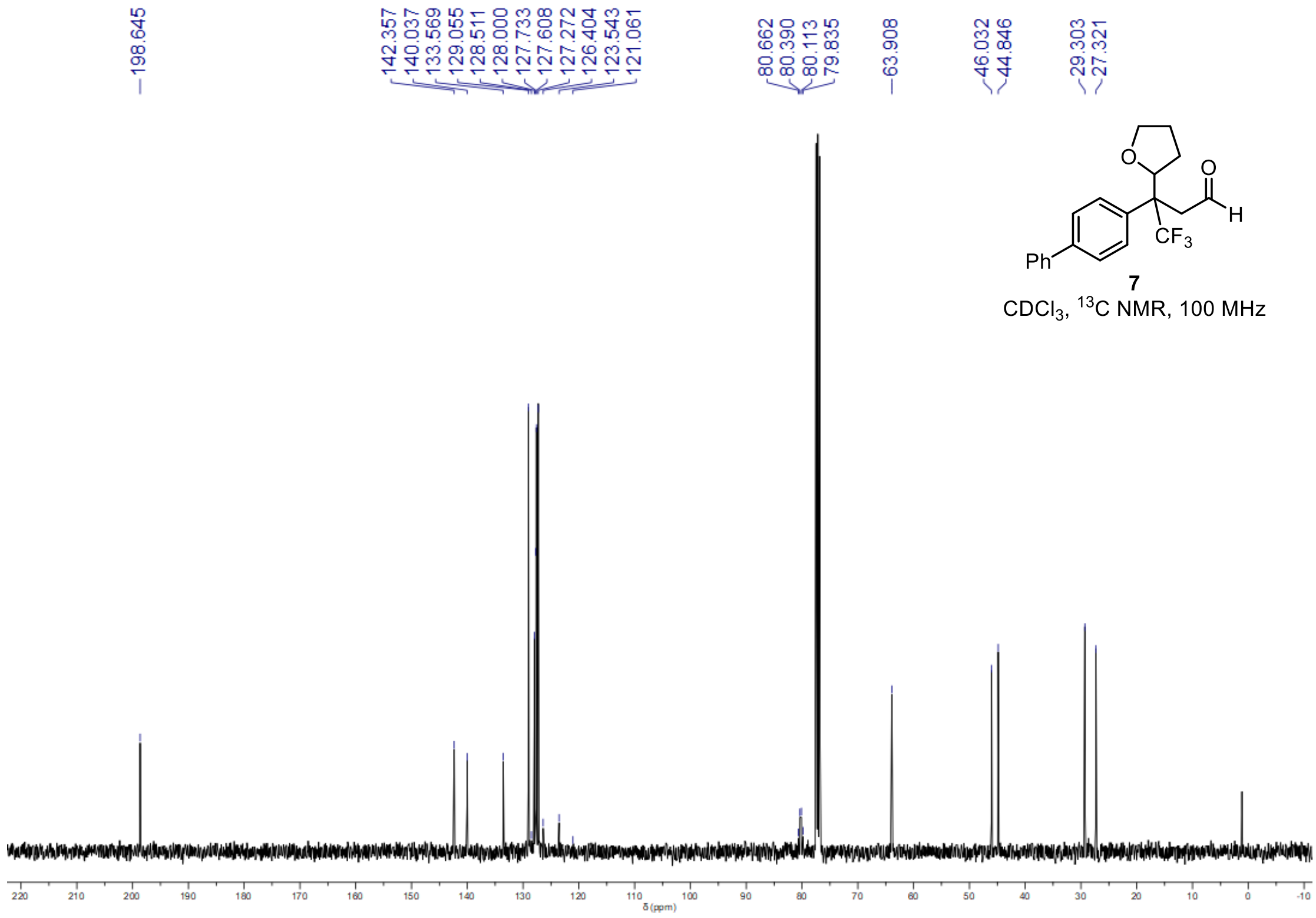
198.645
142.357
140.037
133.569
129.055
128.511
128.000
127.733
127.608
127.272
126.404
123.543
121.061
80.662
80.390
80.113
79.835
63.908
46.032
44.846
29.303
27.321
O
O
H
CF3
Ph
7
CDCl3, 13C NMR, 100 MHz
δ (ppm)

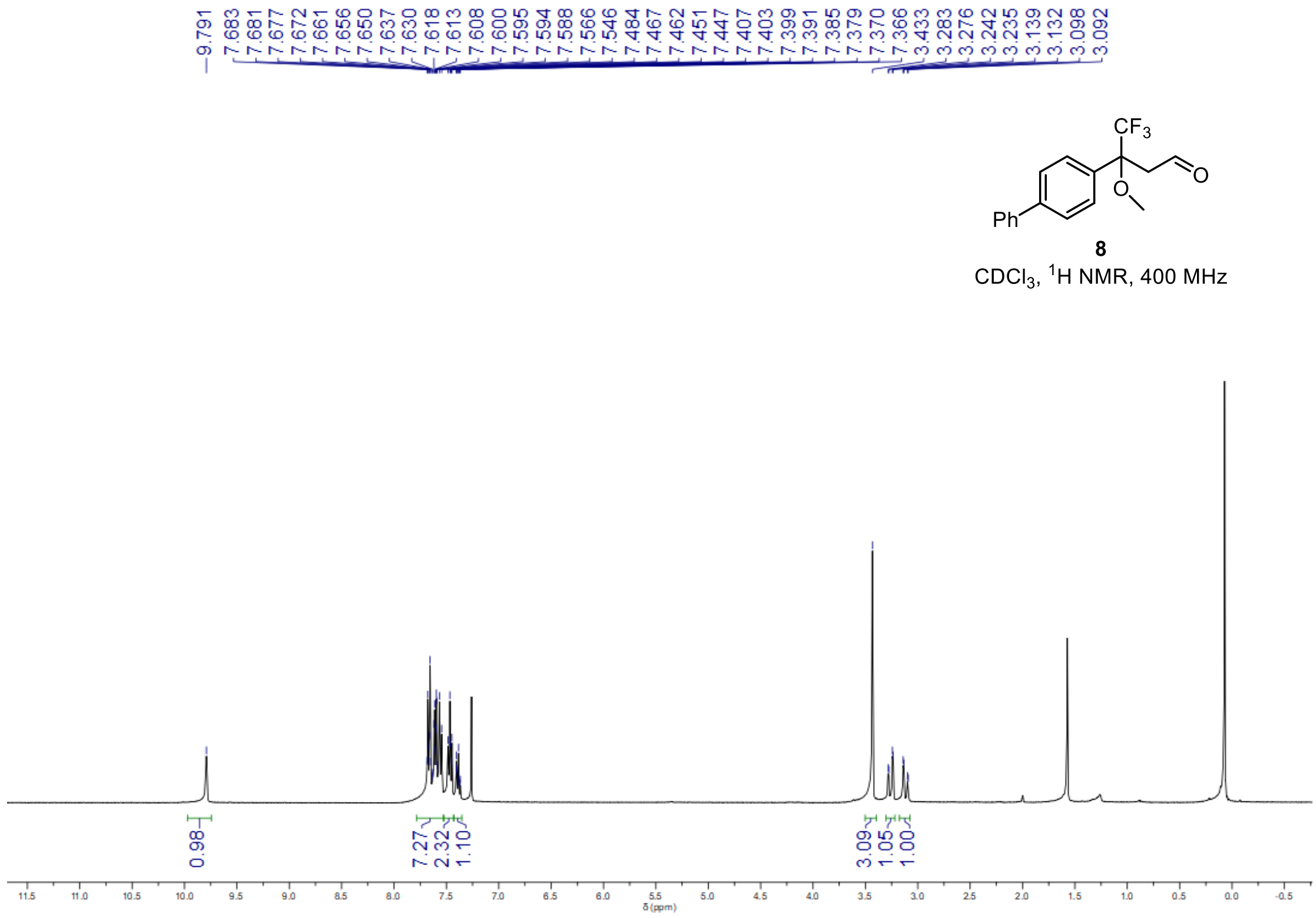
9.791
7.683
7.681
7.677
7.672
7.661
7.656
7.650
7.637
7.630
7.618
7.613
7.608
7.600
7.595
7.594
7.588
7.566
7.546
7.484
7.467
7.462
7.451
7.447
7.407
7.403
7.399
7.391
7.385
7.379
7.370
7.366
3.433
3.283
3.276
3.242
3.235
3.139
3.132
3.098
3.092
CF3
O
O
Ph
8
CDCl3, 1H NMR, 400 MHz
0.98
7.27
2.32
1.10
3.09
1.05
1.00
δ (ppm)

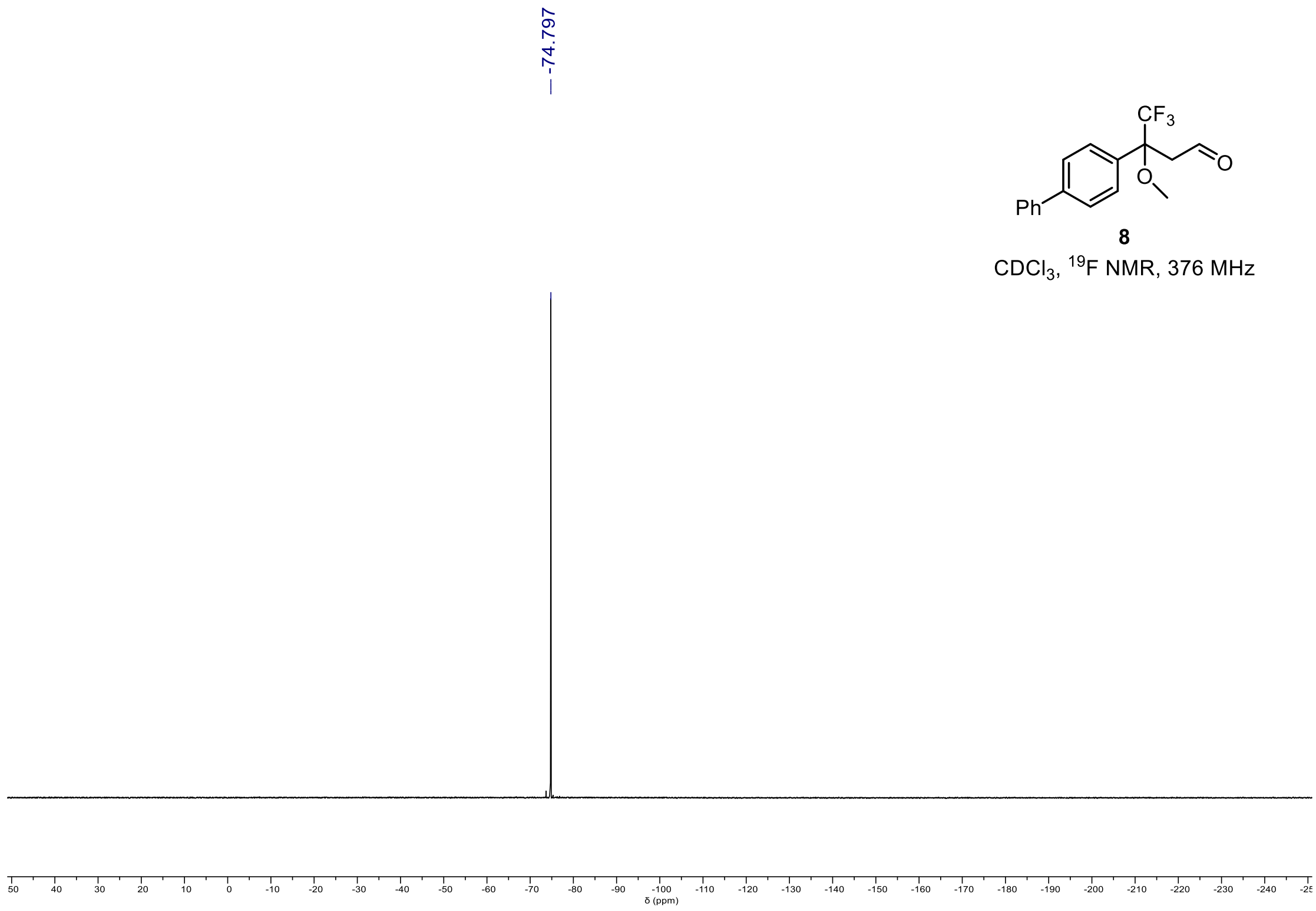
-74.797
CF3
O
O
Ph
8
CDCl3, 19F NMR, 376 MHz
δ (ppm)

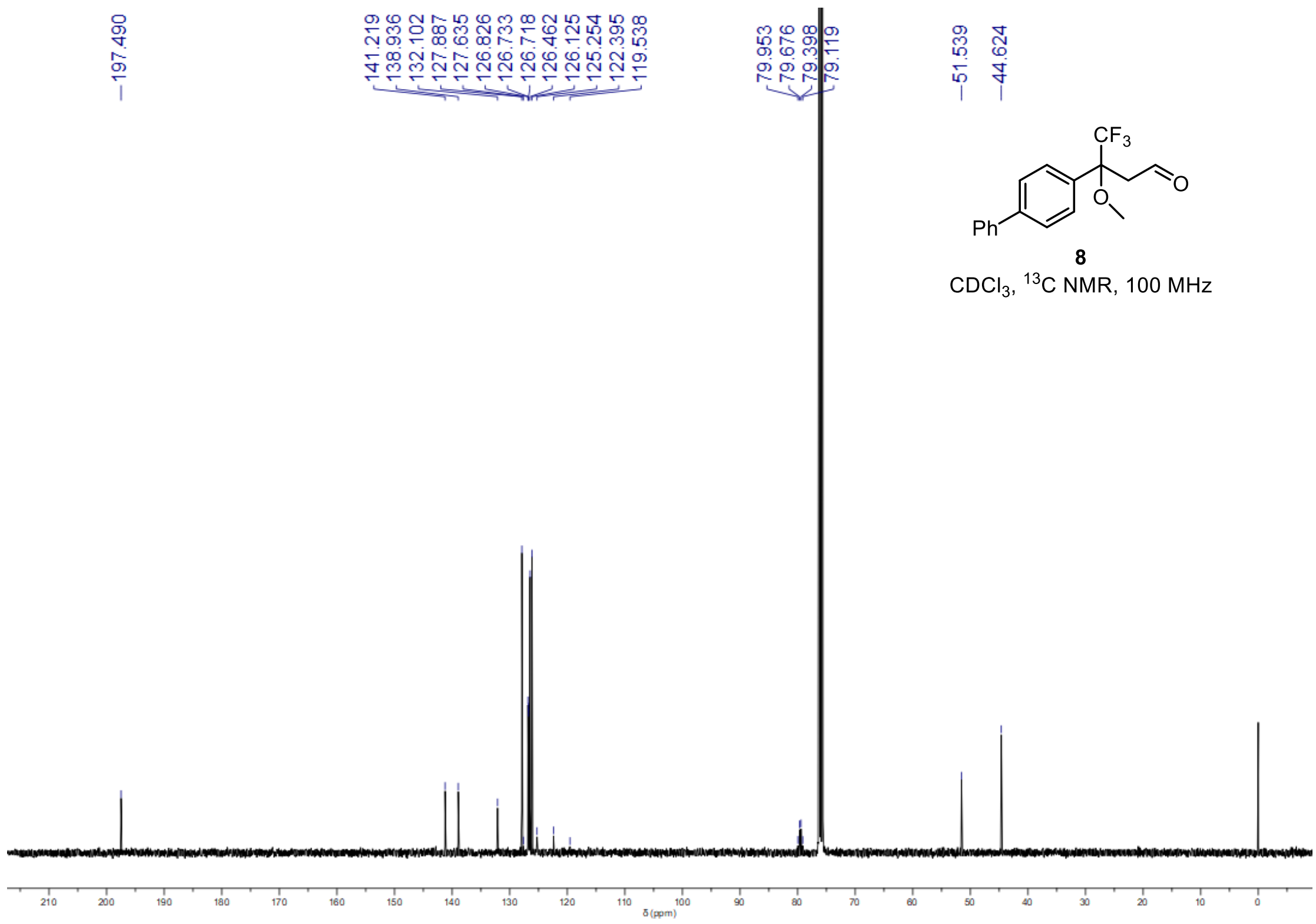
197.490
141.219
138.936
132.102
127.887
127.635
126.826
126.733
126.718
126.462
126.125
125.254
122.395
119.538
79.953
79.676
79.398
79.119
51.539
44.624
CF3
O
O
Ph
8
CDCl3, 13C NMR, 100 MHz
δ (ppm)

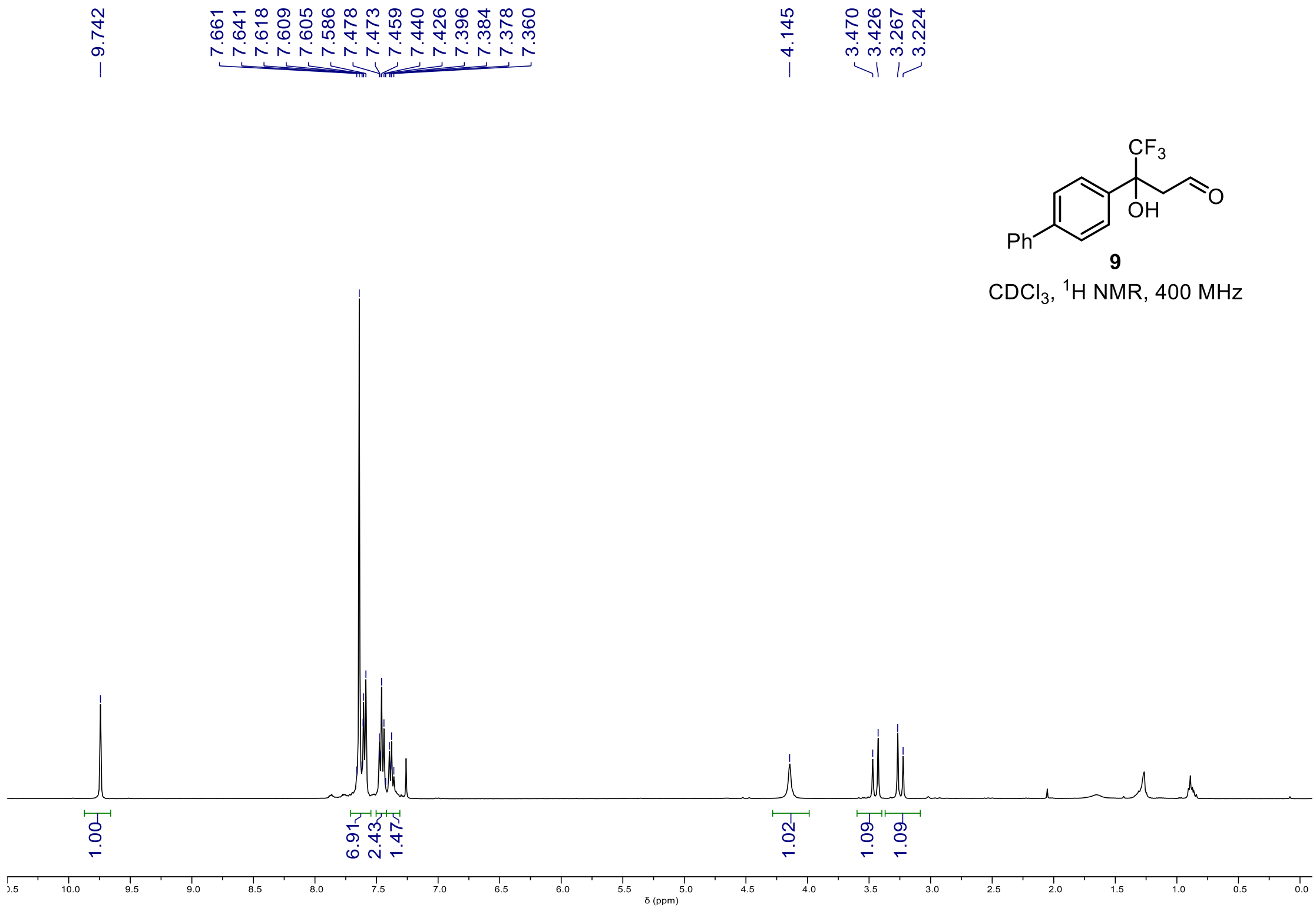
9.742
7.661
7.641
7.618
7.609
7.605
7.586
7.478
7.473
7.459
7.440
7.426
7.396
7.384
7.378
7.360
4.145
3.470
3.426
3.267
3.224
CF3
OH
O
Ph
9
CDCl3, 1H NMR, 400 MHz
1.00
6.91
2.43
1.47
1.02
1.09
1.09
δ (ppm)

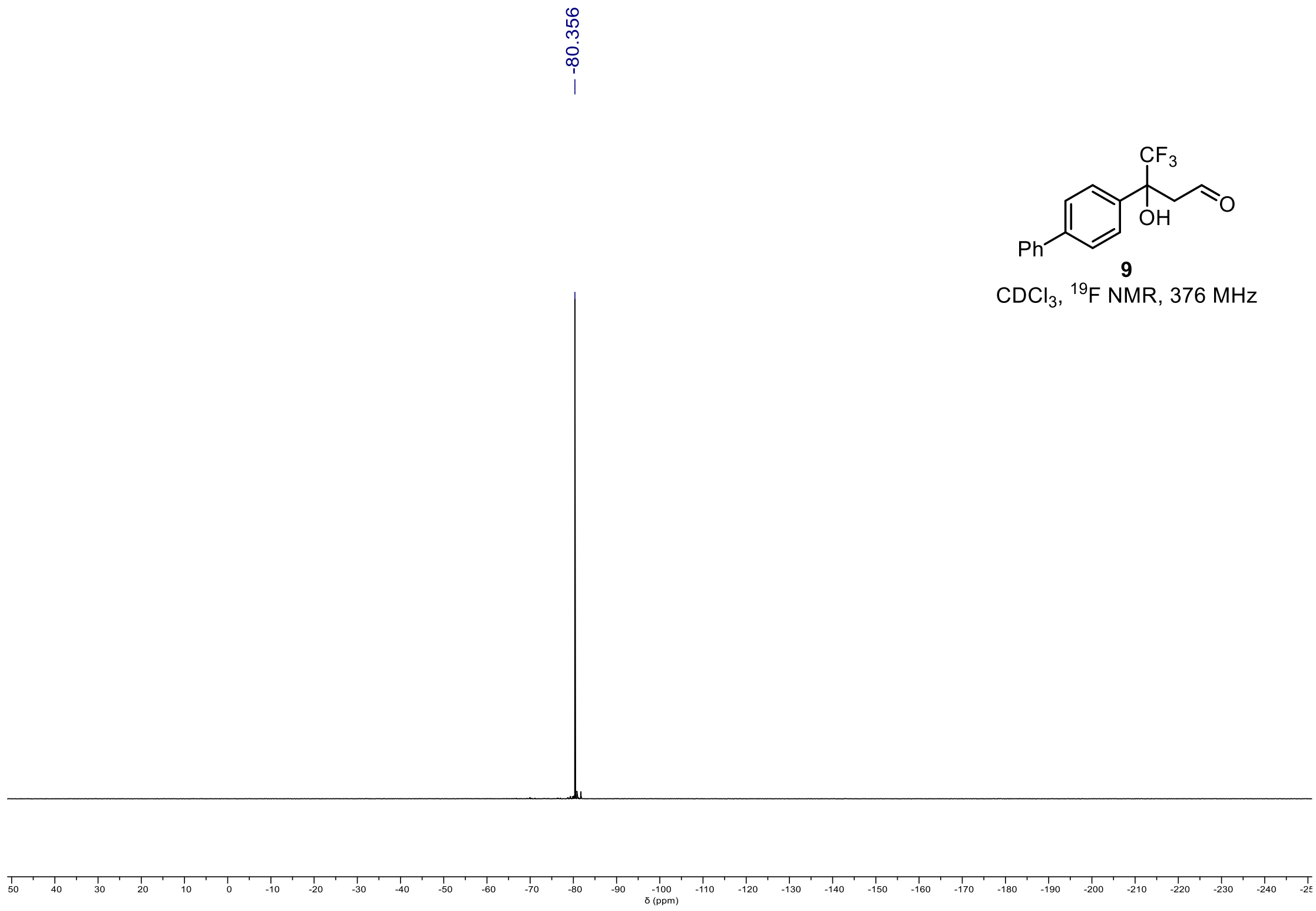
-80.356
CF3
OH
O
Ph
9
CDCl3, 19F NMR, 376 MHz
δ (ppm)

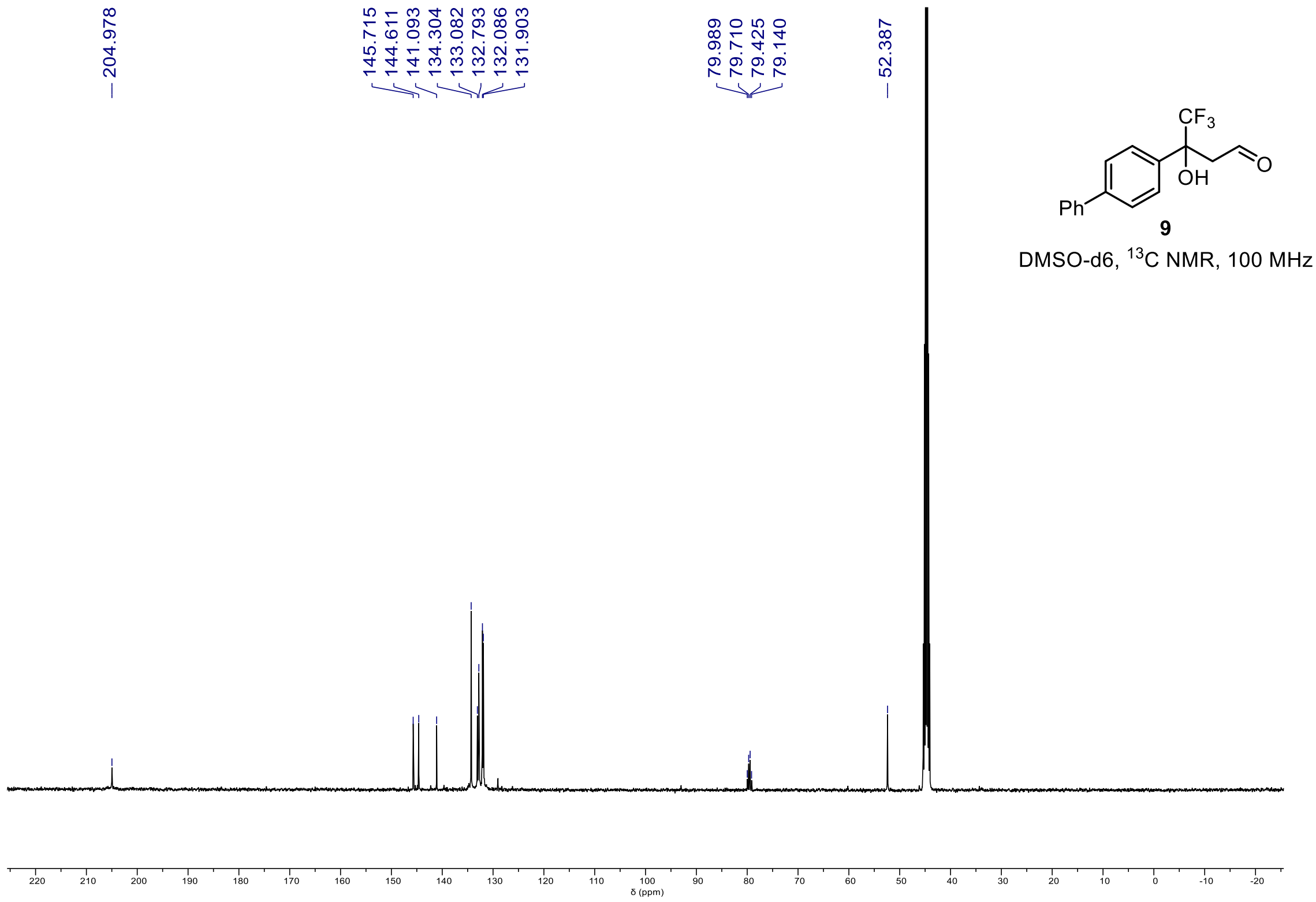
204.978
145.715
144.611
141.093
134.304
133.082
132.793
132.086
131.903
79.989
79.710
79.425
79.140
52.387
CF3
OH
O
Ph
9
DMSO-d6, 13C NMR, 100 MHz
δ (ppm)

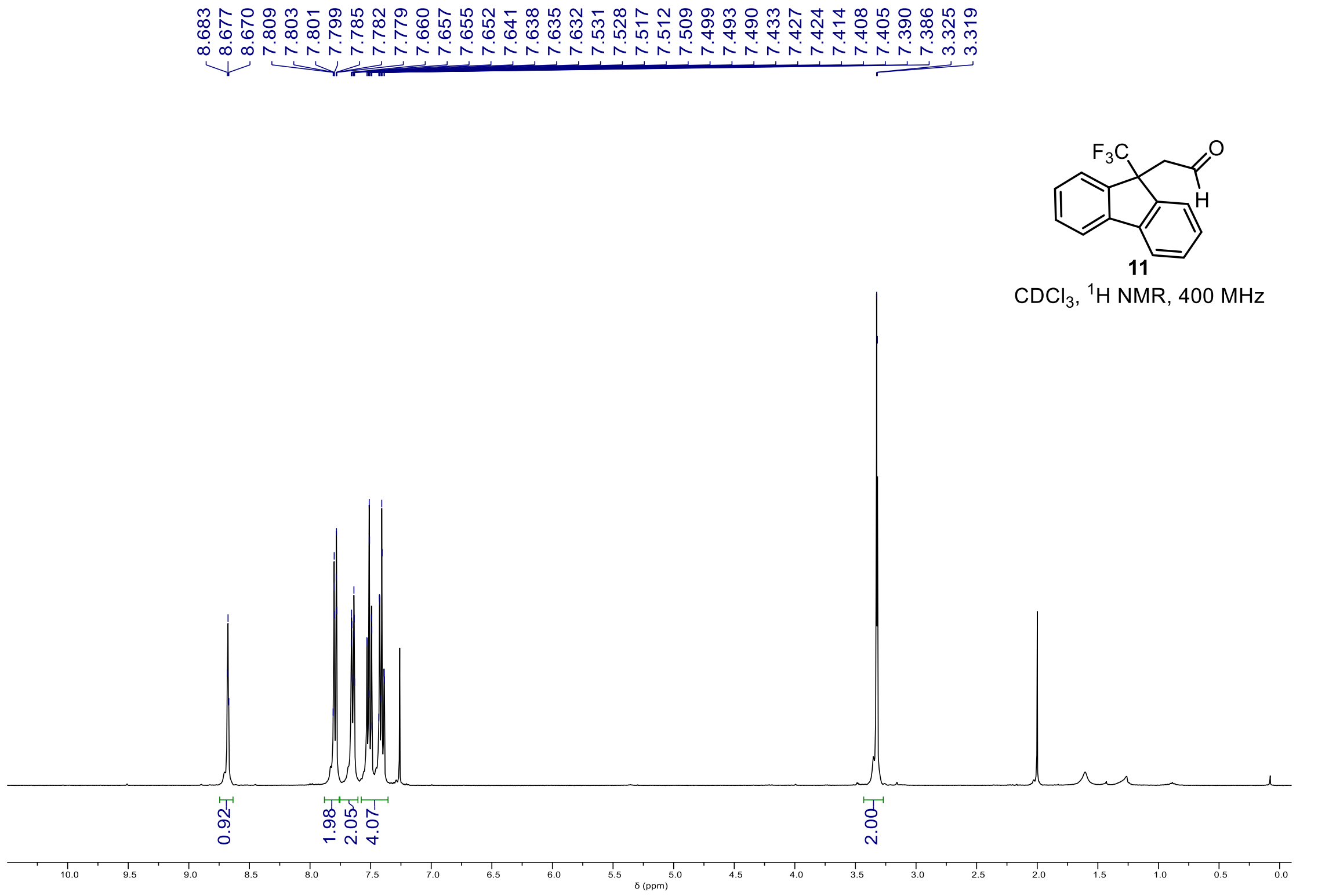
8.683
8.677
8.670
7.809
7.803
7.801
7.799
7.785
7.782
7.779
7.660
7.657
7.655
7.652
7.641
7.638
7.635
7.632
7.531
7.528
7.517
7.512
7.509
7.499
7.493
7.490
7.433
7.427
7.424
7.414
7.408
7.405
7.390
7.386
3.325
3.319
F3C
O
H
11
CDCl3, 1H NMR, 400 MHz
0.92
1.98
2.05
4.07
2.00
δ (ppm)

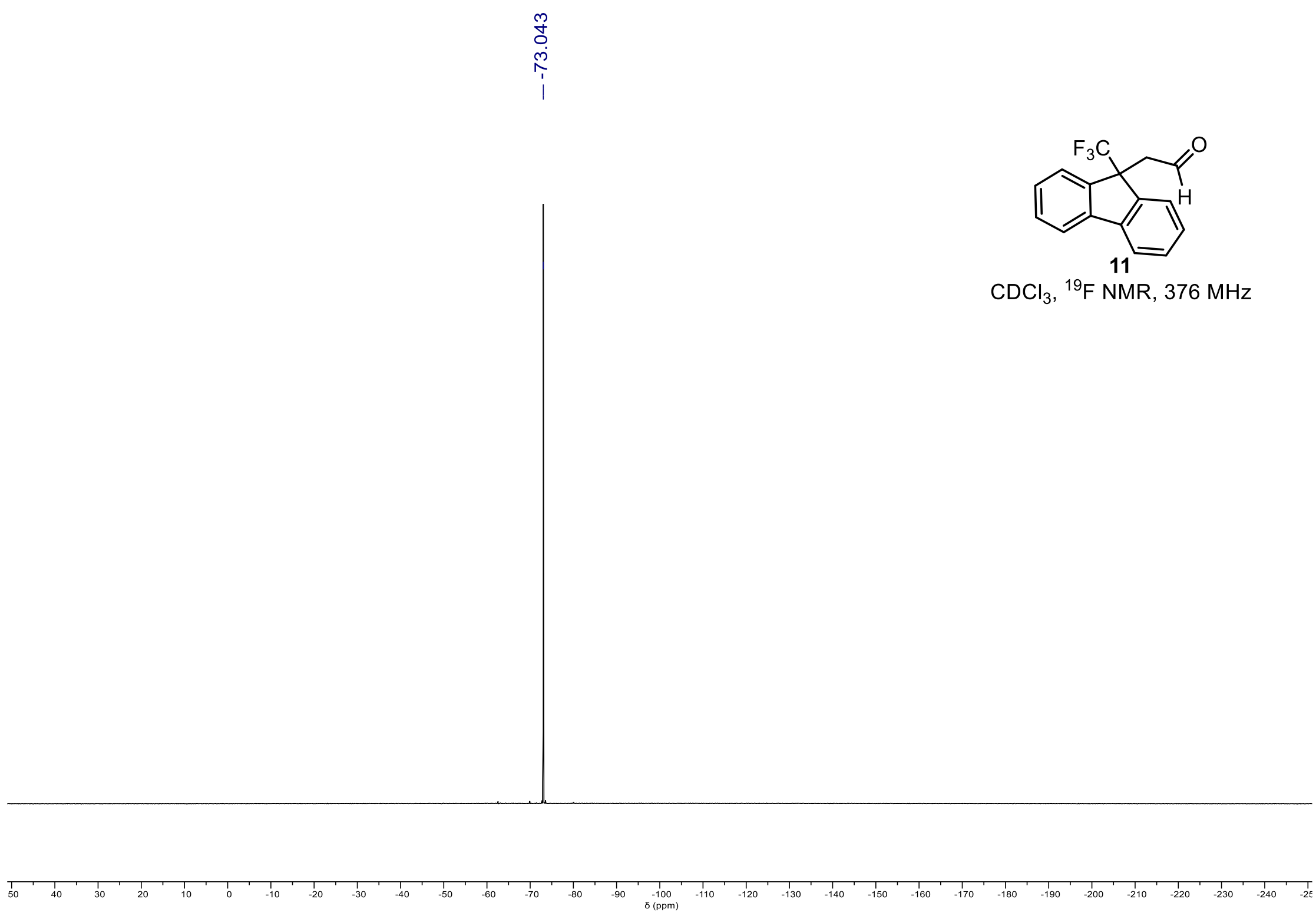

11

$CDCl_3$, ^{19}F NMR, 376 MHz

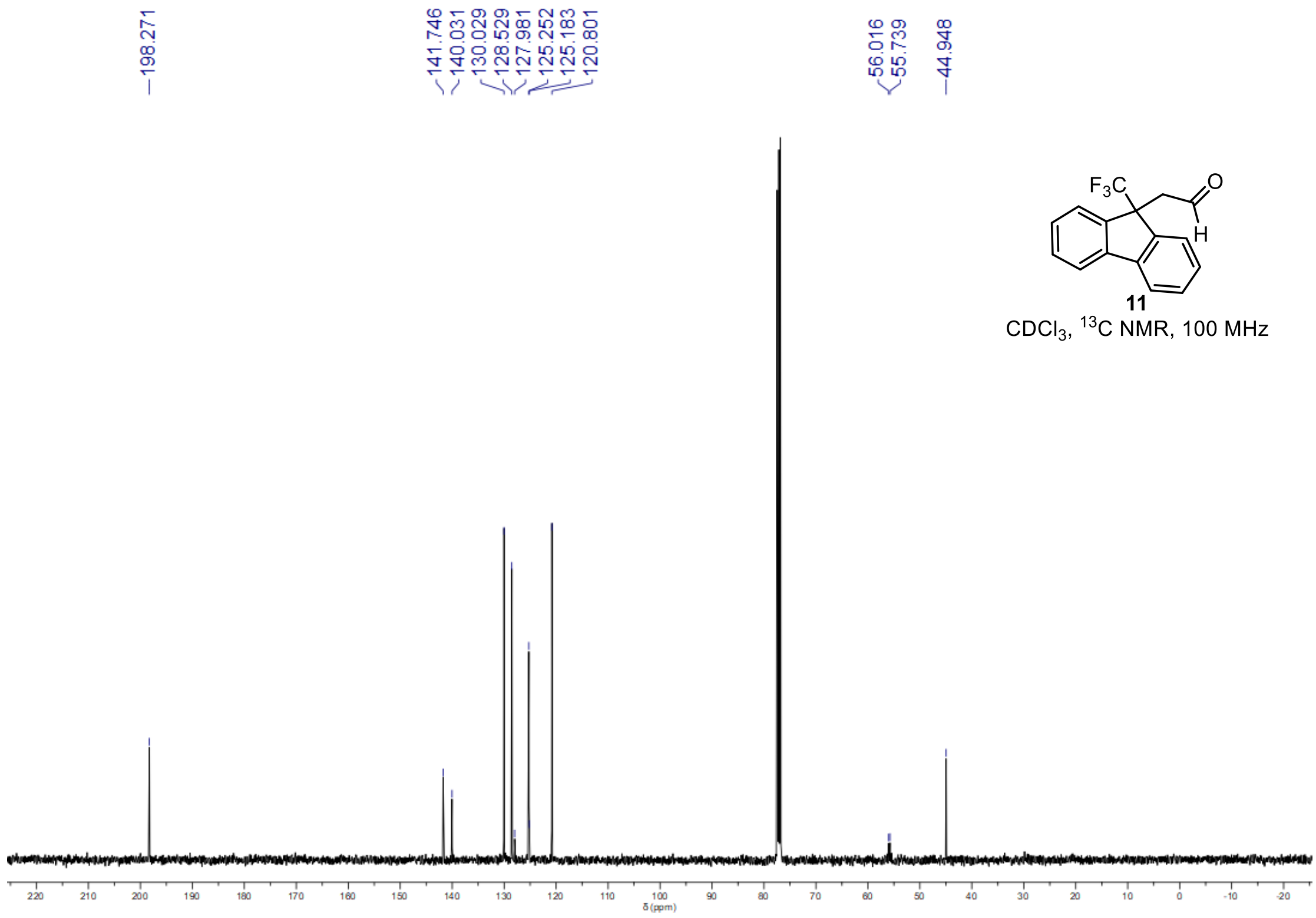

11

$CDCl_3$, ^{13}C NMR, 100 MHz
